# Is HPA axis reactivity in childhood gender-specific? A systematic review

**DOI:** 10.1186/s13293-017-0144-8

**Published:** 2017-07-11

**Authors:** Jonneke J. Hollanders, Bibian van der Voorn, Joost Rotteveel, Martijn J. J. Finken

**Affiliations:** Department of Pediatric Endocrinology, VU University Medical Center, Postbus 7057, 1007 MB Amsterdam, The Netherlands

**Keywords:** Glucocorticoid, Stress hormone, Infant, Pediatric, Sex characteristics, Stress response, Stress reaction, HPA axis, Cortisol

## Abstract

**Background:**

In adults, hypothalamus–pituitary–adrenal (HPA) axis activity shows sexual dimorphism, and this is thought to be a mechanism underlying sex-specific disease incidence. Evidence is scarce on whether these sex differences are also present in childhood. In a meta-analysis, we recently found that basal (non-stimulated) cortisol in saliva and free cortisol in 24-h urine follow sex-specific patterns. We explored whether these findings could be extended with sex differences in HPA axis reactivity.

**Methods:**

From inception to January 2016, PubMed and EMBASE.com were searched for studies that assessed HPA axis reactivity in healthy girls and boys aged ≤18 years. Articles were systematically assessed and reported in the categories: (1) diurnal rhythm, (2) cortisol awakening response (CAR), (3) protocolled social stress tests similar or equal to the Trier Social Stress Test for children (TSST-C), (4) pharmacological (ACTH and CRH) stress tests, and (5) miscellaneous stress tests.

**Results:**

Two independent assessors selected 109 out of 6158 records for full-text screening, of which 81 studies (with a total of 14,591 subjects) were included. Studies showed that girls had a tendency towards a more variable diurnal rhythm (12 out of 29 studies), a higher CAR (8 out of 18 studies), and a stronger cortisol response to social stress tests (9 out of 21 studies). We found no evidence for sex differences in cortisol response after a pharmacological challenge or to miscellaneous stress tests.

**Discussion:**

Sex differences in HPA axis reactivity appear to be present in childhood, although evidence is not unequivocal. For a better evaluation of sex differences in HPA axis reactivity, standardization of protocols and reports of stress tests is warranted.

**Electronic supplementary material:**

The online version of this article (doi:10.1186/s13293-017-0144-8) contains supplementary material, which is available to authorized users.

## Background

Marked gender differences exist in the incidence of several diseases. While men are more prone to obesity, cardiovascular disease, and infectious diseases, women are more susceptible to anxiety, depression, and autoimmune diseases. Sex-specific risks for chronic, non-communicable diseases are thought to result from a combination of genotype, phenotype, and environmental influences during life. Whereas adjustment to environmental challenges is healthy in the short term, developmental plasticity can cause sex-specific adverse effects in the long term [1].

One of the possible explanations for this sexual dimorphism in disease is a sex-specific reactivity of the hypothalamus–pituitary–adrenal (HPA) axis. HPA axis functioning can be distinguished by on the one hand the maintenance of homeostasis by controlling basal activity as well as the sensitivity to stressors and, on the other hand, coping with, adapting to, and recovery from reactions to stressors. These processes are controlled by mineralocorticoid and glucocorticoid receptors (MRs and GRs). MRs are mainly involved with basal HPA axis activity, whereas GRs predominantly regulate HPA axis reactivity [2]. In animals, receptor expression patterns appear to develop in a sex-specific manner, with sex differences already present at birth [3]. In humans, sexually dimorphic HPA axis reactivity has also been reported in adulthood: men showed a greater cortisol response to acute real-life or controlled laboratory psychological stress compared to women [4]. Additionally, cortisol responses increased with age in both men and women, but the effect was threefold stronger in women compared to men, which could possibly be attributed to menopause [5]. These patterns closely resemble those of cardiovascular disease mortality and morbidity [6]. While the setting of HPA axis functioning results from the balance between MR and GR expression [2], interactions with the hypothalamus–pituitary–gonadal (HPG) axis are thought to mediate sex-specific stress reactions as well as pathophysiology [7].

It has previously been hypothesized that disease susceptibility can originate in childhood, possibly through permanent alterations in HPA axis activity to environmental challenges [1]. We recently showed that basal HPA axis activity, represented by non-stimulated cortisol concentrations in saliva and free cortisol in 24-h urine, show sexual dimorphism, with a sex-specific change induced by puberty [8]. In addition, gender differences in the reactivity of the HPA axis have also been described in children [4, 9, 10], although evidence is scarce and not systematically reviewed. Therefore, we aimed to examine whether sex-specific differences in HPA axis reactivity are present in childhood.

To study this sex-specific reactivity of the HPA axis, we performed a systematic review of the literature. The reactivity of the HPA axis was defined as the response to either exogenous (e.g., pharmacological, physical, or social) or endogenous (e.g., cortisol awakening response (CAR)) stimuli. In addition, we included diurnal rhythm as a marker of the responsiveness of the HPA axis, although it functions differently from reactions of the HPA axis to stressors. We hypothesized that sex-specific HPA axis reactivity is already present early in life.

## Methods

### Search strategy

PubMed and Embase.com were searched from inception up to January 14, 2016 for studies addressing HPA axis reactivity in serum or saliva in boys and girls aged ≤18 years by reports of either absolute cortisol values, slopes, AUCs, and/or through visualization of the data in figures. The full search strategy is detailed in Additional file 1 and was based on the index terms or free-text words “cortisol” or “glucocorticoid,” and “sex difference” or “sexual characteristics,” and “child” or “adolescent.” We excluded studies on children with (psycho)pathology, on synthetic glucocorticoids or with a risk of abnormal HPA axis reactivity (e.g., maltreatment). We did not impose restrictions on the year of publication or study design, apart from reviews and case reports, but we did apply an English language restriction. The review protocol was based on the Preferred Reporting Items for Systematic Reviews and Meta-Analysis (PRISMA) statement.

### Data collection

Two independent assessors (BvdV and JJH) screened 6158 titles and abstracts for assessment of sex-specific HPA axis reactivity. Studies were not assessed blindly. Disagreement between assessors was discussed until consensus was reached. One hundred nine were eligible for full-text screening, of which 81 studies were included in the systematic review.

**Fig. 1:**
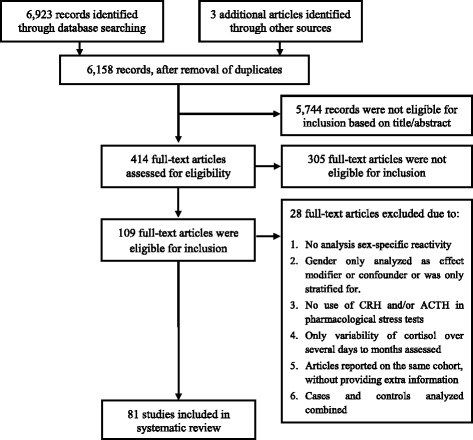
This flowchart presents the different phases of the systematic review and conforms to the PRISMA statement. (www.prisma-statement.org)

Figure 1 shows the flowchart of the search. When reports of results were unclear, the authors were contacted (*n* = 4); two authors responded. One author did not reply and one replied but could not provide sufficient data, resulting in exclusion of these studies. Additionally, articles were excluded when (1) no statistical analysis of reactivity was performed (*n* = 9); (2) pharmacological stress tests did not use corticotropin-releasing hormone (CRH) and/or ACTH (*n* = 2); (3) HPA axis reactivity was presented stratified by gender, without analyzing gender differences (*n* = 6); (4) gender was analyzed only as a confounder or effect modifier (*n* = 3); (5) analyses of sex differences were performed with cases and controls combined (*n* = 2); or (6) cortisol reactivity was defined as the variability of cortisol concentrations over several days to months (*n* = 3). Several articles reported on the same cohort. Provided that extra information was presented, all articles were included in the review. Two articles were excluded as no new information was provided compared to other articles describing the same cohort. With respect to case-control studies, we included only the control group.

### Data analysis

HPA axis reactivity was classified as follows: (1) diurnal rhythm, (2) CAR, (3) protocolled social stress tests similar or equal to the Trier Social Stress Test for children (TSST-C), (4) pharmacological (ACTH and/or CRH) tests, or (5) miscellaneous stress tests. One assessor (JJH) assessed all the articles and sorted them according to the categories above. Data were extracted from the articles and systematically summarized. If more than one type of reactivity was assessed within one article, the data were included in all applicable categories.

## Results

A short overview of all articles is presented in Tables 1, 2, 3, 4, and 5. For a more in-depth summary of the articles, see Additional file 2. Data on 14,591 subjects were included in this review, with an age range of 31 h to 18 years.

**Table 1:** Summary of articles describing sex differences in diurnal rhythmicity

Author (year)	Sample size	Age	Sampling points	Sampling medium	Results
Adam (2010)	230	17.04 ± 0.36 years	6×/day on 3 days	Saliva	Lower diurnal cortisol curves in boys
Bae (2015)	138 (70 controls)	10.7 ± 1.7 years	3×/day on 3 days	Saliva	Higher levels at awakening, 30 min after awakening, and higher total daily output in girls; levels in the evening and diurnal slope: no sex differences
Barbosa (2012)	145	8-10 yr group: 9.0 ± 0.8 years; 11-14 yr group: 11.9 ± 1.0 years	2×	Saliva	No sex differences, higher diurnal decline in children aged 11-14 years old
Bartels (2003)	360	12 years	4×/day on 2 days	Saliva	No sex differences; pubertal status not assessed
Carrion (2002)	31	Mean: 10.9 years	4×/day on 3 days	Saliva	No sex differences; pubertal status not associated with reactivity
Doom (2013)	110	9.42 ± 0.88 years	3×/day on 5 days	Saliva	No sex differences; pubertal status not assessed
Fransson (2014)	157	14–16 years	4× (including CAR)	Saliva	Steeper decline in girls
Garcia (1990)	76 (21 controls)	11.2 ± 0.37 years	3 hourly during 24 h	Blood	No sex differences; pubertal status not assessed
Haen (1984)	64	1 month to 15 years	6 hourly (4×)	Blood	No sex differences; pubertal status not assessed
Jones (2006)	140	7–9 years	5×	Saliva	No sex differences; pubertal status not assessed
Kelly (2008)	2995	15.4 ± 0.32 years	2×, 30 min apart in the morning	Saliva	Steeper decline in girls
Kjolhede (2014)	342	9.5 ± 1.9 years	3×/day on 4 days	Saliva	No sex differences; pubertal status not assessed
Knutsson (1997)	235	2.2–18.5 years	7×	Blood	No sex differences, except for higher values in girls at pubertal stage 2
Kuhlman (2015)	121	12.8 ± 2.3 years	4×/day on 2 days	Saliva	No impact of sex on cortisol at awakening or linear decline, but boys showed less deceleration of the diurnal decline between dinner and bedtime
Lumeng (2014)	331	3–4 years	3×/day on 3 days	Saliva	No sex differences; pubertal status not assessed
Martikainen (2013)	252	8.1 ± 0.3 years	7×	Saliva	Higher morning cortisol in girls; no sex difference in nadir
Matchock (2007)	120	Boys: 9, 11, or 13 years; girls: 8, 10, or 12 years	6× (including CAR)	Saliva	Cortisol peak occurred later in boys than girls during later puberty. Higher morning cortisol in boys at pubertal stage 2. AUCg: no effect of sex but significant pubertal stage effect
Michels (2012)	385	5–10 years	4× (including CAR)	Saliva	No sex differences except for somewhat steeper decline in girls (*p* = 0.30)
Morin-Major (2016)	88	14.5 ± 1.8 years	4×/day on 2 days	Saliva	Higher AUC in girls
Netherton (2004)	129	12.8 ± 0.19 years	2×/day on 4 days	Saliva	Mid-post pubertal girls have higher morning cortisol than boys. No sex differences in variance across the 4 days
Osika (2007)	84	9.9 ± 0.55 years	5× (including CAR)	Saliva	No sex differences; pubertal status not assessed
Rosmalen (2005)	1768	11.08 ± 0.55 years	3× (including CAR)	Saliva	Higher morning cortisol levels in girls, no sex differences in evening cortisol, already present in prepubertal children. Age or pubertal status not associated with cortisol levels
Ruttle (2013)	346	11, 13, and 15 years	3×/day on 3 days	Saliva	Steeper slope in girls at ages 11 and 13 and in longitudinal analyses; higher cortisol levels in girls throughout the day at age 15
Shirtcliff (2012)	357	9, 11, 13, and 15 years	3×/day on 3 days	Saliva	Steeper slopes, more curvature in girls. Advancement through puberty: rhythm becomes flatter, especially in girls
Susman (2007)	111	Boys: 9, 11, or 13 years; girls: 8, 10, or 12 years	6× (including CAR)	Saliva	No sex differences; pubertal status not associated with reactivity
Tzortzi (2009)	21	10–14 years	20× (including CAR)	Saliva	No sex differences; pubertal status not assessed
Vaillancourt (2008)	154	147 ± 9.07 months	2×/day on 3 days	Saliva	Higher morning levels in girls on Saturday, multilevel regression: consistently higher production in girls
Vanaelst (2013)	355	5–10 years	4×/day on 2 days (including CAR)	Saliva	No sex differences; pubertal status not assessed
Williams (2013)	27	9.13 ± 1.41 years	3×/day on 2 days (including CAR)	Saliva	Boys exhibited flatter slopes than girls

**Table 2:** Summary of articles describing sex differences in cortisol awakening response (CAR)

Author (year)	Sample size	Age	Sampling points	Sampling medium	Results
Adam (2010)	230	17.04 ± 0.36 years	0 and 40 min after awakening	Saliva	No sex differences; pubertal status not assessed
Bae (2015)	138 (70 controls)	10.7 ± 1.7 years	0 and 30 min after awakening	Saliva	Higher levels in girls at awakening and 30 min after awakening, no sex differences in awakening response
Bouma (2009)	644	16.13 ± 0.59 years	0 and 30 min after awakening	Saliva	Higher basal levels in girls, no difference in awakening responses
Bright (2014)	47	12–24 months	0 and 30 min after awakening	Saliva	No sex differences; pubertal status not assessed
Dietrich (2013)	1604	11.1 ± 0.55 years	0 and 30 min after awakening	Saliva	AUCg and absolute cortisol values higher in girls, AUCi no sex differences
Fransson (2014)	157	14–16 years	0, 30, and 60 min after awakening	Saliva	Higher CAR in girls
Hatzinger (2007)	102	4.91 ± 0.44 years	0, 10, 20, and 30 min after awakening	Saliva	Higher CAR in girls
Jones (2006)	140	7–9 years	0 and 30 min after awakening	Saliva	CAR present in boys, not in girls
Kuhlman (2015)	121	12.8 ± 2.3 years	0 and 45 min after awakening	Saliva	No sex differences; pubertal status not assessed
Martikainen (2013)	252	8.1 ± 0.3 years	0, 15, and 30 min after awakening	Saliva	Higher AUCg in girls, same increase and AUCi
Michels (2012)	385	5–10 years	0, 30, and 60 min after awakening	Saliva	No sex differences; pubertal status not assessed
Morin-Major (2016)	88	14.5 ± 1.8 years	0 and 30 min after awakening	Saliva	Correlated to sex, higher CAR in girls
Osika (2007)	84	9.9 ± 0.55 years	0 and 15 min after awakening	Saliva	No sex differences; pubertal status not assessed
Pruessner (1997)	42	11.16 ± 1.99 years	On 3 days: 0, 10, 20, and 30 min after awakening	Saliva	Marginal differences: higher in girls
Susman (2007)	111	Boys: 9, 11, or 13 years; girls: 8, 10, or 12 years	0, 20, and 40 min after awakening	Saliva	No sex differences; pubertal status not associated with reactivity
Tzortzi (2009)	21	10–14 years	From waking: every 20 min until 3 h after awakening	Saliva	No sex differences; pubertal status not assessed
Vanaelst (2013)	355	5–10 years	0, 30, and 60 min after awakening	Saliva	No sex differences; pubertal status not assessed
Williams (2013)	27	9.13 ± 1.41 years	0 and 30 min after awakening	Saliva	No sex differences; pubertal status not assessed

**Table 3:** Summary of articles describing sex differences in protocolled social stress test similar or equal to the TSST-C

Author (year)	Sample size	Age	Sampling points	Sampling medium	Results
Bae (2015)	169 (81 controls)	10.8 ± 1.8 years	8× (3 before, 5 after)	Saliva	No sex differences; pubertal status not associated with reactivity
Bouma (2009)	644	16.13 ± 0.59 years	5× (2 before, 3 after) (Groningen Social Stress Test)	Saliva	Cortisol responses were stronger in boys
Bouma (2011)	553	16.07 ± 0.90 years	4× (1 before, 3 after) (Groningen Social Stress Test)	Saliva	Boys had higher cortisol levels on sample 2
De Veld (2012)	158	10.61 ± 0.52 years	7× (2 before, 5 after)	Saliva	Cortisol response stronger in girls
Dockray (2009)	111	Boys: 9, 11, or 13 years; girls: 8, 10, or 12 years	5×, 2 before, 3 after	Saliva	No sex differences; age but not pubertal stage associated with reactivity in girls, no associations in boys.
Evans (2013)	707	13.77 ± 3.56 years	After each period/task, at the middle of the documentary, and at the end of it (in figure 2: 6 samples, 2 before, 4 during/after) (social stress tests based on TSST)	Saliva	In children (7–12): lower cortisol reactivity in boys experiencing less emotional warmth
					Adolescents (13–20): no sex differences
Gunnar (2009)	82	Four age groups: 9 (9.79 ± 0.16), 11 (11.57 ± 0.15), 13 (13.55 ± 0.46), and 15 (15.55 ± 0.47)	10×, 3 before, 7 after	Saliva	No sex differences, except higher cortisol reactivity in girls at age 13
Hostinar (2014)	191	14.4 ± 1.93 years	6× (2 before, 4 after) (TSST for groups)	Saliva	No sex differences; higher intercepts and greater anticipatory responses with increasing age, pubertal status not assessed
Hostinar (2015)	81 (40 children, 41 adolescents)	Children: 9.97 ± 0.52 years; adolescents: 16.05 ± 0.39 years	4× (1 before, 3 after)	Saliva	Stronger response in 9–10-year old girls, no sex differences among adolescents
Ji (2016)	135	Boys: 9, 11, or 13 years; girls: 8, 10, or 12 years	5× (2 before, 3 after)	Saliva	At wave 3 (each wave separated by 6 months): girls have stronger reaction to stressor; no sex differences in recovery
Jones (2006)	140	7–9 years	7× (3 before, 4 after)	Saliva	Anticipatory rise in both, further increment in girls
Kudielka (2004)	31	12.1 ± 0.3 years	5×, 1 before, 4 after	Saliva	No sex differences; pubertal status not assessed
Lu (2014)	87	12.7 ± 0.3 years	9×, not specified when	Saliva	More negative logAUCi in girls (less increase)
Martikainen (2013)	252	8.1 ± 0.3 years	7× (2 before, 5 after)	Saliva	Higher peak, AUCg, and AUCi in girls
Martin (2011)	40	16–18 years	7× (1 before, 6 after)	Saliva	No sex differences; pubertal status not assessed
Mrug (2016)	84	13.36 ± 0.95 years	3×, 1 before, 2 after	Saliva	Higher post-test cortisol and AUCi in girls
Peckins (2012)	124	10.49 ± 1.68 years; boys: 9, 11, or 13 years; girls: 8, 10, or 12 years	5×, 2 before, 3 after	Saliva	No sex differences; pubertal status not associated with reactivity
Portnoy (2015)	446	11.92 ± 0.59 years	4×, 1 before, 3 after	Saliva	No sex differences in AUCg; pubertal status not associated with reactivity
Raikkonen (2010)	292	8.1 ± 0.3 years	7× (2 before, 5 after)	Saliva	Boys lower than girls
Strahler (2010)	62	6–10 years	4×, 1 before, 3 after	Saliva	No sex differences; pubertal status not assessed
Trickett (2014)	303 maltreated, 151 control	Maltreated: 10.84 ± 1.16 years; comparison: 11.11 ± 1.15 years	6× (2 before, 4 after)	Saliva	Cortisol response blunted in girls compared to boys

**Table 4:** Summary of articles describing sex differences in pharmacological stress tests

Author (year)	Sample size	Age	Study protocol	Sampling points	Sampling medium	Results
Dahl (1992)	25	10.3 ± 1.6 years	CRH challenge: 1 μg/kg i.v. in the late afternoon	9×, 3 before, 6 after	Blood	Greater peak in boys
Dorn (1996)	20 control subjects	15.1 ± 1.0 years	CRH challenge: 1 μg/kg i.v. in the evening	12×, 6 before, 6 after	Blood	No sex differences; groups matched for pubertal status, effect not analyzed
Forest (1978)	20 infants, 35 prepubertal children	Infants: 5–365 days; children: 1–12.6 years	ACTH test: 500 μg/m^2^ i.m. at 8:00 and 20:00 on 3 days	2×, 1 before, 1 after	Blood	No sex differences; pubertal status not assessed
Lashansky (1991)	102	2 months–17 years	ACTH test: 0.25 mg i.v. in the morning	2×, 1 before, 1 after	Blood	No sex differences; decrease in stimulated cortisol levels with puberty, more pronounced in boys
Ross (1986)	21	6–15 years	CRH challenge: 1 μg/kg i.v. in the evening	7×, 2 before, 5 after	Blood	No sex differences; pubertal status not associated with reactivity
Stroud (2011)	68	11.6 ± 1.9 years	CRH challenge: 1 μg/kg i.v. in the late afternoon	9–10×, 3 before, 6–7 after	Blood	Sex by Tanner differences: girls increase and boys decrease in cortisol with pubertal maturation, girls decrease and boys are stable in reactivity. Boys have larger peak change
Tsvetkova (1977)	31	4–14 years	ACTH test: 0.5 mg i.m. in the morning	2×, 1 before, 1 after	Blood	No sex differences; pubertal status not assessed

**Table 5:** Summary of articles describing sex differences in miscellaneous stress tests

		Author (year)	Sample size	Age	Study protocol	Sampling points	Sampling medium	Results
0–1 year old		Davis (1995)	36	30.99 ± 8.09 h	Neonatal Behavior Assessment Scale	5×, 1 before, 4 after test	Saliva	Higher reactivity in boys
		Eiden (2015)	217	9 months	Laboratory Temperament Assessment Battery	4×, 1 before, 3 after test	Saliva	Cortisol increase in boys, not in girls
		Grunau (2010)	32	4.2 ± 1.0 months	Cortisol response after vaccination	3×, 1 before, 2 after	Saliva	No sex differences; pubertal status not assessed
1–7 years old		De Weerth (2013)	42	68.0 ± 4.3 months	CREST paradigm	6× (2 before, 4 after)	Saliva	No sex differences; pubertal status not assessed
		Gunnar (2010)	151	3.81 ± 0.23 years	Daycare attendance	2×/day on 2 days	Saliva	No sex differences; pubertal status not assessed
		Hatzinger (2007)	102	4.91 ± 0.44 years	MSSB	5× (2 before, 3 after)	Saliva	Higher reactivity in girls
		Kryski (2013)	409	40.72 ± 3.51 months	Matching task	6× (1 before, 5 after)	Saliva	No sex differences; pubertal status not assessed
		Mills (2008)	214	4.14 ± 0.24 years	Easy and difficult matching tasks	6×, 1 before, 5 after	Saliva	Further decreases in boys after initial decrease for both sexes
		Plusquellec (2011)	376	18.85 ± 0.74 months	Two unfamiliar situations (clown and robot)	2×, 1 before, 1 after	Saliva	No sex differences; pubertal status not assessed
		Spinrad (2009)	84	54.07 ± 0.97 months	Preschool Laboratory Assessment Battery	3×, 1 before, 2 after	Saliva	No sex differences; pubertal status not assessed
		Yong Ping (2014)	94	29.9 ± 1.1 months	Maternal separation	4× (2 before, 2 after)	Saliva	No sex differences; pubertal status not assessed
≥7 years old	Psychological stress	Daughters (2013)	132	16.1 ± 1.0 years	Behavioral Indicator of Resiliency to Distress	4×, 1 before, 3 after	Saliva	Boys: higher baseline, greater peak. No sex differences in AUCg
		Hackman (2012)	180	12–14 years	Parent–adolescent conflict discussion	3× (2 before, 1 after)	Saliva	No sex differences; pubertal status not assessed
		Minkley (2012)	93	17.86 ± 0.096 years	Examination challenge (reproduction of knowledge or transfer and problem-solving)	2×, 1 before, 1 after	Saliva	Not statistically significant, but higher increases in boys. More in reproduction of knowledge group, but also greater in transfer and problem-solving group
		Zijlmans (2013)	52	12.5 ± 1.21 years	Social Evaluative Stress Test	7×, 1 before, 6 after	Saliva	Higher reactivity in boys
	Physical stress	Allen (2009)	235	12.7 ± 2.9 years	Laboratory Pain Tasks	Saliva: 3×, 1 before, 2 after	Saliva/blood	No sex differences; pubertal status not associated with reactivity
						Blood: 2× (after)		
		Chiodo (2011)	16	Boys: 14 ± 0 years; girls: 13 ± 1 years	Taekwondo competition	5× (2 before, 3 after)	Saliva	Lower overall values in girls, but higher peak.
		Covelli (2012)	106	15.3 ± 1.1 years	Cold water hand immersion	2×, 1 before, 1 after	Saliva	No sex differences; pubertal status not assessed
		Frias (2000)	48	13–17 years	Acute alcohol intoxication	1× (after); controls as reference	Blood	More pronounced increase in girls
		Gecgelen (2012)	40	10.9–14.7 years	Rapid maxillary expansion	13×, 1 before, 3 after, and 9 during a period of treatment	Saliva	No sex differences; pubertal status not assessed
		Khilnani (1993)	98	2–20 years	Elective surgery	2×, 1 before, 1 after	Blood	No sex differences; pubertal status not assessed
		Kuhlman (2015)	121	12.8 ± 2.3 years	Socially evaluated cold pressor test	7× (2 before, 5 after)	Saliva	No sex differences; pubertal status not assessed
		Lopez-Duran (2015)	115	12.79 ± 2.26 years	Socially evaluated cold pressor test	8× (2 before, 6 after)	Saliva	No sex differences; pubertal status not assessed
		Stupnicki (1995)	29	Boys: 17.3 ± 0.8; girls: 16.4 ± 0.6 years	Exercise	2×, 1 before, 1 after	Blood	Boys decrease in cortisol; girls increase in cortisol after exercise
		Yfanti (2014)	97	89.73 ± 15 months	Dental treatment	5×, 1 before, 4 after	Saliva	No sex differences; pubertal status not assessed

### Diurnal rhythm

Twenty-nine studies (with the data of 8971 subjects) described diurnal rhythmicity and/or decline of cortisol throughout the day in children, of which 15 studies reported no significant sex differences [11–25]. Fourteen studies reported significant sex differences, of which 12 reported higher cortisol levels and/or a steeper decline over the day in girls. Both Adam et al. [26] (*n* = 230, age 17.04 ± 0.36 years) and Williams et al. [27] (*n* = 27, age 9.13 ± 1.41 years) reported a steeper diurnal cortisol curve in girls. Morin-Major et al. [28] (*n* = 88, age 14.5 ± 1.8 years) found a higher area under the curve as measured from the ground (AUCg) in girls. Martikainen et al. [29] (*n* = 252, age 8.1 ± 0.3 years) reported a higher cortisol level at awakening in girls, while there was no difference between sexes at nadir, suggesting a steeper cortisol decline over the day in girls compared to boys. This was also found by Rosmalen et al. [30] (*n* = 1768, age 11.08 ± 0.55 years), who found this to be already present prepubertally, while age and pubertal status were not associated with diurnal rhythm. Fransson et al. [31] (*n* = 157, age 14–16 years) found a higher cortisol level at awakening and a steeper diurnal decline in girls. Kelly et al. [32] (*n* = 2995, age 15.4 ± 0.3 years) found a greater decrease in cortisol concentration in girls as compared to boys between ±9 a.m. and 9:30 a.m. Ruttle et al. [33] (*n* = 346, age 11, 13, and 15 years) and Shirtcliff et al. [34] (*n* = 357, age 9, 11, 13, and 15 years) examined the same cohort. Ruttle et al. found a significantly steeper diurnal decline in girls aged 11 and 13 years. At age 15, gender differences in cortisol slope had disappeared, although girls had higher cortisol levels throughout the day. Shirtcliff et al. found similar differences, with higher cortisol and steeper slopes, as well as more curvature, in girls. Moreover, the circadian rhythm became flatter with advancing puberty, particularly among girls. Vaillancourt et al. [35] (*n* = 154, age 147 ± 9.1 months) examined morning and evening cortisol levels on Monday, Thursday, and Saturday. They only found a higher cortisol concentration in girls on Saturday morning. Moreover, after modeling the circadian pattern, they found that girls consistently had higher cortisol levels than boys throughout the day. Bae et al. [36] (*n* = 138, age 10.7 ± 1.7 years) found higher cortisol levels in girls at awakening and 30 min after awakening, as well as a higher total daily output. However, no sex differences were found with regard to diurnal slope or evening levels. Netherton et al. [37] (*n* = 129, age 12.8 ± 0.19 years) found higher morning cortisol levels in mid- to postpubertal girls compared to boys, but no sex differences were found in evening cortisol levels. In pre- to early-pubertal children, no sex differences were found in either morning or evening cortisol levels. Contrastingly, Kuhlman et al. [38] (*n* = 121, age 12.8 ± 2.3 years) reported no sex differences in cortisol levels at awakening or in linear decline, although girls showed more deceleration of the diurnal decline between dinner and bedtime than boys. Matchock et al. [39] (*n* = 120, age: boys 9, 11, or 13 years; girls 8, 10, or 12 years) found an earlier cortisol peak in the morning in girls and, at pubertal stage 2, a lower morning cortisol levels in girls. However, although a pubertal stage effect was found, there were no sex differences in the AUCg.

### CAR

Eighteen studies (with the data of 3549 subjects) described the CAR in children. Nine studies did not find differences between boys and girls [15–18, 21, 26, 27, 38, 40], although four of these [15–18] studied the CAR as part of the diurnal rhythm, and did not perform separate analyses for the CAR, with therefore limited data available on the CAR. Additionally, Michels et al. [18] (*n* = 385, age 5–10 years) and Vanaelst et al. [21] (*n* = 355, age 5–10 years) reported on the same cohort, and Osika et al. [15] (*n* = 84, age 9.9 ± 0.55 years) only took samples between 0 and 15 min after awakening. Nine studies found significant differences in CAR between sexes, of which eight found a higher CAR in girls. Martikainen et al. [29] (*n* = 252, age 8.1 ± 0.3 years) found a higher peak after awakening in girls, as well as a higher AUCg. However, the awakening response (i.e., the peak value after awakening minus the value immediately after awakening) as well as the AUC increase (AUCi) were not significantly different between the sexes. This was also found by Bouma et al. [41] (*n* = 644, age 16.1 ± 0.6 years) and Dietrich et al. [42] (*n* = 1604, age 11.1 ± 0.6 years), who reported on the same cohort (albeit at different ages) and found higher morning cortisol concentrations in girls, but a similar response to awakening in boys and girls, manifesting as a higher AUCg in girls but a similar AUCi between sexes. Additionally, Bae et al. [36] (*n* = 138, 10.7 ± 1.7 years) found higher cortisol levels in girls at awakening and 30 min after awakening, although they did not find sex differences in the AUCg. Fransson et al. [31] (*n* = 157, age 14–16 years) and Hatzinger et al. [43] (*n* = 102, age 4.9 ± 0.4 years) both found a higher CAR in girls, and Pruessner et al. [44] (*n* = 42, age 11.2 ± 2.0 years) showed a tendency towards larger increases in girls compared to boys. Morin-Major et al. [28] (*n* = 88, age 14.5 ± 1.8 years) found a correlation between the CAR and sex, with a higher CAR in girls. Contrastingly, Jones et al. [14] (*n* = 140, age 7–9 years) found the CAR to be absent in girls but present in boys.

### Protocolled social stress tests similar or equal to the TSST-C

Twenty-one studies (with the data of 3500 subjects) examined responses to standardized social stress tests. Eighteen used the TSST-C (validated in children aged ≥7 years), while three used other laboratory-based social stress tests that closely resemble the TSST-C [41, 45, 46]: the Groningen Social Stress Test (GSST) which consisted of a 6-min speech, a brief interlude, and a subtracting task; and a psychosocial stress test which consisted of a mental arithmetic task, a public speaking task, and a computer mathematics task. Eight studies, of which two studied the same cohort, did not find sex differences [36, 47–53], while 13 did find sex differences. Ji et al. [54] (*n* = 135, age: boys 9, 11, or 13 years; girls 8, 10, or 12 years) reported on the same cohort as Dockray et al. [48] and Peckins et al. [50], who did not find sex differences. However, Ji et al. found that at wave 3, where each wave is separated by 6 months, girls had a stronger cortisol response to the stressor, although they did not find sex differences with regard to cortisol recovery. Raikkonen et al. [55] (*n* = 292, age 8.1 ± 0.3 years) and Martikainen et al. [29] (*n* = 252, age 8.1 ± 0.3 years) reported on the same cohort and found a higher peak after stress and higher AUCs (both ground and increase) in girls, while no pre-test differences were found. De Veld [56] (*n* = 158, age 10.61 ± 0.52 years) found a stronger cortisol response in girls. Jones et al. [14] (*n* = 140, age 79 years) found an anticipatory rise in cortisol in both sexes, but only an additional increase after the TSST-C in girls. Evans et al. [45] (*n* = 707, age 13.8 ± 3.6 years) found that girls aged ≤12 years displayed higher cortisol reactivity to the psychological stress test, while sex differences were not present in subjects aged 13–20 years. A similar result was found by Hostinar et al. [57] (*n* = 81, age 9.97 ± 0.52 (children) and 16.05 ± 0.39 (adolescents) years), who found a stronger cortisol response in girls at ages 9 to 10, and no sex differences among the adolescents. Gunnar et al. [58] (*n* = 82, age 9, 11, 13, and 15 years) found a significantly higher AUCi in girls in response to the TSST-C at age 13, while no sex differences were found at ages 9, 11, and 15 years. Mrug et al. [59] (*n* = 84, age 13.4 ± 1.0 years) found a higher cortisol 55 min post-test as well as a greater AUCi in girls. On the other hand, Lu et al. [60] (*n* = 87, age 12.7 ± 0.3 years) found a significantly more negative logAUCi in girls, indicative of a smaller increase in cortisol in girls compared to boys after the TSST-C, and Trickett et al. [61] (*n* = 151 controls, age 11.11 ± 1.15 years) found a blunted cortisol response in girls compared to boys. Additionally, Bouma et al. [41] (*n* = 644, age 16.1 ± 0.6 years), who used the GSST, found lower cortisol responses in girls compared to boys, which was further specified in a study published by Bouma et al. in 2011 [46] (*n* = 553, age 16.07 ± 0.90 years), who found lower cortisol levels in girls on the first sample after completing the GSST.

### Pharmacological stress tests

Seven studies (with the data of 322 subjects) investigated cortisol responses to pharmacological ACTH or CRH. Five studies (3 with ACTH, 2 with CRH) did not find significant sex differences [62–66], and 2 studies found a smaller cortisol increase in girls. Stroud et al. [67] (*n* = 68, age 11.9 ± 1.9 years), who performed a CRH challenge with 1 μg/kg human CRH, found a smaller increase from baseline in girls compared to boys for all Tanner pubertal stages. Additionally, sex-specific pubertal changes were observed, with a baseline cortisol that increased in girls and decreased in boys with advancing puberty. Moreover, girls showed decreases in reactivity/recovery rates (in μg/dL/min), as well as increases in total cortisol response (AUCg) and time to peak cortisol levels with pubertal maturation. Boys, on the other hand, showed little change in reactivity/recovery rates and no changes across puberty for the other parameters. Dahl et al. [68] (*n* = 25, age 10.3 ± 1.6 years) also performed a 1 μg/kg human CRH challenge and found a smaller increase in cortisol concentration in girls compared to boys.

### Miscellaneous stress tests

Twenty-five studies (with the data of 3004 subjects) performed a wide range of other stress tests.

Three studies were performed in infants aged <1 year (with the data of 285 subjects) [69–71], of which two found a lower cortisol reactivity in girls: Davis and Emory [69] (*n* = 36, age 31.0 ± 8.1 h), who used the Neonatal Behavior Assessment Scale, and Eiden et al. [70] (*n* = 217, age 9 months), who used the Laboratory Temperament Assessment Battery.

Eight studies (with the data of 1472 subjects) were performed in children aged 1–7 years, of which six [72–77] found no sex differences. Hatzinger et al. [43] (*n* = 102, age 4.9 ± 0.4 years) used the MacArthur Story Stem Battery and found a higher reactivity in girls. Mills et al. [78] (*n* = 214, age 4.1 ± 0.2 years) used easy and difficult matching tasks with standardized failure and success. They found decreases in cortisol concentrations in both sexes up to 15 min post-stressor but only further decreases in boys.

Fourteen studies (with the data of 1247 subjects) assessed stress in children aged ≥7 years using miscellaneous protocols. Four studies performed psychological stress tests: one found no sex differences [79], while three found lower reactivity in girls. Zijlmans et al. [80] (*n* = 52, age 12.5 ± 1.2 years) used a computerized testing paradigm, the social evaluative stress test (SEST), containing elements of social evaluation, unpredictability, and uncontrollability. A lower reactivity was found in girls. Daughters et al. [81] (*n* = 132, age 16.1 ± 1.0 years) used the Behavioral Indicator of Resiliency to Distress (BIRD) and found no cortisol increase and slower cortisol decrease in girls, while there were no sex differences in AUCg. Minkley and Kirchner [82] (*n* = 93, age 17.9 ± 0.1 years) used two knowledge tests aimed at testing “reproduction of knowledge” or “transfer and problem-solving.” A lower reactivity was found in girls, although this was not statistically significant. Ten other studies assessed cortisol reactivity to physical stressors, of which seven did not find sex differences [38, 83–88], of which two reported on the same cohort [38, 88]. Chiodo et al. [89] (*n* = 16, age: boys 14 ± 0 years, girls 13 ± 1 years) used a Taekwondo competitions as stressor, and found lower overall values in girls, although they did exhibit a higher peak compared to boys. Stupnicki et al. [90] (*n* = 29, age 16–17 years) used physical exercise and found a cortisol increase after physical exercise in girls, compared to a decrease in boys. Frias et al. [91] (*n* = 48, age 13–17 years) assessed cortisol reactivity after acute alcohol intoxication (AAI). Both boys and girls showed an increase in cortisol concentrations after AAI compared to controls, but this increase was more pronounced in girls, although this was not statistically tested.

## Discussion

In this systematic review, we found that sex differences in HPA axis reactivity are suggested to be present in childhood. In general, with regard to diurnal rhythm, the CAR and social stress tests, around 50% of the studies, notably the larger ones, found sex differences, of which approximately 80% found a more variable diurnal rhythm, a higher CAR, and/or a stronger cortisol response to social stress tests in girls, suggestive of a more variable HPA axis. We found no evidence for a sex difference in cortisol response after a pharmacological challenge, with only two out of 7 studies reporting a higher cortisol response in boys. Findings from studies addressing sex differences in cortisol response after miscellaneous (social or physical) stress tests were inconsistent, due to different types of stressors applied.

In total, 12 out of 29 studies found a more variable diurnal rhythm in girls, while 2 found this in boys and 15 did not find sex differences. A higher CAR in girls was found in 8 out of 18 studies, although 1 study found a higher CAR in boys and 9 studies found no sex differences. Girls had a stronger cortisol response to social stress tests in 9 out of 21 studies, whereas boys had a stronger response in 4 studies and no sex differences were found in 8 studies. Therefore, although results are suggestive of a more responsive HPA axis in girls during childhood, these results must be interpreted with caution as the evidence is not unequivocal. However, the sample sizes of the studies that found sex differences were on average larger, while the studies that did not find sex differences more often had a sample size <100.

Our results differed considerably with findings from studies in adults. Notably, psychological stress studies in adults either found no gender difference or a more pronounced cortisol response in men [4]. This difference might be explained by gonadal hormones, more specifically estrogens. In childhood, as we have shown in this review, cortisol reactivity appears to be more pronounced in females. However, other research has shown that in adults, females were found to exhibit attenuated cortisol responses to stress, and males displayed a higher cortisol reactivity [4]. Consequently, it could be hypothesized that postmenopausal women once again show a stronger cortisol response to stress compared to men of the same age. Otte et al. [5], who performed a meta-analysis to evaluate and quantify age-related changes in cortisol response, found a threefold higher increase in cortisol reactivity with aging in women compared to men. However, studies examining cortisol reactivity in elderly subjects are inconclusive with regard to gender differences [92–95].

According to the Developmental Origins of Health and Disease (DOHaD) hypothesis, disease susceptibility arises early in development [1] and might be mediated by HPA axis (re)activity. Dysfunctional (hypo- or hyperreactive) HPA axis responses have previously been associated with cardiovascular disease risk [96]. In addition, more subtle differences in early HPA axis settings can also contribute to sex-specific disease risks throughout life [10, 97].

Sex differences in HPA axis reactivity might be due to interactions between the HPA and HPG axes, and several mechanisms have been proposed. Estradiol has been shown to enhance, while testosterone inhibited CRH gene transcription in the hypothalamus [9]. In addition, estradiol has been found to sensitize the pituitary, thereby increasing the ACTH response, while progesterone seemed to oppose this effect [9]. Moreover, estrogen receptors (ERs) are widely expressed throughout the brain, especially in the limbic system. Although not unequivocal, the distribution of the ER subtypes α and β, which have opposing actions on the HPA axis [98], is probably sex-dependent [99]. In rats, gender differences in the expression of ERs were already present early in life [100]. It is possible that sex differences in the balance and distribution of ERα and ERβ in the brain are already present before puberty as a result of priming [1] or genetics, which subsequently change after the onset of puberty. In addition, the sensitivity of the adrenal cortex to ACTH is suggested to be increased in young women [9], while estrogens were found to increase the production of corticosteroid-binding globulin (CBG) [101], decrease glucocorticoid receptor (GR) expression and activation [7], and lower hepatic clearance of cortisol by inhibition of A-ring reduction [102]. In contrast, testosterone was found to inhibit the release of ACTH, while progesterone possibly acts as a glucocorticoid antagonist. [9, 47, 103] However, estrogens seem to have different effects in (postmenopausal) women and men [104–106], and ACTH responses to a TSST after 2 weeks of DHEA or placebo treatment was found to be equal for women treated with DHEA to those of men but increased compared to women taking placebos [4]. These HPA/HPG axes interactions might explain why the sex differences in HPA axis reactivity that we found in children are not corroborated by studies in adults. Moreover, some of the included studies in this review took pubertal status into account [13, 24, 30, 33, 34, 36, 37, 39, 45, 48, 50–52, 64, 65, 67, 83, 94]. Although different (sex-specific) effects of pubertal status on cortisol reactivity were found, HPA/HPG axes interactions might nevertheless play a role in the possible sex-specific changes in HPA axis reactivity throughout puberty. Furthermore, levels of estradiol and progesterone are highly variable in post-menarcheal girls and, therefore, HPA-/HPG-axes interactions might even fluctuate across the menstrual cycle.

The different natures and effects of the applied stressors are something to take into account when assessing HPA axis reactivity. Different types of stressors activate different levels along the HPA axis: standard ACTH tests stimulate the adrenals directly, while psychological tests are indirect stimuli of the adrenal cortex through activation of the limbic system. Moreover, the diurnal rhythm and CAR are largely controlled by the suprachiasmatic nucleus, which influences CRH release from the paraventricular nucleus [107]. Additionally, males seem to have a “fight or flight” reaction, with a stronger response when confronted with an achievement challenge (in which you can succeed or fail at a task), while women show a “tend or befriend” response and therefore seem to be more sensitive to stress tests that incorporate social rejection or peer pressure [81, 108, 109]. This might be due to the previously mentioned HPA/HPG axes interactions, as well as possible sexually dimorphic site-specific GR and MR expression patterns in the brain [2, 110]. Consequently, when designing a study, it is important to realize what type of stress and which level of the HPA axis is aimed to be tested. Subsequently, the effect of gender on that specific type of stressor should be taken into account. We recommend using standardized protocols, since gender-specific effects on HPA axis reactivity have been best described with regard to standardized stress protocols.

Additionally, comparing the results of the studies included in our systematic review was hampered by the fact that data were collected and presented in numerous ways. For the same reason, it was impossible to perform a meta-analysis. Moreover, only limited information was often provided, and it is therefore possible that (subtle) sex differences were not found. This was the case for all categories of HPA axis reactivity discussed in this review. In order to draw more precise conclusions concerning gender differences in HPA axis reactivity in childhood, we wish to argue using standardized protocols, as well as a standardized presentation of results for future studies on HPA axis reactivity. Seeman and Robbins [111] have defined stress resiliency as “the overall pattern of HPA response to challenge,” which includes the rate of initial response, the magnitude of the response, and the rate of recovery of the HPA axis. In order to be able to draw conclusions on all of these aspects and to enable unbiased, quantitative comparisons, reporting data on HPA axis reactivity should take all of these aspects into account. This can be done by both reporting absolute cortisol values (e.g., minimum and maximum cortisol levels) as well as derived variables (e.g., time to peak/recovery, delta cortisol, ascending/descending slopes and areas under the curve), preferably analyzing sex differences for all these parameters. This will allow a full appreciation and overview of the course followed by cortisol from pre- to post-stressor.

Our review has several strengths and limitations. Our strengths lie in the systematic and extensive search performed, which has resulted in the inclusion of 81 studies. Our review is limited not only by the previously mentioned concerns but also by the broad range in ages as well as the lack of (reliable) establishment of pubertal stage in the majority of the included articles. Although several studies mention an effect of age or pubertal status on cortisol reactivity [13, 22, 24, 30, 33, 34, 36, 37, 39, 45, 48, 50–52, 54, 57, 58, 64, 65, 67, 83, 94], findings are conflicting between the articles. Moreover, we ourselves were unable to draw any conclusions with regard to age or pubertal status, due to the heterogeneous ways of analyzing these effects as well as limited power within studies. Moreover, pubertal status was often assessed through self-report, which has poor reliability [112]. However, it is possible that the effect of age and/or pubertal status can partly explain our unequivocal conclusions regarding sex differences, as was previously suggested by Jessop and Turner-Cobb [10]. Aside from standardizing the collection and presentation of data, we therefore urge to also always take age and pubertal status into account. This is in line with a recent study in adults, which showed that adjusting for sex hormones significantly alters sex-specific cortisol profiles [113].

## Conclusions

In conclusion, we found that gender differences in HPA axis reactivity appear to be present in childhood, suggestive of priming of the HPA axis during early development, although the evidence is not unequivocal. Overall, girls appear to have a more variable diurnal rhythm, a higher CAR, and a higher cortisol response to social stress tests. These differences are not in line with studies in adults, which might be due to changes in gonadal hormones during puberty impacting on HPA axis reactivity. We found various gender differences depending on the type of stressor applied, which stresses the importance of taking the nature of the stressor into account when designing a new study. Moreover, standardization of protocols and reports of results is warranted.

## Additional files

Additional file 1: Search strategy. (DOCX 18 kb)

Additional file 2: Extracted data of the studies included in the systematic review. (DOCX 29772 kb)

## Abbreviations

ACTH: Adrenocorticotropic hormone
AUCg: Area under the curve ground
AUCi: Area under the curve increase
CAR: Cortisol awakening response
CBG: Corticosteroid-binding globulin
CRH: Corticotropin-releasing hormone
ER: Estrogen receptor
HPA axis: Hypothalamus–pituitary–adrenal axis
HPG axis: hypothalamus–pituitary–gonadal axis
PRISMA: Preferred Reporting Items for Systematic Reviews and Meta-Analysis
TSST-C: Trier Social Stress Test for Children

## References

[1] Hanson MA, Gluckman PD (2014). Early developmental conditioning of later health and disease: physiology or pathophysiology?. Physiol Rev.

[2] De Kloet ER, Vreugdenhil E, Oitzl MS, Joels M (1998). Brain corticosteroid receptor balance in health and disease. Endocr Rev.

[3] Slotkin TA, Seidler FJ, Wood CR, Lau C (2008). Development of glucocorticoid receptor regulation in the rat forebrain: implications for adverse effects of glucocorticoids in preterm infants. Brain Res Bull.

[4] Kudielka BM, Kirschbaum C (2005). Sex differences in HPA axis responses to stress: a review. Biol Psychol.

[5] Otte C, Hart S, Neylan TC, Marmar CR, Yaffe K, Mohr DC (2005). A meta-analysis of cortisol response to challenge in human aging: importance of gender. Psychoneuroendocrinology.

[6] Lerner DJ, Kannel WB (1986). Patterns of coronary heart disease morbidity and mortality in the sexes: a 26-year follow-up of the Framingham population. Am Heart J.

[7] Bourke CH, Harrell CS, Neigh GN (2012). Stress-induced sex differences: adaptations mediated by the glucocorticoid receptor. Horm Behav.

[8] van der Voorn B, Hollanders JJ, Ket JC, Rotteveel J, Finken MJ (2017). Gender-specific differences in hypothalamus-pituitary-adrenal axis activity during childhood: a systematic review and meta-analysis. Biol Sex Differ.

[9] Panagiotakopoulos L, Neigh GN (2014). Development of the HPA axis: where and when do sex differences manifest?. Front Neuroendocrinol.

[10] Jessop DS, Turner-Cobb JM (2008). Measurement and meaning of salivary cortisol: a focus on health and disease in children. Stress.

[11] Haen EH F, Cornelissen G (1984). Cortisol marker rhythmometry in pediatrics and clinical pharmacology. Annual Review of Chronopharmacology.

[12] Garcia L, Hermida RC, Ayala DE, Lodeiro C, Iglesias T (1990). Circadian characteristics of plasma cortisol in children with standard and short stature. Chronobiol Int.

[13] Knutsson U, Dahlgren J, Marcus C, Rosberg S, Bronnegard M, Stierna P, Albertsson-Wikland K (1997). Circadian cortisol rhythms in healthy boys and girls: relationship with age, growth, body composition, and pubertal development. J Clin Endocrinol Metab.

[14] Jones A, Godfrey KM, Wood P, Osmond C, Goulden P, Phillips DI (2006). Fetal growth and the adrenocortical response to psychological stress. J Clin Endocrinol Metab.

[15] Osika W, Friberg P, Wahrborg P (2007). A new short self-rating questionnaire to assess stress in children. Int J Behav Med.

[16] Susman EJ, Dockray S, Schiefelbein VL, Herwehe S, Heaton JA, Dorn LD (2007). Morningness/eveningness, morning-to-afternoon cortisol ratio, and antisocial behavior problems during puberty. Dev Psychol.

[17] Tzortzi C, Proff P, Redlich M, Aframian DJ, Palmon A, Golan I, Muessig D, Wichelhaus A, Baumert U (2009). Cortisol daily rhythm in saliva of healthy school children. Int Dent J.

[18] Michels N, Sioen I, Huybrechts I, Bammann K, Vanaelst B, De Vriendt T, Iacoviello L, Konstabel K, Ahrens W, De Henauw S (2012). Negative life events, emotions and psychological difficulties as determinants of salivary cortisol in Belgian primary school children. Psychoneuroendocrinology.

[19] Kjolhede EA, Gustafsson PE, Gustafsson PA, Nelson N (2014). Overweight and obese children have lower cortisol levels than normal weight children. Acta Paediatr.

[20] Lumeng JC, Miller A, Peterson KE, Kaciroti N, Sturza J, Rosenblum K, Vazquez DM (2014). Diurnal cortisol pattern, eating behaviors and overweight in low-income preschool-aged children. Appetite.

[21] Vanaelst B, Michels N, Clays E, Herrmann D, Huybrechts I, Sioen I, Vyncke K, De Henauw S (2014). The association between childhood stress and body composition, and the role of stress-related lifestyle factors—cross-sectional findings from the baseline ChiBSD survey. Int J Behav Med.

[22] Barbosa TS, Castelo PM, Leme MS, Gaviao MB (2012). Associations between oral health-related quality of life and emotional statuses in children and preadolescents. Oral Dis.

[23] Bartels M, De Geus EJ, Kirschbaum C, Sluyter F, Boomsma DI (2003). Heritability of daytime cortisol levels in children. Behav Genet.

[24] Carrion VG, Weems CF, Ray RD, Glaser B, Hessl D, Reiss AL (2002). Diurnal salivary cortisol in pediatric posttraumatic stress disorder. Biol Psychiatry.

[25] Doom JR, Cicchetti D, Rogosch FA, Dackis MN (2013). Child maltreatment and gender interactions as predictors of differential neuroendocrine profiles. Psychoneuroendocrinology.

[26] Adam EK, Doane LD, Zinbarg RE, Mineka S, Craske MG, Griffith JW (2010). Prospective prediction of major depressive disorder from cortisol awakening responses in adolescence. Psychoneuroendocrinology.

[27] Williams SR, Cash E, Daup M, Geronimi EM, Sephton SE, Woodruff-Borden J (2013). Exploring patterns in cortisol synchrony among anxious and nonanxious mother and child dyads: a preliminary study. Biol Psychol.

[28] Morin-Major JK, Marin MF, Durand N, Wan N, Juster RP, Lupien SJ (2016). Facebook behaviors associated with diurnal cortisol in adolescents: is befriending stressful?. Psychoneuroendocrinology.

[29] Martikainen S, Pesonen AK, Lahti J, Heinonen K, Feldt K, Pyhala R, Tammelin T, Kajantie E, Eriksson JG, Strandberg TE, Raikkonen K (2013). Higher levels of physical activity are associated with lower hypothalamic-pituitary-adrenocortical axis reactivity to psychosocial stress in children. J Clin Endocrinol Metab.

[30] Rosmalen JG, Oldehinkel AJ, Ormel J, De Winter AF, Buitelaar JK, Verhulst FC (2005). Determinants of salivary cortisol levels in 10–12 year old children; a population-based study of individual differences. Psychoneuroendocrinology.

[31] Fransson E, Folkesson L, Bergstrom M, Ostberg V, Lindfors P (2014). Exploring salivary cortisol and recurrent pain in mid-adolescents living in two homes. BMC Psychol.

[32] Kelly SJ, Young R, Sweeting H, Fischer JE, West P (2008). Levels and confounders of morning cortisol collected from adolescents in a naturalistic (school) setting. Psychoneuroendocrinology.

[33] Ruttle PL, Javaras KN, Klein MH, Armstrong JM, Burk LR, Essex MJ (2013). Concurrent and longitudinal associations between diurnal cortisol and body mass index across adolescence. J Adolesc Health.

[34] Shirtcliff EA, Allison AL, Armstrong JM, Slattery MJ, Kalin NH, Essex MJ (2012). Longitudinal stability and developmental properties of salivary cortisol levels and circadian rhythms from childhood to adolescence. Dev Psychobiol.

[35] Vaillancourt T, Duku E, Decatanzaro D, Macmillan H, Muir C, Schmidt LA (2008). Variation in hypothalamic-pituitary-adrenal axis activity among bullied and non-bullied children. Aggress Behav.

[36] Bae YJ, Stadelmann S, Klein AM, Jaeger S, Hiemisch A, Kiess W, Ceglarek U, Gaudl A, Schaab M, Von Klitzing K (2015). The hyporeactivity of salivary cortisol at stress test (TSST-C) in children with internalizing or externalizing disorders is contrastively associated with alpha-amylase. J Psychiatr Res.

[37] Netherton C, Goodyer I, Tamplin A, Herbert J (2004). Salivary cortisol and dehydroepiandrosterone in relation to puberty and gender. Psychoneuroendocrinology.

[38] Kuhlman KR, Geiss EG, Vargas I, Lopez-Duran NL (2015). Differential associations between childhood trauma subtypes and adolescent HPA-axis functioning. Psychoneuroendocrinology.

[39] Matchock RL, Dorn LD, Susman EJ (2007). Diurnal and seasonal cortisol, testosterone, and DHEA rhythms in boys and girls during puberty. Chronobiol Int.

[40] Bright MA, Frick JE, Out D, Granger DA (2014). Individual differences in the cortisol and salivary alpha-amylase awakening responses in early childhood: relations to age, sex, and sleep. Dev Psychobiol.

[41] Bouma EM, Riese H, Ormel J, Verhulst FC, Oldehinkel AJ (2009). Adolescents’ cortisol responses to awakening and social stress; effects of gender, menstrual phase and oral contraceptives. The TRAILS study. Psychoneuroendocrinology.

[42] Dietrich A, Ormel J, Buitelaar JK, Verhulst FC, Hoekstra PJ, Hartman CA (2013). Cortisol in the morning and dimensions of anxiety, depression, and aggression in children from a general population and clinic-referred cohort: an integrated analysis. The TRAILS study. Psychoneuroendocrinology.

[43] Hatzinger M, Brand S, Perren S, Von Wyl A, Von Klitzing K, Holsboer-Trachsler E (2007). Hypothalamic-pituitary-adrenocortical (HPA) activity in kindergarten children: importance of gender and associations with behavioral/emotional difficulties. J Psychiatr Res.

[44] Pruessner JC, Wolf OT, Hellhammer DH, Buske-Kirschbaum A, Von Auer K, Jobst S, Kaspers F, Kirschbaum C (1997). Free cortisol levels after awakening: a reliable biological marker for the assessment of adrenocortical activity. Life Sci.

[45] Evans BE, Greaves-Lord K, Euser AS, Tulen JH, Franken IH, Huizink AC (2013). Determinants of physiological and perceived physiological stress reactivity in children and adolescents. PLoS ONE.

[46] Bouma EM, Riese H, Nolte IM, Oosterom E, Verhulst FC, Ormel J, Oldehinkel AJ (2011). No associations between single nucleotide polymorphisms in corticoid receptor genes and heart rate and cortisol responses to a standardized social stress test in adolescents: the TRAILS study. Behav Genet.

[47] Kudielka BM, Buske-Kirschbaum A, Hellhammer DH, Kirschbaum C (2004). HPA axis responses to laboratory psychosocial stress in healthy elderly adults, younger adults, and children: impact of age and gender. Psychoneuroendocrinology.

[48] Dockray S, Susman EJ, Dorn LD (2009). Depression, cortisol reactivity, and obesity in childhood and adolescence. J Adolesc Health.

[49] Strahler J, Mueller A, Rosenloecher F, Kirschbaum C, Rohleder N (2010). Salivary alpha-amylase stress reactivity across different age groups. Psychophysiology.

[50] Peckins MK, Dockray S, Eckenrode JL, Heaton J, Susman EJ (2012). The longitudinal impact of exposure to violence on cortisol reactivity in adolescents. J Adolesc Health.

[51] Portnoy J, Raine A, Glenn AL, Chen FR, Choy O, Granger DA (2015). Digit ratio (2D:4D) moderates the relationship between cortisol reactivity and self-reported externalizing behavior in young adolescent males. Biol Psychol.

[52] Hostinar CE, McQuillan MT, Mirous HJ, Grant KE, Adam EK (2014). Cortisol responses to a group public speaking task for adolescents: variations by age, gender, and race. Psychoneuroendocrinology.

[53] Martin A, Hellhammer J, Hero T, Max H, Schult J, Terstegen L (2011). Effective prevention of stress-induced sweating and axillary malodour formation in teenagers. Int J Cosmet Sci.

[54] Ji J, Negriff S, Kim H, Susman EJ (2016). A study of cortisol reactivity and recovery among young adolescents: heterogeneity and longitudinal stability and change. Dev Psychobiol.

[55] Raikkonen K, Matthews KA, Pesonen AK, Pyhala R, Paavonen EJ, Feldt K, Jones A, Phillips DI, Seckl JR, Heinonen K (2010). Poor sleep and altered hypothalamic-pituitary-adrenocortical and sympatho-adrenal-medullary system activity in children. J Clin Endocrinol Metab.

[56] De Veld DM, Riksen-Walraven JM, De Weerth C (2012). The relation between emotion regulation strategies and physiological stress responses in middle childhood. Psychoneuroendocrinology.

[57] Hostinar CE, Johnson AE, Gunnar MR (2015). Parent support is less effective in buffering cortisol stress reactivity for adolescents compared to children. Dev Sci.

[58] Gunnar MR, Wewerka S, Frenn K, Long JD, Griggs C (2009). Developmental changes in hypothalamus-pituitary-adrenal activity over the transition to adolescence: normative changes and associations with puberty. Dev Psychopathol.

[59] Mrug S, Tyson A, Turan B, Granger DA (2016). Sleep problems predict cortisol reactivity to stress in urban adolescents. Physiol Behav.

[60] Lu Q, Tao F, Hou F, Zhang Z, Sun Y, Xu Y, Xu S, Zhao Y (2014). Cortisol reactivity, delay discounting and percent body fat in Chinese urban young adolescents. Appetite.

[61] Trickett PK, Gordis E, Peckins MK, Susman EJ (2014). Stress reactivity in maltreated and comparison male and female young adolescents. Child Maltreat.

[62] Dorn LD, Burgess ES, Susman EJ, Von Eye A, DeBellis MD, Gold PW, Chrousos GP (1996). Response to oCRH in depressed and nondepressed adolescents: does gender make a difference?. J Am Acad Child Adolesc Psychiatry.

[63] Forest MG (1978). Age-related response of plasma testosterone, delta 4-androstenedione, and cortisol to adrenocorticotropin in infants, children, and adults. J Clin Endocrinol Metab.

[64] Lashansky G, Saenger P, Fishman K, Gautier T, Mayes D, Berg G, Di Martino-Nardi J, Reiter E (1991). Normative data for adrenal steroidogenesis in a healthy pediatric population: age- and sex-related changes after adrenocorticotropin stimulation. J Clin Endocrinol Metab.

[65] Ross JL, Schulte HM, Gallucci WT, Cutler GB, Loriaux DL, Chrousos GP (1986). Ovine corticotropin-releasing hormone stimulation test in normal children. J Clin Endocrinol Metab.

[66] Tsvetkova V (1977). Adrenocortical function after stimulation with synthetic ACTH. Curr Med Res Opin.

[67] Stroud LR, Papandonatos GD, Williamson DE, Dahl RE (2011). Sex differences in cortisol response to corticotropin releasing hormone challenge over puberty: Pittsburgh Pediatric Neurobehavioral Studies. Psychoneuroendocrinology.

[68] Dahl RE, Siegel SF, Williamson DE, Lee PA, Perel J, Birmaher B, Ryan ND (1992). Corticotropin releasing hormone stimulation test and nocturnal cortisol levels in normal children. Pediatr Res.

[69] Davis M, Emory E (1995). Sex differences in neonatal stress reactivity. Child Dev.

[70] Eiden RD, Molnar DS, Granger DA, Colder CR, Schuetze P, Huestis MA (2015). Prenatal tobacco exposure and infant stress reactivity: role of child sex and maternal behavior. Dev Psychobiol.

[71] Grunau RE, Tu MT, Whitfield MF, Oberlander TF, Weinberg J, Yu W, Thiessen P, Gosse G, Scheifele D (2010). Cortisol, behavior, and heart rate reactivity to immunization pain at 4 months corrected age in infants born very preterm. Clin J Pain.

[72] Gunnar MR, Kryzer E, Van Ryzin MJ, Phillips DA (2010). The rise in cortisol in family day care: associations with aspects of care quality, child behavior, and child sex. Child Dev.

[73] Plusquellec P, Ouellet-Morin I, Feng B, Perusse D, Tremblay RE, Lupien SJ, Boivin M (2011). Salivary cortisol levels are associated with resource control in a competitive situation in 19 month-old boys. Horm Behav.

[74] Spinrad TL, Eisenberg N, Granger DA, Eggum ND, Sallquist J, Haugen RG, Kupfer A, Hofer C (2009). Individual differences in preschoolers’ salivary cortisol and alpha-amylase reactivity: relations to temperament and maladjustment. Horm Behav.

[75] De Weerth C, Zijlmans MA, Mack S, Beijers R (2013). Cortisol reactions to a social evaluative paradigm in 5- and 6-year-old children. Stress.

[76] Kryski KR, Smith HJ, Sheikh HI, Singh SM, Hayden EP (2013). HPA axis reactivity in early childhood: associations with symptoms and moderation by sex. Psychoneuroendocrinology.

[77] Yong Ping E, Laplante DP, Elgbeili G, Hillerer KM, Brunet A, O’Hara MW, King S (2015). Prenatal maternal stress predicts stress reactivity at 2(1/2) years of age: the Iowa Flood Study. Psychoneuroendocrinology.

[78] Mills RS, Imm GP, Walling BR, Weiler HA (2008). Cortisol reactivity and regulation associated with shame responding in early childhood. Dev Psychol.

[79] Hackman DA, Betancourt LM, Brodsky NL, Hurt H, Farah MJ (2012). Neighborhood disadvantage and adolescent stress reactivity. Front Hum Neurosci.

[80] Zijlmans MA, Beijers R, Mack S, Pruessner JC, De Weerth C (2013). Cortisol responses to social evaluation in 10- to 15-year-old boys and girls. Stress.

[81] Daughters SB, Gorka SM, Matusiewicz A, Anderson K (2013). Gender specific effect of psychological stress and cortisol reactivity on adolescent risk taking. J Abnorm Child Psychol.

[82] Minkley N, Kirchner WH (2012). Influence of test tasks with different cognitive demands on salivary cortisol concentrations in school students. Int J Psychophysiol.

[83] Allen LB, Lu Q, Tsao JC, Worthman CM, Zeltzer LK (2009). Sex differences in the association between cortisol concentrations and laboratory pain responses in healthy children. Gend Med.

[84] Covelli MM, Wood CE, Yarandi HN (2012). Biologic measures as epidemiological indicators of risk for the development of hypertension in an African American adolescent population. J Cardiovasc Nurs.

[85] Gecgelen M, Aksoy A, Kirdemir P, Doguc DK, Cesur G, Koskan O, Ozorak O (2012). Evaluation of stress and pain during rapid maxillary expansion treatments. J Oral Rehabil.

[86] Khilnani P, Munoz R, Salem M, Gelb C, Todres ID, Chernow B (1993). Hormonal responses to surgical stress in children. J Pediatr Surg.

[87] Yfanti K, Kitraki E, Emmanouil D, Pandis N, Papagiannoulis L (2014). Psychometric and biohormonal indices of dental anxiety in children. A prospective cohort study. Stress.

[88] Lopez-Duran NL, McGinnis E, Kuhlman K, Geiss E, Vargas I, Mayer S (2015). HPA-axis stress reactivity in youth depression: evidence of impaired regulatory processes in depressed boys. Stress.

[89] Chiodo S, Tessitore A, Cortis C, Cibelli G, Lupo C, Ammendolia A, De Rosas M, Capranica L (2011). Stress-related hormonal and psychological changes to official youth Taekwondo competitions. Scand J Med Sci Sports.

[90] Stupnicki R, Obminski Z, Klusiewicz A, Viru A (1995). Pre-exercise serum cortisol concentration and responses to laboratory exercise. Eur J Appl Physiol Occup Physiol.

[91] Frias J, Rodriguez R, Torres JM, Ruiz E, Ortega E (2000). Effects of acute alcohol intoxication on pituitary-gonadal axis hormones, pituitary-adrenal axis hormones, beta-endorphin and prolactin in human adolescents of both sexes. Life Sci.

[92] Kudielka BM, Hellhammer J, Hellhammer DH, Wolf OT, Pirke KM, Varadi E, Pilz J, Kirschbaum C (1998). Sex differences in endocrine and psychological responses to psychosocial stress in healthy elderly subjects and the impact of a 2-week dehydroepiandrosterone treatment. J Clin Endocrinol Metab.

[93] Lekkakou L, Tzanela M, Lymberi M, Consoulas C, Tsagarakis S, Koutsilieris M (2013). Effects of gender and age on hypothalamic-pituitary-adrenal reactivity after pharmacological challenge with low-dose 1-μg ACTH test: a prospective study in healthy adults. Clin Endocrinol (Oxf).

[94] Seeman TE, Singer B, Charpentier P (1995). Gender differences in patterns of HPA axis response to challenge: Macarthur studies of successful aging. Psychoneuroendocrinology.

[95] Traustadottir T, Bosch PR, Matt KS (2003). Gender differences in cardiovascular and hypothalamic-pituitary-adrenal axis responses to psychological stress in healthy older adult men and women. Stress.

[96] Rosmond R, Bjorntorp P (2000). The hypothalamic-pituitary-adrenal axis activity as a predictor of cardiovascular disease, type 2 diabetes and stroke. J Intern Med.

[97] Kajantie E, Hovi P (2014). Is very preterm birth a risk factor for adult cardiometabolic disease?. Semin Fetal Neonatal Med.

[98] Lund TD, Rovis T, Chung WC, Handa RJ (2005). Novel actions of estrogen receptor-beta on anxiety-related behaviors. Endocrinology.

[99] Gillies GE, McArthur S (2010). Estrogen actions in the brain and the basis for differential action in men and women: a case for sex-specific medicines. Pharmacol Rev.

[100] Yokosuka M, Okamura H, Hayashi S (1997). Postnatal development and sex difference in neurons containing estrogen receptor-alpha immunoreactivity in the preoptic brain, the diencephalon, and the amygdala in the rat. J Comp Neurol.

[101] Moore DE, Kawagoe S, Davajan V, Mishell DR, Nakamura RM (1978). An in vivo system in man for quantitation of estrogenicity. I. Physiologic changes in binding capacity of serum corticosteroid-binding globulin. Am J Obstet Gynecol.

[102] Finken MJ, Andrews RC, Andrew R, Walker BR (1999). Cortisol metabolism in healthy young adults: sexual dimorphism in activities of A-ring reductases, but not 11β-hydroxysteroid dehydrogenases. J Clin Endocrinol Metab.

[103] Kajantie E, Phillips DI (2006). The effects of sex and hormonal status on the physiological response to acute psychosocial stress. Psychoneuroendocrinology.

[104] Kirschbaum C, Schommer N, Federenko I, Gaab J, Neumann O, Oellers M, Rohleder N, Untiedt A, Hanker J, Pirke KM, Hellhammer DH (1996). Short-term estradiol treatment enhances pituitary-adrenal axis and sympathetic responses to psychosocial stress in healthy young men. J Clin Endocrinol Metab.

[105] Komesaroff PA, Esler MD, Sudhir K (1999). Estrogen supplementation attenuates glucocorticoid and catecholamine responses to mental stress in perimenopausal women. J Clin Endocrinol Metab.

[106] Lindheim SR, Legro RS, Bernstein L, Stanczyk FZ, Vijod MA, Presser SC, Lobo RA (1992). Behavioral stress responses in premenopausal and postmenopausal women and the effects of estrogen. Am J Obstet Gynecol.

[107] Clow A, Hucklebridge F, Stalder T, Evans P, Thorn L (2010). The cortisol awakening response: more than a measure of HPA axis function. Neurosci Biobehav Rev.

[108] Stroud LR, Salovey P, Epel ES (2002). Sex differences in stress responses: social rejection versus achievement stress. Biol Psychiatry.

[109] Taylor SE, Klein LC, Lewis BP, Gruenewald TL, Gurung RA, Updegraff JA (2000). Biobehavioral responses to stress in females: tend-and-befriend, not fight-or-flight. Psychol Rev.

[110] Kang HJ, Kawasawa YI, Cheng F, Zhu Y, Xu X, Li M, Sousa AM, Pletikos M, Meyer KA, Sedmak G (2011). Spatio-temporal transcriptome of the human brain. Nature.

[111] Seeman TE, Robbins RJ (1994). Aging and hypothalamic-pituitary-adrenal response to challenge in humans. Endocr Rev.

[112] Rasmussen AR, Wohlfahrt-Veje C, Tefre de Renzy-Martin K, Hagen CP, Tinggaard J, Mouritsen A, Mieritz MG, Main KM (2015). Validity of self-assessment of pubertal maturation. Pediatrics.

[113] Juster RP, Raymond C, Desrochers AB, Bourdon O, Durand N, Wan N, Pruessner JC, Lupien SJ (2016). Sex hormones adjust “sex-specific” reactive and diurnal cortisol profiles. Psychoneuroendocrinology.

