# Supplementary material for: Is HPA axis reactivity in childhood gender-specific? A systematic review
Source: Biol Sex Differ. 2017 Jul 11;8:23. doi: 10.1186/s13293-017-0144-8 (PMC5504848; doi:10.1186/s13293-017-0144-8)
Supplement: Supplementary file 2 — Extracted data of the studies included in the systematic review. (DOCX 29772 kb) [file 13293_2017_144_MOESM2_ESM.docx]

**Supplementary file 2. Extracted data of the studies included in the systematic review**

Table of contents

[Adam 2010 [1] 4](#_Toc482883497)

[Allen 2009 [2] 6](#_Toc482883498)

[Bae 2015 [3] 7](#_Toc482883499)

[Barbosa 2012 [4] 10](#_Toc482883500)

[Bartels 2003 [5] 11](#_Toc482883501)

[Bouma 2009 [6] 12](#_Toc482883502)

[Bouma 2011 [7] 14](#_Toc482883503)

[Bright 2014 [8] 15](#_Toc482883504)

[Carrion 2002 [9] 16](#_Toc482883505)

[Chiodo 2011 [10] 17](#_Toc482883506)

[Covelli 2012 [11] 18](#_Toc482883507)

[Dahl 1992 [12] 19](#_Toc482883508)

[Daughters 2013 [13] 20](#_Toc482883509)

[Davis 1995 [14] 22](#_Toc482883510)

[De Veld 2012 [15] 24](#_Toc482883511)

[De Weerth 2013 [16] 25](#_Toc482883512)

[Dietrich 2013 [17] 26](#_Toc482883513)

[Dockray 2009 [18] 27](#_Toc482883514)

[Doom 2013 [19] 28](#_Toc482883515)

[Dorn 1996 [20] 30](#_Toc482883516)

[Eiden 2015 [21] 32](#_Toc482883517)

[Evans 2013 [22] 34](#_Toc482883518)

[Forest 1978 [23] 35](#_Toc482883519)

[Fransson 2014 [24] 36](#_Toc482883520)

[Frias 2000 [25] 37](#_Toc482883521)

[Garcia 1990 [26] 38](#_Toc482883522)

[Gecgelen 2012 [27] 39](#_Toc482883523)

[Grunau 2010 [28] 40](#_Toc482883524)

[Gunnar 2009 [29] 41](#_Toc482883525)

[Gunnar 2010 [30] 43](#_Toc482883526)

[Hackman 2012 [31] 45](#_Toc482883527)

[Haen 1984 [32] 46](#_Toc482883528)

[Hatzinger 2007 [33] 47](#_Toc482883529)

[Hostinar 2014 [34] 48](#_Toc482883530)

[Hostinar 2015 [35] 49](#_Toc482883531)

[Ji 2016 [36] 50](#_Toc482883532)

[Jones 2006 [37] 51](#_Toc482883533)

[Kelly 2008 [38] 52](#_Toc482883534)

[Khilnani 1993 [39] 53](#_Toc482883535)

[Kjolhede 2014 [40] 54](#_Toc482883536)

[Knutsson 1997 [41] 55](#_Toc482883537)

[Kryski 2013 [42] 56](#_Toc482883538)

[Kudielka 2004 [43] 57](#_Toc482883539)

[Kuhlman 2015 [44] 58](#_Toc482883540)

[Lashansky 1991 [45] 60](#_Toc482883541)

[Lopez-Duran 2015 [46] 61](#_Toc482883542)

[Lu 2014 [47] 62](#_Toc482883543)

[Lumeng 2014 [48] 63](#_Toc482883544)

[Martikainen 2013 [49] 64](#_Toc482883545)

[Martin 2011 [50] 65](#_Toc482883546)

[Matchock 2007 [51] 66](#_Toc482883547)

[Michels 2012 [52] 68](#_Toc482883548)

[Mills 2008 [53] 69](#_Toc482883549)

[Minkley 2012 [54] 71](#_Toc482883550)

[Morin-Major 2016 [55] 73](#_Toc482883551)

[Mrug 2016 [56] 75](#_Toc482883552)

[Netherton 2004 [57] 76](#_Toc482883553)

[Osika 2007 [58] 77](#_Toc482883554)

[Peckins 2012 [59] 78](#_Toc482883555)

[Plusquellec 2011 [60] 79](#_Toc482883556)

[Portnoy 2015 [61] 80](#_Toc482883557)

[Pruessner 1997 [62] 81](#_Toc482883558)

[Raikkonen 2010 [63] 82](#_Toc482883559)

[Rosmalen 2005 [64] 83](#_Toc482883560)

[Ross 1986 [65] 85](#_Toc482883561)

[Ruttle 2013 [66] 86](#_Toc482883562)

[Shirtcliff 2012 [67] 87](#_Toc482883563)

[Spinrad 2009 [68] 88](#_Toc482883564)

[Strahler 2010 [69] 89](#_Toc482883565)

[Stroud 2011 [70] 91](#_Toc482883566)

[Stupnicki 1995 [71] 93](#_Toc482883567)

[Susman 2007 [72] 94](#_Toc482883568)

[Trickett 2014 [73] 95](#_Toc482883569)

[Tsvetkova 1977 [74] 96](#_Toc482883570)

[Tzortzi 2009 [75] 97](#_Toc482883571)

[Vaillancourt 2008 [76] 98](#_Toc482883572)

[Vanaelst 2013 [77] 99](#_Toc482883573)

[Williams 2013 [78] 100](#_Toc482883574)

[Yfanti 2014 [79] 101](#_Toc482883575)

[Yong Ping 2014 [80] 102](#_Toc482883576)

[Zijlmans 2013 [81] 103](#_Toc482883577)

### Adam 2010 [1]

| **Methods** | **Design** Longitudinal study |
| --- | --- |
|  | **Setting** Chicago and Los Angeles, USA |
|  | **Timing**  Diurnal rhythm + CAR |
| **Participants** | ***n=*** 230 |
|  | **Subjects** High school Juniors from two diverse public high schools. “Adolescents high on neuroticism were oversampled, such that 60% of the resulting sample scored in the top third of the neuroticism distribution.” |
|  | **Age** 17.04 ± 0.36 yr |
|  | **Sex** 173 females and 57 males (of note “the greater predominance of females over males in this sample is accounted for by the fact that individuals with high levels of neuroticism were oversampled ”) |
|  | **Exclusion criteria** if adolescents “used corticosteroid-based medications, had psychotic symptoms, provided insufficient cortisol data, or had more than 3  months delay between baseline psychopathology and cortisol measurements” |
| **Protocol** | “The current analyses used three assessments: baseline questionnaires/ interviews, a cortisol assessment (within 3 months after baseline, average = 39 days) and follow-up interviews (approximately 1 year after the cortisol assessment, average = 368 days) (…) Measures of basal cortisol activity included: wake-up, wake-up plus 40 min values, and bedtime values, size of the CAR (wake-up plus 40 min minus wake-up cortisol level), slope of the diurnal cortisol rhythm from wake-up to bedtime (bedtime minus wake-up level divided by total time awake), and average cortisol (calculated by taking the area under the curve (AUC) defined by all cortisol data points across the day, divided by the total time awake). Each measure was averaged across the 3 collection days.” Cortisol samples: “ Salivary cortisol was gathered six times per day over 3 consecutive typical weekdays during the school year: at wake-up, 40 min after waking, at approximately 3, 8, and 12 h post-awakening (signaled by a programmed watch), and bedtime. “  Ethical approval: not described in the manuscript. However, at the website of the Youth Emotion Study (<http://anxiety.psych.ucla.edu/yep.php> ) it is described that subjects needed parental consent. |
| **Outcomes** | **Primary** to predict clinical diagnoses of major depressive disorder from the CAR |
|  | **Secondary** to predict clinical diagnoses of major depressive disorder from waking cortisol levels, bedtime cortisol levels, the size of the CAR, average cortisol, and the slope of the diurnal cortisol rhythm across the waking day. |
| **Results** | Figure 3: “the overall diurnal cortisol curves appear lower for males than for  females, for both genders, the cortisol awakening responses is more pronounced for youth who go on to develop MDD by follow-up”  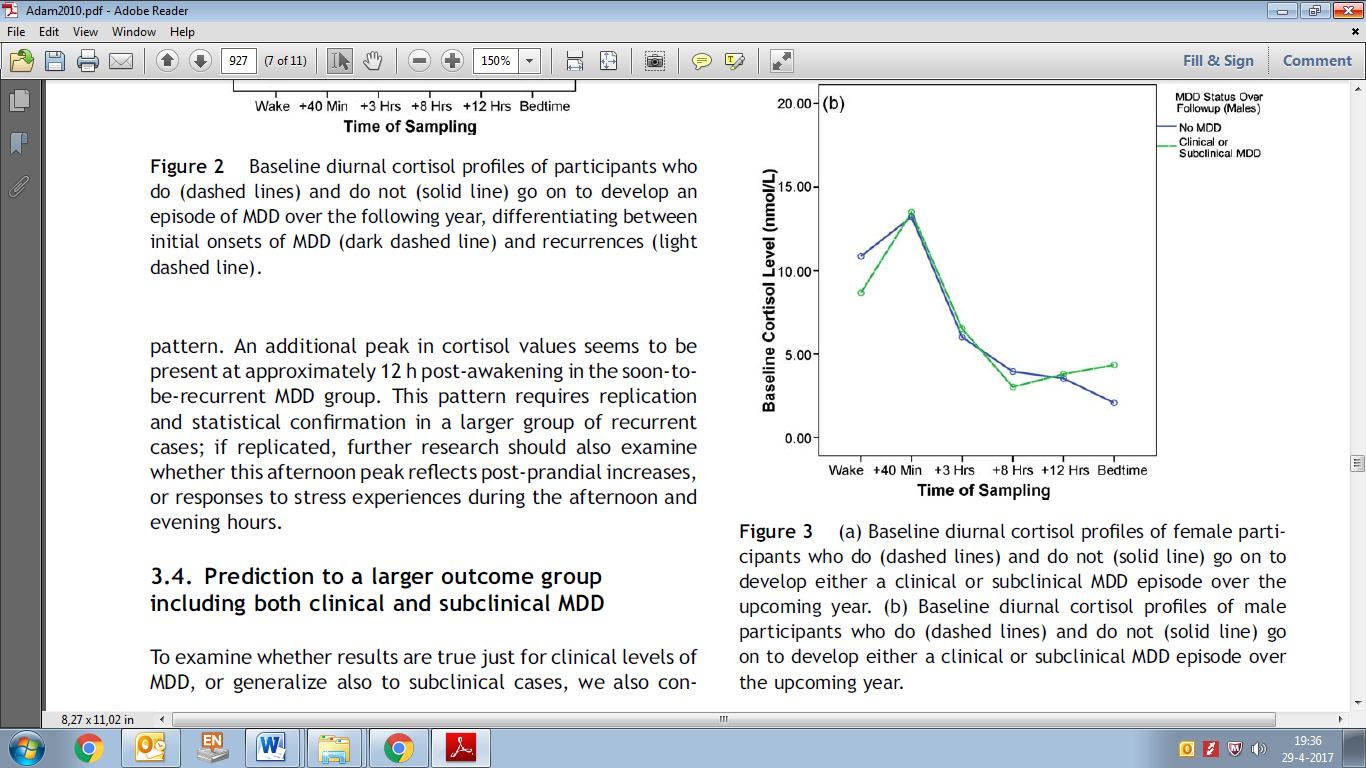  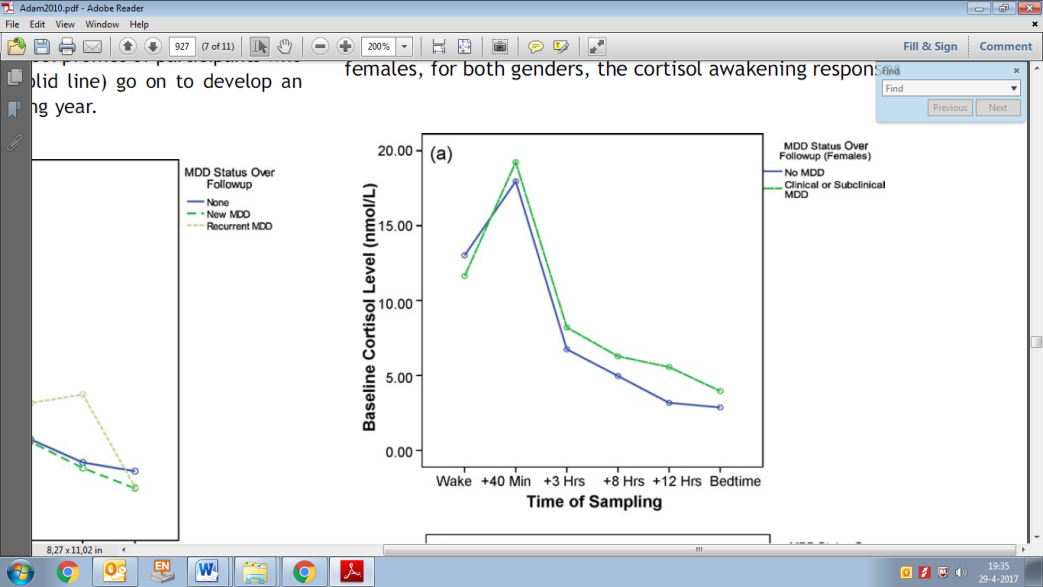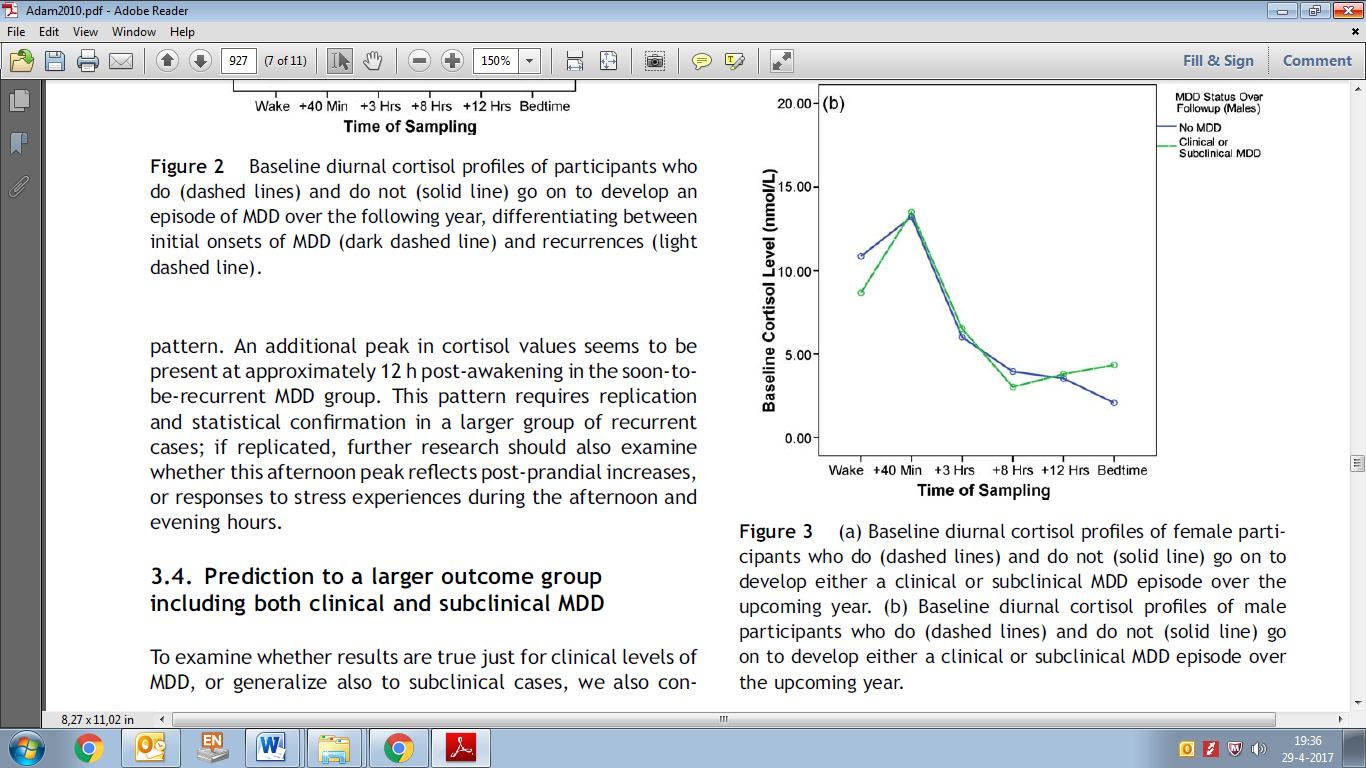 |

### Allen 2009 [2]

| **Methods** | **Design** Cross-sectional |
| --- | --- |
|  | **Setting** University of California |
|  | **Timing**  Laboratory Pain Tasks (pressure task, heat task, cold pressor task) |
| **Participants** | ***n=*** 235 |
|  | **Subjects**  Participants were recruited from the greater Los Angeles, California, area through mass mailings, posted advertisements, and classroom presentations. Eligible subjects were self-reported healthy children and adolescents, aged 8 to 18 years. In the present study, the term healthy referred to children who, by parent and self-report, had no acute or chronic illness such as a heart condition or arthritis, recent surgery, injury to any limb, history of frostbite, history of fainting spells, or develop-mental delay. |
|  | **Age** 8-18 years (mean: 12.7 years, SD 2.9) |
|  | **Sex** 119 boys, 116 girls |
|  | **Exclusion criteria** Participants were excluded from study participation for the following reasons: (1) acute or chronic illness at the time of study participation; (2) developmental delay or significant anatomic impairment that would preclude understanding of the study procedures (eg, developmental age <8 years) as assessed by parents and principal investigator, or participation in pain-induction procedures (eg, arm immersion in cold water); or (3) daily use of opioid medication. |
| **Protocol** | The timeline for sample collection is illustrated in Figure 1 . Salivary cortisol samples were obtained from the subjects after they entered the laboratory ([SCb] baseline), after the completion of all pain tasks (SC1), and at the end of the session ([SC2] 20 minutes after the previous assessment). Blood cortisol samples were also obtained after the completion of the pain tasks (BC1) and at the end of the session (BC2), 20 minutes later, for the assessment of recovery.  Ethical approval: obtained |
| **Outcomes** | **Primary** To examine cortisol-oain response relationships across 2 methods of assessment (sampling salivary and blood cortisol levels). |
|  | **Secondary** To characterize changes in cortisol levels in response to a series of laboratory pain tasks, as well as potential sex differences in patterns of cortisol reactivity. Finally, we tested for sex differences in the cortisol-pain response relationship. |
| **Results** | “With regard to sex differences in cortisol levels, boys had marginally higher salivary cortisol at SCb compared to girls (P=0.05), but no significant sex differences emerged at SC1 and SC2. There were also no significant sex differences found for BC1 and BC2.”  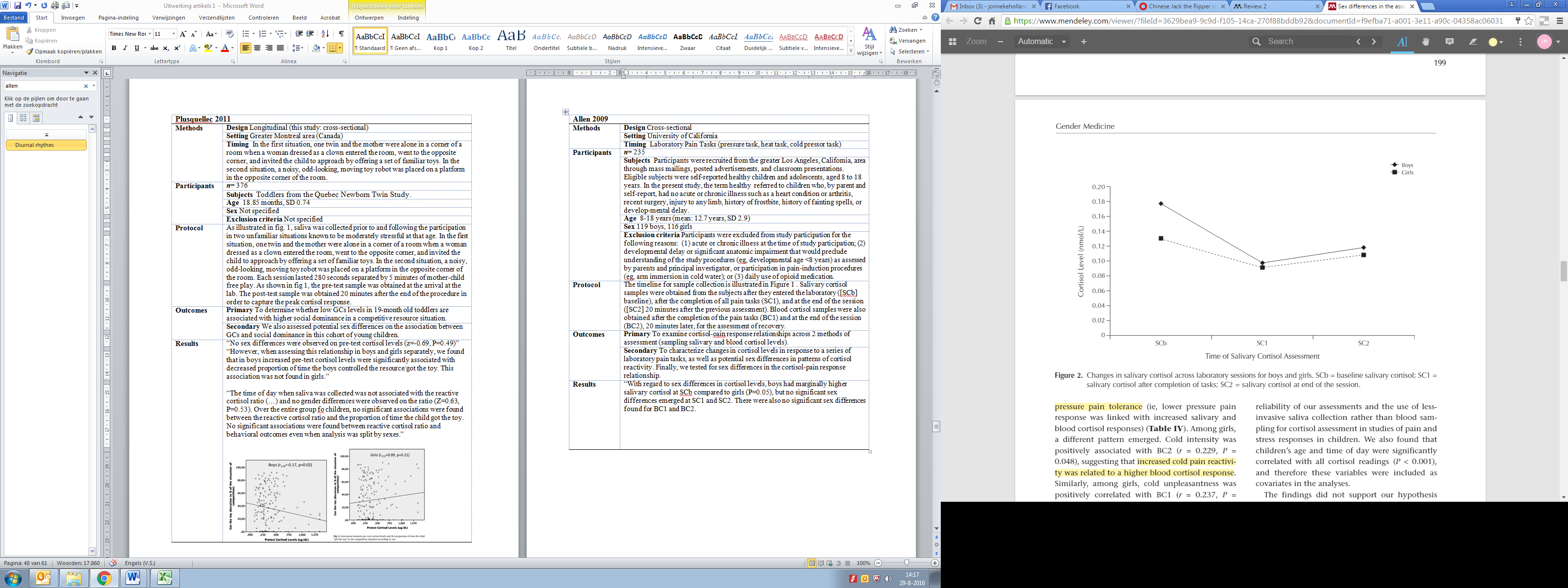  “In partial correlations controlled for age and time of day among boys, there was a significant association between pressure tolerance and SC1 and BC1, indicating greater pressure pain tolerance (ie, lower pressure pain response was linked with increased salivary and blood cortisol responses). Among girls, a difference pattern emerged. Cold intensity was positively associated with BC2, suggesting that increased cold pain reactivity was related to a higher blood cortisol response. Similarly, among girls, cold unpleasantness was positively correlated with BC1. In girls, the associations between cold intensity and BC1 and SC1, cold unpleasantness and SC1 and cold unpleasantness and BC2 were not statistically significant. |

### Bae 2015 [3]

| **Methods** | **Design** cohort study |
| --- | --- |
|  | **Setting** Leipzig, Germany |
|  | **Timing**  Diurnal rhythm + TSST-C |
| **Participants** | ***n=*** circadian activity: 70 healthy children; TSST-C: 81 healthy children |
|  | **Subjects** healthy controls that were part of a large cohort study of the Leipzig Research Centre for Civilization Diseases (LIFE study) |
|  | **Age** circadian activity: 10.7 ± 1.7 yrs. Mean of Tanner stage of breast/testis development and Tanner stage of pubic hair development (self-rating according to Morris and Udry, 1980): stage 1.1 ± 0.8  TSST-C: 10.8 ± 1.8 yrs. Mean of Tanner stage: 1.1 ± 0.9 |
|  | **Sex** circadian activity: 29 males and 41 females; TSST-C: 35 males and 46 females |
|  | **Exclusion criteria** no fluency in German, IQ lower than 80 confirmed by the Culture Fair Intelligence Test-revised version (CFT-20-R; Weiss, 2006), and concurrent endocrine diseases or concomitant administration of glucocorticoid medications. |
| **Protocol** | “Saliva samples (n = 2893) from children with internalizing (n = 55) or externalizing disorders (n = 33) and healthy children (n = 81) were analyzed for cortisol, cortisone, and AA under circadian conditions and TSST-C”  Diurnal rhythm: : “Saliva was collected upon awakening (time, mean ± SD: 06:48 ± 0:58), 30 min after waking, and 30 min before bedtime (time, mean ± SD: 20:18 ± 1:18) on Monday, Wednesday and Friday. The mean values of the three days for each time-point were used”  TSST-C: “was performed from 15:35 to 15:55 at the research center in three phases: 5 min of preparation for story-telling, 5 min of story-telling, and 5 min of arithmetic problem solving in front of a panel of two researchers. Saliva was collected at -30, -10,-1, +1, +10, +20, +30 and +60 min with TSST-C at  0 min.”  Ethical approval: obtained |
| **Outcomes** | **Primary** “to determine the response patterns of cortisol, cortisone, and AA under both circadian conditions and the Trier Social Stress Test for Children (TSST-C)” |
|  | **Secondary** “2) which reactivity index is most suitable to differentiate internalizing or externalizing disorders from controls, and to explore 3) the interaction between AA and cortisol in the presence of internalizing or externalizing disorders” |
| **Results** | Gender (p = .04) was a significant positive predictors for cortisol (boy = 1,girls = 2) (Table 3)  TSST: no gender differences appear to be present |


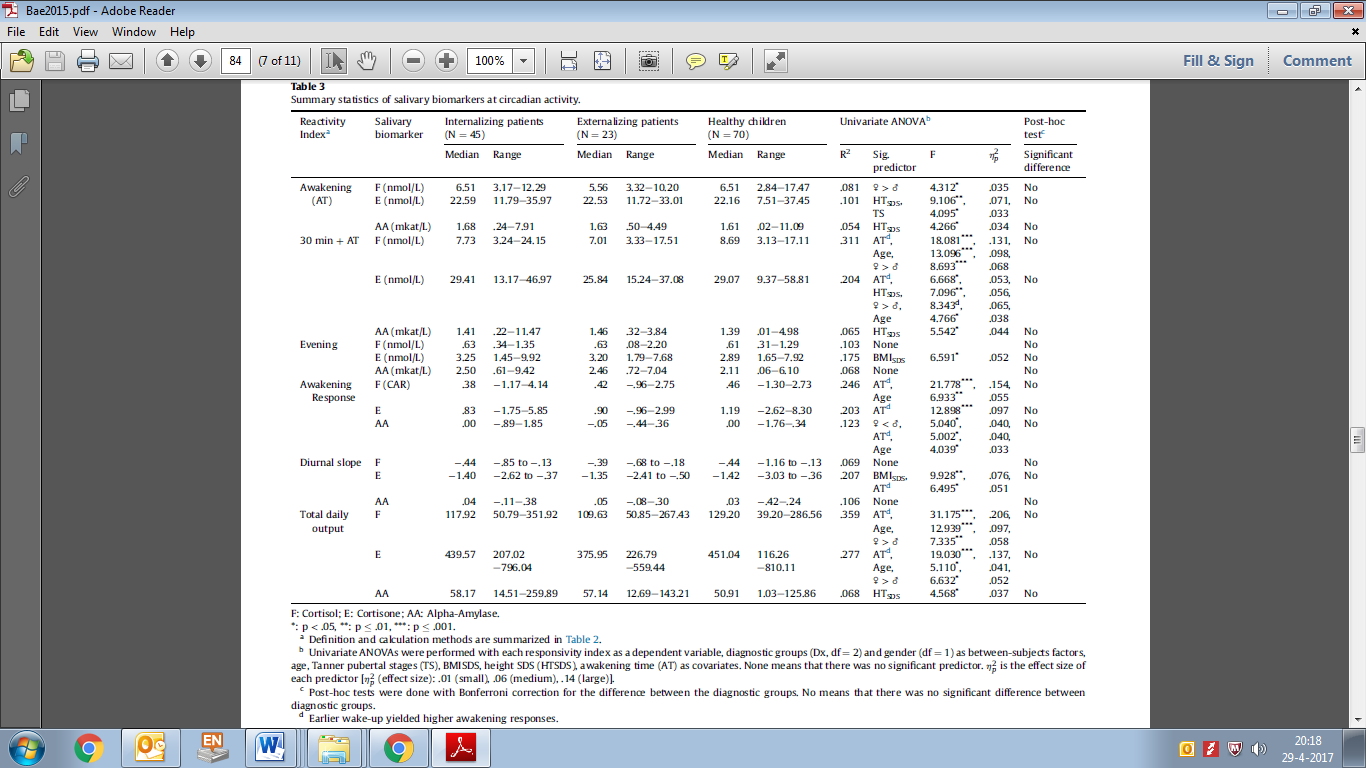

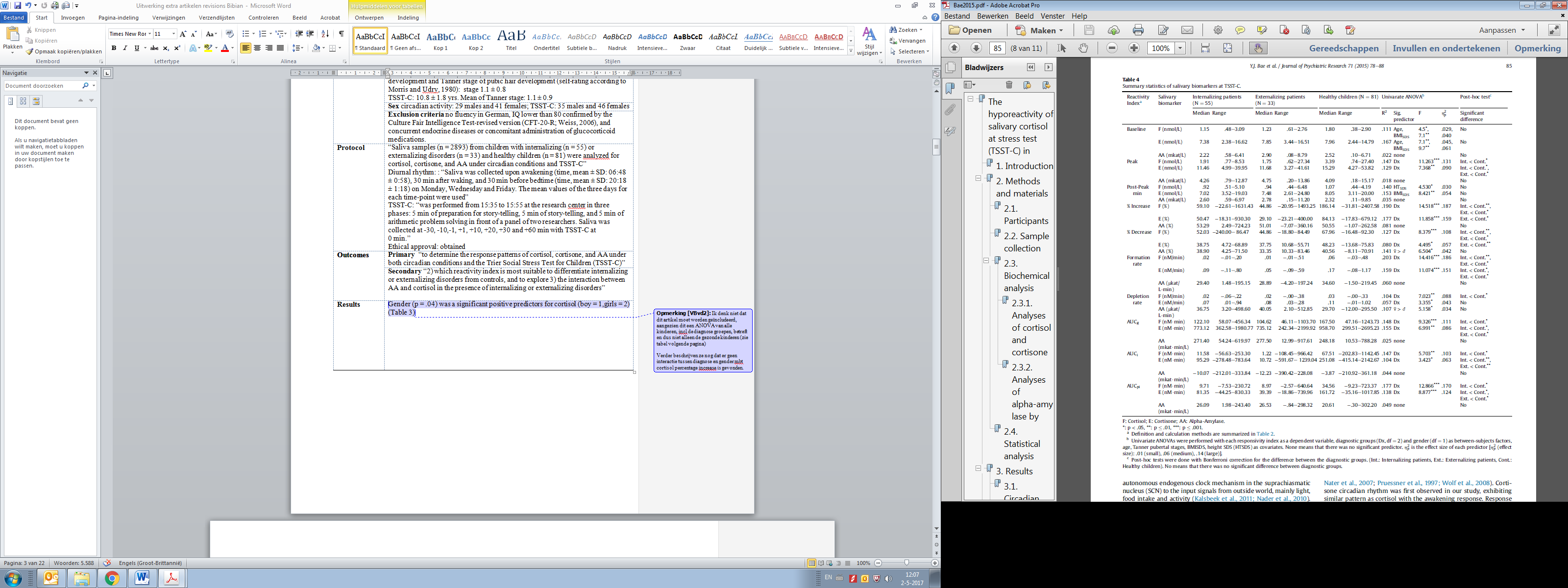


### Barbosa 2012 [4]

| **Methods** | **Design** Cross-sectional study |
| --- | --- |
|  | **Setting** Saõ Paulo, Brazil |
|  | **Timing**  Diurnal rhythm |
| **Participants** | ***n=*** 145 |
|  | **Subjects** “public school students were recruited after screening. A total of 145 public school students (49 boys and 96 girls), aged 8–14-year-old with no systemic diseases or communication and ⁄ or neuro-muscular problems, composed the ﬁnal sample” |
|  | **Age** 8-10yr group: 9.0 ± 0.8yr; 11-14yr group: 11.9 ± 1.0yr; |
|  | **Sex** 8-10yr group: 29 boys and 44 girls; 11-14yr group:20 boys and 52 girls |
|  | **Exclusion criteria** systemic diseases, communication and/or neuromuscular problems, neurological or psychiatric disorders  “Exclusion criteria were facial trauma, neurological or psychiatric disorders, use of dental prostheses, current use of medications that could interfere with the central nervous system (e.g., antidepressants, muscle relaxants, narcotics, or non-steroidal anti-inﬂammatory drugs), previous or current orthodontic treatment, or other painful orofacial conditions that could interfere with TMD diagnoses. Participants who did not collect saliva or submitted insuﬃcient ⁄ contaminated samples were also excluded.” |
| **Protocol** | “On a weekday, after waking normally, the subjects chewed the cotton rolls for  two minutes, until they had been soaked with saliva, and then placed them into the salivettes. The first sample was taken 30 min after waking (fasting), and the second  sample was taken at night (bedtime) (…) The diurnal decline of salivary cortisol data (in µg ⁄ dl) was calculated as the difference between cortisol levels at  30 min after waking and at bedtime”  Saliva samples: “Saliva was collected 30 min after waking and at night.”  Ethical approval: obtained |
| **Outcomes** | **Primary** “the relationships between oral health, self-perceived oral health, QoL, and emotional status, such as symptoms of anxiety and depression and salivary cortisol concentration (as a biomarker of stress), in 8–14-year-old public school students” |
|  | **Secondary** similar as the primary outcome but then for oral well-being |
| **Results** | The diurnal decline of salivary cortisol data  8-10yr group: 0.15 ± 0.11 µg ⁄ dl in boys and 0.12 ± 0.10 µg ⁄ dl in girls  11-14yr group: 0.22 ± 0.15 µg ⁄ dl in boys and 0.25 ± 0.16 µg ⁄ dl in girls  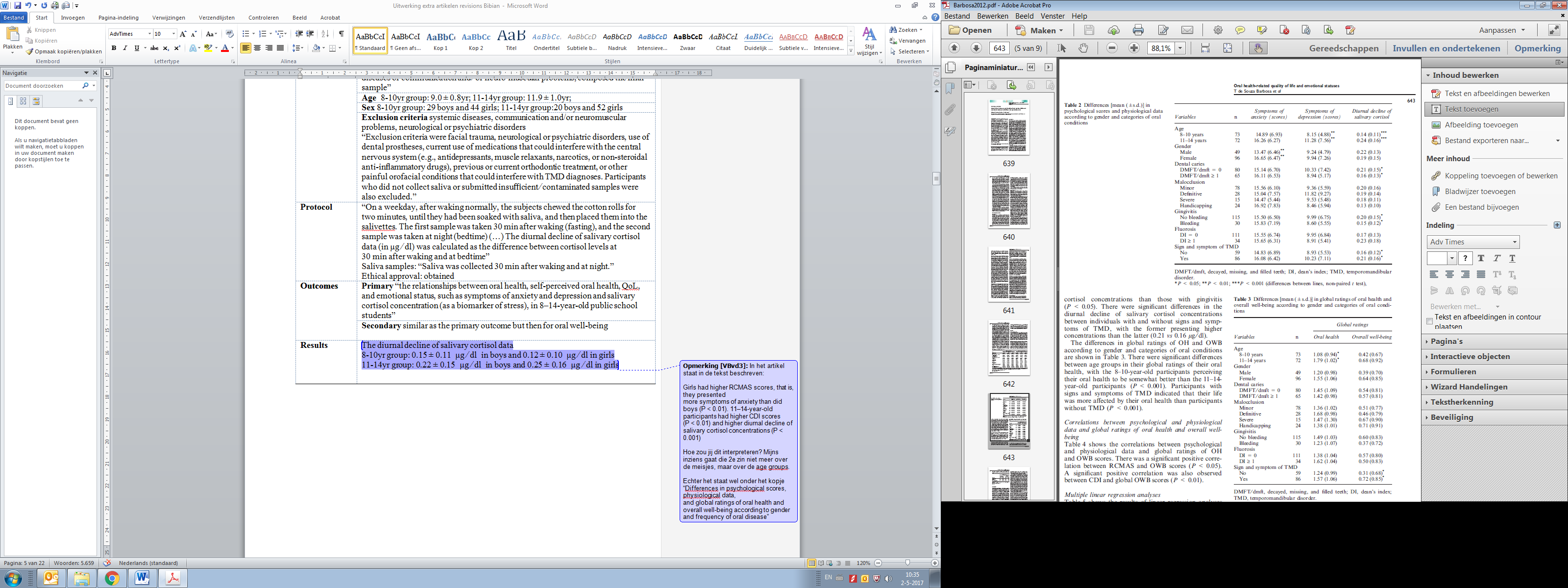 |

### Bartels 2003 [5]

| **Methods** | **Design** Longitudinal |
| --- | --- |
|  | **Setting** The Netherlands |
|  | **Timing**  Diurnal rhythm |
| **Participants** | ***n=*** 180 twin pairs = 360 subjects |
|  | **Subjects** “Young twins and multiples are recruited a few weeks or months after their birth. Currently around 50% of all newborn multiples in The Netherlands are registered. The initial sample of 209 twin pairs was selected on the basis of age and zygosity of the twins and their city of residence.” “For the determination of cortisol levels, saliva was collected in 1999/2000 when the twins were 12 years old. Mean age of the subjects was 12 years (80% ranging from 11 years and 11 months to 12 years and 1 month). Zygosity of the same-sex twins was established by either blood group polymorphisms or DNA analyses. The initial twin sample at age 12 consisted of 47 monozygotic female (MZF), 37 dizygotic female (DZF), 42 monozygotic male (MZM), 44 dizygotic male (DZM), and 39 dizygotic pairs of opposite sex (DOS). Because of difficulties during saliva collection or laboratory analyses, data of 29 twin pairs were not usable, resulting in a final sample of 180 twin pairs.” |
|  | **Age** 12 years (80% ranging from 11 years and 11 months to 12 years and 1 month) |
|  | **Sex** Not completely specified |
|  | **Exclusion criteria** Difficulties with saliva collection |
| **Protocol** | “Four samples of cortisol per day on two consecutive days were collected”  “On the first day the first sample (day 1—0730H) was taken in the morning just be-fore getting up (still lying in bed) (mean time 0728H), the second (day 1—0830H) sample was taken at least half an hour after getting up but before going to school (mean time 0817H), the third sample (day 1—1230H) was taken before lunch (mean time 1234H), and the fourth sample (day 1—2030H) was taken in the evening (mean time 2032H). On the second day the same schedule was adapted for four repeated samples. The twins were instructed to collect saliva on two school days to restrict the awakening time and time of sampling”  Ethical approval: not specified |
| **Outcomes** | **Primary** “to determine the heritability of variation in daytime cortisol levels in children” |
|  | **Secondary** |
| **Results** | “The results of the bivariate model-fitting procedure for each time point demonstrate different contributions of genetic and environmental influences at the four cortisol measures (Table V). However, no significant sex differences have been found.”  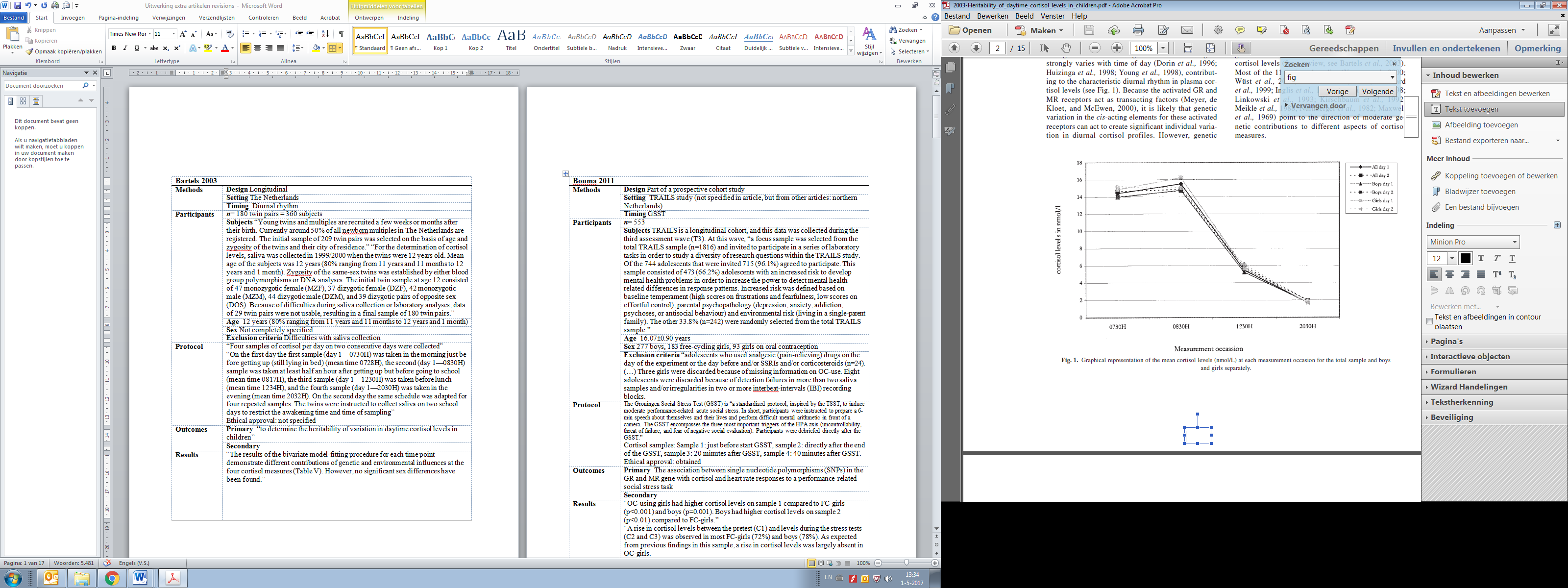 |

### Bouma 2009 [6]

| **Methods** | **Design** Longitudinal / cohort study |
| --- | --- |
|  | **Setting** Five municipalities in the Northern part of the Netherlands |
|  | **Timing**  CAR and Groningen Social Stress Test (GSST) |
| **Participants** | ***n=*** 644 |
|  | **Subjects**  Participants were selected from five municipalities in the North-ern part of the Netherlands. Only part of these subjects were invited to perform a series of laboratory tasks. Adolescents with a high risk of mental health problems had a greater chance of being selected for the experimental session. High risk was defined based on baseline temperament (high scores on frustration and fearfulness, low scores on effortful control), parental psychopathology (depression, anxiety, addiction, psychoses, or antisocial behavior), and environmental risk (living in a single-parent family). In total, 66.0% of the sample had at least one of the above-described risk factors. The remaining 34.0% were randomly selected from the total TRAILS sample |
|  | **Age** mean age 16.13, S.D. = 0.59 |
|  | **Sex** 352 boys, 83 girls in follicular phase, 84 girls in luteal phase, 125 girls on OAC |
|  | **Exclusion criteria** Not specified |
| **Protocol** | The sessions lasted about 3 h and 15 min, and started between 08:00 h and 09:30 h (morning sessions, 49%) or between 01:00 h and 02:30 h (afternoon sessions, 51%).  The participants were asked to collect two morning saliva samples on the day of the experimental session, one directly after waking up (Co1) (mean time of awakening = 07:39 h, S.D. = 1:10 h) and one 30 min later (Co2).  Ce1: taken after filling in questionnaire and a waiting period of 35 minutes, before starting the GSST; Ce2: just before the start of the GSST; Ce3: directly after the end of the GSST; Ce4 and 5: taken 20 and 40 minutes after the end of the GSST  Ethical approval: obtained |
| **Outcomes** | **Primary** “We studied the effects of gender, menstrual cycle phase and oral contraceptive (OC) use on cortisol responses” |
|  | **Secondary** |
| **Results** | “We found significant group differences for (…) Co1, Co2, Ce3, CAR and maximum increase to the GSST.”  CAR: “Boys had lower morning cortisol levels than girls, but boys and girls did not differ in their response to awakening. OC users and free-cycling girls did not differ in their morning cortisol levels, but OC using girls showed a blunted response to awakening. We did not find any effect of menstrual cycle phase.”  GSST: “Boys and free-cycling girls differed with respect to the overall cortisol levels during the GSST. Furthermore, we observed a significant interaction of gender with the cortisol response, particularly due to differences in the quadratic trend, indicating that boys reacted differently to the social stress test than free-cycling girls. **Cortisol responses were stronger in boys** than girls. Cortisol levels and responses were also modified by OC use. Differences between OC users and free-cycling girls pertained to both the linear and the quadratic trend in the cortisol response. As opposed to free-cycling girls, cortisol levels of OC users linearly decreased during the GSST and no response was displayed whatsoever, no in the morning and not in the afternoon sessions. Girls in the luteal phase did not differ from girls in the follicular phase regarding cortisol levels or responses towards the GSST.  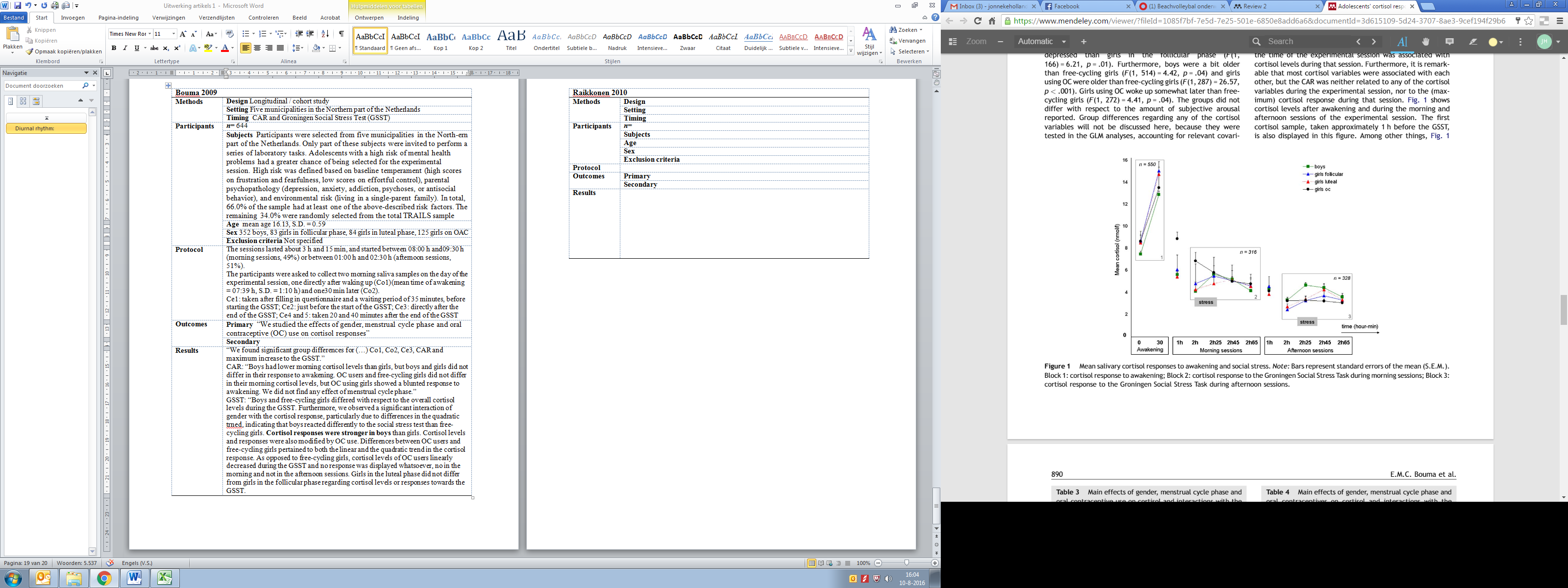  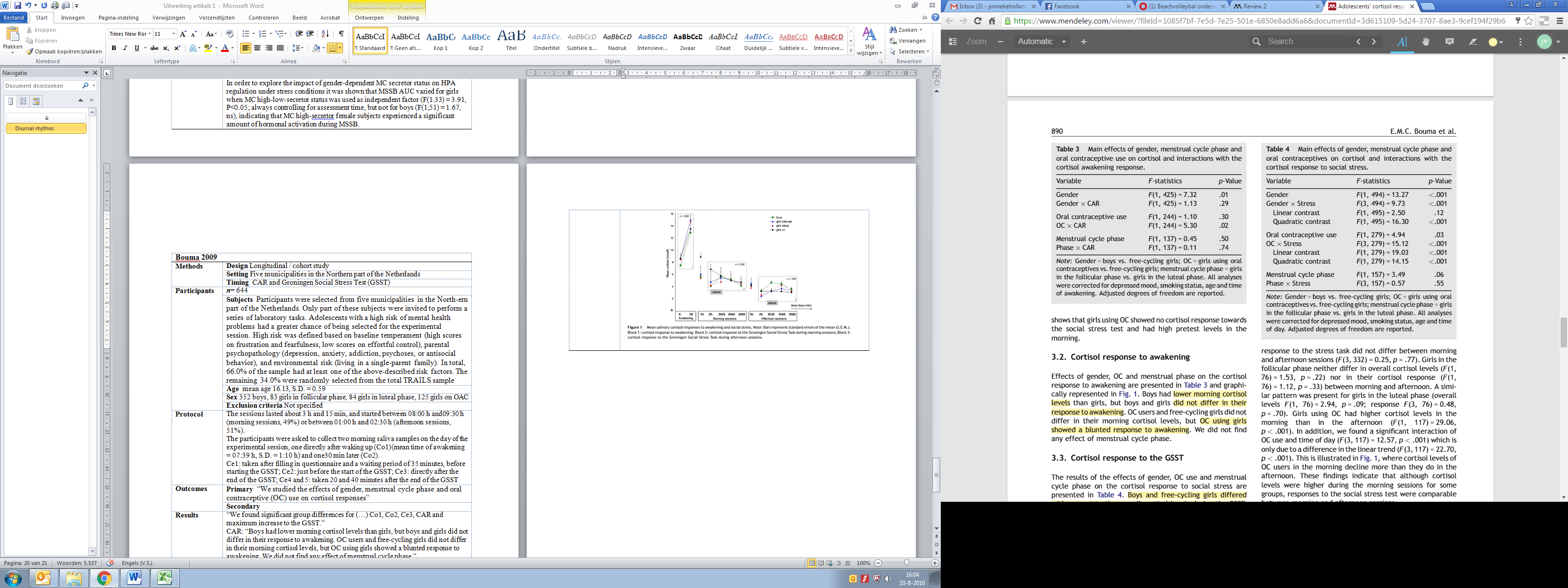 |

### Bouma 2011 [7]

| **Methods** | **Design** Part of a prospective cohort study |
| --- | --- |
|  | **Setting** TRAILS study (not specified in article, but from other articles: northern Netherlands) |
|  | **Timing** GSST |
| **Participants** | ***n=*** 553 |
|  | **Subjects** TRAILS is a longitudinal cohort, and this data was collected during the third assessment wave (T3). At this wave, “a focus sample was selected from the total TRAILS sample (n=1816) and invited to participate in a series of laboratory tasks in order to study a diversity of research questions within the TRAILS study. Of the 744 adolescents that were invited 715 (96.1%) agreed to participate. This sample consisted of 473 (66.2%) adolescents with an increased risk to develop mental health problems in order to increase the power to detect mental health-related differences in response patterns. Increased risk was defined based on baseline temperament (high scores on frustrations and fearfulness, low scores on effortful control), parental psychopathology (depression, anxiety, addiction, psychoses, or antisocial behaviour) and environmental risk (living in a single-parent family). The other 33.8% (n=242) were randomly selected from the total TRAILS sample.” |
|  | **Age** 16.07±0.90 years |
|  | **Sex** 277 boys, 183 free-cycling girls, 93 girls on oral contraception |
|  | **Exclusion criteria** “adolescents who used analgesic (pain-relieving) drugs on the day of the experiment or the day before and/or SSRIs and/or corticosteroids (n=24). (…) Three girls were discarded because of missing information on OC-use. Eight adolescents were discarded because of detection failures in more than two saliva samples and/or irregularities in two or more interbeat-intervals (IBI) recording blocks. |
| **Protocol** | The Groningen Social Stress Test (GSST) is “a standardized protocol, inspired by the TSST, to induce moderate performance-related acute social stress. In short, participants were instructed to prepare a 6-min speech about themselves and their lives and perform difficult mental arithmetic in front of a camera. The GSST encompasses the three most important triggers of the HPA axis (uncontrollability, threat of failure, and fear of negative social evaluation). Participants were debriefed directly after the GSST.”  Cortisol samples: Sample 1: just before start GSST, sample 2: directly after the end of the GSST, sample 3: 20 minutes after GSST, sample 4: 40 minutes after GSST. Ethical approval: obtained |
| **Outcomes** | **Primary** The association between single nucleotide polymorphisms (SNPs) in the GR and MR gene with cortisol and heart rate responses to a performance-related social stress task |
|  | **Secondary** |
| **Results** | “OC-using girls had higher cortisol levels on sample 1 compared to FC-girls (p<0.001) and boys (p=0.001). Boys had higher cortisol levels on sample 2 (p<0.01) compared to FC-girls.”  “A rise in cortisol levels between the pretest (C1) and levels during the stress tests (C2 and C3) was observed in most FC-girls (72%) and boys (78%). As expected from previous findings in this sample, a rise in cortisol levels was largely absent in OC-girls.  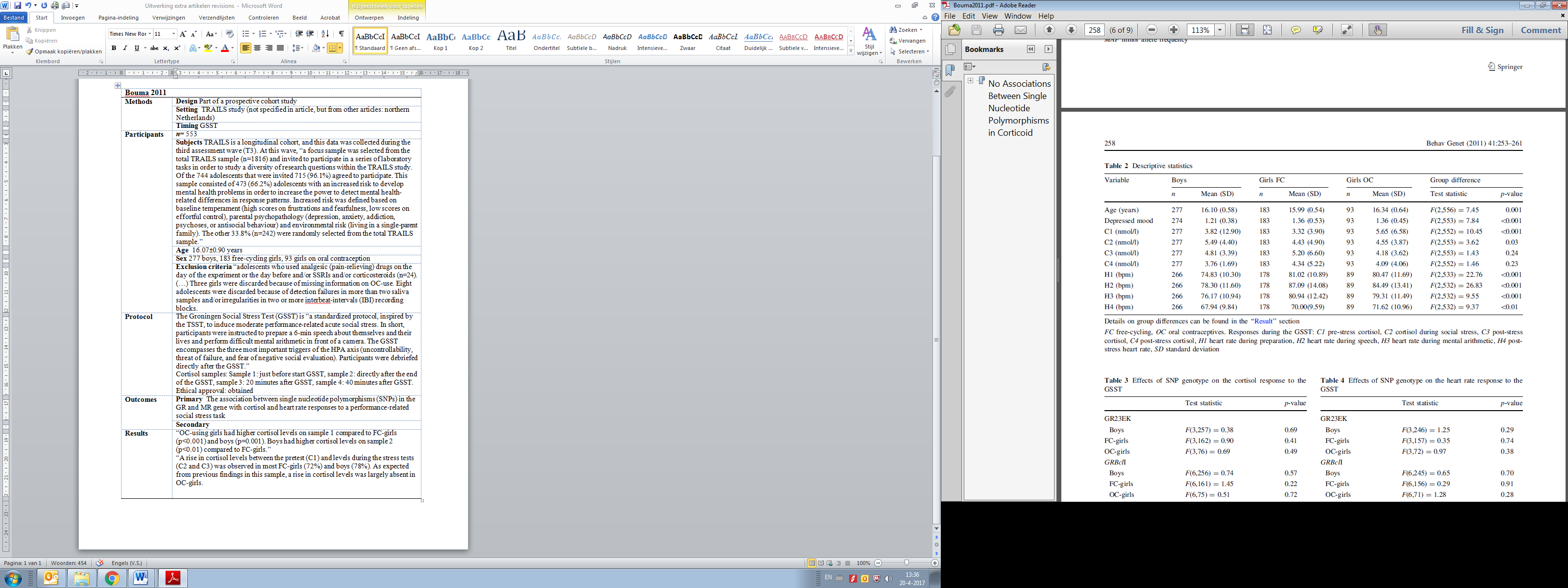 |

### Bright 2014 [8]

| **Methods** | **Design** Cross-sectional |
| --- | --- |
|  | **Setting** Not specified |
|  | **Timing**  CAR |
| **Participants** | ***n=*** 47 |
|  | **Subjects** Toddlers. “Average maternal age was 34 years (SD ¼ 4.48) and 36 years (SD ¼ 5.23) for co-parents. Most (85%) infants were born full-term (i.e., 􀀂37 weeks). This sample was predominately (80%) Caucasian or African-American (7%) with most mothers having at least post-graduate education and co-parents at least a college degree.” |
|  | **Age** “12 (n = 14, M = 12.19, SD = .58), 18 (n = 21, M = 18.13, SD = .56), and 24 months (n = 12, M = 24.33, SD = .56).” |
|  | **Sex** 30 boys, 17 girls |
|  | **Exclusion criteria** Not specified |
| **Protocol** | “Families were recruited via telephone from public birth listings. After agreeing to participate, parents were provided detailed written and video instructions for collecting data. Children wore an actigraph (described further below) for one night. On the subsequent morning (collection day) parents collected two saliva samples from their child: when their child awoke from morning sleep (waking) and 30–45 min post morning waking (post-waking). Parents were free to choose any day of the week for collection day”  “For waking, parents were instructed to collect saliva samples immediately after awakening and before feeding to prevent contamination of the specimen by food or drink. Parents were instructed that their child may eat/drink or take medication (as necessary) after waking but to wait at least 20–30 min after feeding to collect a post-waking sample”  Ethical approval: not specified |
| **Outcomes** | **Primary** (i) examine the presence of and developmental changes in the CAR and sAA-AR in a toddler sample |
|  | **Secondary** (ii) determine if and how sleep relates to the CAR and sAA-AR in toddlers |
| **Results** | “Independent t-tests for waking cortisol levels, rate of change and percent increase by sex indicated that there were no signiﬁcant differences between males and females, all ps > .05” |

### Carrion 2002 [9]

| **Methods** | **Design** case-control design |
| --- | --- |
|  | **Setting** California, USA |
|  | **Timing**  Diurnal rhythm |
| **Participants** | ***n=*** 31 age- and gender-matched healthy control subjecs |
|  | **Subjects** “A healthy control group (n =31) was obtained from an archived sample in our laboratory. This sample was derived from the siblings of participants in a study of children with fragile X and selected for comparability to the age  and gender composition of the PTSD group.” |
|  | **Age** mean age 10.9 yrs. “Children’s median pubic hair Tanner stage was 2; for girls, median breast Tanner stage was 3; for boys, median genital Tanner stage was 2.” |
|  | **Sex** 18 boys and 13 girls |
|  | **Exclusion criteria** history of neurologic disorders; and history of alcohol or drug abuse/dependence, scores outside the clinical range for either internalizing or externalizing symptoms or lack of subjective or parent-observed distress or dysfunction. |
| **Protocol** | “Participants’ pubertal development was determined by self-report. Participants selected from drawings with written descriptions representing the five Tanner Stages (Marshal and Tanner 1970) of pubic hair development and genital development for boys and breast development for girls (…)  Saliva was collected four times a day (prebreakfast, prelunch, predinner, and prebed) over the course of 3 days producing 12 samples. To maximize appropriate collection, detailed instructions and an illustration were provided to parents and children regarding the collection of saliva samples. A handout with a checklist indicating all 12 required collection times was provided for collection monitoring (…) As recommended for increased reliability (see Gunnar 2001) an aggregate score from the 3 days (i.e., the mean score across the assessment days) was created for each time period so that each participant had one prebreakfast, prelunch, predinner, and prebed sample. Participants missing three samples from the same time point were not included in this study”  Ethical approval: Obtained |
| **Outcomes** | **Primary** Baseline diurnal cortisol levels in children with history of trauma who already have manifested symptoms of PTSD compared with age- and gender-matched controls. |
|  | **Secondary** N/A |
| **Results** | “Differences in the cortisol levels of boys and girls were found. Overall, results  indicated that girls showed significantly higher cortisol levels than boys but only in the PTSD group (…) No significant differences across gender were found in the control group” |

### Chiodo 2011 [10]

| **Methods** | **Design** Cross-sectional |
| --- | --- |
|  | **Setting** An official youth Taekwondo competition |
|  | **Timing**  Taekwondo competition |
| **Participants** | ***n=*** 16 |
|  | **Subjects** Black belt athletes (competition experience: 3±1 years). “The participants included in this study had to fulﬁll the following inclusion criteria: (1) compete at the black belt level; (2) have at least 5 years of previous training (consisting of three 2 hour sessions/week); (3) have obtained at least the fourth position in the previous Italian Youth Championship; and (4) have won their respective matches under investigation.: |
|  | **Age** Boys: 14±0 years, girls: 13±1 years |
|  | **Sex** 10 boys, 6 girls |
|  | **Exclusion criteria** “only the matches scheduled between 12:00 and 13:00 hours were considered” |
| **Protocol** | “Cotton swabs and saliva collecting tubes (Salivette, Sarstedt, Germany) were used to obtain saliva samples (40.05 mL) in the morning (09:00 hours), 15 min before (pre-match) and right after (post-match) the competition, and during the recovery phase (30 min and 90 min recovery) with the athletes resting.”  Ethical approval: obtained |
| **Outcomes** | **Primary** “to evaluate the heart rate (HR), hormonal (i.e., sC, sA-A), and mood (POMS) responses of young female and male Taekwondo athletes during their competition and to investigate whether a relationship exists between sA-A, sC, and POMS scores before and after the match” |
|  | **Secondary** |
| **Results** | “For sC values, the main eﬀects emerged for gender (F(1, 14) 5 8.74, P 5 0.01, ES 5 0.37) and sampling (F(4, 56) 5 0.45; Po0.0001). Although lower values were obtained from female athletes (Fig. 3(a)) as opposed to their male counterparts (Fig. 3(b)), sC showed a robust pattern, with a decrease in the morning to pre-match values, an increase after the match, reaching the peak values at 30 min of the recovery, and the lowest values at 90 min of the recovery. For female athletes, post hoc analysis showed diﬀerences (P ranging from 0.005 to 0.0001, ES ranging from 0.48 to 0.67) only between peak and the other sC values. For male athletes, post hoc diﬀerences (P ranging from 0.01 to o0.0001, ES ranging from 0.53 to 0.82) emerged between peak values and pre-, post-match, and 90 min recovery sC collections. Furthermore, for male athletes, sC values recorded at 90 min of recovery showed diﬀerences with respect to morning (P5 0.003, ES 5 0.49) and post-match (P5 0.01, ES 5 0.56) values. With respect to pre-match values, post-match sC increments were 58%and 24% for female and male athletes, respectively. The corresponding picture for increments between pre-match and peak sC values was 199% and 73% for female and male athletes, respectively.”  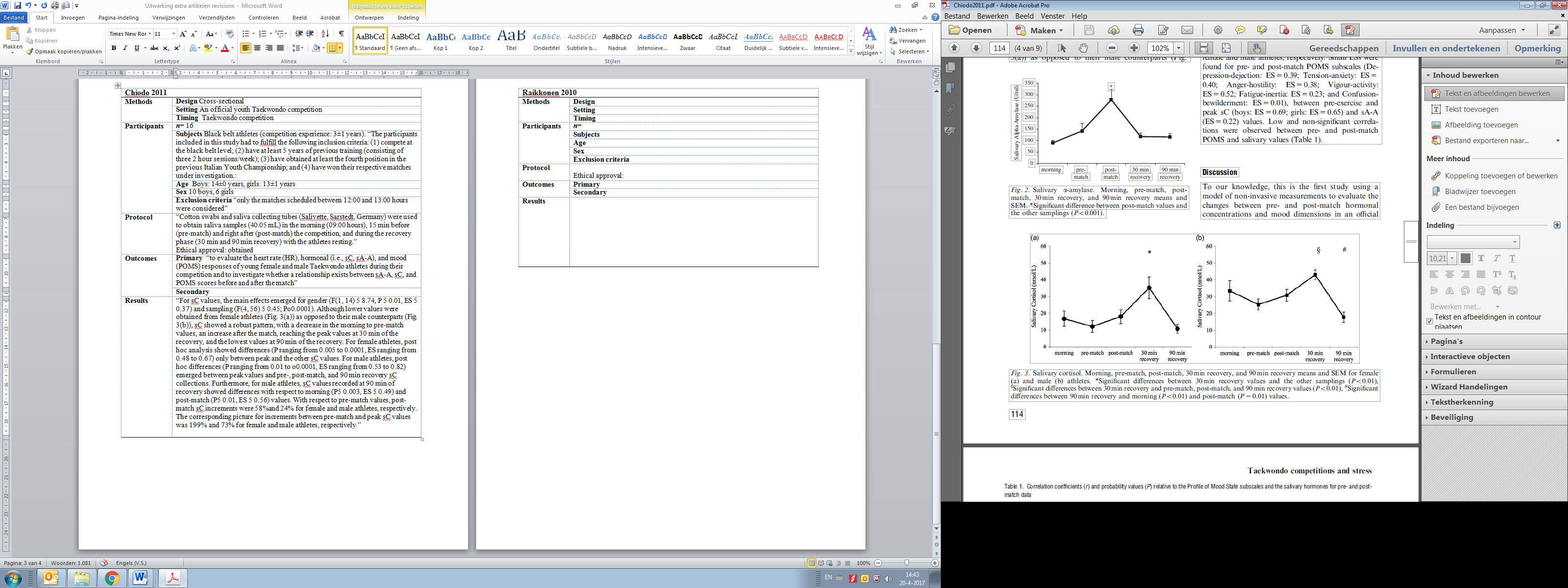 |

### Covelli 2012 [11]

| **Methods** | **Design** Exploratory descriptive |
| --- | --- |
|  | **Setting** Low socioeconomic community in Florida |
|  | **Timing**  Physiologic stress by cold water hand immersion |
| **Participants** | ***n=*** 106 |
|  | **Subjects**  Participants were recruited from a historically African American high school (9thY 12th grades) with student population of 1000, located in an urban, low socioeconomic community in Florida. Participants were drawn from students (N = 150) enrolled in 4 classes of Personal Fitness and Life Management over two 9-week course sections. Within the school curriculum, these courses are generally taken in the 9th and 10th grades with older students participating because of previous schedule conflicts, school transfer, or f ailing grades. These courses were selected because, as required courses, students were representative of the general student population and not particular academic tracts. (…) Participants who met the criteria of African American A ethnicity, were 14 to 18 years old, signed parental consent, signed participant assent, and completed the demographic form were included. |
|  | **Age** 15.3 years, SD 1.1 |
|  | **Sex** 49 males, 57 females |
|  | **Exclusion criteria** Adolescents with a history of peripheral circulation problems or diabetes were excluded because the study protocol included cold water hand immersion. |
| **Protocol** | On the day before testing, participants were instructed not to eat a major meal within 60 minutes before sample collection. Saliva was collected by using a cotton swab and saliva-collecting tube (Salivette, Salimetrics, LLC, State College, Pennsylvania). Participants were instructed to place a cotton swab in the mouth for 2 minutes timed by the research assistant to allow for sufficient saliva volume absorption and then place the swab into the provided collecting tube. Saliva specimens were frozen and stored atj20-C for future assay.  The second salivary sample was collected 20 to 25 minutes after hand removal from the water.  Ethical approval: obtained |
| **Outcomes** | **Primary** What is the prevalence of biologic measures of risk of hypertension specifically FHH, prehypertension, elevated salivary cortisol, hyperresponsive cortisol and CVR |
|  | **Secondary** Is there gender difference in the prevalence of biologic risk factors of hypertension? |
| **Results** | “there was no difference in cortisol levels by gender.”  “Thirty-seven participants (35%) had hyperresponsive cortisol levels. Blood pressure and cortisol reactivity were comparable between men and women.”  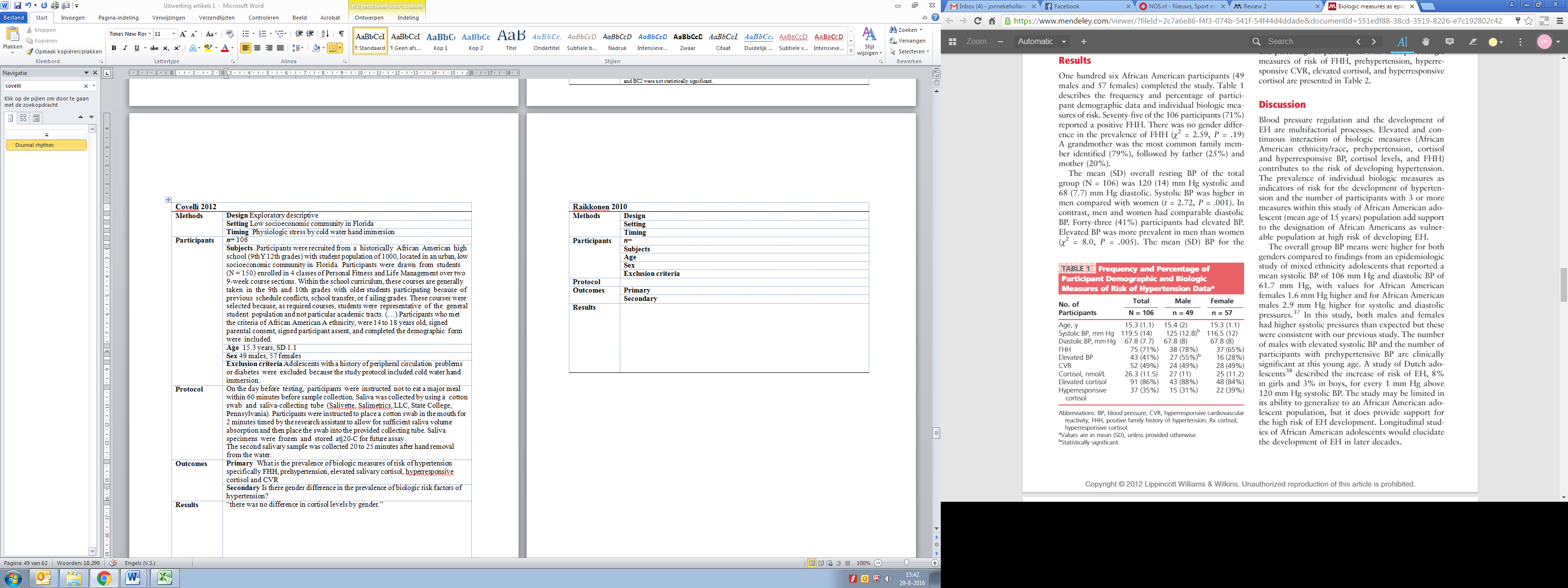 |

| **Methods** | **Design** “large psychobiologic study” – this study seems cross-sectional |
| --- | --- |
|  | **Setting** University of Pittsburgh |
|  | **Timing**  hCRH test |
| **Participants** | ***n=*** 25 |
|  | **Subjects** The children and their families were carefully screened for medical and psychiatric disorders. By design, they were selected to form a group of "supernormals" for the purpose of the psychiatric studies. Families were recruited through printed advertisements, health fairs, direct mailings, and personal contacts. All subjects had a medical history, physical examination, laboratory tests (including electrolytes, liver function, thyroid function, renal function, urinalysis, complete blood count, and ECG), and a comprehensive psychiatric evaluation. In addition, first and second degree relatives of the research subjects were assessed for psychiatric disorders by the family study method using DSM-111-R criteria (17). Over 90% of all first degree relatives were directly interviewed for these assessments. Families were paid for their participation. |
|  | **Age** 6-13 years (mean 10.3, SD 1.6) |
|  | **Sex** 14 boys, 11 girls |
|  | **Exclusion criteria** Exclusion criteria were I) presence or history of psychiatric illness or significant medical disorder; 2) taking any medications (except acetaminophen) within 2 wk of the study; 3) obesity (greater than 150% of ideal body weight); 4) height or weight below the 3rd percentile; and 5) intelligence quotient below 70 or a specific learning disability. |
| **Protocol** | On the second night, baseline nocturnal cortisol sampling (every 20 min) began at bedtime and continued until morning awakening. (…) Subjects spent the remain- der of the day at leisure in the unit, participating in activities such as movies and games with the staff. hCRH (1.0 pg/kg) i.v. infusion over 2 min was given at 1730 h. Subjects had received nothing by mouth from 1400 h through the completion of the hCRH test. A physician and research nurse were in attendance throughout the test. Basal blood samples for ACTH and cortisol were obtained at -30, -15, and 0 min. After hCRH infusion, blood samples were obtained at 15, 30, 60, 90, 120, and 150 min.  Ethical approval: obtained |
| **Outcomes** | **Primary** Although a primary purpose of this study was to provide normative (control) data for similar measures in depressed children, it also created a unique opportunity to examine normal HPA axis physiology in carefully screened, normal children adapted to the testing environment. The effects of age, sex, socioeconomic status, and pubertal status and the interrelationship of physiologic measures of stress are examined |
|  | **Secondary** |
| **Results** | The cortisol peak was greater in boys [472.6 + 129.6 pmol/L (17.2 + 4.7 pg/mL)] than in girls [366.9 k 52.4 pmol/L (1 3.3 + 1.9 pg/mL, p < 0.05)] (Fig. 3).  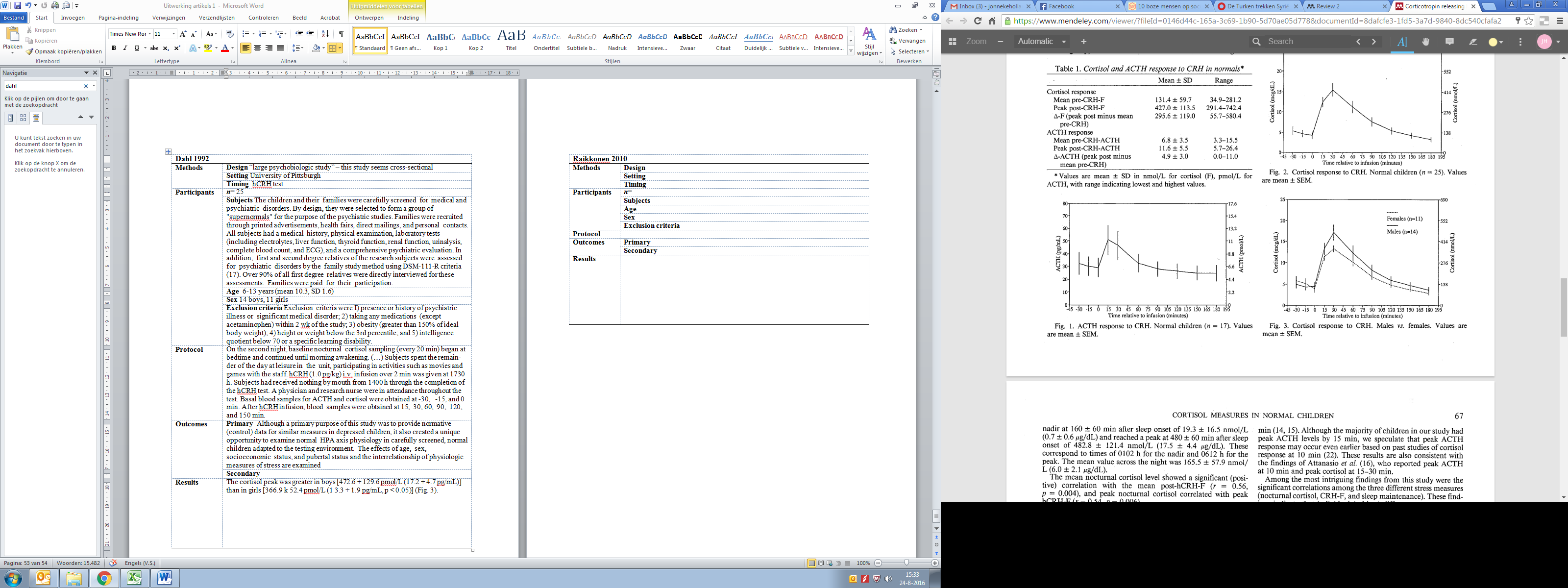 |

### Dahl 1992 [12]

### Daughters 2013 [13]

| **Methods** | **Design** Cross-sectional |
| --- | --- |
|  | **Setting** Not specified |
|  | **Timing**  Automatic Balloon Analogue Risk Task (BART) and Behavioral Indicator of Resiliency to Distress (BIRD) |
| **Participants** | ***n=*** 132 |
|  | **Subjects** Recruited via newspaper advertisements and letters sent to guardians of all high school students in the local county asking for adolescents and their primary caregiver to participate in a study examining the relationship between adolescence and stress. |
|  | **Age** 14-18 years (mean 16.1, SD 1.0) |
|  | **Sex** 73 female, 59 male |
|  | **Exclusion criteria** Eighteen adolescents were excluded from analyses due to either the use of corticosteroids (n=14)or regular smoking in the past 30 days (n=4) which are both known to effect salivary cortisol levels. |
| **Protocol** | Upon arrival to the testing session, guardians and adolescents provided written informed consent and assent, respectively. Experimental sessions took place on weekdays from 3 to 5 pm to control for the effects of circadian variation in cortisol levels. All aspects of the study and the consent form were approved by the University Institutional Review Board. Following informed consent, participants completed the Automatic Balloon Analogue Risk Task (BAR\|T-Auto) to assess baseline levels of risk taking (RT). Afterwards, participants completed a 10 min deep breathing exercise. The first cortisol sample (C1) was collected approximately 10 min after the relaxation exercise. Following the first cortisol collection, participants were exposed to a 15-min psychological stressor, the Behavioral Indicator of Resiliency to Distress (BIRD). A second sample (C2) was collected immediately following stress exposure (i.e., 20 min after C1 collection), and then the BART was readministered to capture stress induced risk taking (RT-stress). Participants then competed a battery of self-report measures while providing two additional saliva samples 9C3 and C4) at 20 min interval.  Ethical approval: obtained |
| **Outcomes** | **Primary** To use an established laboratory-based risk task to evaluate how psychological stress, gender and HPA axis functioning relate to adolescent risk behavior. |
|  | **Secondary** |
| **Results** | “Males had significantly greater baseline cortisol levels. “  “Gender differences in cortisol reactivity to the stress task are also displayed in table 1. Males demonstrated a significantly greater peak cortisol (PC) response to stress. There were no gender differences in total cortisol output (AUCg). Figure 1 displays gender differences in the mean salivary cortisol change from baseline (pre-stress) through the three post-stress assessment time points.”  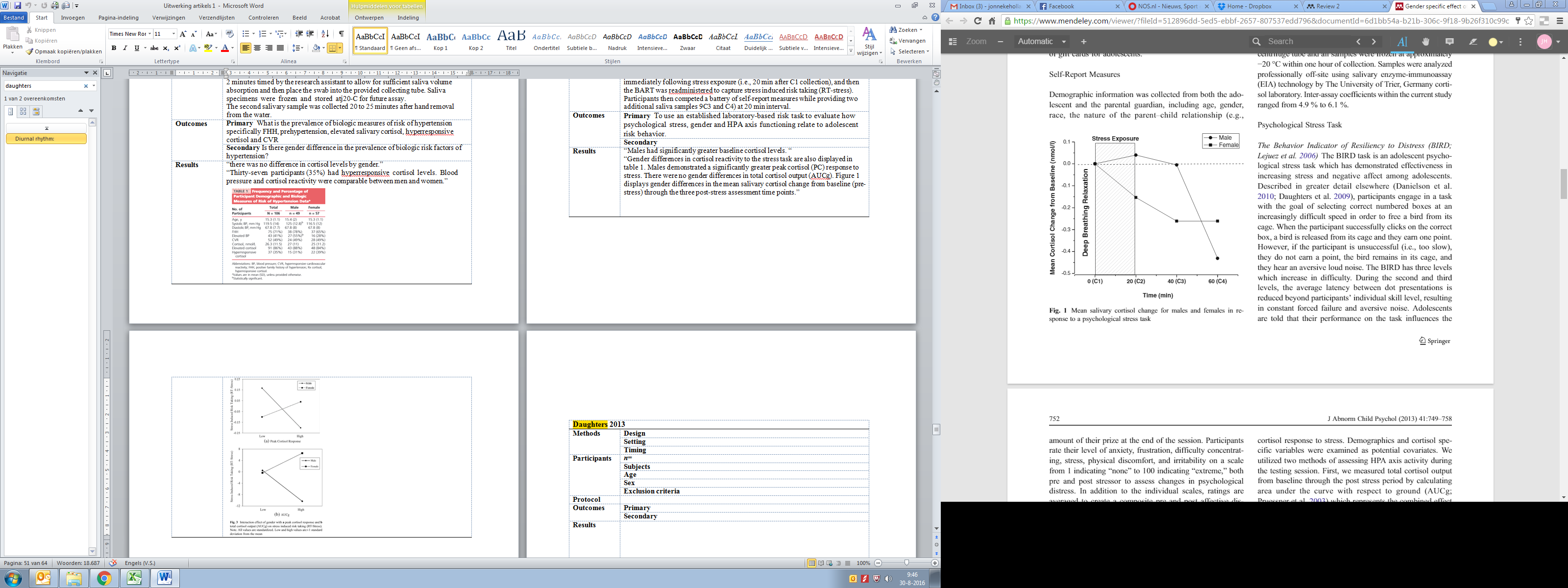  “Post-hoc analysis of the simple slopes as outlined by Aiken & west (1991) indicated that adolescent males demonstrated a significant inverse relation between PC and RT-stress, whereas the relation between PC and RT-stress among female adolescents did not reach significance”  “Post hoc analysis of the simple slopes as outlined by Aiken & West (1991) indicate that adolescent males demonstrated a significant inverse relation between AUCg and RT-stress, whereas the relation between AUCg and RT-stress among female adolescents did not reach significance.”  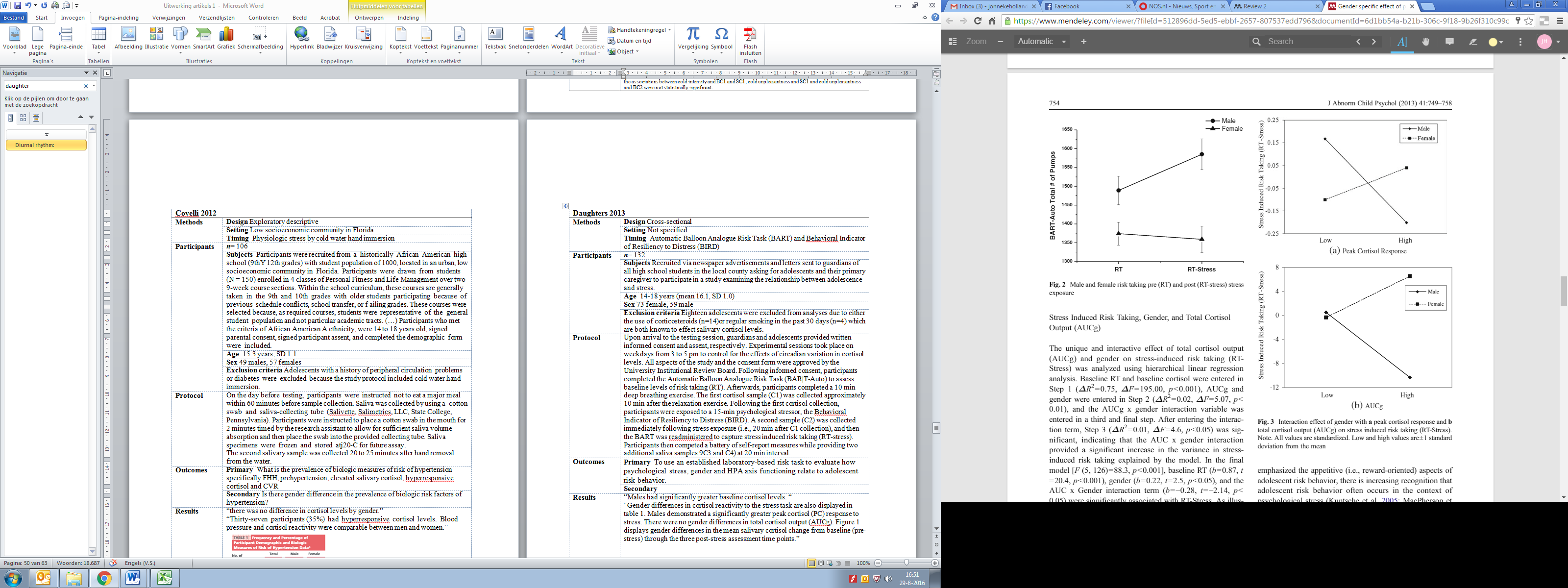 |

### Davis 1995 [14]

| **Methods** | **Design** Cross-sectional |
| --- | --- |
|  | **Setting** Crawford E. Long Hospital, Atlanta, GA, USA |
|  | **Timing**  Serum measurements right after delivery + Neonatal Behavior Assessment Scale |
| **Participants** | ***n=*** 36 |
|  | **Subjects** Subjects were healthy, term (37-42 weeks) infants born at Crawford E. Long Hospital in Atlanta, GA. All subjects underwent at least 90 min of labor (mean in hours = 9.48 +/- 6.69 SD). One male and one female were sesareanction births; the remainder were vaginal deliveries. Two males were vacuum extraction deliveries and two females were low forceps deliveries. All subjects had Apgar scores >8 at 5 min. No subjects were at risk for sepsis or on antibiotics at the time of testing. |
|  | **Age** Age at testing aver-aged 30.99 hours (SD = 8.09, range =19.5-57.75). There was no sex difference in age at testing (hours: M ± SD males = 32.3± 10.1, females = 29.6 ± 5.3), t(34) = 1.01,p > .10. |
|  | **Sex** 18 males and 18 females |
|  | **Exclusion criteria** Not specified other than mentioned under “subjects” |
| **Protocol** | NBAS testing began approximately 10 min after attachment of HR monitoring electrode leads and 5 min after the start of the first saliva sample. Handling and saliva sampling resulted in most infants remaining awake, which precluded administration of habituation items. Consequently, to obtain uniformity, testing began with orientation or motor items for all infants. The remaining 23 individual item scores were summarize d into the seven clusters described by Lester , Als, and Brazelton (1982). Clusters consisted of the following : 1. Orientation: All orientation items plus alertness ; 2. Motor performance: Muscle tonus, motor maturity, pull-to-sit, defensive movements, and activity level ; 3. Range of states: Peak excitement, rapidity of buildup, irritability, and lability of states ; 4. Regulation of states: Cuddliness, consolability, self-quieting activity, hand-to-mouth facility ; 5. Autonomic regulation: Tremulousness, startles, lability of skin color ; 6. Abnormal reflexes: Total number of abnormal reflexes . Three NBAS items (peak of excitement, rapidity of buildup, irritability) were analysed separately. These items have loaded on the factor that reflects arousal or excitability in several different cluster schemes based on factor structure.  After 5 min of baseline HR, the first saliva sample was collected. Five min after the onset of the first saliva sample, the monitor leads were re-moved and the NBAS was administered. Immediately following the last NBAS item, the monitor leads were reattached, the infant's state was recorded, and the second saliva sample was collected. Ten minutes after on-set of collection of the second saliva sample, a third sample was collected, and then the adhesive electrodes were removed. The fourth saliva sample was collected immediately following removal of the electrodes. The last saliva sample was collected 5 min after the fourth saliva sample collection commenced; then the test was ended.  Ethical approval: not specified |
| **Outcomes** | **Primary** The present study hypothesized that sex differences in cardiovascular, adrenocortical, and behavioral activity would exist among neonates following administration of the NBAS, which is a mildly stressful procedure. |
|  | **Secondary** |
| **Results** | “There were no sex differences in baseline salivary cortisol levels M(j,g/dL ± SD males= 1.09 ± 1.32, females = 1.07 ± 1.19),Mann-Whitney U,Z = -.38, p > .10. However, males had significantly higher salivary cortisol levels at each time post-NBAS, Mann-Whitney U: +1 min, Z = - 2.0, p <.05; -F 10 min, Z = -2.31, p < .05; -f-11 min, Z = -3.07, p < .005; -M5 min, Z = -2.69,p < .01 (see Fig. 1). Males also had significantly greater mean changes in cortisol from baseline cortisol than did females (Mfig/dL± SD males = 1.01 ± 1.46, females =-0.07 ± 1.12), ((29) = 2.34, p < .03. Fried-man tests for related samples conducted separately in males and females revealed a non-significant tendency for male cortisol to change with time, xH^ = 13) = 8.88, p =.064, while female cortisol did not vary with time, XHN = 15) = 1.76, p > .10. There were no significant sex differences in umbilical cord blood cortisol (serum cortisol M\i,g/dL ± SD males = 15.57 ± 5.26, females =15.26 ± 7.25), t(33) = .15, p >0.10  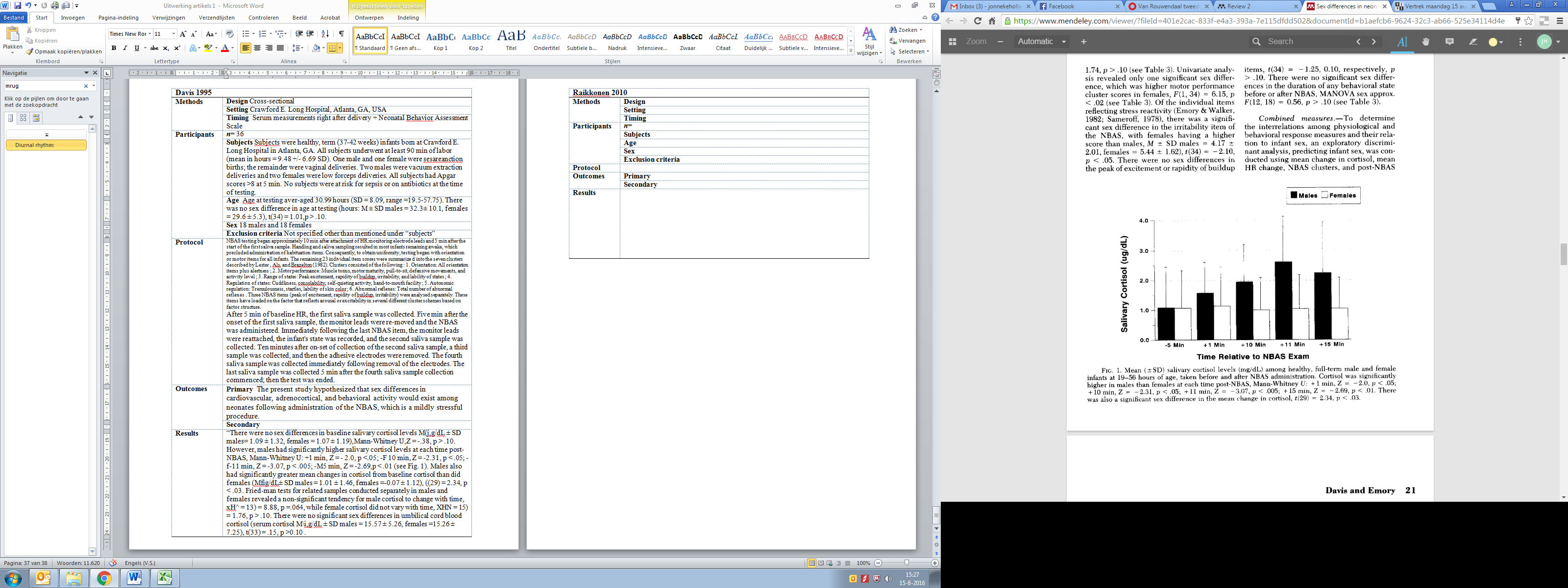 |

| **Methods** | **Design** Cross-sectional |
| --- | --- |
|  | **Setting** Nijmegen and surrounding areas (The Netherlands) |
|  | **Timing**  TSST-C |
| **Participants** | ***n=*** 158 |
|  | **Subjects** Invited through 31 primary schools. “Schools handed out information packages to the children in grades 4 and 5 (age 9—11). Each package contained information about the research project and an application form. Parents of children willing to participate sent in the application form. Inclusion criteria were: birth date between 1 February 1998 and 1 September 2000, and proﬁciency in the Dutch language.” |
|  | **Age** 10.61±0.52 years |
|  | **Sex** 83 girls, 75 boys |
|  | **Exclusion criteria** “Exclusion criteria were: stuttering, a diag-nosis of a developmental disorder, and the use of centrally acting corticosteroid medication.” Additionally: not completing entire data collection protocol. |
| **Protocol** | The TSST-C was used.  Cortisol sampling: “Seven saliva samples were obtained throughout the course of the procedure, at -57, -2, 26, 36, 42, 58, and 80 min from the onset of the stressor.”  Ethical approval: obtained |
| **Outcomes** | **Primary** “to assess the extent to which 10-year-old children had used the emotion regulation strategies reappraisal and sup-pression during their performance on a psychosocial stress task, and related these scores to their cortisol and sAA responses to the task.” |
|  | **Secondary** |
| **Results** | “There was a signiﬁcant main effect of sex, indicating that girls showed a stronger cortisol response than boys.” |

### De Veld 2012 [15]

### De Weerth 2013 [16]

| **Methods** | **Design** Cross-sectional |
| --- | --- |
|  | **Setting** Four regular primary school in the Netherlands, one in the city of Nijmegen and three in nearby villages. |
|  | **Timing**  Experimental protocol: CREST paradigm |
| **Participants** | ***n=*** 42 |
|  | **Subjects** “Parents of children attending their second year of school were approached by letter (N ¼ 179). In this letter, the study was described, and parents were invited to enroll their child if he/she wished to participate.” |
|  | **Age** 68.0±4.3 months |
|  | **Sex** 20 boys, 22 girls |
|  | **Exclusion criteria** Clinically referred diagnosis and/or daily use of medication affecting cortisol secretion. One child was excluded due to earache on the testing day. |
| **Protocol** | Carried out in a research van parked outside the school. “The testing took place in the afternoon, starting between 13:15 and 15:30 h, in order to avoid the circadian morning peaks of cortisol secretion”  “The paradigm had a duration of 20 min, and consisted of three tasks (15 min), followed by a period of stress from an anticipated evaluation (5 min).” “The children were tested by two researchers: an experimenter and a judge.”  Child presented with four presents: chooses one, and is then told to perform some task in front of the judge, after which the judge would decide which present the child deserves.  First task: stand still and not move for 60 seconds, an alarm would go off if they moved. No matter how little the child moved, the alarm would go off twice (at 20 and 40seconds)  Second task: The child listened to a story (3 minutes) about animals, and every time an animal was mentioned (8 times), the child was asked to imitate the sound made by the animal. Red or green cards were shown for wrong/right. Irrespective of performance, the green card was only shown 3 times out of 8 animals.  Third task: the child was asked to make a tower of empty soft drink cans identical to the one shown by the experimenter. The experimenter then uncovered an example tower which was invisibly glued, and consisted of a pyramid of cans on their side [four, three, two, and one can(s) in each layer]. The judge told the child that the task was very easy for children to perform, and should therefore work out ﬁne. In reality, when the child tried to build the tower, the cans kept on rolling away making the task impossible. After 3 min, the judge instructed the child that he/she had to stop building the tower.  Cortisol samples: 6 in total: “Two samples measured baseline concentrations: one was taken just before the stress test (C1; pre-stress) and one 15 min after starting the stress test (C2; pre-response; after completion of the three tasks, but before the period of stress due to an anticipated evaluation. (…) Two samples were obtained 25 (C3) and 35 (C4) min after the beginning of the stress test. Lastly, two samples obtained at 45 and 60 min after the beginning of the stress test were used as recovery measurements (C5 and C6, respectively)”  Ethical approval: obtained |
| **Outcomes** | **Primary** “to develop a paradigm that would elicit cortisol elevations in 5- and 6-year-olds in the group as a whole.” |
|  | **Secondary** |
| **Results** | “Correlations between the actual increase in cortisol (highest peak concentration minus lowest baseline concentration) and the possible confounders child’s sex and age, parental educational level, time of testing, sex of experimenter, and sex of judge were non-signiﬁcant (Table II).”  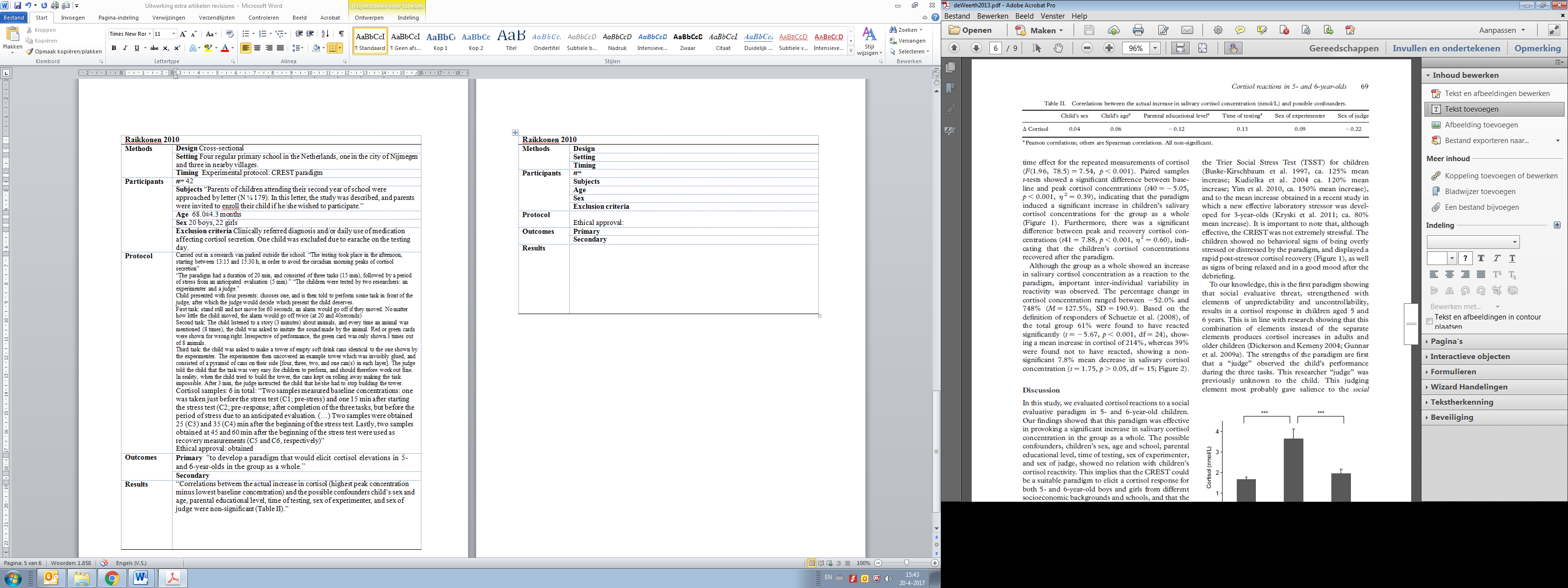 |

### Dietrich 2013 [17]

| **Methods** | **Design** Longitudinal / cohort study (only baseline was used in this study) |
| --- | --- |
|  | **Setting** TRAILS study (not specified in article, but from other articles: northern Netherlands) |
|  | **Timing**  CAR |
| **Participants** | ***n=*** 1604 (population cohort) and 357 (clinic-referred cohort) |
|  | **Subjects**  Population based, also: the clinic-referred cohort runs parallel to the population cohort and started about two years later. Inclusion in the clinic-referred cohort was based on referral to the Groningen university child psychiatric outpatient clinic. |
|  | **Age** Baseline: mean age 11.1 years, SD 0.55 (population sample) |
|  | **Sex** 50.8% girls (population sample) |
|  | **Exclusion criteria** Subjects were excluded from the analyses due to the use of corticosteroid-containing medication (n=22), lack of compliance with the protocol (n=9), and extreme cortisol values >3sd from the mean, n=32). |
| **Protocol** | Participants received a verbal and written instruction to collect slaiva at home immediately after waking up as they were still lying in bed (Cort1) and 30 min later (Cort2), using the Sarstedt Salivette Devices. Saliva was sampled on a single day.  Ethical approval: obtained |
| **Outcomes** | **Primary** we tested hypotheses on the association between single day cortisol (basal morning levels and CAR) and specifically constructed dimensions of anxiety (cognitive versus somatic), depressive (cognitive-affective versus somatic), and externalizing problems (reactive versus proactive aggression), and explored the modifying role of sex. |
|  | **Secondary** |
| **Results** | Cotisol 1 and 2, as well as AUCg (total morning cortisol): significantly higher in girls. CAR (AUCi): not significantly different, but higher in girls.  (data only from population cohort, not referred cohort)  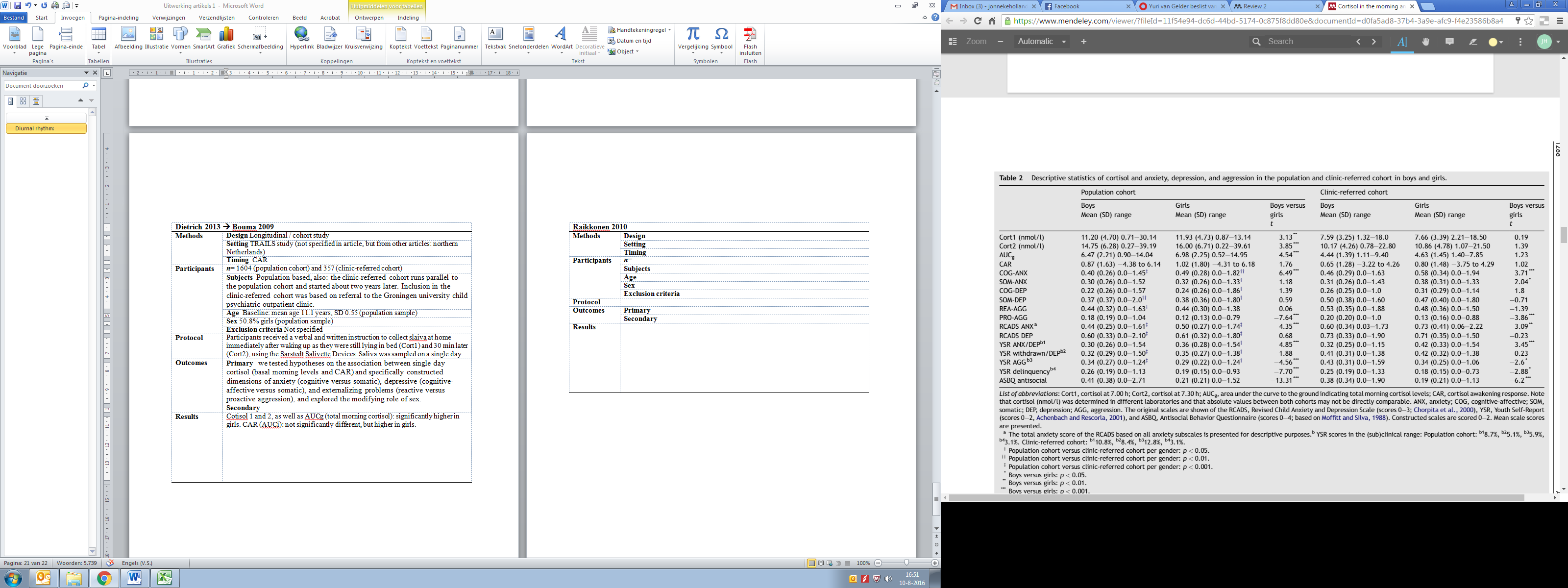 |

| **Methods** | **Design** Longitudinal study (only first wave of assessment used) |
| --- | --- |
|  | **Setting** Not specified |
|  | **Timing**  TSST-C |
| **Participants** | ***n=*** 111 |
|  | **Subjects** Participants were healthy children and adolescents and a parent or caregiver who are participating in a longitudinal study of puberty and behavior.  All participants were free from chronic health problems, and were not using any medications known to interfere with hormone levels (e.g., oral steroids) or influence weight gain (e.g., selective serotonin uptake inhibitors). |
|  | **Age** Girls were aged 8, 10, or 12 years (mean 10.49, SD 1.5) and boys are aged 9, 11, or 13 years (mean 11.44, SD 1.6) |
|  | **Sex** 55 girls, 56 boys |
|  | **Exclusion criteria** “89 were ineligible on the basis of study criteria” not specified which |
| **Protocol** | A total of five saliva samples were collected (Sample 1 at 0 minutes, Sample 2 at 20 minutes, Sample 3 immediately post-TSST, Sample 4 at 10 minutes post-TSST, and Sample 5 at 20 minutes post-TSST)  Ethical approval: obtained |
| **Outcomes** | **Primary** To examine the associations between symptoms of depression, cortisol reactivity and BMI in a cross-sectional study |
|  | **Secondary** |
| **Results** | There were no significant sex differences in measures of depression, phys-ical activity, cortisol reactivity, or BMI.  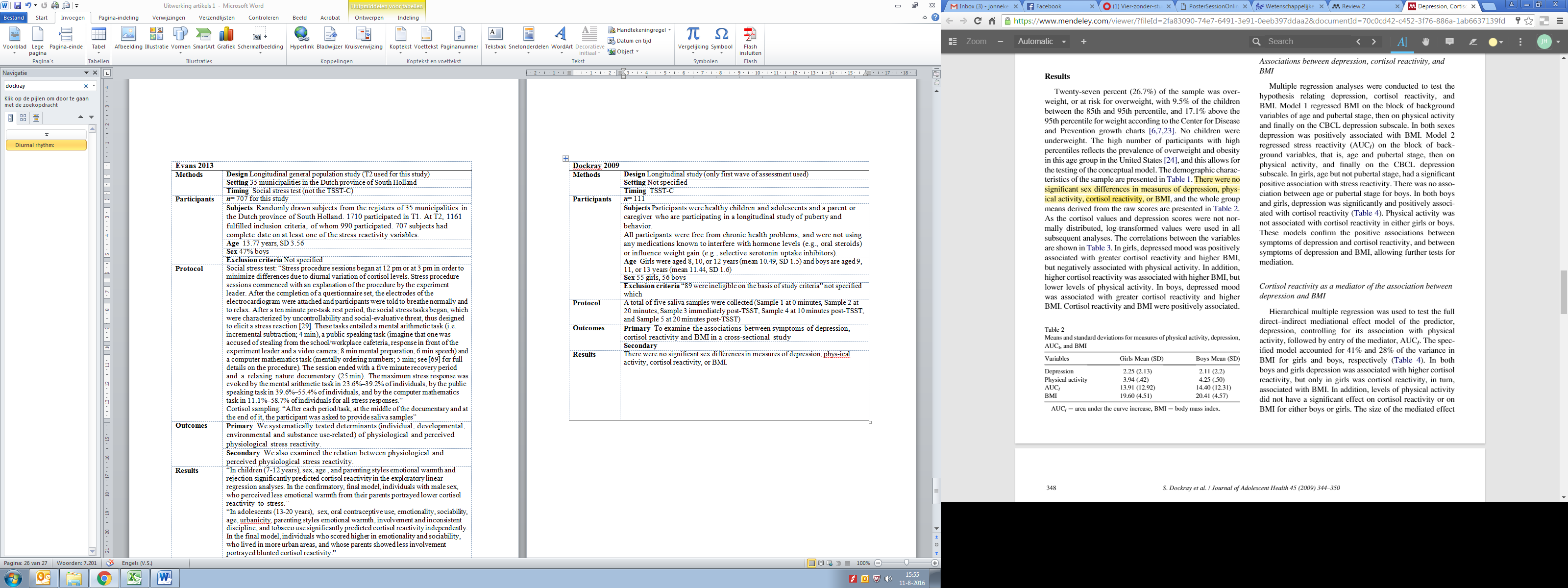  “In both boys and girls depression was associated with higher cortisol reactivity, but only in girls was cortisol reactivity, in turn, associated with BMI.”  “In summary, for girls, the effect of depressed mood on higher BMI is mediated and accentuated by higher cortisol responses to a stressor. However, in boys, depressed mood is directly associated with BMI and cortisol reactivity, but cortisol reactivity is not, in turn, associated with BMI, and so therefore does not mediate the direct effects of depressed mood” |

### Dockray 2009 [18]

### Doom 2013 [19]

| **Methods** | **Design** cross-sectional study |
| --- | --- |
|  | **Setting** Minnesota, USA |
|  | **Timing** Diurnal rhythm |
| **Participants** | ***n=*** 110 controls |
|  | **Subjects** Nonmaltreated racially and ethnically diverse children who attended a summer research day camp for low-income children. Nonmaltreating families were recruited from families that received Temporary Assistance to Needy Families in order to make comparisons with SES held constant. |
|  | **Age** 9.42 ± 0.88 yrs |
|  | **Sex** In the entire group (including maltreated children) 52% were males |
|  | **Exclusion criteria** “Families were excluded from the nonmaltreatment group if they received preventative DHS services due to concerns about maltreatment. Qualified research assistants interviewed the mothers of children recruited for the nonmaltreatment group using the Maternal Child Maltreatment Interview (Cicchetti et al., 2003) to inquire about any maltreatment the child may have experienced that was not reported to DHS. To assure that all information had been evaluated, research assistants also examined DHS records in the year after camp participation.” |
| **Protocol** | “Saliva was collected 3 times across the day for 5 days for cortisol and dehydroepiandosterone (DHEA) analysis. Children chewed Trident sugarless original flavor gum to stimulate saliva flow and passively drooled through a short straw into a 20-ml plastic vial.”  Cortisol samples: “Children provided saliva samples at the same time for each of the 5 days of the camp week. Samples were collected when the child arrived at camp at 0900 h, before lunch at 1200 h, and before they departed at 1600 h. “  Ethical approval: obtained |
| **Outcomes** | **Primary** the influence on neuroendocrine profiles in children by the interaction between gender and the stress of maltreatment |
|  | **Secondary** N/A |
| **Results** | “There were no gender differences in cortisol levels in the nonmaltreated group and the group with less pervasive maltreatment, F(1, 101) = .20, p > .05, and F(1, 46) = 1.96, p > .05, respectively.” (see figure 1)  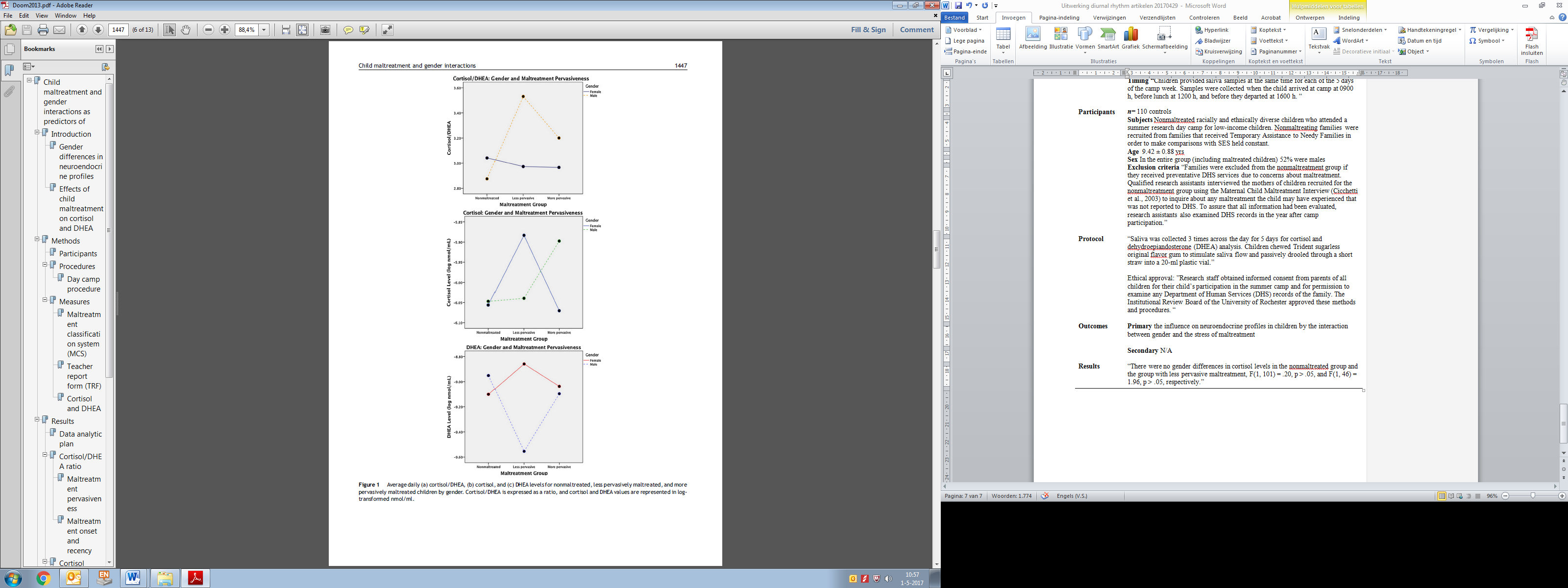 |

### Dorn 1996 [20]

| **Methods** | **Design** Cross-sectional |
| --- | --- |
|  | **Setting** Not specified |
|  | **Timing**  oCRH stimulation test |
| **Participants** | ***n=*** 20 control subjects, 21 depressed adolescents |
|  | **Subjects** The subjects were depressed adolescents and controls matched by age, gender, race, socioeconomic status and pubertal stage. (…) Eligibility criteria for the adolescents included the following: 1) age 10 through 16 years, 2) taking no medication, 3) experiencing no chronic illness that would interfere with psychological or endocrine testing, 4) not pregnant and 5) spoke English as the primary language. |
|  | **Age** Controls: 15.1, SD 1.0 |
|  | **Sex** Controls: 11 girls, 9 boys |
|  | **Exclusion criteria** Adolescents could neither have schizoid symptoms nor could they meet criteria for an eating disorder or be current drug or alcohol abusers, as these disorders are known to influence the HPA axis hormones. |
| **Protocol** | Visit 3.The adolescent returned on another day to complete an evening oCRH stimulation test in a quiet environment. The adolescent fasted after 2P.M. (except for water) and refrained from smoking. At 4P.M. the adolescent arrived at the clinic for a physical examination which included Tanner staging (Marshall and Tanner, 1969, 1970) for pubertal development. At 6P.M. an intravenous line (IV) was inserted and kept open with normal saline. Blood was drawn for the determination of ACTH and cortisol concentration at -120(6P.M.),-60,-45, -30, -15,0,+5, +15, +30, +60, +90, and +120 minutes. At 8P.M. (0minutes), a bolus of oCRH (1ug/kg) was given. The IV was removed at 10P.M. Paper-and-pencil psychological tests were administered throughout the evening. Adolescents were permitted to have a parent/guardian in attendance. They were permitted only to read, do homework, or listen to a set of selected music tapes using earphones  Ethical approval: obtained |
| **Outcomes** | **Primary** To determine whether there were group and gender differences in measures of HPA axi activity in depressed and nondepressed adolescents. |
|  | **Secondary** |
| **Results** | For cortisol, there were no significant gender or group differences, group by gender interactions, or time differences for mean baseline cortisol concentration. For peak cortisol concentration, there were no significant gender or group differences, or group by gender interactions.  There were no significant gender or group differences, or group by gender interactions, for cortisol AUC or NET.  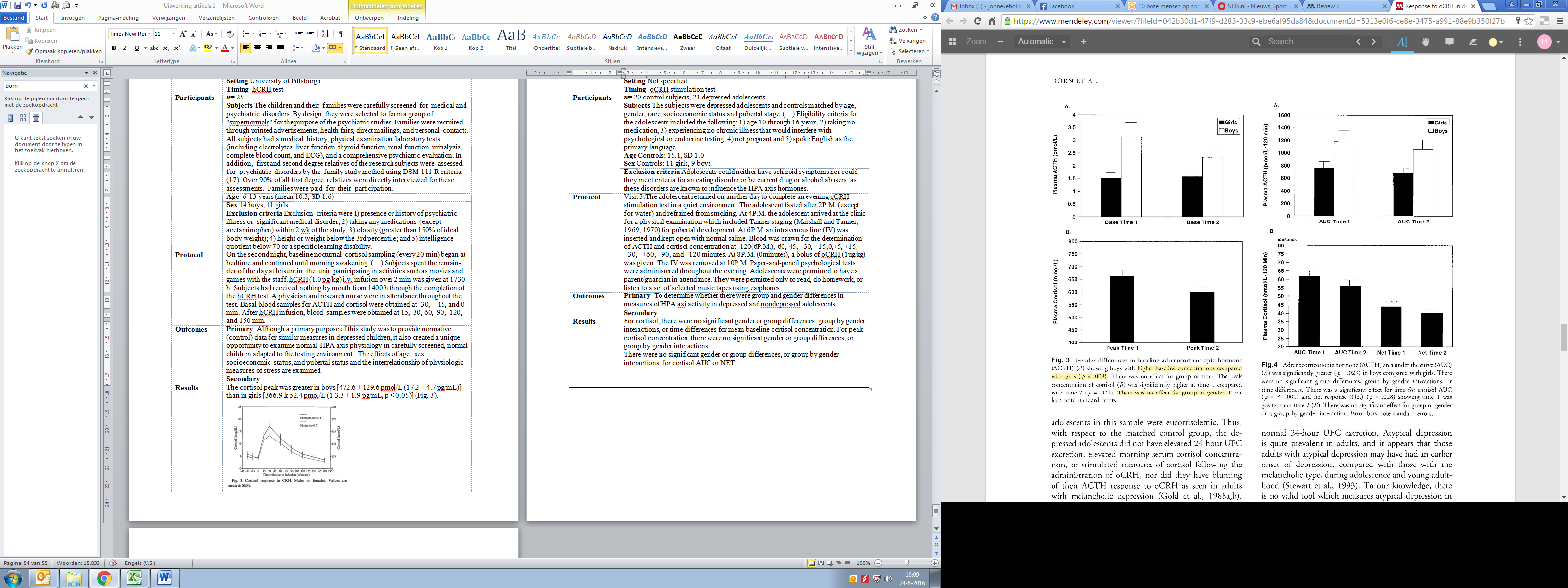 |

### Eiden 2015 [21]

| **Methods** | **Design** Longitudinal |
| --- | --- |
|  | **Setting** “large urban hospital” |
|  | **Timing**  Laboratory Temperament Assessment Battery (LabTAB) |
| **Participants** | ***n=*** 217 (69 non-tobacco-exposed infants) |
|  | **Subjects**  Women who presented for care at a large urban hospital’s prenatal clinic were asked to complete a screening form during their first prenatal appointment. Eligible women were invited to participate in an ongoing longitudinal study of maternal health and child development. Initial exclusionary criteria included: less than 20 weeks gestation, maternal age of less than 18 years, and multiple fetuses. Additional eligibility criteria were: no illicit drug use (other than cannabis based on maternal self-reports, salivary assays in each trimester, and infant meconium assays), no heavy alcohol use (more than 1 drink/day on average or 4 drinks on one occasion based on maternal self-reports on a calendar based interview) after pregnancy recognition, and no heavy marijuana use (more than 1 marijuana joint/day on average based on maternal self-reports) after pregnancy recognition (see below for measurement details). Women who agreed to participate were scheduled for four appointments: one at the end of each trimester of pregnancy and one at 2 months postpartum (at age corrected for prematurity). A second postpartum assessment was scheduled for 9 months postpartum (at age corrected for prematurity). At the end of each month of recruitment, the closest matching non-smoker (based upon age and education) was invited to participate. Smokers were over-sampled so that one non-smoker was recruited for every two smokers (taking the average of age and education of both) |
|  | **Age** 9 months |
|  | **Sex** not specified |
|  | **Exclusion criteria** See “subjects” |
| **Protocol** | At 9 months, infant reactivity and regulation were assessed during a positive and negative affect paradigm taken from the Laboratory Temperament Assessment Battery (LabTAB; Goldsmith & Rothbart, 1996 ). These included the puppet show designed to elicit positive affect and an arm restraint paradigm to elicit anger/frustration. The order of procedures was as follows: The Time 1 or pretask saliva sample (T1) was collected after the infant arrived at the laboratory; the infant was then seated in a high chair, hooked up to electrodes for measurement of heart rate, and watched a Baby Einstein video for 3 minutes for a baseline assessment of physiology (see Calkins, 1997 ) followed by a 6 minute focused attention paradigm (presentation of 4 novel toys for 90 seconds each), a 2-minute puppet show, and a second 3-minute baseline interval (watching a video). This was followed by the arm restraint paradigm designed to elicit anger/frustration. The Time 2 (T2) saliva sample was collected at the end of the arm restraint paradigm. This was followed by another 3 minutes of video. The infant was then unhooked from the heart rate monitor and placed on a play mat on the floor with a variety of toys for measurement of infant activity level. Mothers were asked to respond to their infants as they normally would but not to initiate interaction. This was followed by an 8-minute free play procedure and another 3 minutes of the infant interacting with toys in a basket. The third saliva sample was collected 20 minutes after the end of arm restraint (T3) and the fourth sample was collected 40 minutes after the end of arm restraint (T4)  Ethical approval: obtained |
| **Outcomes** | **Primary** We examined the role of postnatal tobacco exposure as a significant additional predictor of stress reactivity |
|  | **Secondary** |
| **Results** | Repeated measures ANOVA was used to examine sex-related differences in cortisol over time. Results indicated a significant sex by time interaction, F(3, 512.6) = 3.11, p = .035, with a significant cubic trend (see Figure 1). Simple effects analyses indicated that boys had significantly higher cortisol values compared to girls at Times 2 and 4, but not at Times 1 and 3. There was no significant effect of time on cortisol for girls, but a significant increase in cortisol from Time 1 to 2 and a significant decline between Time 2 and 3 for boys  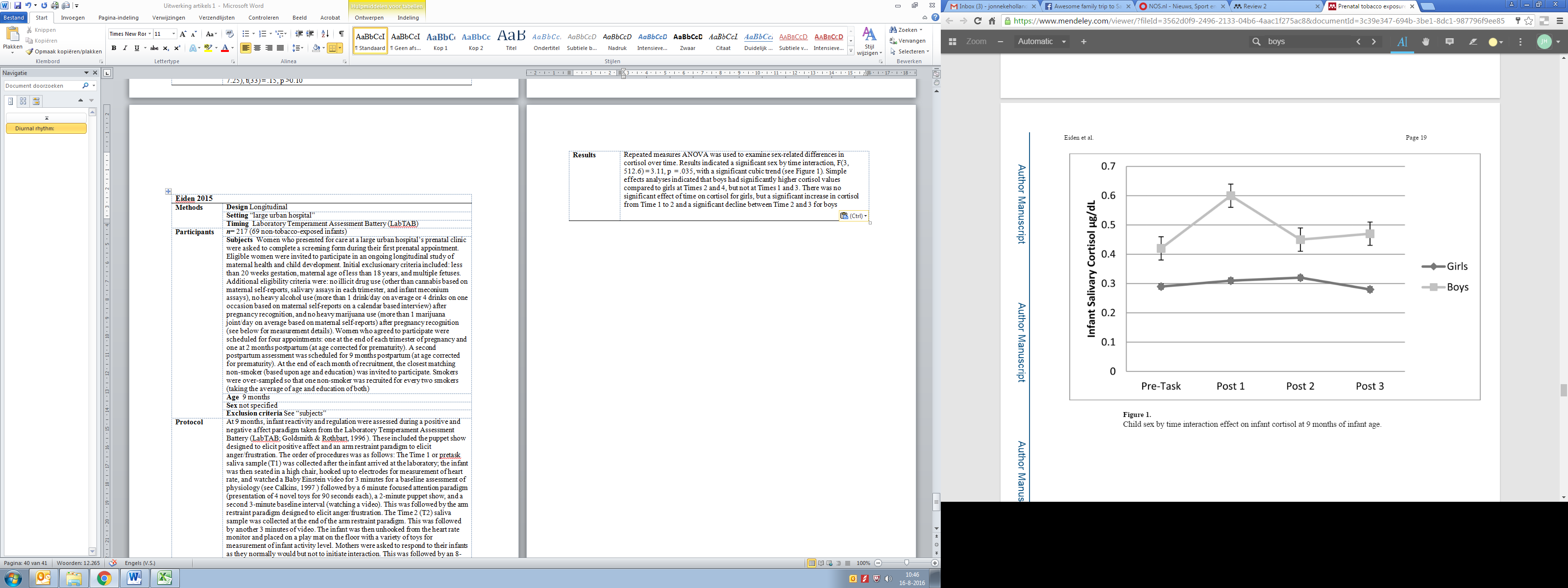 |

### Evans 2013 [22]

| **Methods** | **Design** Longitudinal general population study (T2 used for this study) |
| --- | --- |
|  | **Setting** 35 municipalities in the Dutch province of South Holland |
|  | **Timing**  Social stress test (not the TSST-C) |
| **Participants** | ***n=*** 707 for this study |
|  | **Subjects**  Randomly drawn subjects from the registers of 35 municipalities in the Dutch province of South Holland. 1710 participated in T1. At T2, 1161 fulfilled inclusion criteria, of whom 990 participated. 707 subjects had complete data on at least one of the stress reactivity variables. |
|  | **Age** 13.77 years, SD 3.56 |
|  | **Sex** 47% boys |
|  | **Exclusion criteria** Not specified |
| **Protocol** | Social stress test: “Stress procedure sessions began at 12 pm or at 3 pm in order to minimize differences due to diurnal variation of cortisol levels. Stress procedure sessions commenced with an explanation of the procedure by the experiment leader. After the completion of a questionnaire set, the electrodes of the electrocardiogram were attached and participants were told to breathe normally and to relax. After a ten minute pre-task rest period, the social stress tasks began, which were characterized by uncontrollability and social-evaluative threat, thus designed to elicit a stress reaction [29]. These tasks entailed a mental arithmetic task (i.e. incremental subtraction; 4 min), a public speaking task (imagine that one was accused of stealing from the school/workplace cafeteria, response in front of the experiment leader and a video camera; 8 min mental preparation, 6 min speech) and a computer mathematics task (mentally ordering numbers; 5 min; see [69] for full details on the procedure). The session ended with a five minute recovery period and a relaxing nature documentary (25 min). The maximum stress response was evoked by the mental arithmetic task in 23.6%–39.2% of individuals, by the public speaking task in 39.6%–55.4% of individuals, and by the computer mathematics task in 11.1%–58.7% of individuals for all stress responses.”  Cortisol sampling: “After each period/task, at the middle of the documentary and at the end of it, the participant was asked to provide saliva samples”  Ethical approval: obtained |
| **Outcomes** | **Primary** We systematically tested determinants (individual, developmental, environmental and substance use-related) of physiological and perceived physiological stress reactivity. |
|  | **Secondary** We also examined the relation between physiological and perceived physiological stress reactivity. |
| **Results** | “In children (7-12 years), sex, age , and parenting styles emotional warmth and rejection significantly predicted cortisol reactivity in the exploratory linear regression analyses. In the confirmatory, final model, individuals with male sex, who perceived less emotional warmth from their parents portrayed lower cortisol reactivity to stress.”  “In adolescents (13-20 years), sex, oral contraceptive use, emotionality, sociability, age, urbanicity, parenting styles emotional warmth, involvement and inconsistent discipline, and tobacco use significantly predicted cortisol reactivity independently. In the final model, individuals who scored higher in emotionality and sociability, who lived in more urban areas, and whose parents showed less involvement portrayed blunted cortisol reactivity.”  “In sum, cortisol reactivity was determined by sex and perceived parental emotional warmth in children and emotionality, sociability, urbanicity and parental involvement in adolescents.”  E-mail response from author:  “sex was coded 0=male, 1=female, so a positive beta indicated that males showed lower cortisol reactivity in children.  In adolescents, sex was only significant in the preliminary model, in the opposite direction: girls showed lower cortisol reactivity.” |

### Forest 1978 [23]

| **Methods** | **Design** Cross-sectional |
| --- | --- |
|  | **Setting** Not specified |
|  | **Timing**  ACTH test |
| **Participants** | ***n=*** 20 infants and 35 prepubertal children |
|  | **Subjects**  All infants and children studies were investigated to rule out pituitary or adrenal dysfunction; all had normal levels of 17-ketosteroids and ketogenic steroids, plasma ACTH and corticoids. |
|  | **Age** Infants: range: 5-365 days old; children: range: 1-12.6 years |
|  | **Sex** Infants: 17 boys, 3 girls; children: 21 boys, 14 girls |
|  | **Exclusion criteria** Not specified |
| **Protocol** | Synthetic Zn β1-24 ACTH (500ug/m2 body surface area; tetracosactrin-depot, Ciba) was administered IM at 0800 and 2000h for 3 days. Blood was collected from a peripheral vein before the first ACTH injection and the day after the last injection between 0800-0900h.  Ethical approval: “All infants and children studies were investigated to rule out pituitary or adrenal dysfunction; all had normal levels of 17-ketosteroids or ketogenic steroids, plasma ACTH and corticoids” We therefore assume that this data was obtained through standard care, after which it was used for this study. |
| **Outcomes** | **Primary** To establish whether the suppressive effect of ACTH on plasma T levels also occurs in male infants. |
|  | **Secondary** To document whether the stimulatory effect of ACTH on T levels, described in prepubertal female children, varies with advancing age. |
| **Results** | Infants:  In response to ACTH, significant (P<0.0001) increases in Δ4 and F levels were observed in both sexes, whereas the response of T varied with sex and age. “ AND “basal and post-ACTH levels of F were lower (P<0.02) in group B (male infants without testicular T production and girls) than in group A infants (males with testicular function) but were not significantly lower for Δ4. The difference seemed to be age- but not sex-related; when comparing infants of boys sexes between 1-4 months of age with those 4-12 months old, post-ACTH values for Δ4 and F were significantly lower (P<0.01) in the older age group.”  Children: “responses to T, Δ4 and F to ACTH stimulation were similar in prepubertal boys and girls” |

| **Methods** | **Design** Case-control / cross-sectional |
| --- | --- |
|  | **Setting** Stockholm, Sweden |
|  | **Timing**  Diurnal rhythm + CAR |
| **Participants** | ***n=*** 157 |
|  | **Subjects** Community based sample, divided in intact families and subjects that live in two homes |
|  | **Age** 14-16 years |
|  | **Sex** Intact families: 89 girls and 43 boys, two homes: 17 girls and 8 boys |
|  | **Exclusion criteria** Only living with one parent, chronic illnesses or taking medication possibly interfering with HPA-axis, taking saliva sample >5 min after waking up. |
| **Protocol** | Saliva samples were collected within two weeks after completing the initial questionnaire, at four points in time during an ordinary school day: 1) immediately at waking up, 2) at 30 minutes post-awakening, 3) at 60 minutes post-awakening, and 4) at 8 p.m.  Ethical approval: obtained |
| **Outcomes** | **Primary** To investigate the associations between living arrangements and salivary cortisol in mid-adolescent boys and girls |
|  | **Secondary** The associations between living arrangements and subjective health in terms of recurrent pain. |
| **Results** | Girls in intact families: at waking: 2.39 (SD = .70); +30 min.: 3.10 (SD = .53); +60 min.: 2.90 (SD = .65); evening: .38 (SD = .82); Girls in two homes: at waking; 2.32 (SD = .72); +30 min.: 2.99 (SD = .44); +60 min.: 2.80 (SD = .47); even-ing: .50 (SD = 1.00); Boys in intact families: at waking: 1.88 (SD = .71); +30 min.: 2.77 (SD = .57); +60 min.: 2.74 (SD = .46); evening: .31 (SD = .81); Boys in two homes: at waking: 2.24 (SD = .71); +30 min.: 2.79 (SD = .43); +60 min.: 2.74 (SD = .44); evening: .43 (SD = 1.53)  For cortisol during the first hour of awakening (CAR), sex emerged as a significant predictor across both steps with girls exhibiting a higher cortisol awakening response. Similarly, girls had a greater diurnal decline value. |

### Fransson 2014 [24]

### Frias 2000 [25]

| **Methods** | **Design** Cross-sectional |
| --- | --- |
|  | **Setting** Not specified, presumably Granada, Spain |
|  | **Timing**  Cortisol levels after acute alcohol intoxication |
| **Participants** | ***n=*** 48 (21 controls) |
|  | **Subjects** Young adolescents with AAI. They arrived at the emergency department with evident behavioural symptoms of drunkenness. Controls without alcohol consumption were also studied. |
|  | **Age** 13-17 years |
|  | **Sex** 21 males, 27 females (controls: 10 males and 11 females) |
|  | **Exclusion criteria** Endocrine disorders |
| **Protocol** | Blood samples were drawn from each subject (AAI and C) from 12.00 to 3.00 am.  Ethical approval: “carried out in accordance with the Helsinki Declaration” |
| **Outcomes** | **Primary** the effects of AAI on PG axis hormones and the possible contribution of BEND, PRL and PA axis hormones to the alcohol-induced dysfunction of PG axis hormones |
|  | **Secondary** |
| **Results** | Serum ACTH and F levels increased significantly in AAI adolescents. The responses to ACTH and F to AAI were higher in females than in males. ACTH and F increased 10 and 1.6-fold respectively in females, versus 5.9 and 1.4 fold in males. The rates of ACTH and F increase were 914% and 58.5% respectively in females and 499% and 46% respectively in males.  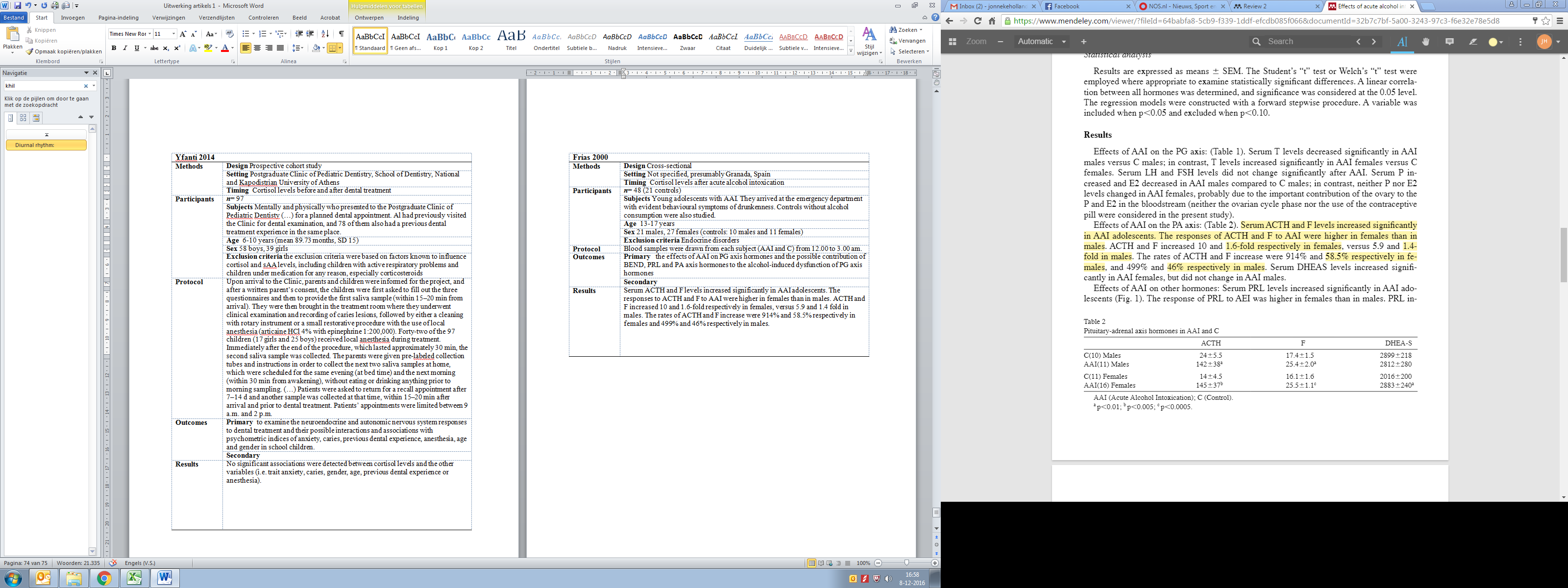 |

| **Methods** | **Design** Case-control |
| --- | --- |
|  | **Setting** Hospital Materno-Infantil, La Coruña, Spain |
|  | **Timing**  Diurnal rhythm |
| **Participants** | ***n=*** 76 (55 short stature, 21 standard stature) |
|  | **Subjects**  Children with short and normal stature |
|  | **Age** 11.2 +/- 0.37 years |
|  | **Sex** Short stature: 42 boys, 13 girls; normal stature: 11 boys and 10 girls |
|  | **Exclusion criteria** Not specified, “all subjects were in apparent good health at the time of the study” |
| **Protocol** | Subjects were living on a diurnal waking (7:30-22:30), nocturnal resting routine, consuming the usual hospital diet. Blood was drawn at about 3-hr intervals during a 24-hr sampling span, and serum stored frozen at 60C until radioimmunoassay for CT concentration (in ug/dl).  Ethical approval: not specified |
| **Outcomes** | **Primary** To study possible differences in rhythmic characteristics of CT secretion with stature |
|  | **Secondary** To establish reference standard for circadian CT parameters |
| **Results** | “No gender difference for standard children (P=0.361, 0.169, 0.189)  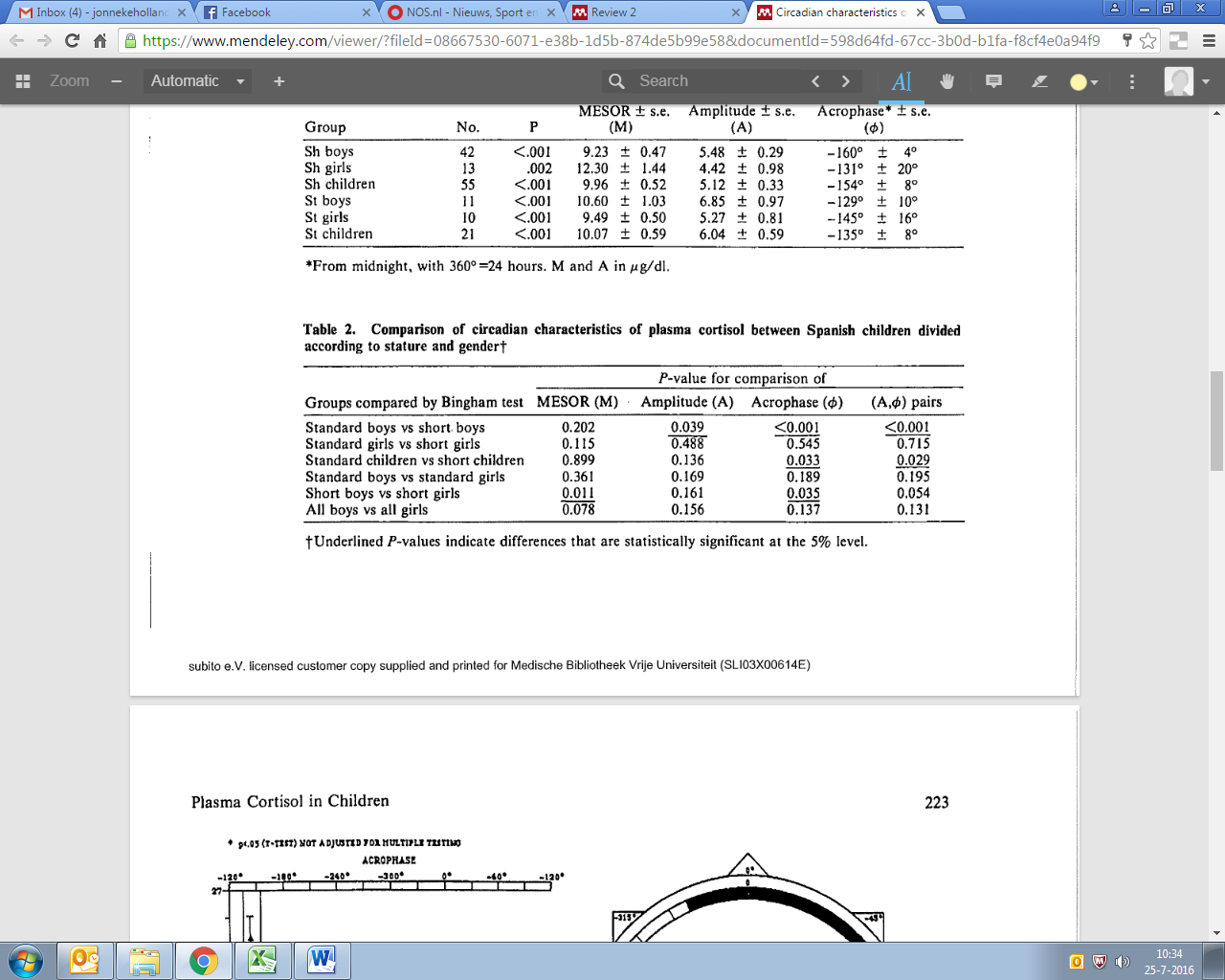 |

### Garcia 1990 [26]

### Gecgelen 2012 [27]

| **Methods** | **Design** Longitudinal study |
| --- | --- |
|  | **Setting** Department of Orthodontics of Suleyman Demirel University, Turkey |
|  | **Timing**  Cortisol levels before and after rapid maxillary expansion |
| **Participants** | ***n=*** 40 |
|  | **Subjects** Children with either a functional unilateral or bilateral posterior crossbite with transverse deficiency |
|  | **Age** 10.9-14.7 years |
|  | **Sex** 20 girls, 20 boys |
|  | **Exclusion criteria** Craniofacial anomalies, usage of medications that could interfere with cortisol levels |
| **Protocol** | Saliva samples were collected from each patients 15 min prior to and 10, 30, 60 min after activation of expansion appliance which took place at 09:15, 09:40, 10:00 and 10:30 (AM). The patients were asked to give saliva samples at 9 days: a week before RME treatment (T0), at the day of the expansion appliance was bonded (T1), at the days of 1st, 4th, 7th, 14th, 25th, 36th activations of expansion screw (T2, T3, T4, T5, T6, T7) and after the retention period of 3 months (T8).  Ethical approval: obtained |
| **Outcomes** | **Primary** to use salivary cortisol levels, pressure pain threshold (PPT) and Spielberger’s State–Trait Anxiety Inventory for Children (STAIC) to assess stress, anxiety and pain during the expansion and retention phase of RME in children and investigate whether this parameters are associated with gender or skeletal maturity stages |
|  | **Secondary** |
| **Results** | “The mean salivary cortisol values were similar with respect to gender (P>0.05).” |

| **Methods** | **Design** Cohort study |
| --- | --- |
|  | **Setting** British Columbia, Canada |
|  | **Timing**  Cortisol response after vaccination |
| **Participants** | ***n=*** 32 full-term |
|  | **Subjects**  Full-term infants were recruited by advertisement |
|  | **Age** 4.2 months (SD 1.0) |
|  | **Sex** 20 boys, 12 girls |
|  | **Exclusion criteria** Received other than 3 immunization injections, or were fed within 30 minutes of study |
| **Protocol** | Saliva was collected 3 times: Basal (before vaccination), Reactivity (20 min after first Injection), Recovery (30 min after first Injection).  Three intramuscular injections of 0.5ml volume were administered in alternate thighs using 25 gauge needles: Injection 1: Diphteria, Tetanus, acellular Persussis, inactivated Polio, *Haemophilus influence* type b conjugate; Injection 2: recombinant Hepatitis B; Injection 3: pneumococcal conjugate vaccine.  Ethical approval: obtained |
| **Outcomes** | **Primary** we examined reactivity and recovery to pain of immunization injections, in preterm compared with full-term infants at 4 months CCA. |
|  | **Secondary** We explored sex differences in this study |
| **Results** | "There was no main effect for Subgroup or Sex, and CCA was not significant (each P >0.49). Importantly, there was a significant Subgroup X Sex interaction (P=0.045, Z=0.07)" AND "The girls did not differ significantly (P=0.64). For boys, after adjusting for age (CCA, P=0.36), planned contrasts indicated lower overall cortisol concentrations in the ELGA and VLGA preterm boys combined compared with the full-term boys (P=0.028), but no difference between the ELGA and VLGA boys."   - Apparently no differences between boys and girls in full-term group (“no main effect”), although not quite sure if that was analyzed. |

### Grunau 2010 [28]

### Gunnar 2009 [29]

| **Methods** | **Design** Cross-sectional |
| --- | --- |
|  | **Setting** “An urban region of the Midwest”, United States |
|  | **Timing**  Baseline home cortisol, TSST-C |
| **Participants** | ***n=*** 82 |
|  | **Subjects** Subjects were recruited from a list of families who previously had indicated interest in taking part in developmental research |
|  | **Age** Four ages groups: 9 (m=9.79, SD 0.16), 11 (m=11.57, SD 0.15), 13 (m=13.55, SD 0.46) and 15 (m=15.55, SD 0.47) |
|  | **Sex** 22 9-year-olds (12 girls), 18 11-year-olds (9 girls), 22 13-year-olds (10 girls), and 20 15-year-olds (9 girls) |
|  | **Exclusion criteria** Seven children were excluded from analysis because of obesity (n = 3), use of asthma medication on the day of testing (n = 1), deviation from normal sleep patterns (waking after 1 p.m.) on the day of testing (n = 2), and refusal to complete the session (n = 1) |
| **Protocol** | Eight saliva samples were taken in the laboratory as follows: three were obtained over the adaptation period, one after consent/assent (M = 4:10 p.m.), one 15 and one 25 min later (adaptation cortisol 1–3). The last was considered baseline (see below) and its collection started the timing for the TSST-C. Samples were then taken immediately after the speech preparation period (+10 min), after the speech/math period (+20 min), and then at 10-min intervals until +70 min. To provide a home baseline, participants took saliva samples on two regular school days at 4 p.m. and 5 p.m. The 4 p.m. sample corresponded to the first laboratory adaptation sample and the 5 p.m. sample corresponded to the +30 min response sample which was anticipated to be the timing for peak cortisol response to the TSST-C  Ethical approval: consent/assent was obtained from participants |
| **Outcomes** | **Primary** The study below was designed to examine the following questions about the normative development of both basal and stress reactivity of the HPA system as indexed using salivary cortisol: (a) are there increases in salivary cortisol basal levels and/or stress reactivity between 9 and 15 years, (b) are any increases similar for boys and girls, (c) are increases relatively linear across these years, and (d) are they associated with measures of sexual maturation? |
|  | **Secondary** |
| **Results** | Cortisol at home: Neither the main nor interaction effect for gender was significant  To coming to the lab: Although a significant cortisol response to the laboratory was obtained, F (1, 69) = 5.38, p = .023, η G2 = .07 (Bakeman, 2005), there were no significant interaction effects of trials with gender or age group.  Adaptation period: only a significant age group effect.  In the present study, although the means for cortisol reactivity to the TSST-C were higher for the 13-year-old girls and 15-year-olds of both genders than for the 9-year-olds, perhaps because of insufficient power, the difference was statistically nonsignificant. The evidence of a marginally significant association with puberty scores, however, would tend to support the possibility that rising HPA reactivity with sexual maturation is superimposed on rising basal cortisol levels. There was no evidence that by 15 years, greater HPA reactivity was peculiar to girls. However, at 13 years, girls were clearly more HPA reactive than boys of the same age. We cannot tell from the present study whether this gender difference in HPA reactivity during early adolescence might contribute to the gender difference in rising rates of depression at this age.  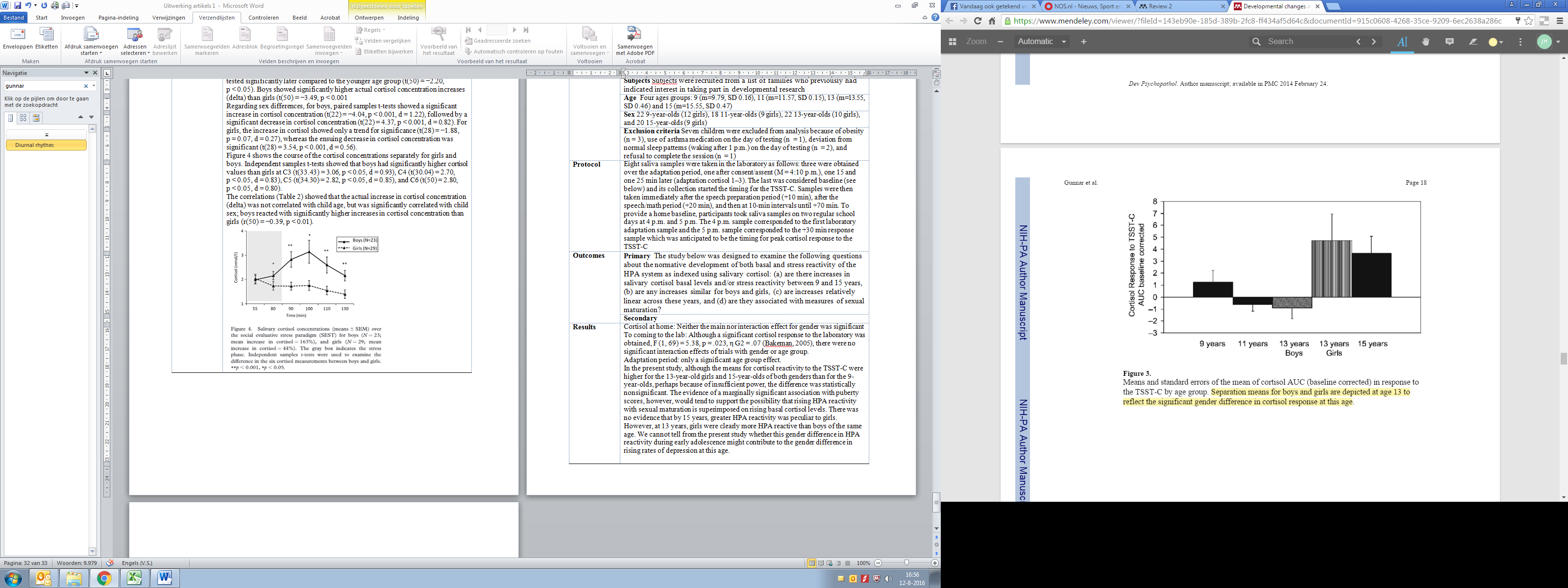 |

### Gunnar 2010 [30]

| **Methods** | **Design** Cross-sectional |
| --- | --- |
|  | **Setting** “Major metropolitan area” |
|  | **Timing**  Daycare attendance |
| **Participants** | ***n=*** 151 |
|  | **Subjects** recruited from family-based day-care settings in a major metropolitan area. Selection criteria were: child age 3–4.5 years (M= 3.81,SD = 0.23), full-time care (‡20 hr per week; M=40.61 hr, SD = 8.26 hr), and at least 2 months attending the present day-care home (M= 27.52 months, SD = 13.40 months, range = 2–48 months). |
|  | **Age** 3–4.5 years (M= 3.81,SD = 0.23) |
|  | **Sex** 54.3% girls |
|  | **Exclusion criteria** Not further specified than mentioned under “subjects” |
| **Protocol** | At day care, samples were collected by the day-care provider on 2 days between 10:00 and 11:00 a.m. and 3:00 and 4:00 p.m. These times were chosen based on previous work showing that when four samples of cortisol are obtained (midmorning, noon, immediately postnap, late afternoon), the rise in cortisol over the child-care day can be reliably assessed at these two time points (Watamura, Sebanc, & Gunnar, 2002). Providers were asked to avoid sampling immediately before a meal, to not give the child caffeinated drinks or dairy products within an hour of sampling, and to wait until 30 min after the child got up from a nap to sample to avoid the decrease in cortisol typically seen over nap time (Watamura et al., 2002). To determine the actual time of sampling, the cotton dental rolls were supplied in a bottle with a MEMS V Track Cap (Aardex, Zug, Switzerland), which automatically recorded the time when the container was opened. Use of such devices allows verification of compliance with sampling protocols and also increases compliance (Kudielka, Broderick, & Kirschbaum, 2003). The care provider also completed a brief diary on each day of sampling recording timing of sample, nap times, and meal times.  Ethical approval: parental consent was obtained |
| **Outcomes** | **Primary** The purpose of this study was to develop a better understanding of the factors associated with rise in cortisol over the child-care day. There were five goals: a) determine whether cortisol increases of the day in family da-care settings; b) examine the extent to which structural and process measures in child-care quality are associated with the magnitude of cortisol increase over the child-care day; c) explore whether anxious, vigilant or angry, aggressive behavior is related to the child-care cortisol stress response; d) examine whether gender moderates child behavior-cortisol rise associations; and e) examine whether child behavior mediates or moderates associations between process measures of child-care quality and the child-care cortisol stress response. |
|  | **Secondary** |
| **Results** | “The 2·2· 2 ANOVA of the home and day-care cortisol measures revealed that neither the effect of child sex nor interactions with child sex was significant.”  As shown in figure 1a, for girls but not boys, Anxious, Vigilant Behavior was associated with larger rises in cortisol over the day-care day. As shown in Figure 1b, for boys, Angry, Aggressive Behavior was associated with the rise in cortisol.  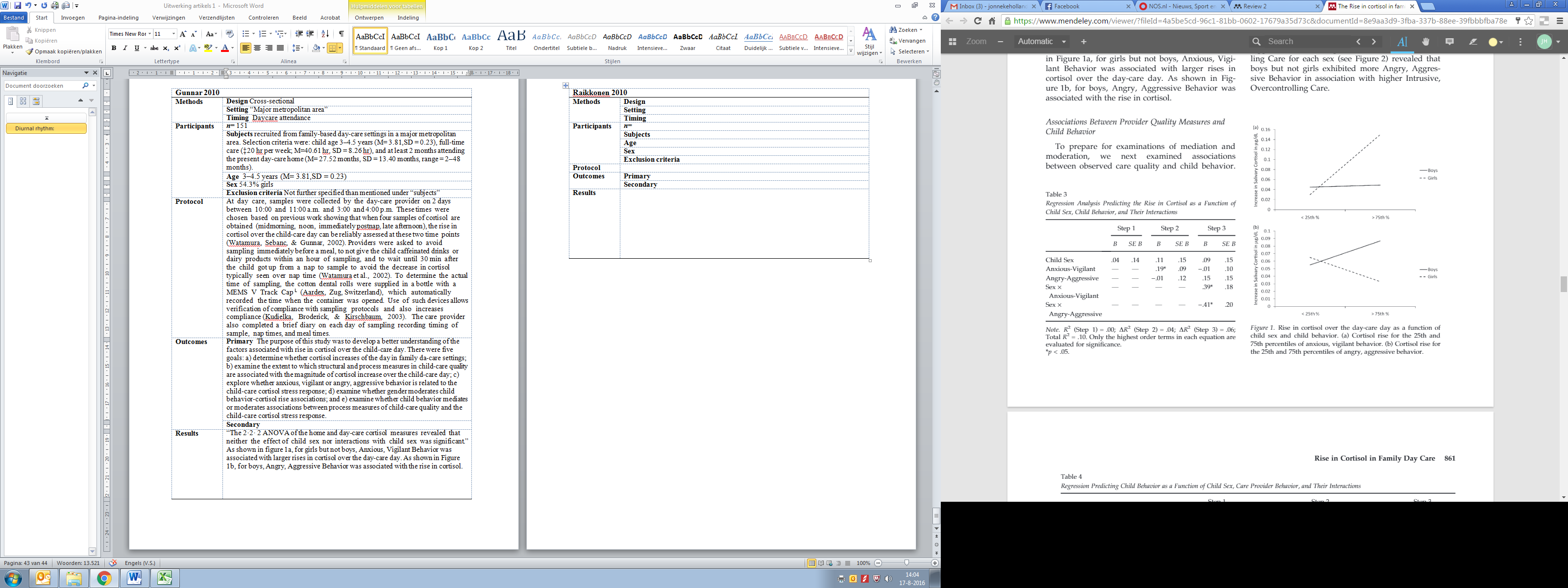 |

### Hackman 2012 [31]

| **Methods** | **Design** Part of a longitudinal study |
| --- | --- |
|  | **Setting** Not specified |
|  | **Timing**  Parent-Adolescent Conflict Discussion |
| **Participants** | ***n=*** 180 |
|  | **Subjects** The families were recruited from two sources: 62 families were recruited from an earlier longitudinal study of children who had participated in Head Start (Ackerman, Kogos, Youngstrom, Schoff, & Izard, 1999) and 54 families were recruited from a list of 13-year old children whose families met income guidelines for free and reduced priced lunch. |
|  | **Age** 12 to 14 (M = 13.2) years |
|  | **Sex** 92 female and 88 male |
|  | **Exclusion criteria** Families attending morning lab sessions (Due to typically high diurnal levels of cortisol during morning hours, response to the morning laboratory visits may be confounded with steeper declines in cortisol levels) |
| **Protocol** | “Parent-adolescent conflict discussions tested caregivers’ and adolescents’ ability to maintain constructive conversation about a disagreement. Conflict discussions were established by using the major areas of disagreement identified by the adolescent and caregiver on the Issues Checklist, which included 13 topics thought to be sources of parent-adolescent conflict (e.g., money, communication, and curfew). The order of the adolescent and caregiver topics was counterbalanced. When both caregiver and adolescent identified the same topic, a topic that was rated second was chosen. Dyads were instructed to discuss the topic and to try to reach an agreement. The interviewer then left the room and returned after 5 minutes to introduce the next topic.”  Cortisol samples: “The first salivary sample was taken during the first 5 minutes after the dyads arrived in the lab, immediately following the signing of consent forms, with an average time of 4:26 p.m. (SD = 1 hour 40 minutes, range = 12:05 p.m. to 7:15 p.m.). Caregivers and adolescents were then separated for approximately 75 minutes (range 58 to 92 minutes) to complete individually administered interviews and questionnaires. A second salivary sample was taken after the adolescent completed his or her interview. The average time for the second sample was 5:40 p.m. (SD = 1 hour 50 minutes, range = 1:05 p.m. to  8:30 p.m.). When both the caregiver and adolescent had completed the interviews, they were reunited in an observation room where they participated in a series of interactions. The final salivary sample was taken at the end of the interactions, 30 minutes after the beginning and 20 minutes after the end of the conflict discussion. The average time for the final post-conflict sample was 6:55 p.m. (SD = 1 hour 39 minutes, range = 1:55 p.m. to 10:10 p.m.).”  Ethical approval: consent form were signed, not specified whether a review board approved the study |
| **Outcomes** | **Primary** to examine gender differences in adrenocortical activity and antisocial behavior during early adolescence |
|  | **Secondary** |
| **Results** | “In the first set of analyses, T1, T2, and T3 cortisol samples were used as a within-subjects repeated measure, with gender as an independent variable and start time of the visit as a covariate. Time of visit produced a large between-subject effect on cortisol levels, with subjects who attended sessions earlier in the day having higher levels of cortisol, F(1, 119) =30.53, p < .001. Within-subject contrasts in cortisol levels between T1 and T2 and between T2 and T3 were non-significant. Gender did not produce a between-subjects effect.”  Other gender associations: only as moderator between cortisol reactivity and anti-social behavior |

### Haen 1984 [32]

| **Methods** | **Design** Cross-sectional |
| --- | --- |
|  | **Setting** Japan (and Germany) |
|  | **Timing**  Diurnal rhythm (no car) |
| **Participants** | ***n=*** 64 Japanese children, 29 obese German children |
|  | **Subjects**  All patients were in apparent good health at the time they were tested, *just before discharge from the hospital* |
|  | **Age** 1 month to 15 years old |
|  | **Sex** Not specified |
|  | **Exclusion criteria** Not specified |
| **Protocol** | Published data on blood cortisol at 6-hour intervals, usually at 6:00, 12:00, 18:00 and 00:00, were available. All patients were in apparent good health at the time they were tested, just before discharge from the hospital.  Ethical approval: not specified |
| **Outcomes** | **Primary** We tested whether the (Minnesotan) paradesm in nand could be used as a reference standard |
|  | **Secondary** |
| **Results** | “A comparison between boys and girls showed no statistically significant differences in circadian cortisol parameters.” |

### Hatzinger 2007 [33]

| **Methods** | **Design** Cross-sectional |
| --- | --- |
|  | **Setting** Basel, Switzerland |
|  | **Timing**  CAR and TSST-C |
| **Participants** | ***n=*** 102 |
|  | **Subjects**  Starting with kindergarten |
|  | **Age** 4.91 +/- 0.44 years |
|  | **Sex** 59 boys and 43 girls |
|  | **Exclusion criteria** Any relevant medical and/or neurological diseases (previously described by Perren et al 2006). None of the participants had been subjected to sleep deprivation, time shifts or intake of any disturbing sub-stances/medication during the 3 months prior to the investigation |
| **Protocol** | CAR: four saliva cortisol samplings were performed by the parents in the morning at 0, 10, 20, and 30 min after child’s awakening.  TSST-C: tests had to be performed during different day times, i.e. in the morning (between 09.00 and 11.00 a.m.;n = 54), in the early (between 2:00 and 4:00 p.m.;n = 30) or later after-noon (between 4:00 and 6:00 p.m.;n = 7). […] In order to reach a standardized contextual baseline, children were familiarized to the situation by the initial contact with the investigator conducting a short pleasant activity such as playing an easy card game, building a tower with wooden pieces and applying a short vocabulary test for distraction (Peabody Picture Vocabulary Test; Dunn and Dunn, 1981 ). During this time frame the first two salivary cortisol samplings were collected taking the first one 10 min and the second one 1 min before starting the MSSB-task. The third (20 min after the MSSB start) and the fourth (35 min after the MSSB start) samples were taken during and at the end of the MSSB-task. After a recovery time of about 10 min the fifth and last MSSB-related saliva sample was collected  Ethical approval: obtained |
| **Outcomes** | **Primary** To characterize basal and stress-responsive HPA system functioning in children during an important period of development, i.e. at the beginning of kindergarten |
|  | **Secondary** 2) to examine whether HPA axis activity is influenced by other factors such as gender already in this age group as young as 5 years; 3) to identify possible different subgroups of kindergarten children and to detect commonalities and differences in their reactive HPA physiology in order to gain hormonal profiles in relation to psychological/behavioural symptom patterns. |
| **Results** | CAR: MC AUC showed a highly significant gender difference (MC AUC: 35.56 ± 19.84 (boys, n = 59) vs. 54.14 ± 30.28 lg/dl (girls, n = 43)" [higher in girls than boys]  TSST-C: "a gender difference was also present in stress- elicited hormone release during MSSB (Fig. 2) (MSSB AUC: 26.34 ± 17.99 (boys, n = 55) vs. 39.02 ± 32.79 (girls, n = 36), F(1,88) = 4.92, p < 0.05, controlling for assessment time)"  Girls (27 high- vs 16 low-secretors) were significantly more high-secretors than boys (24 high- vs 35 low-secretors) (X2(1) – 4.86; P <0.05), which was also supported by the additional odds ratio (OR) calculations showing a significantly increased OR of high-secretor status in girls vs. boys (OR = 2.46, P<0.050. Hence, a strong gender-dependent cortisol release could be observed in the morning.  In order to explore the impact of gender-dependent MC secretor status on HPA regulation under stress conditions it was shown that MSSB AUC varied for girls when MC high-low-secretor status was used as independent factor (F(1.33) = 3.91, P<0.05; always controlling for assessment time, but not for boys (F(1,51) = 1.67, ns), indicating that MC high-secretor female subjects experienced a significant amount of hormonal activation during MSSB. |

### Hostinar 2014 [34]

| **Methods** | **Design** Cross-sectional |
| --- | --- |
|  | **Setting** Large suburban area in the Midwestern United States |
|  | **Timing**  TSST for groups (TSST-G) |
| **Participants** | ***n=*** 191 |
|  | **Subjects** Public school students from two middle schools and one high school |
|  | **Age** 14.4±1.93 years |
|  | **Sex** 100 girls, 91 boys |
|  | **Exclusion criteria** “N = 5 reported using corticosteroid-based medications, which confound cortisol assays; N = 3 used oral contraceptives and exhibited ﬂat cortisol curves; N = 1 had abnormally high cortisol values suggestive of acute infection or other conditions; N = 3 were part of an atypical group of only three participants, which was too different from the conditions experienced by most participants, i.e., group sizes of 5—8 individuals; N = 1 left the study before the beginning of the TSST-G; N = 1 declined to deliver the speech; and, lastly, N = 1 did not provide information on race or ethnicity, which was used as a covariate in cortisol analyses” |
| **Protocol** | TSST for groups.  “Our protocol was inspired by the original TSST-G, but we have made several modiﬁcations for use with adolescents, to reduce participant burden, to ﬁt the constraints of the current study design, and to increase the ecological validity of the task. Com-pared to the original TSST-G, this protocol had the following modiﬁcations: (a) we used speech instructions designed for children or adolescents (i.e., introducing oneself to a hypothetical classroom of students, as suggested by Yim et al., 2010); (b) we only used the public speaking component and not the mental arithmetic task in order to decrease participant burden, reduce the length of the protocol, reduce the time lag between saliva samples, and increase the eco-logical validity of the task (i.e., make the situation more similar to what youth would encounter in daily life); (c) judges wore professional business attire instead of white lab coats to impersonate adults/ofﬁcials that adolescents typically encounter in school settings; (d) participants were seated instead of standing up to eliminate any impact of standing up from the heart rate record (data not included in this report); (e) we varied group size (from 5 to 8 par-icipants instead of equal groups of 6 used by Von Dawans et al., 2011) in order to empirically test whether group size affects cortisol reactivity. However, we retained the critical aspect of the task, which is the social-evaluative threat posed by the judges and the other adolescents in the room (Dickerson and Kemeny, 2004).”  Cortisol sampling: -15, 0, 15, 30, 40 and 50 minutes from start of TSST-G  Ethical approval: obtained |
| **Outcomes** | **Primary** “to test the feasibility and effectiveness of a group protocol for a public speaking task with adolescents (ages 11—18) from diverse racial backgrounds” |
|  | **Secondary** “We tested the effectiveness of our group public speaking task with respect to eliciting increases in cortisol levels and subjective ratings of negative affect, as well as decreases in positive affect. We next examined whether cortisol response trajectories differed by age, gender, and race. Finally, we tested whether group size or speaking order mattered for cortisol stress reactivity.” |
| **Results** | “Males tended to have higher intercepts than females, but this difference was not statistically signiﬁcant (ˇ = 0.23, SE = 0.12, p = 0.056), with no other gender differences in anticipatory responses or task reactivity.” |

### Hostinar 2015 [35]

| **Methods** | **Design** Cross-sectional |
| --- | --- |
|  | **Setting** Large urban Midwestern area |
|  | **Timing**  Modified TSST (TSST-M), an adaptation of the TSST-C |
| **Participants** | ***n=*** 81 (40 children, 41 adolescents) |
|  | **Subjects** “ typically developing participants”, “ All participants were raised by their birth families in a large urban Midwestern area” |
|  | **Age** Children: 9.97±0.52 years, adolescents: 16.05±0.39 years |
|  | **Sex** Children: 20 girls, 20 boys. Adolescents: 20 girls, 21 boys |
|  | **Exclusion criteria** Autism Spectrum Disorder, Fetal Alcohol Syndrome, or any other major developmental disorder; and use of steroid medications (due to their interference with cortisol assay results) |
| **Protocol** | “This paradigm consisted of a public speaking task (introducing oneself to a hypothetical new classroom of students) and a mental arithmetic task (subtracting out loud by 7s from 758 for adolescents or by 3s from 307 for children). The participant was alone in the room when giving the speech and performing the mental arithmetic in front of a two-way mirror and a conspicuously placed video camera. The participant was told that the experimenter and two other teachers (one male, one female) would watch them from the other side of the mirror and rate their speech performance and their arithmetic accuracy. This was accomplished using an audio recording of two adults who sternly provided instructions for the speech.”  Cortisol samples: Session 1: 45, 65, 85, and 105 minutes after arrival, corresponding to 20 minutes since the end of the relaxation period and 20, 40, and 60 minutes after the end of the stress task. Session 2: 45 and 65 minutes from arrival  Ethical approval: obtained |
| **Outcomes** | **Primary** to investigate the effect of experimentally-provided parent support on the cortisol response to a modified TSST in 9-10-year-olds and 15-16-year-olds. |
|  | **Secondary** the effect of sex and its interactions with age and condition were also explored |
| **Results** | “Sex differences were also considered in this first model. Main effects were not interpreted given that there was a significant interaction of age x sex on the linear term, F(1,216) = 4.15, p =.04, and quadratic term, F(1,158) = 5.73, p =.02. Follow-up simple slope analyses indicated that 9-10-year-old girls had greater cortisol reactivity than 9-10-year-old boys (linear term: β = .16, SE = .06, t(216) = 2.79, p = .006; quadratic term: β = -.05, SE = .02, t(158) = -3.13, p = .002). Among adolescents there were no sex differences in cortisol intercepts or slopes (p's >.27).” |

### Ji 2016 [36]

| **Methods** | **Design** Longitudinal |
| --- | --- |
|  | **Setting** rural and semi-rural areas of Pennsylvania |
|  | **Timing**  TSST-C |
| **Participants** | ***n=*** 135 |
|  | **Subjects** Healthy young adolescents recruited through letters sent to eligible students through American Student List (ASL), or through flyers and from telephone responses to e-mails sent to staff at a large university |
|  | **Age** Girls were ages 8, 10, or 12 years (n: 69, M: 10.5 years, SD: 1.6) and boys were ages 9, 11, or 13 years (n: 66, M: 11.5 years, SD: 1.6) |
|  | **Sex** 69 girls, 66 boys |
|  | **Exclusion criteria** not on medications that would interfere with hormone levels (e.g., oral steroids); and free from chronic physical (e.g., diabetes) or serious mental health problems |
| **Protocol** | The TSST-C was administered  Cortisol samples: “Two saliva samples were collected 25 min (S1) and 5 min (S2) prior to administration of the TSST-C. The TSST-C procedure required an average of 15 min to complete the tasks. Three samples were collected 15 min (S3), 25 min (S4), and 35 min (S5) after the start of the TSST-C.”  Ethical approval: obtained |
| **Outcomes** | **Primary** “ (1) to examine a reactivity and recovery model of cortisol response using linear, quadratic and piece-wise latent growth models in a sample of young adolescents; (2) to investigate whether age or sex explained the variability in cortisol reactivity and recovery; and (3) to examine heterogeneity in the patterns of the cortisol response through exploration of the existence of meaningful subgroups using latent class analysis.” |
|  | **Secondary** |
| **Results** | “A sex difference in cortisol reactivity emerged at wave 3 controlling for the effect of pubertal timing; girls showed a greater reaction to the stressor than boys (b ¼ .65, p < .05). No signiﬁcant sex differences in cortisol recovery were found at any wave.”  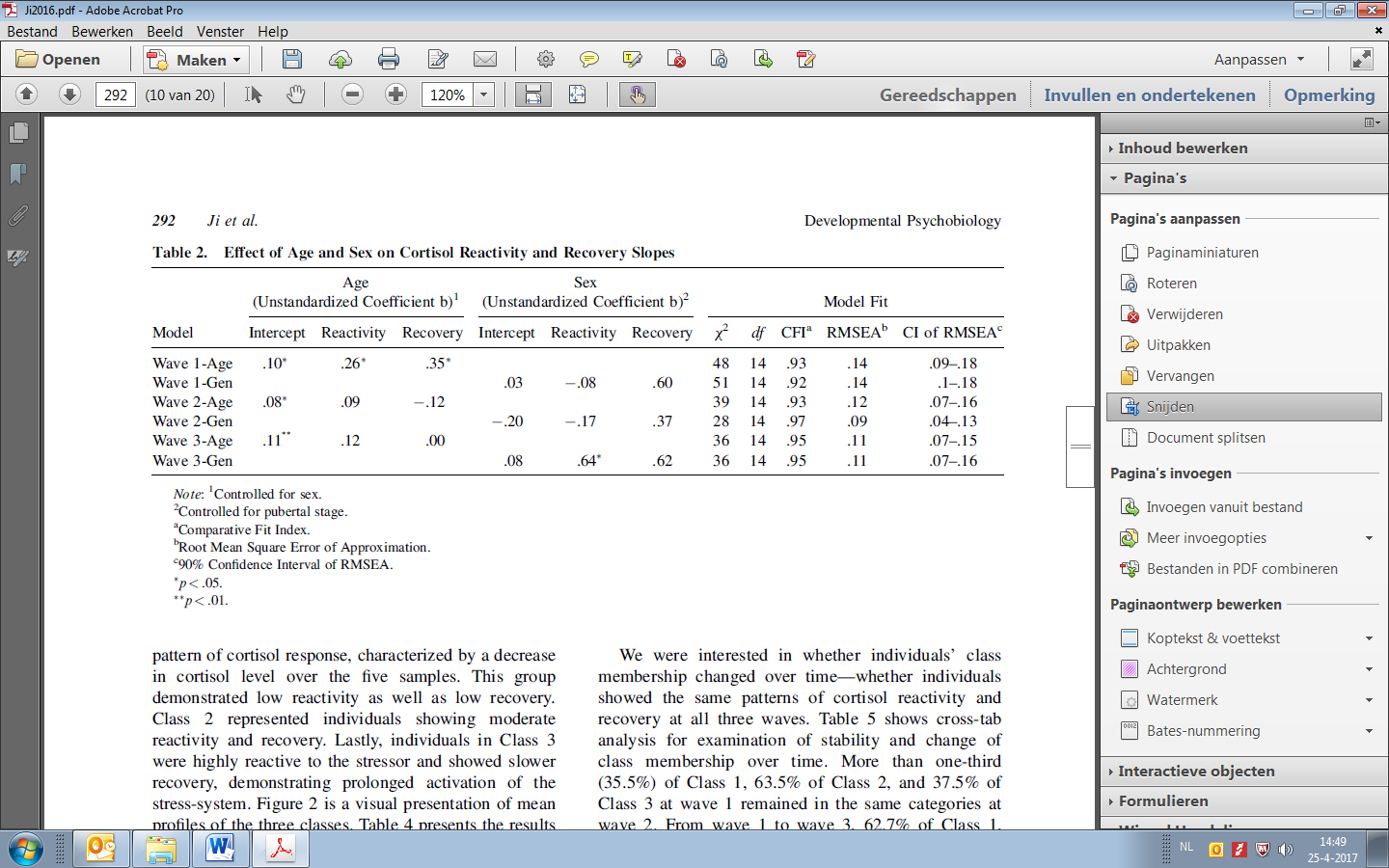 |

### Jones 2006 [37]

| **Methods** | **Design** Cohort study |
| --- | --- |
|  | **Setting** Southampton, UK |
|  | **Timing**  Diurnal rhythm + CAR, TSST-C |
| **Participants** | ***n=*** 140 |
|  | **Subjects** Healthy children who have been followed up since 12 wk gestation |
|  | **Age** 7-9 years |
|  | **Sex** 68 boys, 72 girls |
|  | **Exclusion criteria** Not specified |
| **Protocol** | To assess their baseline adrenocortical function, the children were asked to use a home-testing kit to collect salivary cortisol at five time points (on awakening, 30 min later, 12:30, 15:30 and 18:30 on a restful day, when the children were taking no part in activities.  TSST-C: Start: in the afternoon (1330-1430). Saliva samples were collected at seven time points during their visit (on arrival, 1h later, just before the TSST-C, and then at 10-min intervals after the stress test).  Ethical approval: obtained |
| **Outcomes** | **Primary** to examine the relationship between birth weight and HPAA stress responsivity |
|  | **Secondary** |
| **Results** | Home profiles did not differ significantly between genders with the exception of the awakening response, which was evident in boys but not girls (P = 0.04 for difference in increment between awakening and 30 min later). During the clinic visit, cortisol profiles were similar in boys and girls until the poststress measures, which were greater in the girls (P<0.05) for the last three samples). In both genders, cortisol had risen in anticipation of the stress test (P<0.001 for comparisons of third samples with all following samples), whereas boys showed no further increment in cortisol.  In boys, we found a strong inverse relationship between birth weight adjusted for gestational age and HPAA stress responsivity when home cortisol profiles were used as a baseline but not when prestress clinic levels were used.  Birth weight of girls was not associated with HPAA responsivity or evening nadir levels but was inversely associated with morning peak cortisol.  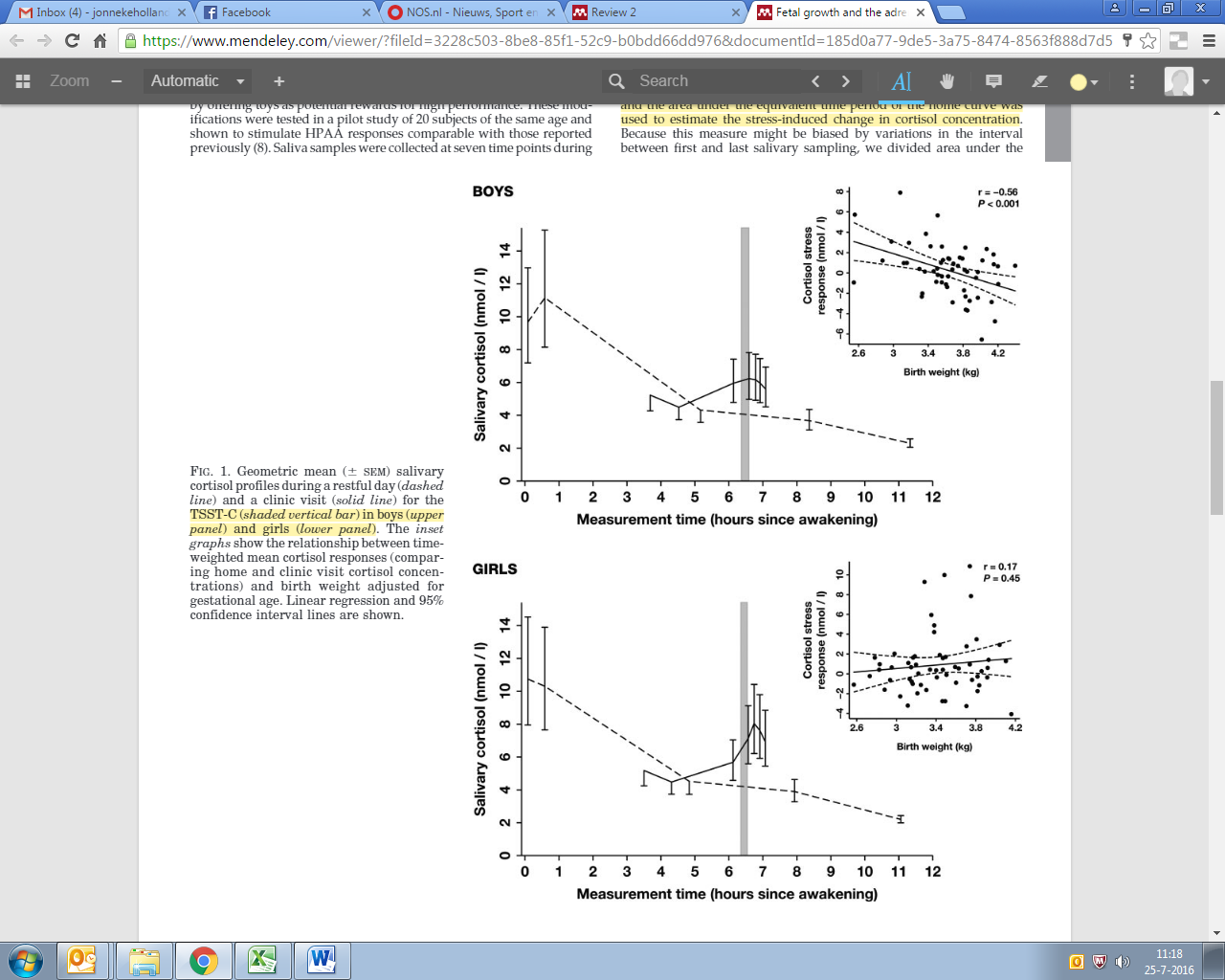 |

### Kelly 2008 [38]

| **Methods** | **Design** Cross-sectional |
| --- | --- |
|  | **Setting** Central Clydesied Conurbation, a predominantly urban area in and around Glasgow city in the West of Scotland |
|  | **Timing**  Diurnal rhythm |
| **Participants** | ***n=*** 2995 |
|  | **Subjects** Pupils from 22 schools, attending Secondary (the final statutory year of schooling) |
|  | **Age** 15 years + 5 months, SD 3.8 months |
|  | **Sex** Not in article? |
|  | **Exclusion criteria** No known weight/length, not providing (both) cortisol samples or extreme values (blood contamination assumed) |
| **Protocol** | At around 5 minutes into questionnaire completion, the whole group was instructed to remove and chew on the cotton wool for approximately 2 min, after which completed samples were collected by the survey team. This process was repeated half an hour later, around 5 min before the end of the session (mean time: 31.3 min).   - From this, a rate of decline/min was calculated   Ethical approval: obtained |
| **Outcomes** | **Primary** To present salivary cortisol levels among a large sample of community-living adolescents |
|  | **Secondary** to examine differences in respect of factors previously found to be associated with salivary cortisol measures. |
| **Results** | Median (IQR) cortisol levels for males and females were 10.5 (8.1) and 11.6 (9.3) nmol at time 1 (T1), and 8.2 (6.0) and 8.1 (6.5) nmol/L at time 2 (T2), representing a change (decline) per minute of 0.07 (0.16) and 0.10 (0.15). Sex differences in respect of the T1 levels and the change per minute were significant (t=-4.9, p<0.001; t=-5.9, p<0.001).  Also: several associations between decline and other factors, differing between genders. |

### Khilnani 1993 [39]

| **Methods** | **Design** Cross-sectional |
| --- | --- |
|  | **Setting** Massachusetts General Hospital |
|  | **Timing**  Cortisol response to elective surgical procedures |
| **Participants** | ***n=*** 98 |
|  | **Subjects** Patients undergoing elective surgical procedures. All patients received general anesthesia. None of the patients received a narcotic premedication. |
|  | **Age** 2-20 years |
|  | **Sex** 62 males, 36 females |
|  | **Exclusion criteria** Patients receiving medications known to interfere with hormonal responses to stress |
| **Protocol** | Venous blood samples were collected preoperatively and 1 hour after the termination of anesthesia. Baseline preoperative samples were collected at least 20 minutes after intravenous catheter insertion in order to allow the sympathetic nervous system to stabilize following the catheter insertion. For ethical reasons, baseline preoperative samples were collected after inhalational anesthetic induction but before surgical incision, in all patients younger than 5 years of age and in older children who did not want an intravenous cannula placed before anesthesia. Postoperative venous blood samples were obtained 1 hour after surgery from the indwelling intravenous catheter after discarding 2 ml of blood in the recovery room.  Ethical approval: obtained |
| **Outcomes** | **Primary** To study changes in serum prolactin and cortisol concentration in children undergoing elective surgical procedures, under general anesthesia. |
|  | **Secondary** |
| **Results** | “Females in all four age groups showed a greater surgery-induced increase in prolactin values than did males (P<0.05), while no differences were found in the cortisol responses between males and females.” |

| **Methods** | **Design** Cross-sectional |
| --- | --- |
|  | **Setting** Linköping, Sweden |
|  | **Timing**  Diurnal rhythm |
| **Participants** | ***n=*** 342 (complete data on 299 children) |
|  | **Subjects** Subjects from two schools were chosen to participate. Taken together, these two schools had essentially the same demographic make-up as the Swedish population at large regarding socioeconomic level and ethnicity. |
|  | **Age** 9.5 years (SD 1.9), range 6-12 years |
|  | **Sex** 160 girls, 175 boys (complete data: 149 girls, 150 boys) |
|  | **Exclusion criteria** Not specified |
| **Protocol** | Salivary samples were collected on four consecutive days during the autumn. (…) sampling was mainly conducted during school hours. Samples at school were taken in the early morning (8:30 am) and late morning (10.30 am), while samples at home were taken in the evening (9:00 pm or at bed time if earlier) on the first 3 days, and at 8:30 pm on day four.  Ethical approval: obtained |
| **Outcomes** | **Primary** “The aim of this study was to examine diurnal salivary cortisol levels in relation to BMI, age and sex in a healthy population of schoolchildren.” |
|  | **Secondary** |
| **Results** | “No significant differences were found between the sexes for cortisol levels at any of the time points” |

### Kjolhede 2014 [40]

### Knutsson 1997 [41]

| **Methods** | **Design** Cross-sectional (also partly longitudinal) |
| --- | --- |
|  | **Setting** Goteborg, Sweden |
|  | **Timing**  Diurnal rhythm |
| **Participants** | ***n=*** 235 (28 for the longitudinal study) |
|  | **Subjects**  All children were healthy and well-nourished and had normal thyroid, liver, and kidney functions. Coeliac disease was excluded. No child was receiving any medical treatment |
|  | **Age** 2.2–18.5 y |
|  | **Sex** 162 boys, 73 girls (18 boys and 10 girls for longitudinal study) |
|  | **Exclusion criteria** Not specified aside from what has previously been mentioned |
| **Protocol** | The children were accommodated at the hospital for at least a 24-h period with the minimal stress possible, during which time they received a normal diet and were allowed routine activity and sleep. A heparinized needle was inserted into an antecubital vein mainly in the afternoon of the day before the sampling began. Blood sampling was initiated at 1400 h, and further samples were obtained at 1800, 2200, 0200, 0400, 0600, and 1000  Ethical approval: obtained |
| **Outcomes** | **Primary** to gain insight into adrenocortical activity throughout childhood and to assess the relationship, if any, between serum cortisol levels and age, sex, weight, height, body composition, and pubertal stage. |
|  | **Secondary** to obtain reference data for serum cortisol levels by studying a large group of healthy boys and girls |
| **Results** | Prepubertal: There was no evidence of any significant differences in the mean cortisol profiles in relation to gender or height.  Pubertal: there was no difference between boys and girls during pubertal development, except for a minor difference (P<0.05) at pubertal stage 2. |

### Kryski 2013 [42]

| **Methods** | **Design** Cross-sectional |
| --- | --- |
|  | **Setting** Not specified |
|  | **Timing**  Matching task |
| **Participants** | ***n=*** 409 |
|  | **Subjects** “Participants were an unselected community sample of 409 children (201 boys; 49.1%) between 36- and 47-months old (M = 40.72, SD = 3.51) and their primary caregivers recruited for a study examining biological and contextual correlates of child temperament. Children were recruited by contacting families through a university’s research participant pool and by advertisements placed in local daycares, preschools, recreational facilities, and on websites.” |
|  | **Age** 40.72±3.51 months |
|  | **Sex** 201 boys, 208 girls |
|  | **Exclusion criteria** “Children with signiﬁcant medical or psychological problems were excluded from participation via a screening procedure administered by trained study personnel at the recruitment stage” |
| **Protocol** | “Brieﬂy, each child was videotaped performing a matching task using a large felt board on which numerous bear and frog icons were afﬁxed. A large toy replica of a trafﬁc stoplight (a Yacker-Tracker1, modiﬁed for the present study) was adjacent to the board, and the experimenter used an unobtrusive remote control to manipulate the trafﬁc light. At the beginning of the task, the child was allowed to choose a prize from an assortment of small toys that s/he was told would be awarded for successful completion of the task. The child was then told that each icon on the board should be matched with a speciﬁc colored ball. The child was instructed that the trafﬁc light would show how much time they had to place all the balls correctly, and that when the light turned red and a buzzer sounded, they were out of time. Children were told that the matching task was easy to do, and that even ‘‘little kids’’ could ﬁnish on time. After 3 min of matching, the red light was activated, and the child was told that they had not ﬁnished in time. Two subsequent, identical trials occurred in which children were again unsuccessful at ﬁnishing the task. Upon the conclusion of the third trial, the experimenter exclaimed that the light was broken and that the child had not been given enough time to ﬁnish the task. The child’s matching skills were praised and s/he was given the selected prize.”  Cortisol samples: “Saliva samples were obtained at baseline (immediately before the introduction of the stress task), and at 10, 20, 30, 40, and 50 min following the end of the matching task”  Ethical approval: Written consent of subjects was obtained |
| **Outcomes** | **Primary** “examined whether child sex moderated associations between symptoms of psychopathology and cortisol reactivity to a standardized stress task” |
|  | **Secondary** |
| **Results** | 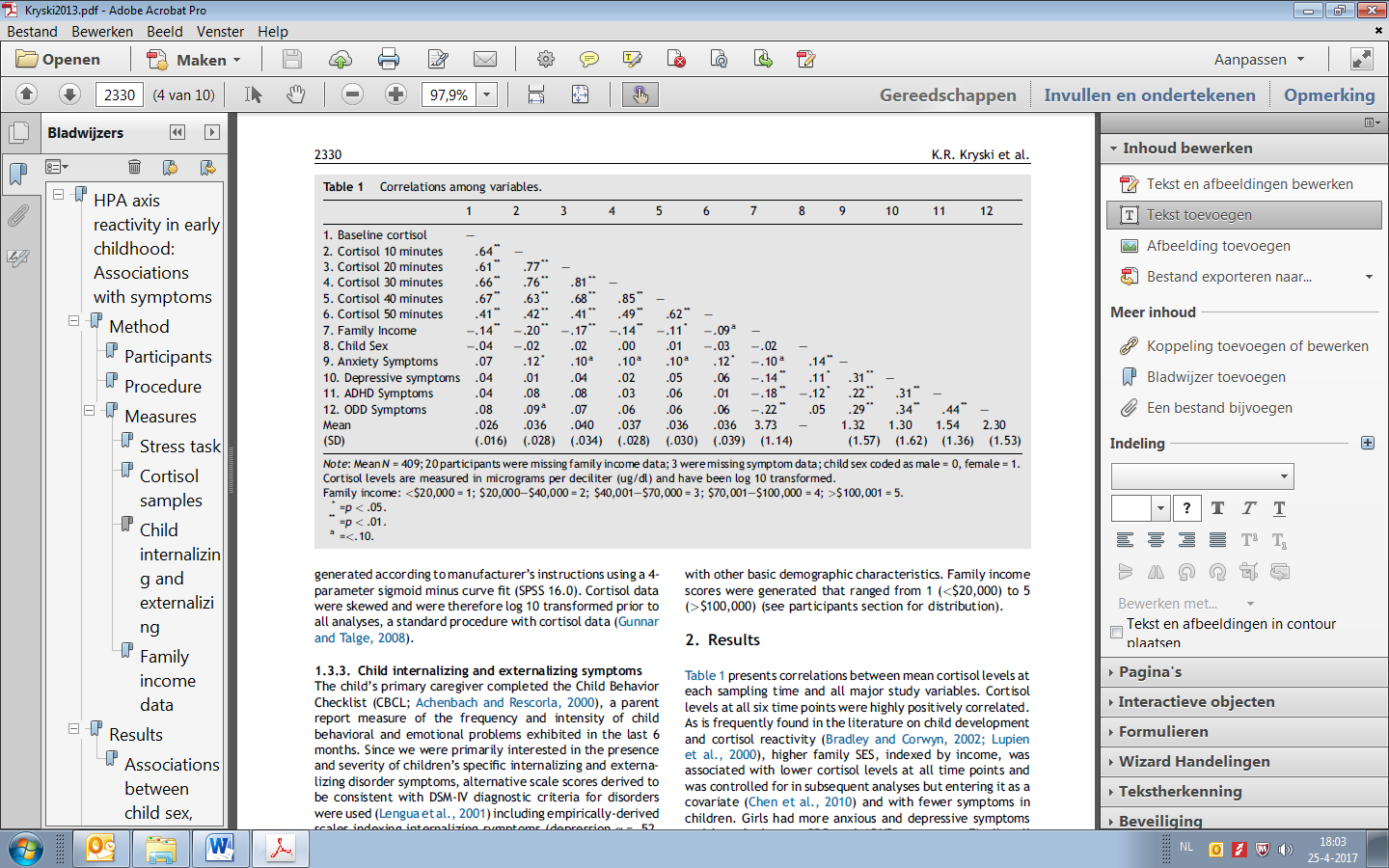 |

### Kudielka 2004 [43]

| **Methods** | **Design** Cross-sectional (?) |
| --- | --- |
|  | **Setting** University of Trier |
|  | **Timing**  TSST-C |
| **Participants** | ***n=*** 31 |
|  | **Subjects** “The children studies focused on group differences in the stress reactivity between healthy volunteers and children with atopic dermatitis or allergic asthma” |
|  | **Age** 12.1 years (SD: 0.3 years) |
|  | **Sex** 16 boys and 15 girls |
|  | **Exclusion criteria** “Volunteers with psychiatric, endocrine, cardiovascular, other specific chronic diseases or those medicated with psychoactive drugs, B-blockers, estrogens (including oral contraceptives), or glucocorticoids were not admitted to the studies.” |
| **Protocol** | “All stress sessions took part in the afternoon (3pm-7pm). For blood samples, an intravenous catheter was inserted in older and younger adults. The sampling collection begun after a rest period of 45 min. (…) Saliva samples were obtained in all 102 subjects using Salivette sampling devices directly before onset of the stress test, as well as 1, 10, 20, and 30 min after stress exposure.”  Ethical approval: obtained |
| **Outcomes** | **Primary** “the present reanalysis aims to contribute to the question of age and gender effects on HPA axis stress responses including healthy male and female elderly adults, young adults, as well as children. |
|  | **Secondary** |
| **Results** | “No gender differences were observed in either young adults or children (both p=n.s.).  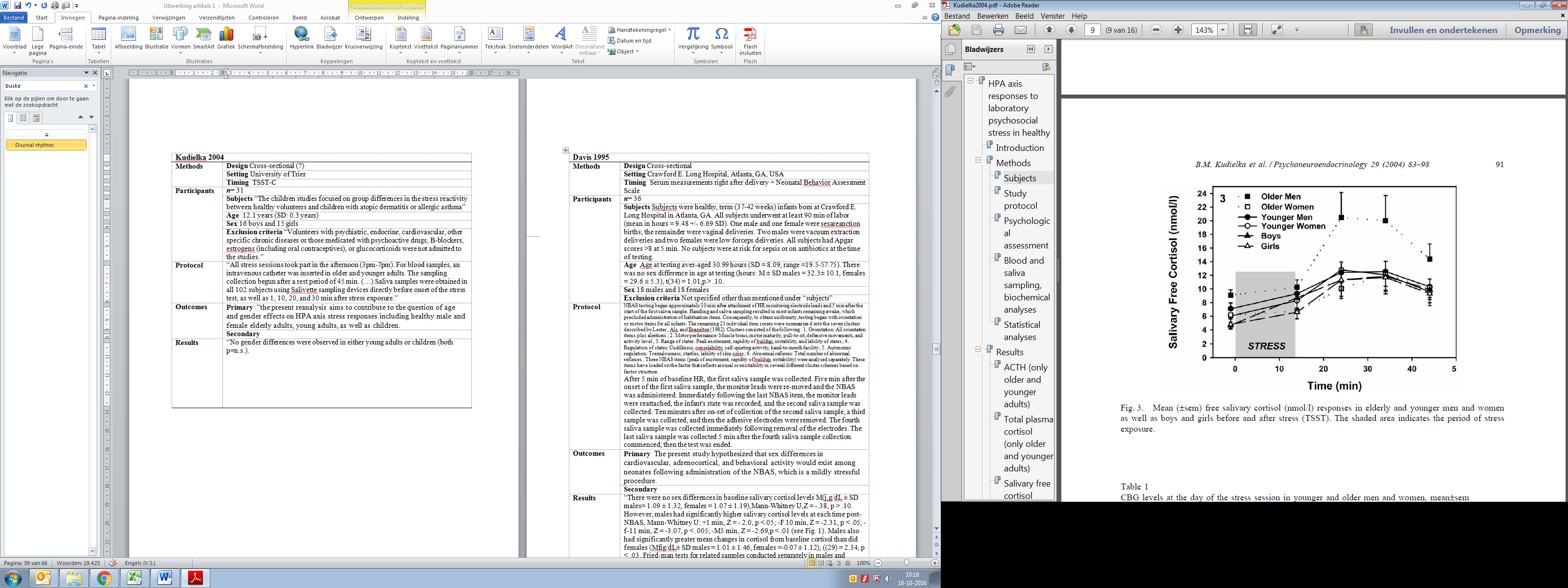 |

### Kuhlman 2015 [44]

| **Methods** | **Design** Cross-sectional study |
| --- | --- |
|  | **Setting** Michigan, USA |
|  | **Timing**  Diurnal rhythm + CAR + Socially-Evaluated Cold Pressor Task |
| **Participants** | ***n=*** 121 |
|  | **Subjects** “Participants were recruited from communities in and around Southeast Michigan via flyers, referrals from clinicians and primary care providers, and advertisements on websites targeting parents of adolescents who have concerns about their child’s mental health. Participants in this study were 70% Caucasian, 10% biracial, 6% African American, 3% Latino, 2% Asian, 6% other, and 3% did not to respond. Participants were from educated families, where mater-nal education for 76% of the participants was a Bachelor’s degree or higher, and 71% of youth lived in families with both biological parents.” |
|  | **Age** 12.8 ± 2.3 yrs |
|  | **Sex** 51% males |
|  | **Exclusion criteria** “a history of a pervasive developmental disorder, were currently taking medications for asthma, were experiencing psychotic symptoms, or currently had any significant medical conditions. “ |
| **Protocol** | CAR and diurnal cortisol regulation:  “Four diurnal salivary cortisol samples at home across 2 consecutive weekdays (…) Participants were asked to refrain from eating or drinking for 1 h before each saliva sample, store saliva samples in a freezer until returning to the laboratory, and keep a log on the days of their home saliva sampling including the time each sample was taken, sleep and wake times, and whether the day included any significant stressors.”  Cortisol samples: “four samples on two consecutive weekdays: immediately after waking, 45 min after waking, just before dinner, and immediately before bed.”  Acute stress reactivity  “All visits began between 1:00pm and 4:00pm and consisted of a 30-min baseline phase, a 5-min stress task, and a 60-min regulation/recovery period for a total of 95 min. Eight saliva samples were collected. Baseline phase. A 30-min baseline phase was used to allow for the regulation of the stress response to any stressors that occurred prior to arrival, including any stress associated with visiting a university laboratory. Participants were encouraged to read or play with puzzles and blocks during this phase and were discouraged from using their mobile phones. The stress task used in this study was the Socially-Evaluated Cold Pressor Task, which was specifically designed and validated for the activation of the HPA-axis in laboratory settings by combining thermal stress and social evaluation (Schwabeet al., 2008). In this task, participants immersed their non-dominant hand into a large bucket of ice water (33—39◦F) for up to 3 min, and were instructed to look directly into a video-camera approximately 12-in. from their face so that their “facial expressions could be recorded.’’ A research assistant stared stoically and redirected participants’ attention towards the camera if needed. If the participant removed their hand before 10 s passed, the research assistant asked the participants to repeat the task until 30 s of immersion were reached.  Regulation phase: Immediately following the stress task, participants watched one of four 60-minNational Geographic documentaries, ‘‘Appalachian Trail’’, ‘‘Ocean Drifters’’, ‘‘The Ballad of the Irish Horse’’, or ‘‘Rainforest.’’ These videos were selected for their lack of significant emotionally arousing content.  Stress reactivity: Seven samples were collected via passive drool at 30 min before the task (−30), immediately before the task (0) and at 25, 35, 45, 55, and 65 min after the start of the task.”  Ethical approval: “All eligible participants and their parents provided signed consent to participate in the study” |
| **Outcomes** | **Primary** “the contribution of physical abuse, emotional abuse, and non-intentional trauma to multiple indices of neuroendocrine functioning (CAR, diurnal regulation, acute reactivity)” |
|  | **Secondary** N/A |
| **Results** | CAR:  “Age and sex were not significant predictors of awakening response (p > .10)”  Diurnal cortisol regulation:  “Sex did not impact cortisol at wakening or the linear decline of diurnal cortisol. However, males showed less deceleration of the diurnal decline later in the day (between dinner and bed-time) than females”  Acute stress reactivity  “Age and sex were not associated with peak cortisol or slopes of activation and recovery” |

### Lashansky 1991 [45]

| **Methods** | **Design** Cross-sectional |
| --- | --- |
|  | **Setting** National University, Santo Domingo |
|  | **Timing**  ACTH stimulation test |
| **Participants** | ***n=*** 43 females, 59 males |
|  | **Subjects** “Normal subjects”. All patients were examined by a single examined (T.G.) to ensure that all children were healthy, their nutritional status was adequate, their growth was appropriate and to evaluate their pubertal development |
|  | **Age** 2months to 17 years |
|  | **Sex** 43 females, 59 males |
|  | **Exclusion criteria** not specified |
| **Protocol** | An ACTH stimulation test was performed with a single iv injection of 0.25mg ACTH. The patients did not receive overnight dexamethasone suppression. All tests were performed between 0800 and 1000h. Serum samples were drawn at baseline and 1 h after ACTH infusion.  Ethical approval: obtained |
| **Outcomes** | **Primary** To establish normative data for age- and sex-related changes in adrenal steroidogenesis after ACTH. |
|  | **Secondary** |
| **Results** | No differences were found between males and females or in baseline levels among the various age groups. In females, stimulated cortisol levels only fell significantly in prepubertal children (group 2 vs group 3, P<0.01). In contrast, male infants (group 1) had significantly higher stimulated cortisol levels than older boys in group 2 (P<0.01); a further decline in stimulated levels occurred in early to midpubertal males (group 3 vs group 4, P<0.01).  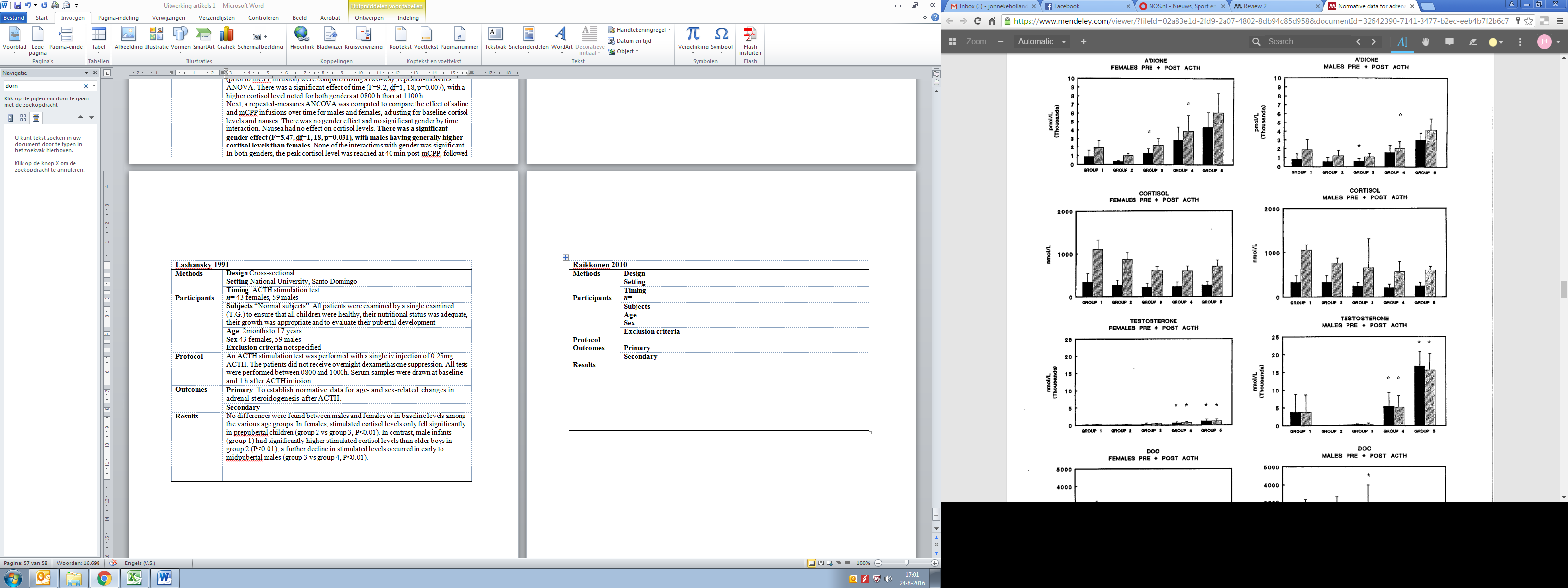  Females:  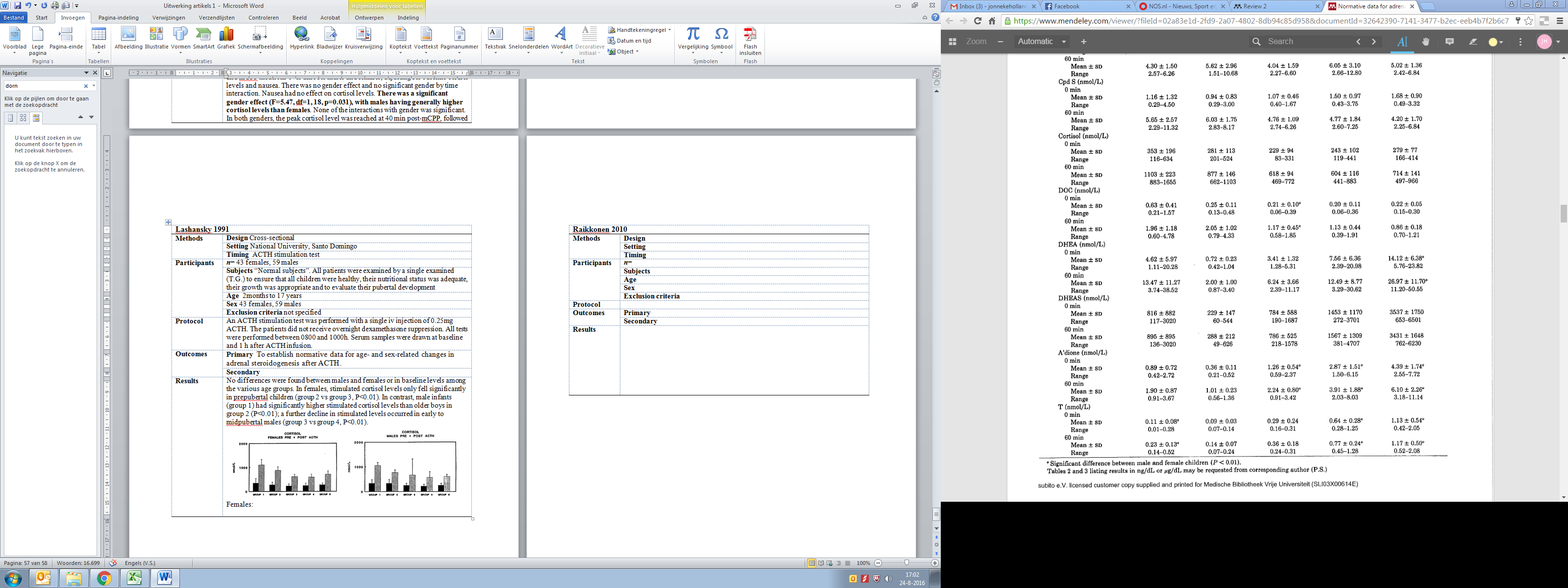  Males:  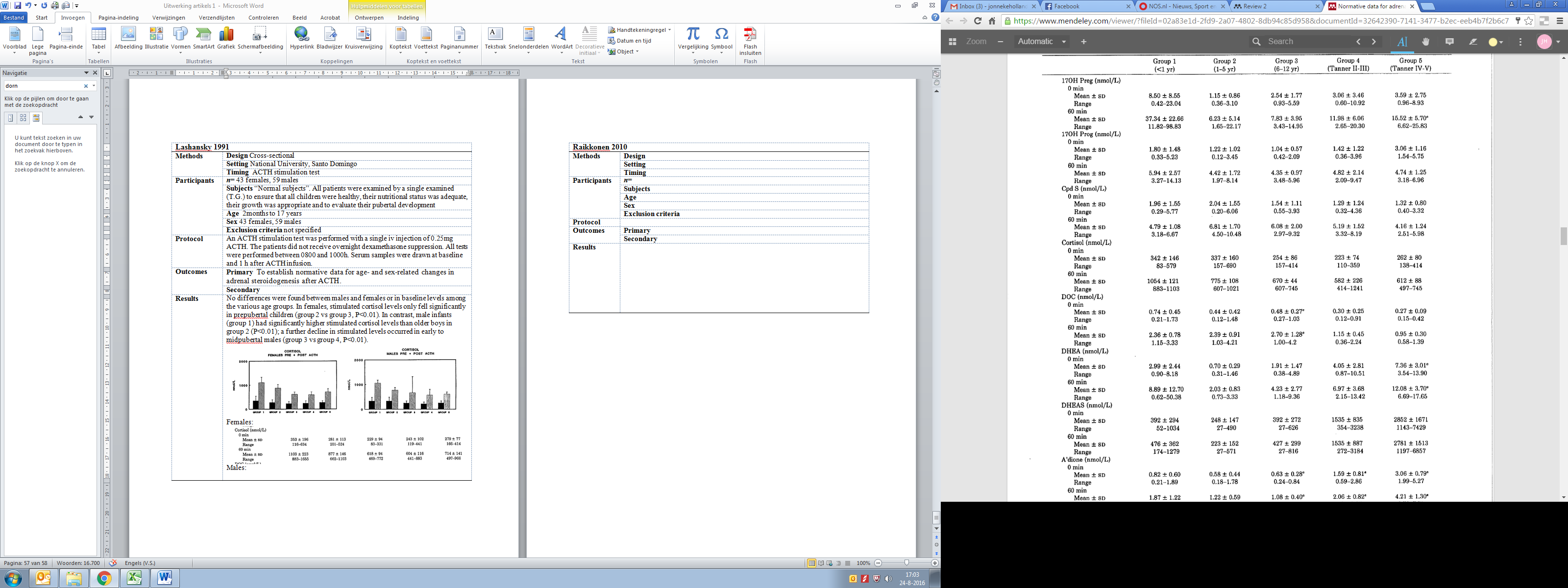 |

### Lopez-Duran 2015 [46]

| **Methods** | **Design** Cross-sectional |
| --- | --- |
|  | **Setting** Not specified |
|  | **Timing**  Socially evaluated cold pressor test (SE-CPT) |
| **Participants** | ***n=*** 115 |
|  | **Subjects** “Participants were recruited to represent the full range of internalizing symptoms with an overrepresentation of children with clinical levels of depressive symptoms. Participant were recruited through online and printed advertisements placed in local newspapers and community centers seeking ‘‘healthy’’ youth, as well as through targeted advertisement seeking youth with significant depression symptoms or a current depression diagnosis.” |
|  | **Age** 12.79±2.26 years |
|  | **Sex** 52 girls, 63 boys |
|  | **Exclusion criteria** (1) having evidence of mental retardation, any pervasive developmental disorder or a major systemic medical condition (e.g. diabetes); (2) having a comorbid diagnoses of Obsessive Compulsive Disorder, Post-traumatic Stress Disorder (PTSD), Bipolar Spectrum Disorder or a history of psychosis as determined by the diagnostic evaluation. |
| **Protocol** | SE-CPT: “Participants were told that they would be videotaped and that the videos would be used to examine their facial expressions. Children then sat next to a bucket of ice water (1–3 􀀁C) and were asked to stare into a video camera while putting their non-dominant hand in the water. A research assistant stared stoically and redirected the participants’ attention toward the camera if needed. If participants removed their hands before 10 s, they were instructed to place it back in the water until at least 30 s of immersion was completed. Anyone who kept the hand in the water for 3 min was asked to remove their hand from the water. Participants stared at the camera for 3 min regardless of total hand immersion time. The entire task takes approximately 5 min, which helps reduce individual variability in peak times that can add ‘‘noise’’ during statistical modeling (Lopez-Duran et al., 2014), while still activating the stress response (Schwabe et al., 2008; Schwabe & Wolf, 2010a,b). Following the SE-CPT, participants watched a calming documentary by National Geographic for 60 min in a separate room (e.g. The Appalachian Trail).”  Cortisol samples: “Eight samples were collected via passive drool at 30 min before the task (􀀂30), immediately before the task (0) and at 25, 35, 45, 55, 65 and 75 min after the start of the task.”  Ethical approval: obtained |
| **Outcomes** | **Primary** to probe different aspects of the HPA-axis response to psychosocial stress in youth depression with the goal of identifying specific areas of dysregulation and to probe age and sex effects |
|  | **Secondary** |
| **Results** | “Responders and non-responders did not differ in (…) sex distribution”  “Overall cortisol levels via repeated measures”: “There was no main effect of symptoms, age or sex (all p<0.20).” |

### Lu 2014 [47]

| **Methods** | **Design** Longitudinal study |
| --- | --- |
|  | **Setting** Xuzhou city, China |
|  | **Timing**  TSST-C |
| **Participants** | ***n=*** 87 |
|  | **Subjects**  Healthy and medication-free adolescents with a BMI between 17 and 32 kg/m 2 were recruited from interested 7th grade students at two urban public schools. |
|  | **Age** 12.7 years, SD 0.3 |
|  | **Sex** 48 boys, 39 girls |
|  | **Exclusion criteria** 6 girls were excluded because they were at their menstrual period, and 13 were removed because of deviated data in delay dis-counting measure as described in the delay discounting methods section.  Additional exclusion criteria for our study were: acute or chronic illness, medicine use, smoking, depression, using any medications, a history of eating disorders or current dieting. |
| **Protocol** | A total of nine saliva samples were collected during the TSST-C. [not specified at what time points]  Ethical approval: obtained |
| **Outcomes** | **Primary** The current study aims to investigate the relationship of cortisol reactivity, delay discounting and obesity in Chinese adolescents. |
|  | **Secondary** |
| **Results** | “No significant sex differences were observed except for weight, height and AUCi value (AUCi: F(1.85) = 2,15, p=0.03). [boys -0.28±7.42, girls -3.91±8.54, p=0.03]  “In boys, no significant correlation was found between AUCi and BMI (or PBF), and AUCi was no significant associated with AUCdd […] In girls, AUCi was positively related with PBF but negatively related to AUCdd.”  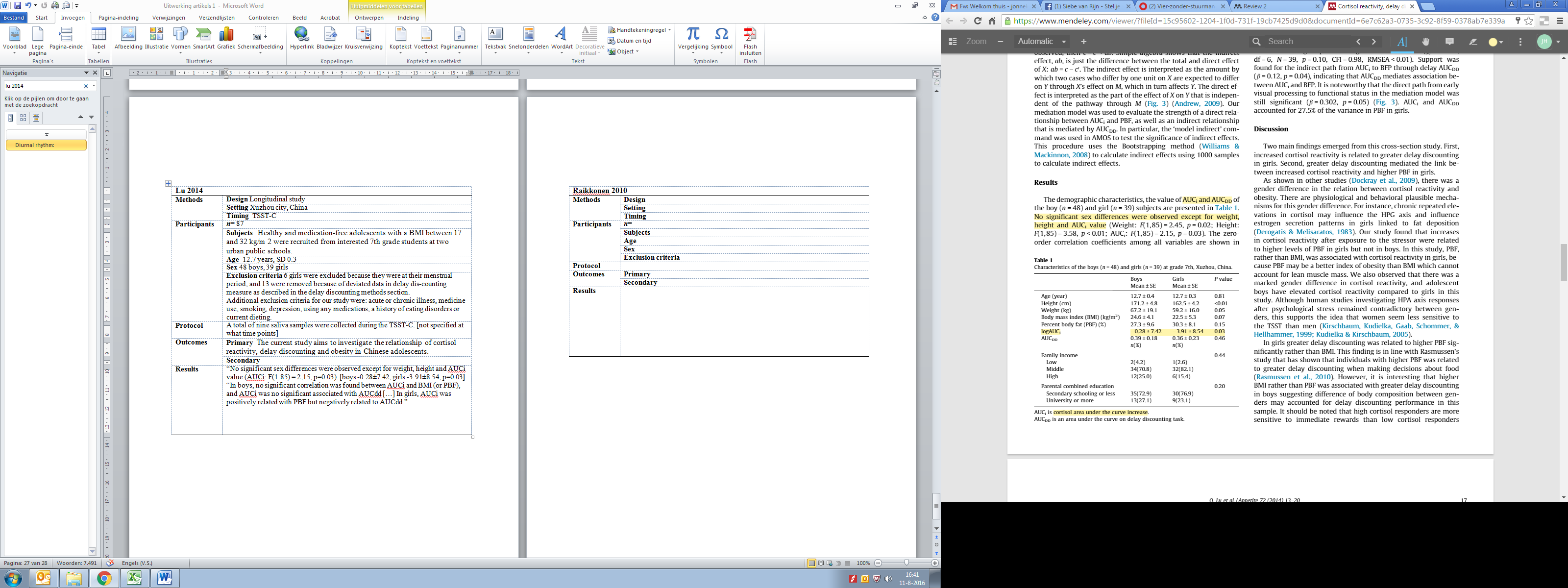 |

### Lumeng 2014 [48]

| **Methods** | **Design** Cross-sectional |
| --- | --- |
|  | **Setting** Ann Arbor, Michigan: |
|  | **Timing**  Children provided saliva samples 3 times per day (on arrival to preschool, before breakfast, about 8:30 am; before lunch, about 11:30 am; and at 4:30 pm) on 3 consecutive days |
| **Participants** | ***n=*** 331 |
|  | **Subjects**  Children attending Head Start, a free, federally-funded pre-school program for low-income children |
|  | **Age** 3-4 years |
|  | **Sex** 167 girls and 164 boys |
|  | **Exclusion criteria** parent with > 4 year college degree; parent or child not English-speaking; child in foster care, with food allergies, significant medical problems or perinatal complications, gestational age <35 weeks, or use of medication known or hypothesized to affect cortisol |
| **Protocol** | Children provided saliva samples 3 times per day (on arrival to preschool, before breakfast, about 8:30 am; before lunch, about 11:30 am; and at 4:30 pm) on 3 consecutive days by drooling in a tube or chewing on a piece of cotton. Daily logs included primary caregiver report of any med-ication use, illness, unusually good or bad events, exact time of morning awakening and if it was the usual time, napping or eating prior to the saliva sample; and location at the time of the afternoon sample. Saliva was stored at20° C until extracted and assayed in duplicate using an Expanded Range High Sensitivity Salivary Cortisol Enzyme Immunoassay Kit (Salimetrics LLC, PA, USA) with a detection limit of 0.007l g/dL and intra and inter-assay coefficients of variation of 7% respectively  Ethical approval: obtained |
| **Outcomes** | **Primary** The present study sought to test the conceptual model that exposure to psychosocial stress is associated with hypocortisolism, which is associated with specific eating behaviors that contribute to higher weight status among young low-income children |
|  | **Secondary** |
| **Results** | 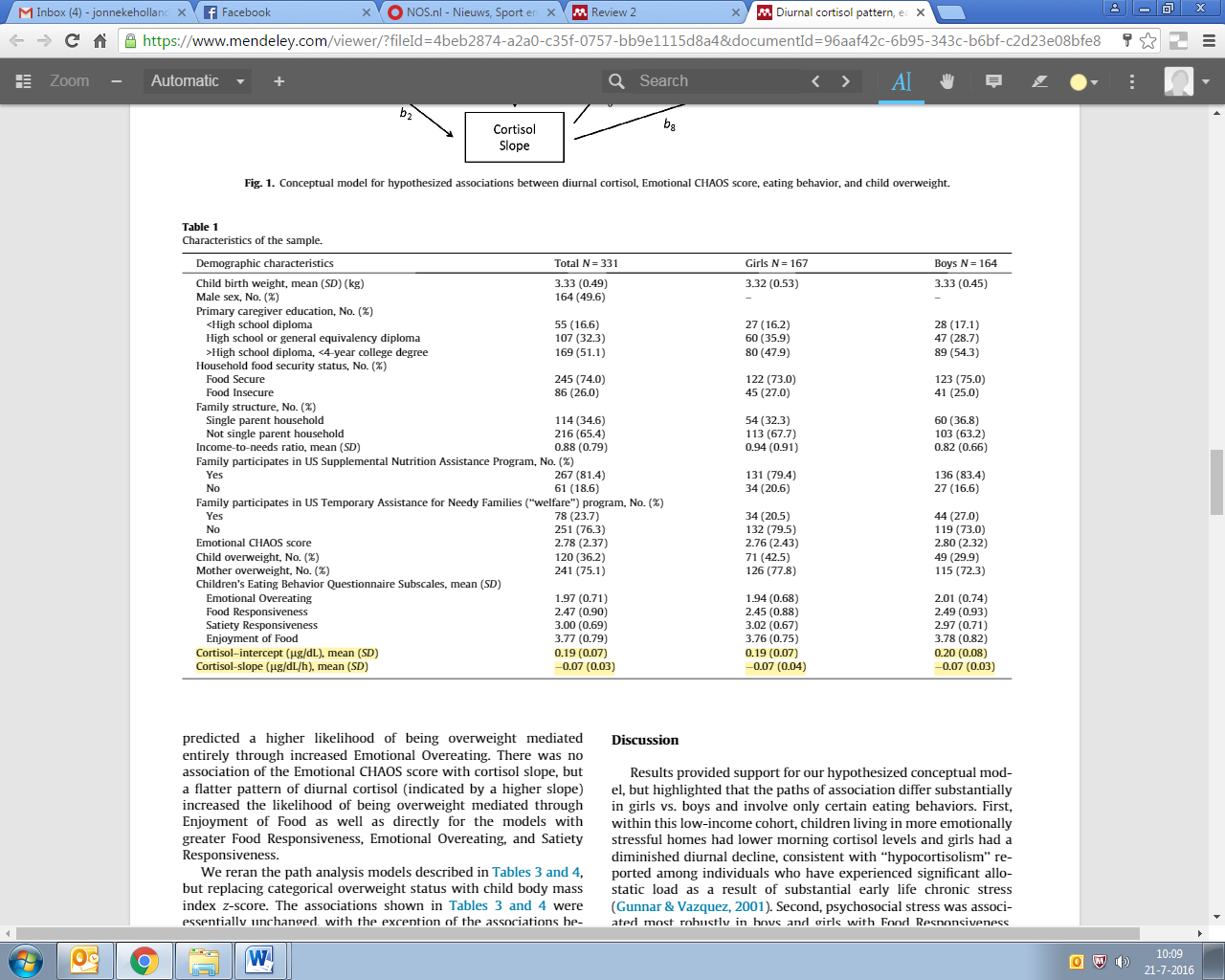  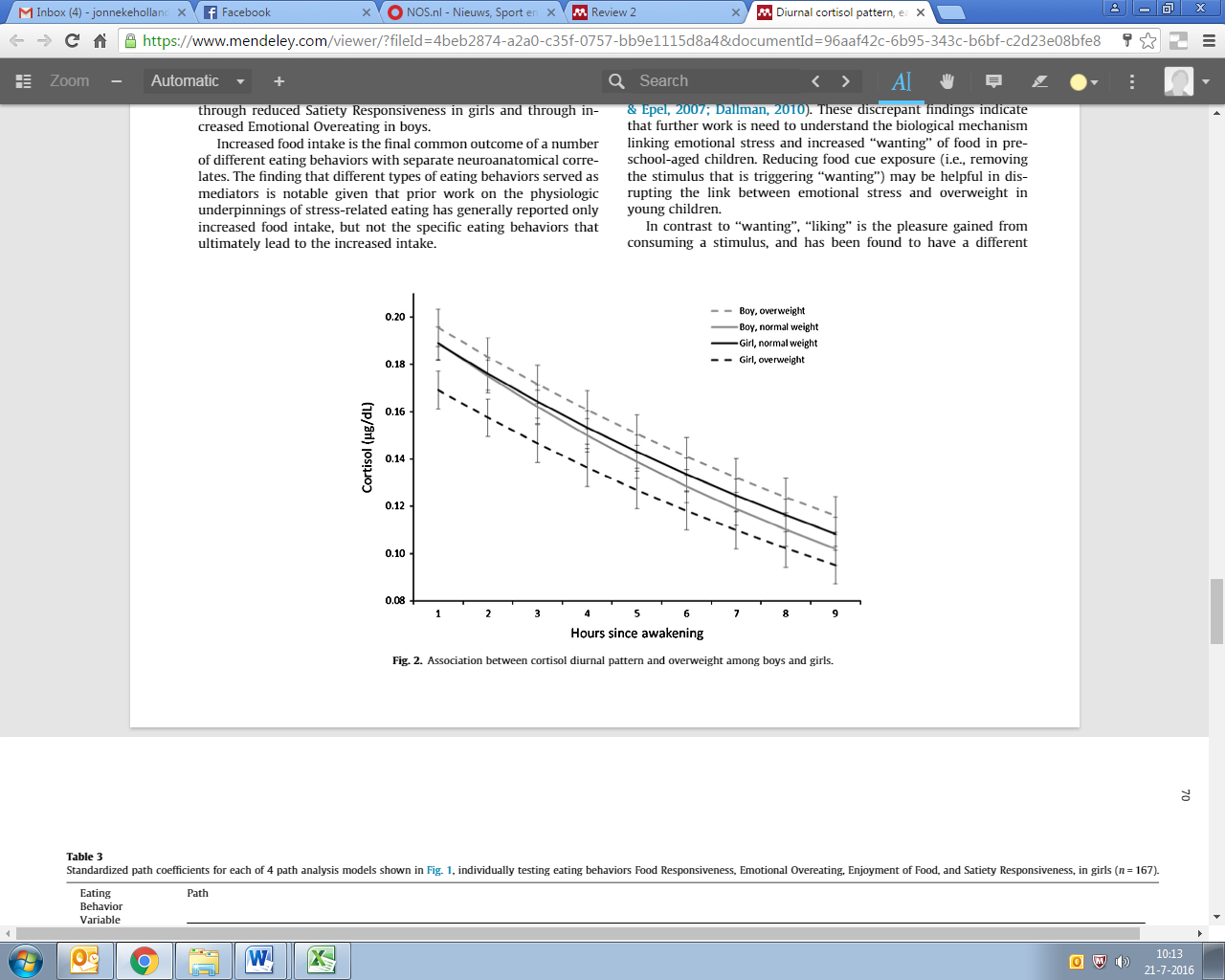🡪 no apparent difference  Among girls, the hypocortisolism pattern [lower cortisol with more chaotic home] predicted a higher likelihood of being overweight both directly and mediated through reduced satiety responsiveness. In boys, the association of the hypocortisolism pattern with being overweight was mediated entirely through emotional overeating |

| **Methods** | **Design** Urban, community based cohort (same as Raikkonen) |
| --- | --- |
|  | **Setting** Helsinki, Finland |
|  | **Timing**  Diurnal rhythm (including CAR) and TSST-C |
| **Participants** | ***n=*** 252 |
|  | **Subjects** Random, population-based sample |
|  | **Age** 8.1, SD 0.3 years |
|  | **Sex** 126 boys, 132 girls |
|  | **Exclusion criteria** Not specified for follow-up, additionally: developmental delay (n=3) or Asperger syndrome (n=1), no 4 days of valid PA data provided (n=54) |
| **Protocol** | Diurnal rhythm: obtained in 1 day, at awakening, 15 and 30 minutes thereafter and at 10:30, 12:00, 17:30 and bedtime.  TSST-C: Arrive at clinic at 12:00 or 14:00. Saliva samples were obtained at arrival and at baseline, and 0, 10, 20, 30 and 45 minutes after stress.  Ethical approval: obtained |
| **Outcomes** | **Primary** association between overall daytime PA and diurnal cortisol pattern |
|  | **Secondary** associations between overall daytime PA and cortisol during TSST-c + associations between vigorous PA and cortisol (diurnal and TSST-C) |
| **Results** | Boys displayed lower levels at awakening (P=0.02), peak after awakening (p=0.03) and lower AUC (p=0.02) of diurnal cortisol as well as lower AUC (p=0.004) and peak after stress (p=0.007)  (boys left, girls right)  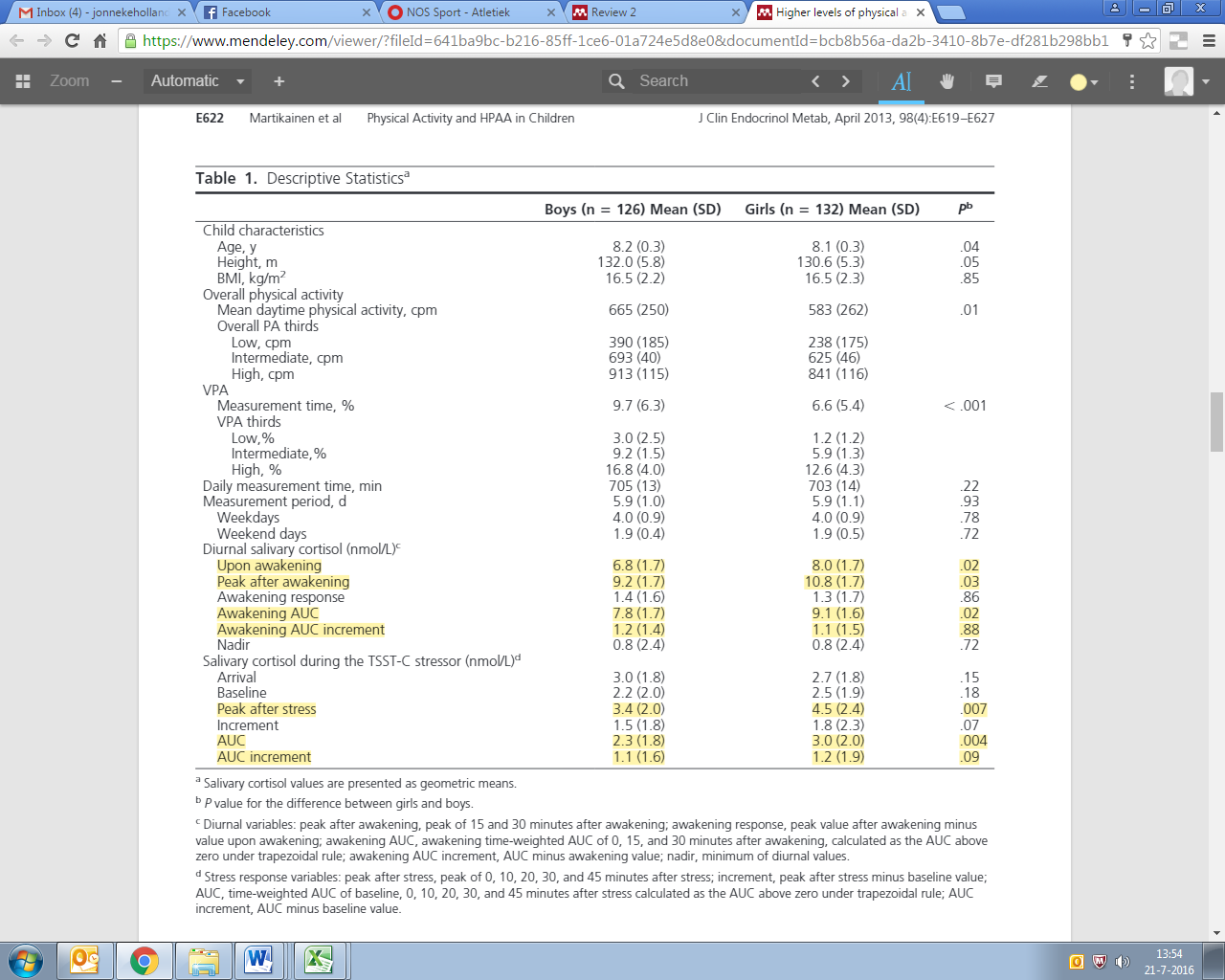 |

### Martikainen 2013 [49]

| **Martin 2011** | |
| --- | --- |
| **Methods** | **Design** Cross-sectional |
|  | **Setting** Not specified |
|  | **Timing**  TSST |
| **Participants** | ***n=*** 40 |
|  | **Subjects** “healthy adolescents without any medication or psychiatric disorders were recruited using ﬂyers.” “Subjects’ state of health was checked by a medical interview.” |
|  | **Age** 16-18 years |
|  | **Sex** 20 boys, 20 girls |
|  | **Exclusion criteria** “Pregnancy, lactation, intolerance to cosmetic products and former TSST participation were exclusion criteria for study participation.” |
| **Protocol** | TSST (not for children)  Cortisol samples: “Subjects provided saliva samples for cortisol determination 2 min before as well as 1, 10, 20, 30, 45 and 60 min after TSST”  Ethical approval: obtained |
| **Outcomes** | **Primary** to investigate stress-induced sweating in teenagers who are known to experience various stressful situations, e.g. exams at school or job interviews. |
|  | **Secondary** |
| **Results** | “No differences between female and male subjects were observed concerning cortisol concentrations (P = 0.77) pointing to comparable endocrine stress reactions to TSST between genders.”  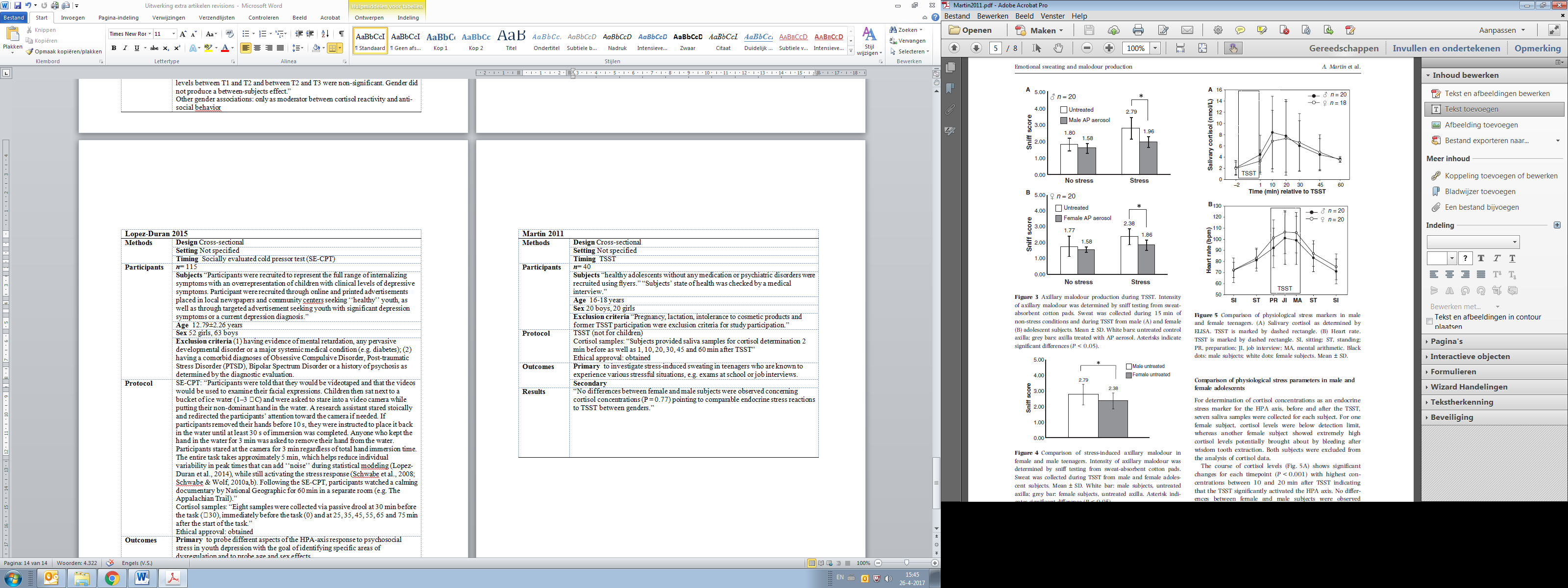 |

### Martin 2011 [50]

### Matchock 2007 [51]

| **Methods** | **Design** longitudinal study |
| --- | --- |
|  | **Setting** Pennsylvania, USA |
|  | **Timing**  Diurnal rhythm |
| **Participants** | ***n=*** 120 |
|  | **Subjects** “healthy children and adolescents who participated in a longitudinal study of puberty and behaviour” |
|  | **Age** In the first wave girls were aged 8, 10, or 12 yrs (n = 55, mean 10.49 yrs) and boys were aged 9, 11, or 13 years (n = 56, M = 11.44 yrs). The age difference was designed to include boys and girls at similar stages of pubertal development, as girls mature earlier than boys.  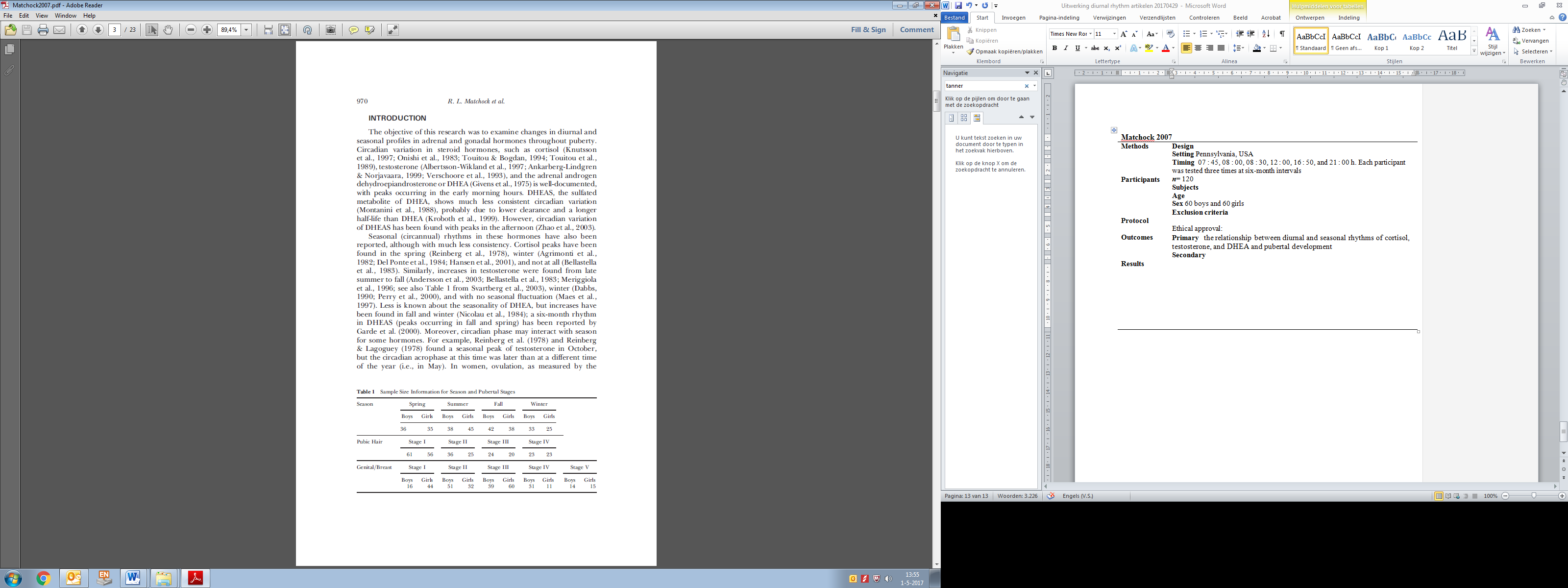”Pubertal stage was assessed by a Master’s prepared pediatric research nurse, using Tanner criterion of genital and pubic hair stage for boys and breast and pubic hair stage for girls (Marshall & Tanner, 1969, 1970). The nurse (…) conducted a physical exam for Tanner stage, which included breast palpation as recommended by Kaplowitz and Oberfield (1999). If the adolescent did not consent to the physical exam (n = 8), the adolescent’s self-rating of his/ her stage of pubertal development was substituted for the nurse rating.“ |
|  | **Sex** 60 boys and 60 girls |
|  | **Exclusion criteria** using medications that would interfere with hormone levels  (e.g., oral steroids); and presence of chronic health problems (e.g., diabetes  or cancer) or serious mental health problems. |
| **Protocol** | “Data were collected from three waves of measurement, each separated by approximately six months. (…) Data were collected during all four seasons of the year because of the staggered nature of the enrollment and occasions of  measurement (…) Day in the menstrual cycle was controlled, as the girls were always assessed between day 5 and 9 in the follicular phase of the menstrual cycle (…) Salivary samples were collected at home by the participants (…) sample 1 was obtained immediately on wakening, sample 2 at 20 min post-wake time, and sample 3 at 40 min post-wake time. Additional samples were collected at noon prior to the midday meal, 16 : 00 h., and at bedtime.”  Cortisol samples:“ mean sampling times for the group were 07 : 45, 08 : 00, 08 : 30, 12 : 00, 16 : 50, and 21 : 00 h”  Ethical approval: Obtained |
| **Outcomes** | **Primary** “to examine diurnal variations, including the daily peak in cortisol, testosterone, and DHEA” |
|  | **Secondary** “  2. to determine if seasonal rhythms exist for these three hormones;  3. to determine if concentrations of these hormones vary by sex and stage  of pubertal development; and  4. to examine cortisol, testosterone, and DHEA correlations across pubertal  development.” |
| **Results** | Peak time (acrophase):  The cortisol acrophase occurred later in the day for boys than for girls during later puberty. “For pubic hair and genital/breast stage 4, boys had a later and girls had an earlier cortisol acrophase compared to other stages (see Figure 2).”  Seasonal rhythms:  “For cortisol awakening secretory activity, a four (Season) x two (Sex) ANOVA found an effect of Season [F(3, 276) = 2.60, p < .05], but not for Sex or the interaction between Sex and Season.”  Pubertal maturation:  Morning cortisol levels were higher in boys at pubertal stage 2. “A four (pubic hair stage) x two (sex) ANOVA on awakening secretory activity indicated a significant sex x pubertal stage interaction, F(3, 238) = 2.59, p < .05, with morning cortisol levels in boys being higher than in girls in pubertal stage 2 (see Figure 4). However, for AUC data, a similar ANOVA indicated a significant effect of pubertal stage, F(3, 226) = 2.60, p <, .05, but no effect for sex or the interaction of sex x stage.”  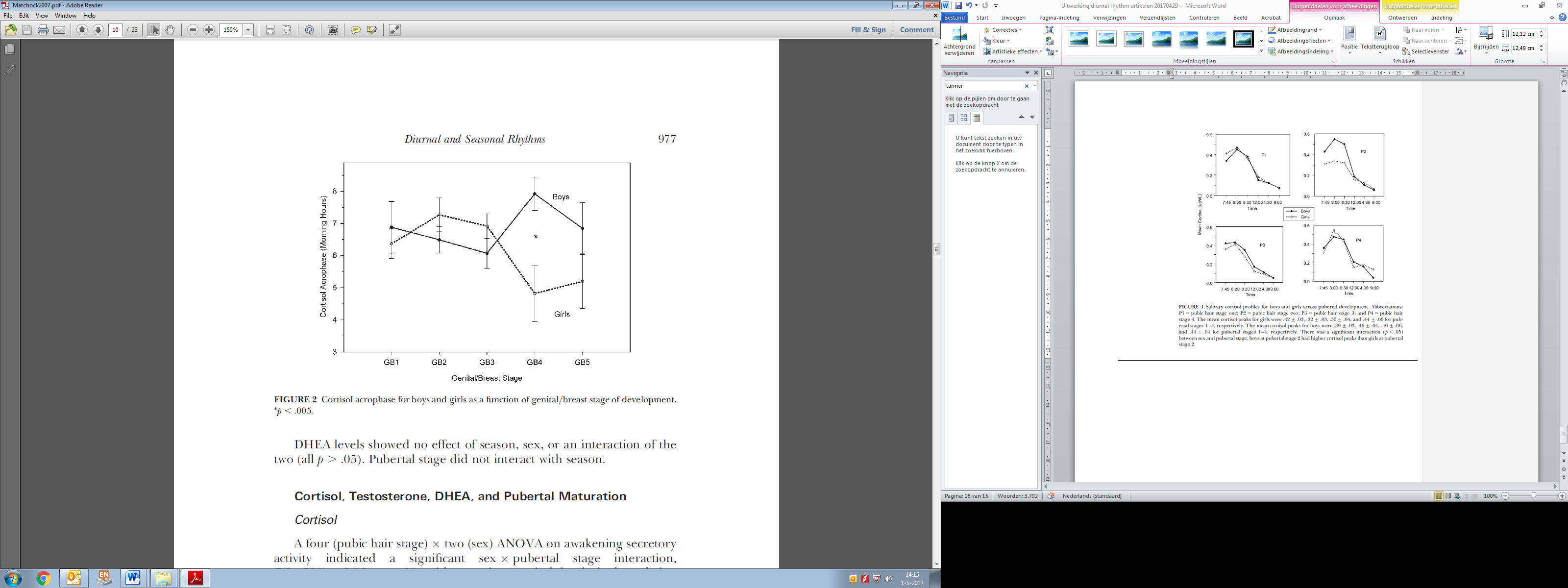  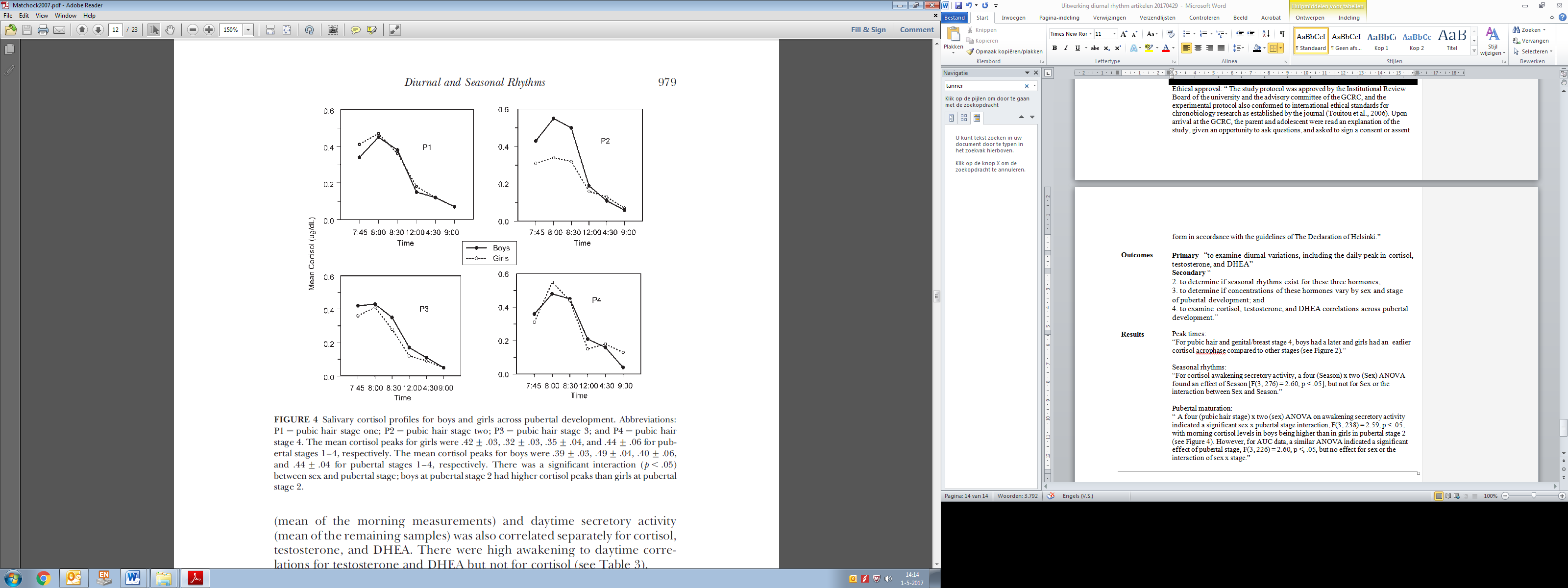 |

### Michels 2012 [52]

| **Methods** | **Design** Part of the Belgian control region of the IDEFICS study: selection by random cluster sampling |
| --- | --- |
|  | **Setting** Aalter, Belgium |
|  | **Timing**  Diurnal rhythm + CAR |
| **Participants** | ***n=*** 385 |
|  | **Subjects** Healthy children |
|  | **Age** 5-10 years old |
|  | **Sex** 183 boys, 202 girls |
|  | **Exclusion criteria** Not specified |
| **Protocol** | Saliva was collected during two consecutive weekdays at four time points: immediately after wake up, 30 min after wake up, 60 min after wake up and in the evening between 1900h and 2000h.  Morning samples collected more than 5 min different from the requested time point and evening samples not collected between 1900h and 2100h were excluded.  Ethical approval: obtained |
| **Outcomes** | **Primary** The response of cortisol patterns on challenges during children’s development, in a non-clinical, healthy population. This was done by studying the relationship of healthy children’s negative life events, emotions and difficulties with their salivary cortisol patterns. |
|  | **Secondary** Special focus is dedicated to sex differences in these preadolescent children  The role of positive emotions is considered. |
| **Results** | No significant differences between boy and girls were observed for salivary cortisol.  When using the multilevel model not stratified for sex, no sex difference was seen in overall mean cortisol, although **girls showed somewhat steeper diurnal slope** (p=0.30, 0.9% higher).  (more number in article, but not specified whether different between boys and girls.  Also, associations with other factors are looked at)  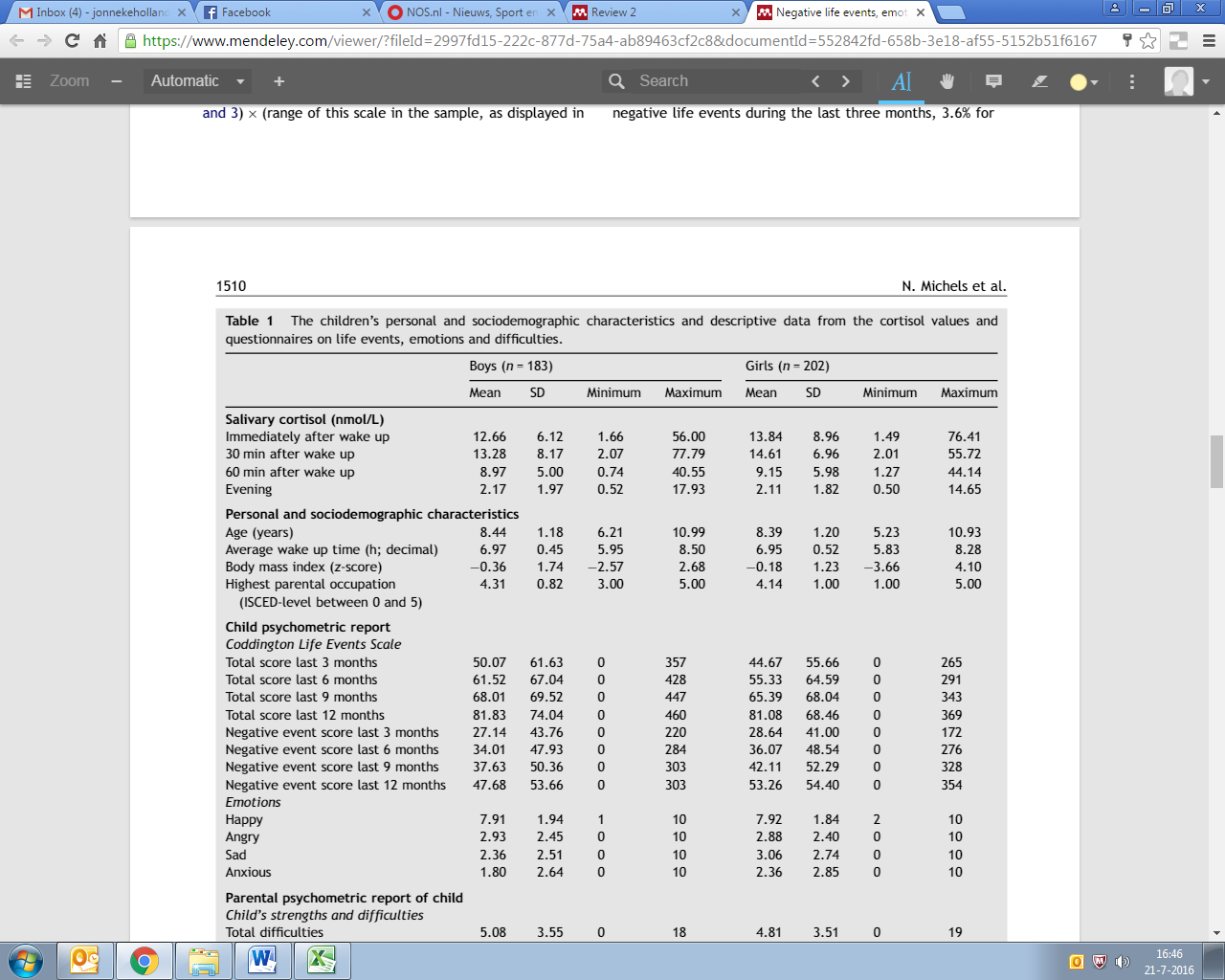 |

### Mills 2008 [53]

| **Methods** | **Design** cross-sectional |
| --- | --- |
|  | **Setting** “An urban area (population 600,000) |
|  | **Timing**  An assessment of the child’s shame responding involving observations of children’s emotion-expressive behaviour in response to failure on six performance tasks used in previous work. |
| **Participants** | ***n=*** 214 |
|  | **Subjects** Recruited through a letter of invitation under a cover letter sent by a government agency responsible for administering health care. |
|  | **Age** 3.7 to 4.5 years (mean: 4.14, SD 0.24) |
|  | **Sex** 124 boys, 90 girls |
|  | **Exclusion criteria** Not specified |
| **Protocol** | 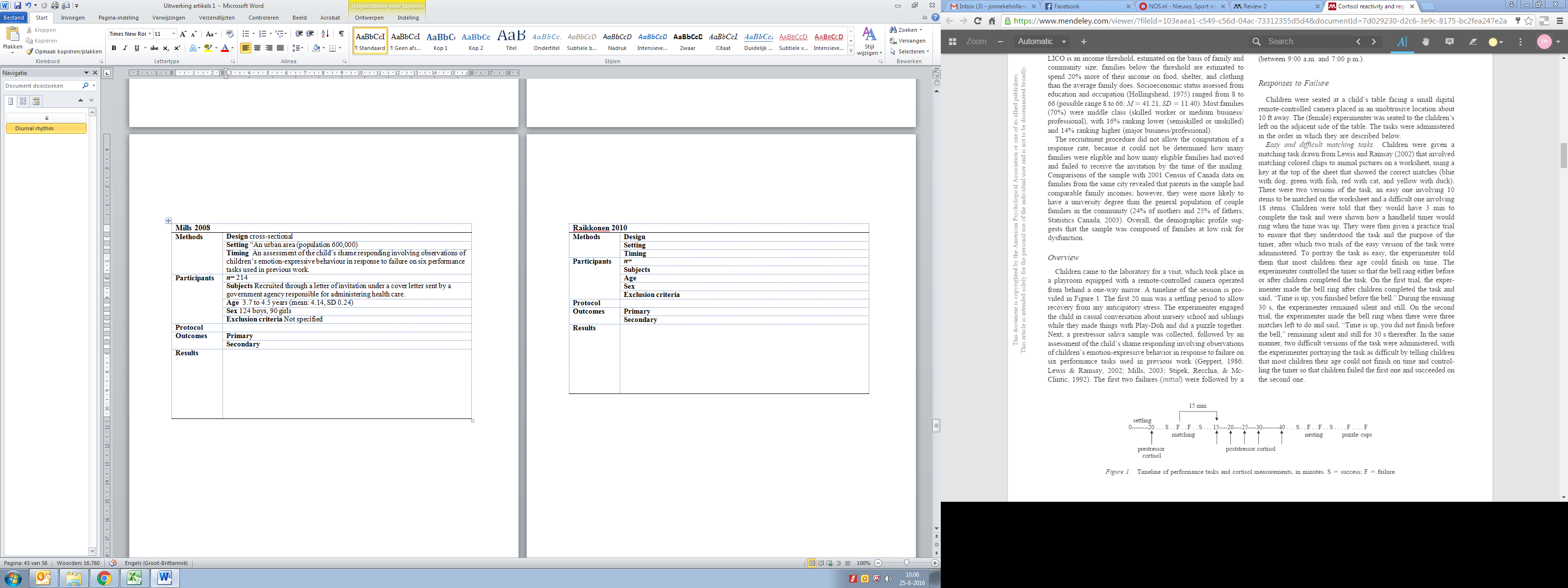  Tasks: easy and difficult matching tasks, easy and difficult competitive nesting tasks, impossible puzzle and nesting cups  Cortisol collection: “We collected salivary cortisol samples before and after the two initial failures (easy and difficult matching tasks): one prestressor sample at the end of the settling period and five poststressor samples taken 15, 20, 25, 30 and 40 min after failure on the easy matching tasks. The samples were timed with reference to the moment of failure.  Ethical approval: obtained |
| **Outcomes** | **Primary** To characterize shame-related cortisol activation in early childhood on the dimensions of reactivity (peak amplitude) and regulation (return to baseline). |
|  | **Secondary** To examine how general the relation was between shame and cortisol |
| **Results** | An analysis of covariance on prestressor cortisol by gender with lab arrival time as the covariate revealed no significant difference between girls and boys in prestressor cortisol. On average, raw cortisol levels declined from the prestressor to poststressor time points, with Wilcoxon signed ranks tests indicating that levels declined significantly from the prestressor to the 15-min poststressor time point both for boys, z=6.11, p<0.001, and girls, z=5.39, p<0.001. For girls, there were no significant further declines in cortisol. For boys, levels did not differ between the 15- and 20-min poststressor time points, z=0.34, ns; declined from the 20- to 25-min poststressor time point, z=2.63, P<0.01; did not differ between the 25- and 30-min poststressor time points, z=0.77, ns; and declined from the 30- to the 40-min poststressor time points, z=2.69, p<0.01.  Children showing an increase in cortisol levels were more likely to be boys (n=43, 64%), than girls (n=24, 36%), p<0.03), whereas those showing a decrease were equally likely to be boys (n=72, 54%) r girls (n=61, 46%), p=0.39. (…) No difference in the proportions of boys and girls showing their peak at the 150min (33% of boys, 36% of girls) or the 20-min (20% of boys, 14% of girls) poststressor time points. (…) There were no gender differences in the measures od cortisol reactivity or regulation.  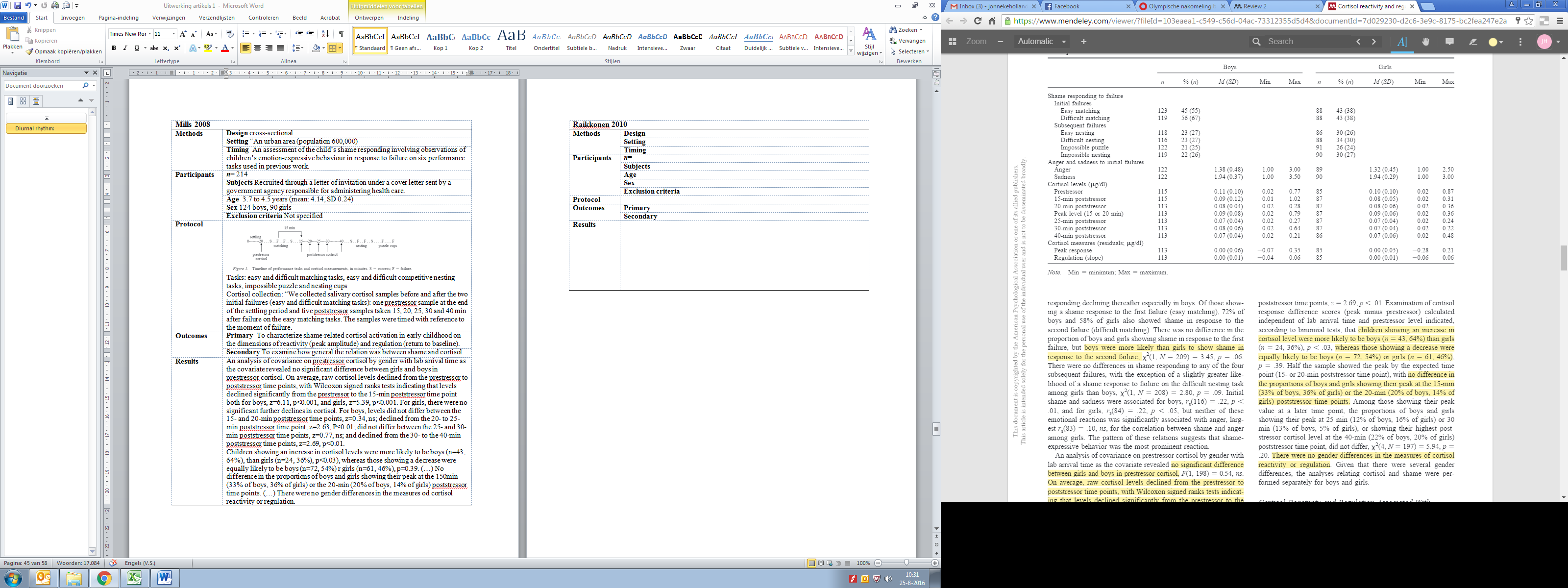 |

### Minkley 2012 [54]

| **Methods** | **Design** Cross-sectional |
| --- | --- |
|  | **Setting** Bochum, Germany |
|  | **Timing**  Two tests: one “reproduction of knowledge”, the other “transfer and problem-solving”, two controls (one group could write about anything, the other had to sit quietly) |
| **Participants** | ***n=*** 93 |
|  | **Subjects** Students of biology courses at secondary or comprehensive schools |
|  | **Age** 17.86 years, SEM: 0.096 |
|  | **Sex** 56 male, 37 females |
|  | **Exclusion criteria** Smoking, over or underweight, use of any long-term medication or oral contraceptives, or presence of a medical condition. |
| **Protocol** | “In this study the participants were randomly assigned to one of the four different treatment groups: the first group was asked to deal with tasks associated with the reproduction of knowledge (hereafter referred to as “reproduction”, e.g., “describe the eight steps of our DNA-extraction protocol”), while students assigned to the second group were asked to deal with tasks associated with transfer and problem-solving (hereafter referred to as “transfer”, e.g., “present a possibility to shorten the DNA-extraction protocol”). All tasks dealt directly with processing the information that the students had recently learned during the previous 2 h at the laboratory, in order to prevent the effects of different previous knowledge. The third group served as the control for the writing activity (hereafter referred to as “control”). Students in this group were free to write anything 9only 5 suggestions were given, for instance, they were asked to write about their hobbies), while their course mates took the tests. The fourth group also served as a control. In this group (“no test”) the students were required to sit quietly without anticipating a test situation. Thus, they were informed about their purpose before any of the participants know that the stressor will be a short written test.” And “all students had to complete the test within a time frame of 10 min.”  Saliva collection: before the test, as soon as possible after the end of the test (+/- 15 min after start test).  Ethical approval: obtained |
| **Outcomes** | **Primary** to investigate whether these different cognitive demands [reproduction, transfer] influence cortisol secretion to different degrees |
|  | **Secondary** |
| **Results** | Repeated measurement ANOVA: sex = not significant  “Post hoc Hochberg’s GT2 test indicated that the cortisol increase was significantly higher during the test associated with reproduction tasks than under either control condition (p<0.05). However, the cortisol increase occurring during transfer and problem-solving tasks was not significantly different compared to the control conditions. The independent variable SEX had no significant effect on the cortisol change (F(1,85) = 1.995, P>0.05, n2=0.023). However, male students showed a higher cortisol increase compared to female students when dealing with reproduction or transfer and problem-solving tasks.”  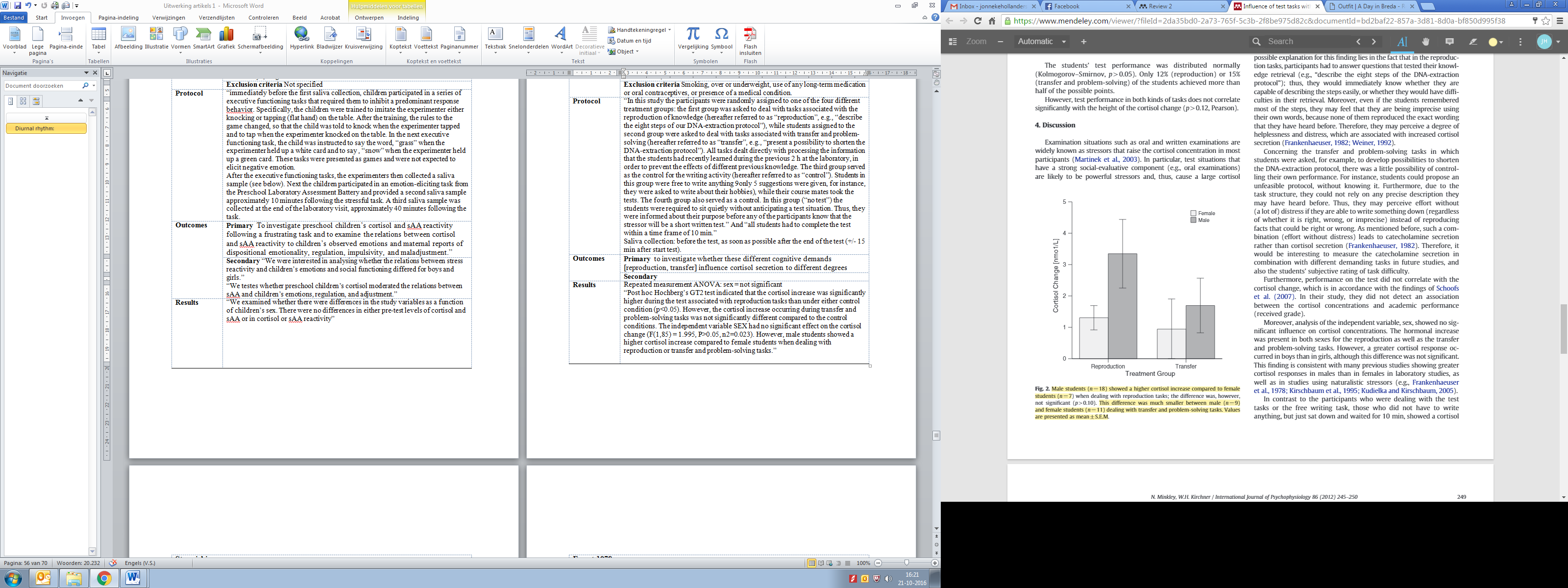 |

### Morin-Major 2016 [55]

| **Methods** | **Design** Cross-sectional study |
| --- | --- |
|  | **Setting** Montreal, Canada |
|  | **Timing**  Diurnal rhythm + CAR |
| **Participants** | ***n=*** 88 |
|  | **Subjects “**recruited from a larger study on family and stress conducted by the Centre for Studies on Human Stress (Montreal, Quebec, Canada). Of the 333 participants of the large study that included parents, children below the age of 12, and adolescents, we recruited the totality of adolescents for the current FB study.” |
|  | **Age** 14.5 ± 1.8 yrs (boys:14.9 ± 1.8; girls; 14.2 ± 1.7) |
|  | **Sex** 41 boys and 47 girls |
|  | **Exclusion criteria** medication that may affect depressive symptoms or cortisol levels (e.g., anti-asthma medication, anxiolytics) or any other psychiatric, neurological, substance use, or general health condition or when a participant is not active on Facebook, did not provide enough saliva or when cortisol levels were considered to be an outlier ( more than three SD above or below the mean). |
| **Protocol** | “To assess diurnal cortisol levels, saliva samples were taken four times a day on two separate days (…) To facilitate sampling and reduce error, each tube cap was color coded in accordance with time of day (…) Given that cortisol was measured on two different weekdays, a mean was computed for each time point. In the case of an individual outlier, the mean was replaced by the non-outlier variable.”  Cortisol sampling: “(1) at awakening, (2) 30 min following awakening, (3) before dinner, and (4) before going to bed on two non-consecutive weekdays (…) during a three week timeframe.”  Ethical approval: obtained |
| **Outcomes** | **Primary** “associations between Facebook behaviors (use frequency, network size, self-presentation and peer-interaction) and basal levels of cortisol among adolescent boys and girls.” |
|  | **Secondary** N/A |
| **Results** | “Table 1 reports the descriptive statistics and correlation matrix for all study variables used for exploratory purposes. Girls showed positive associations with perceived stress, FB use frequency, FB prosocial behaviors, and cortisol systemic output (AUCg) (…) Table 2 reports all regression coefficient information. Model1 with covariates entered was significant (F(3,64)= 7.81, p < .001,R2= 0.268): cortisol systemic output was positively associated with being a girl and older age, while negatively associated with awakening time.”  From table 1: CAR also appears to be correlated to sex, greater in girls  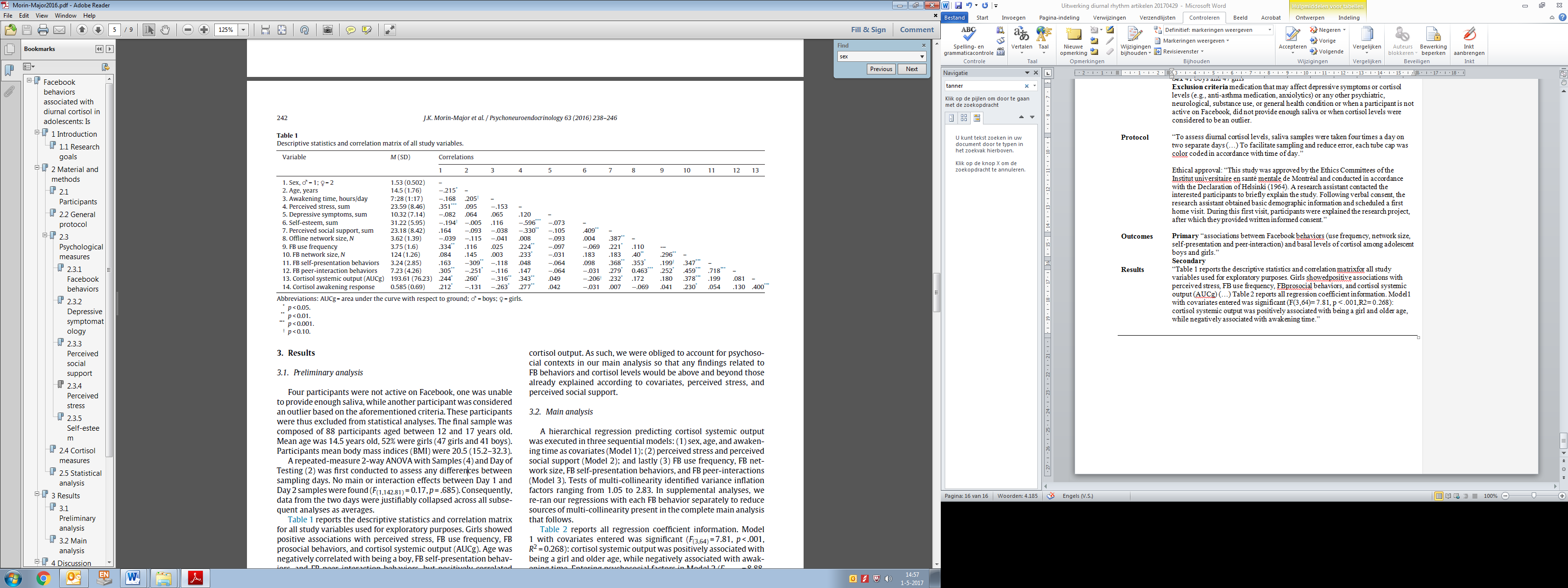  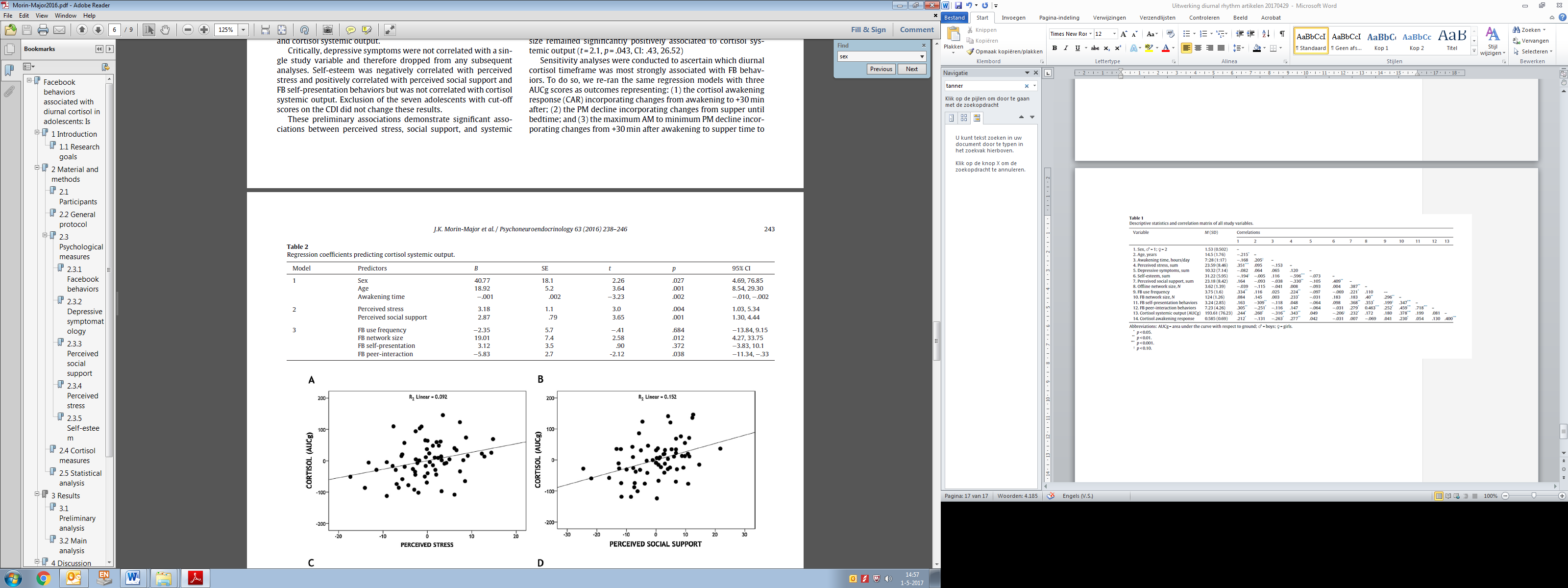 |

### Mrug 2016 [56]

| **Methods** | **Design** Cross-sectional |
| --- | --- |
|  | **Setting** Birmingham, AL, USA |
|  | **Timing**  TSST-C |
| **Participants** | ***n=*** 84 |
|  | **Subjects**  Participants were adolescents who took part in the Coping with Violence Study. The sample was socioeconomically heterogeneous, but comprised primarily low-income families; average annual family income was $20,000–$25,000 (range $5000 to $70,000– $90,000) and average parental education was some college but no degree (see Table 1 for sample characteristics). The adolescents were recruited from four public middle schools (grades 6– 8 or 9) serving low income, urban communities in Birmingham, AL. Across the four schools, 83% to 87% of students were eligible for free or reduced price lunch. |
|  | **Age** M age 13.36 years, SD = 0.95 |
|  | **Sex** 50% male (so 42/42) |
|  | **Exclusion criteria** None of the adolescents were diagnosed with any psychiatric disorders (per parent report) or were taking any medications that would affect cortisol levels (per self-report) |
| **Protocol** | Whole saliva samples were collected by passive drool immediately before the TSST (pre-test), 30 min after the 15-min test began (15 min post-test), and 70 min after the test began (55 min post-test).  Ethical approval: obtained |
| **Outcomes** | **Primary** To examine the effects of sleep on HPA axis activity in adolescence. |
|  | **Secondary** |
| **Results** | Females had higher cortisol at 55 min post-test and greater AUCi. [no absolute cortisol values]  “significant gender differences emerged for child-reported sleep variables predicting cortisol levels 15-min post-test, as well as AUCG and AUCI (all p< .005). In each case, there was a significant effect of sleep problems on elevated cortisol levels for girls, but not for boys. Specifically, sleep problems uniquely predicted girls' 15-min post-test cortisol (β=.15,pb.01), AUCG(β=.28,pb .05), and AUCI(β= .31,pb.01), whereas these effects were not significant for boys (β= .20,p= .07 for 15-min post-test cortisol;β= .19,p = .22 for AUCG; and β=.21,p=.08forAUCI ).” |

### Netherton 2004 [57]

| **Methods** | **Design** cross-sectional study |
| --- | --- |
|  | **Setting** Cambridge, United Kingdom |
|  | **Timing** Diurnal rhythm |
| **Participants** | ***n=*** 129 |
|  | **Subjects** “younger siblings of participants from a previous community study (Goodyer et al., 2000a), and (…) students from two Cambridgeshire schools (…) reported to be currently healthy and without severe learning difficulties “ |
|  | **Age** 12.8 ± 0.19 yrs 30.1% (37; 22 boys, 15 girls) were pre-early puberty and 69.9% (86; 36 boys, 50 girls) were mid-post puberty. |
|  | **Sex** 60 boys and 69 girls |
|  | **Exclusion criteria** “current emotional problems requiring treatment, chronic  physical illness, and severe learning difficulties requiring special assistance.” |
| **Protocol** | “Subjects provided samples of saliva in the morning and evening (0800 h and  2000 h) over four consecutive days (…) during a normal school week (...) The mean level of cortisol at each time point (0800 h and 2000 h) was calculated  from the values obtained from each subject’s individual samples.”  “Pubertal stage was assessed using Tanner stage sketches (…) subjects were provided with gender-appropriate sketches, and were asked to select which of the sketches “looked most like them”. Using the same format, a parent (usually the mother) was also asked to make an independent rating of their child’s current stage of pubertal development (…)  Based on the average of self and parent ratings, subjects were classified as Tanner stage I–V. Using this average rating, subjects were grouped according to their general status of pubertal development: pre to early puberty (PEP, Tanner stage<III) or mid to postpuberty (MPP, Tanner stage>II).”  Ethical approval: obtained |
| **Outcomes** | **Primary** “basal levels of cortisol and dehydroepiandrosterone (DHEA), and their relation to gender and pubertal development” |
|  | **Secondary** N/A |
| **Results** | “Post hoc analysis revealed that MPP girls had significantly higher morning levels than MPP boys (F(3,116)=3.86, p=0.01, Bonferroni; Fig. 1A) (…) Analysis of individual variability in salivary cortisol levels showed no effect of either gender (0800 h z=–1.55, p:n.s., 2000 h z=–0.12, p:n.s., Mann–Whitney) or pubertal status (0800 h z=–0.98, p:n.s., 2000 h z=–1.28, p:n.s., Mann–Whitney) on the variance (SD) across the four days of saliva sample collection.”  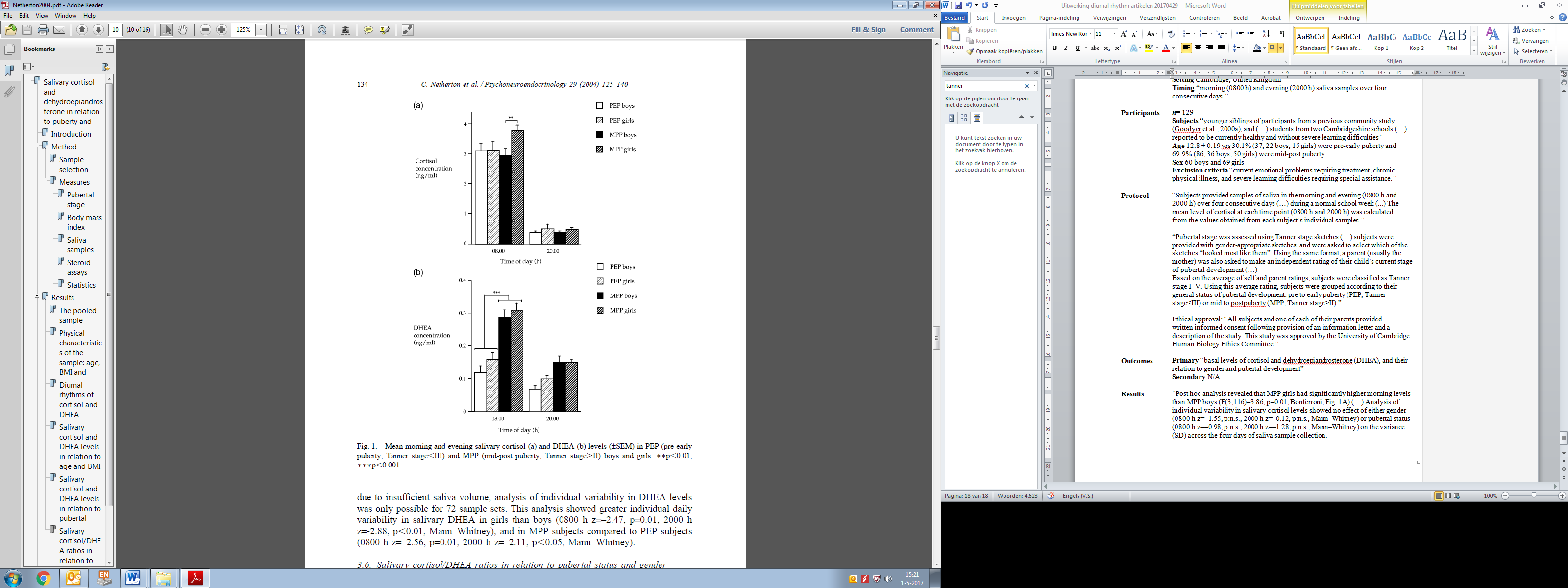 |

| **Methods** | **Design** Cross-sectional |
| --- | --- |
|  | **Setting** “Small rural towns” in Sweden |
|  | **Timing**  Diurnal rhythm + CAR |
| **Participants** | ***n=*** 84 (55 completed collection of all saliva samples) |
|  | **Subjects**  children from eleven third and fourth grade classes in four primary schools in small rural towns |
|  | **Age** 9–11 years, mean 9.9 years,± 0.55 years |
|  | **Sex** 50 girls and 34 boys |
|  | **Exclusion criteria** Not specified |
| **Protocol** | The first sample (SC1) was collected immediately upon waking in the morning, irrespective of time. The next sample (SC2) was collected 15 min later, before eating or brushing teeth. The quotient between these two values (SC1/SC2) provided a measure of the cortisol waking response (Pruessner, Kirschbaum & Hellhammer, 1995). The third sample (SC3) was obtained at 09.00 hours at school (9 a.m.). This time was chosen as we wanted to compare our material with data from a study by Tornhage (Tornhage, 2003) conducted among schoolchildren between 9 and 11 years of age. The fourth sample (SC4) was collected approximately 15 min before lunch at 11.00 hours (11 a.m.). The fifth and final sample (SC5) was collected at bedtime, after 15 min at rest in bed, before falling asleep.  Ethical approval: obtained |
| **Outcomes** | **Primary** our aim was to assess the magnitude of stress in children of both sexes, and to validate this questionnaire against established psychometric measures for closely related states such as depression, anxiety, anger, disruptive behavior, and self-perception |
|  | **Secondary** We also examined the relationships with the questionnaire data for biological markers of stress, salivary cortisol, and urinary cathecholamines, both of which have been associated with demands and self-reported stress |
| **Results** | no significant differences were found between the sexes  we found statistically significant associations between the SiC questionnaire scores and SC1 (r=0.477, r2 = 0.228, p=0.001), SC2 (r=0.313, r2=0.098, p = 0.038), and with the “area under the curve” (AUC) (r=0.330, r2=0.109, p= 0.049) among girls, but no statistically significant associations were observed among boys |

### Osika 2007 [58]

| **Methods** | **Design** Longitudinal (first and second wave of assessment) |
| --- | --- |
|  | **Setting** “from a small city and rural communities”, presumably somewhere in America |
|  | **Timing**  TSST-C |
| **Participants** | ***n=*** 124 |
|  | **Subjects**  The sample was recruited using the American Student List, a database of school-aged children, selected from designated zip codes. Randomly selected parents of children on the list were initially contacted by mail.  Children were eligible to participate if they met the following criteria: boys and girls were 9, 11 or 13, and 8, 10, or 12 years of age, respectively; no current chronic health of severe mental health problems as assessed by a screening questionnaire completed by the parent or guardian that would limit the child’s ability to sit through the interview, such as physically handicapped children with gross motor or speech problems or mentally challenged children; and not currently taking medication that could influence hormone levels (e.g., corticosteroids). |
|  | **Age** 10.49 years, SD 1.68 |
|  | **Sex** 60 boys, 64 girls |
|  | **Exclusion criteria** As specified in “subjects” + no experience with a child protective service agency. |
| **Protocol** | The samples were collected 20 and 5 minutes before the TSST-C, and 0, 10, and 20 minutes after TSST-C by passively drooling into a 5-ml tube.  Ethical approval: obtained |
| **Outcomes** | **Primary** to examine the effect of exposure to violence (ETV) on cortisol reactivity (CR) in children with no identified serious mental health problems or reports of maltreatment. |
|  | **Secondary** |
| **Results** | "Mean ETV and CR did not differ significantly between boys and girls at Time 1 or Time 2, and CR was significantly greater at Time 2 in boys and girls (Table1)." AND related to other factors: "ETV occurring during the 12 months before the first assessment was predictive of CR 12 months later in boys"  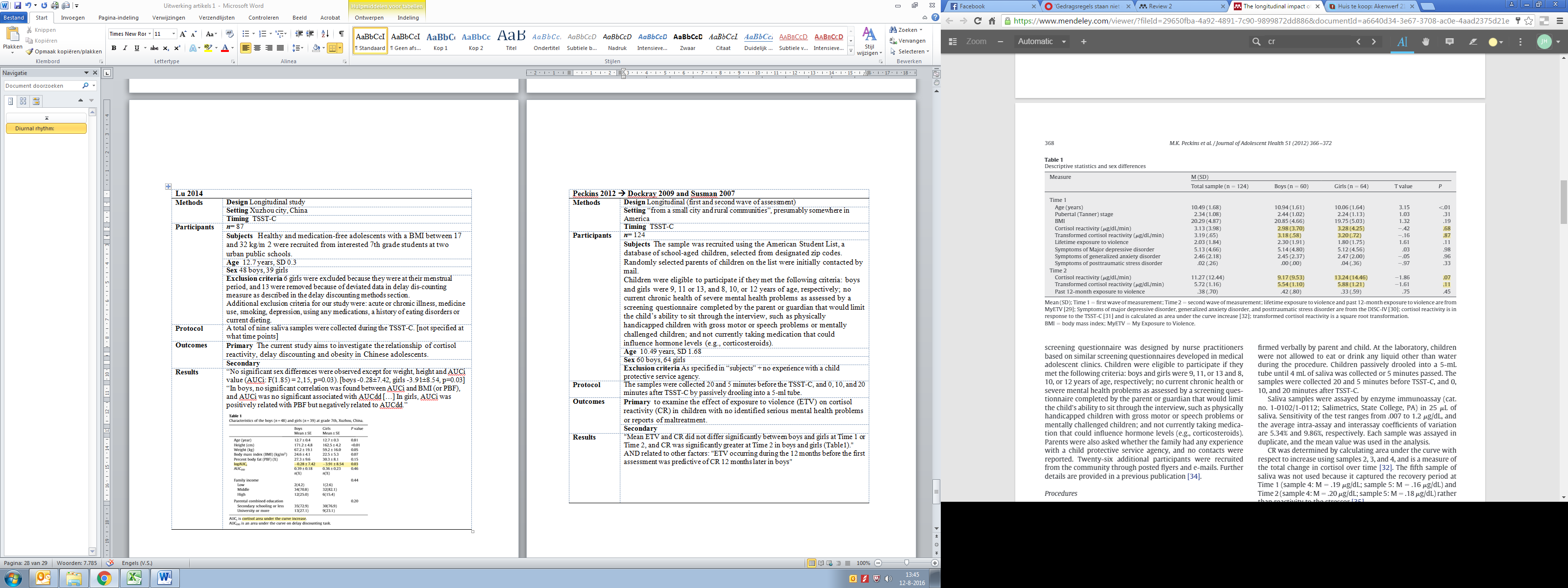 |

### Peckins 2012 [59]

| **Methods** | **Design** Longitudinal (this study: cross-sectional) |
| --- | --- |
|  | **Setting** Greater Montreal area (Canada) |
|  | **Timing**  In the first situation, one twin and the mother were alone in a corner of a room when a woman dressed as a clown entered the room, went to the opposite corner, and invited the child to approach by offering a set of familiar toys. In the second situation, a noisy, odd-looking, moving toy robot was placed on a platform in the opposite corner of the room. |
| **Participants** | ***n=*** 376 |
|  | **Subjects**  Toddlers from the Quebec Newborn Twin Study. |
|  | **Age** 18.85 months, SD 0.74 |
|  | **Sex** Not specified |
|  | **Exclusion criteria** Not specified |
| **Protocol** | As illustrated in fig. 1, saliva was collected prior to and following the participation in two unfamiliar situations known to be moderately stressful at that age. In the first situation, one twin and the mother were alone in a corner of a room when a woman dressed as a clown entered the room, went to the opposite corner, and invited the child to approach by offering a set of familiar toys. In the second situation, a noisy, odd-looking, moving toy robot was placed on a platform in the opposite corner of the room. Each session lasted 280 seconds separated by 5 minutes of mother-child free play. As shown in fig 1, the pre-test sample was obtained at the arrival at the lab. The post-test sample was obtained 20 minutes after the end of the procedure in order to capture the peak cortisol response.  Ethical approval: informed consent was obtained from the parents |
| **Outcomes** | **Primary** To determine whether low GCs levels in 19-month old toddlers are associated with higher social dominance in a competitive resource situation. |
|  | **Secondary** We also assessed potential sex differences on the association between GCs and social dominance in this cohort of young children. |
| **Results** | “No sex differences were observed on pre-test cortisol levels (z=-0.69, P=0.49)”  “However, when assessing this relationship in boys and girls separately, we found that in boys increased pre-test cortisol levels were significantly associated with decreased proportion of time the boys controlled the resource/got the toy. This association was not found in girls.”  “The time of day when saliva was collected was not associated with the reactive cortisol ratio (…) and no gender differences were observed on the ratio (Z=0.63, P=0.53). Over the entire group fo children, no significant associations were found between the reactive cortisol ratio and the proportion of time the child got the toy. No significant associations were found between reactive cortisol ratio and behavioral outcomes even when analysis was split by sexes.”  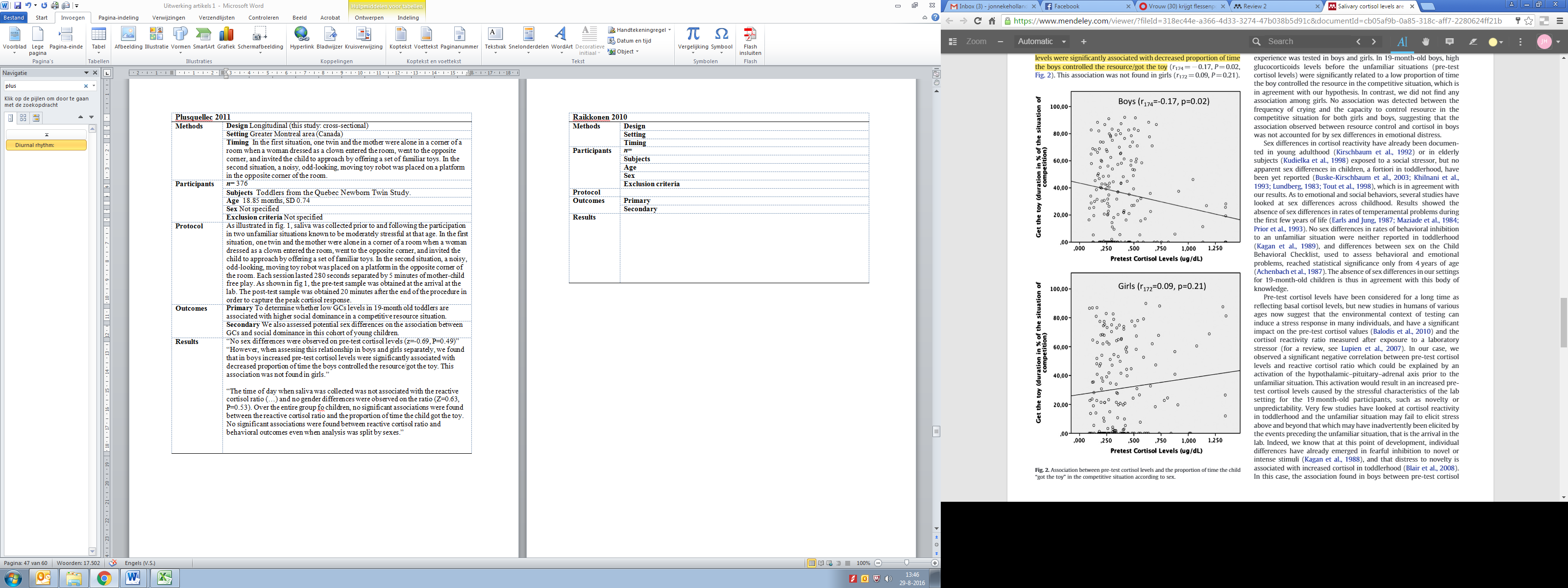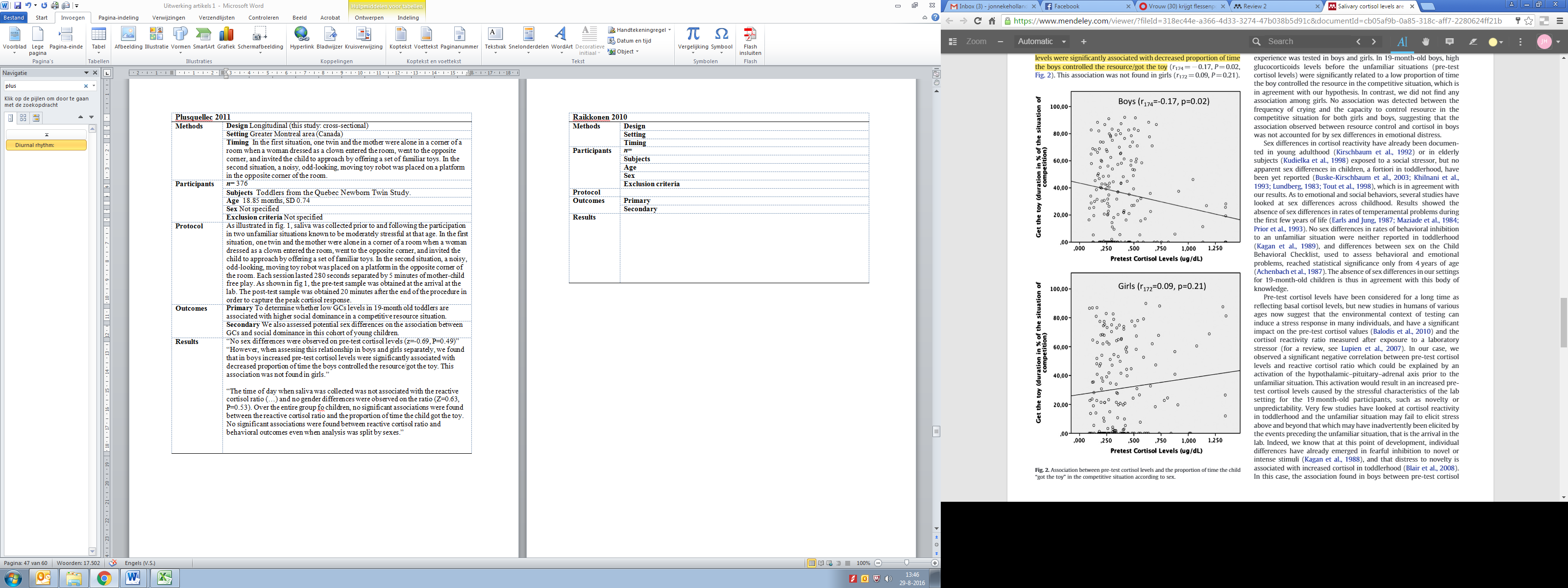 |

### Plusquellec 2011 [60]

### Portnoy 2015 [61]

| **Methods** | **Design** Cross-sectional |
| --- | --- |
|  | **Setting** Philadelphia County, or suburbs of Philadelphia (USA) |
|  | **Timing**  Morning sample and TSST-C |
| **Participants** | ***n=*** 446 |
|  | **Subjects**  Within the study area, fliers soliciting enrollment were placed in recreation centers, libraries, health clinics, and other community centers. Targeted mailings were also sent to parents of 11–12 year old children living in the geographic catchment area. […] 14.2% of subjects had a lifetime diagnosis of conduct disorder and 19.1% had a lifetime diagnosis of oppositional defiant disorder |
|  | **Age** 11.92 years (SD = .59) |
|  | **Sex** 50.6% male |
|  | **Exclusion criteria** Youths with a diagnosed psychotic disorder, mental retardation, or a pervasive developmental disorder were excluded |
| **Protocol** | A morning saliva sample was collected at an average time of 9:18 AM. Between sample collections, subjects completed behavioral questionnaires. In the afternoon, four saliva samples were collected to assess cortisol reactivity to the stressor at the following times: (1) Immediately prior to the laboratory tasks (mean time = 12:36 PM), (2) 5 min after the end of the stress task (mean time = 1:27 PM), (3) 20 min after the end of the stress task (mean time = 1:42 PM), and (4) 40 min after the end of the stress task (mean time = 2:02 PM)  Ethical approval: obtained (Liu, Int J Method Psychiatr Res, 2013) |
| **Outcomes** | **Primary** The purpose of this article is to examine whether 2D:4D interacts with cortisol and adolescent testosterone level to predict externalizing behavior in a sample of young adolescents. |
|  | **Secondary** |
| **Results** | AUCg: Cortisol stress reactivity for each subject was measured by calculating area under curve with respect to ground (AUCG ) using the following formula AUCG = n−1 i= 1 m(i+1)+ mi× t i 2 where mi denotes cortisol level of sample i, n denotes the total number of samples, and ti denotes the time interval between samples i and i + 1 (ti will be specific to each participant).  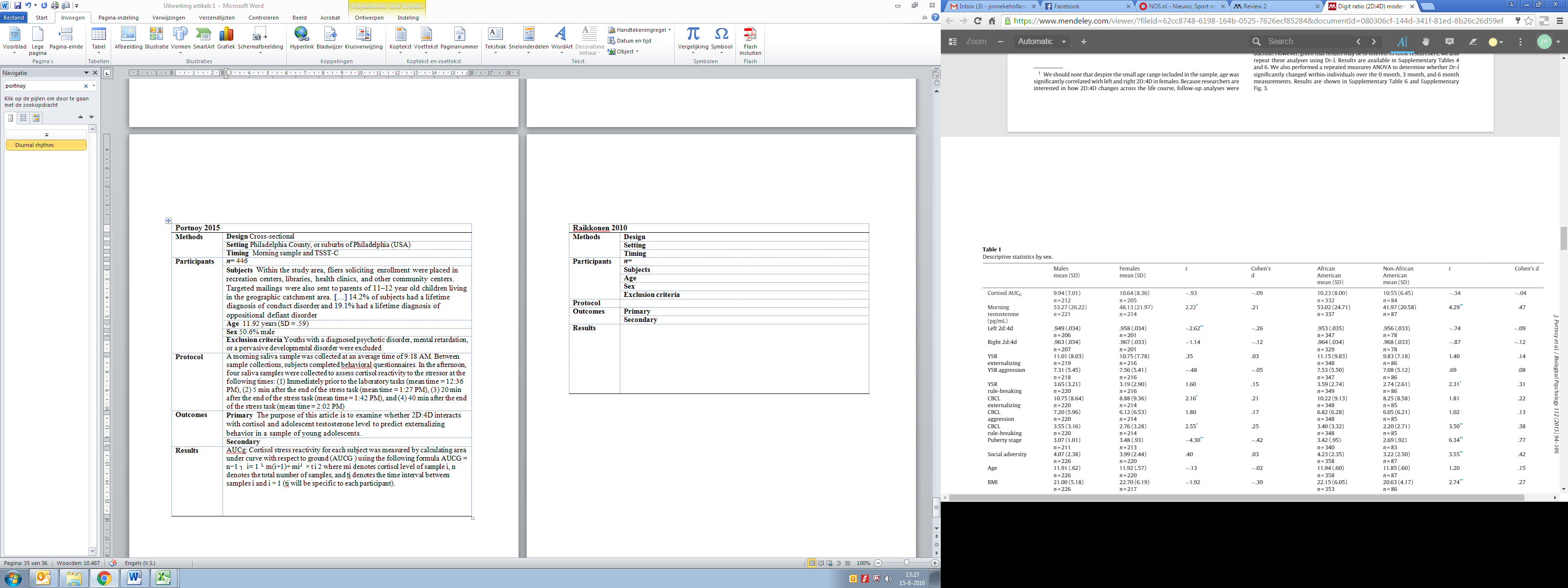 No difference in AUCg.  “In boys, left 2D:4D interacted with cortisol reactivity to predict self-reported externalizing behavior (B = 7.92, p < .05), aggression (B = 5.32, p < .05), and rule-breaking (B = 2.87, p < .05). In boys right 2D:4D also interacted with cortisol reactivity to predict self-reported externalizing behavior (B = 7.60, p < .05), aggression (B = 4.56, p < .05), and rule-breaking (B = 3.03, p < .05). We probed the interactions as shown in Figs. 1 and 2. For subjects with low 2D:4D (indicating higher prenatal testosterone), low cortisol reactivity was associated with higher levels of externalizing behavior. However, for subjects with high 2D:4D (indicating lower prenatal testosterone), there was no relationship between cortisol reactivity and externalizing behavior. Therefore, the expected relationship between low cortisol and increased levels of externalizing behavior was only present in subjects with low 2D:4D (see Table 3). In females, neither left nor right 2D:4D interacted with cortisol reactivity to predict aggression, rule-breaking, or externalizing (p < .05” |

### Pruessner 1997 [62]

| **Methods** | **Design** Cross-sectional |
| --- | --- |
|  | **Setting** Presumably children in the area around Trier |
|  | **Timing**  CAR |
| **Participants** | ***n=*** 42 children |
|  | **Subjects**  recruited through their parents by local newspapers, medication free. All subjects reported to be in good health (verified with questionnaire). |
|  | **Age** 11.16 years, SD 1.99 |
|  | **Sex** 21 girls, 21 boys |
|  | **Exclusion criteria** Not specified |
| **Protocol** | Subjects sampled saliva for cortisol assessment on three consecutive days. Sampling (in minutes after awakening): 0, 10, 20, 30  Ethical approval: not specified |
| **Outcomes** | **Primary** To report on the time course and intraindividual stability of the free cortisol response to awakening over days and weeks. |
|  | **Secondary** To document the influence of gender, age, smoking, and use of estrogen-containing medication on this endocrine response |
| **Results** | “There was a tendency towards larger increased in girls compared to boys.”  “In study 1, early morning free cortisol levels could only be sampled up to 30 minutes after awakening. Thus, there are only marginal differences in the cortisol levels between boys and girls.”  (Also adults taken into account): “While women tended to show a further increase (or a delayed decrease), cortisol concentrations clearly decreased in men after the first half hour resulting in smaller AUCS compared to women” AND “In Study 1, where saliva sampling ended after 30 minutes, a tendency for further increases in women was observed.”  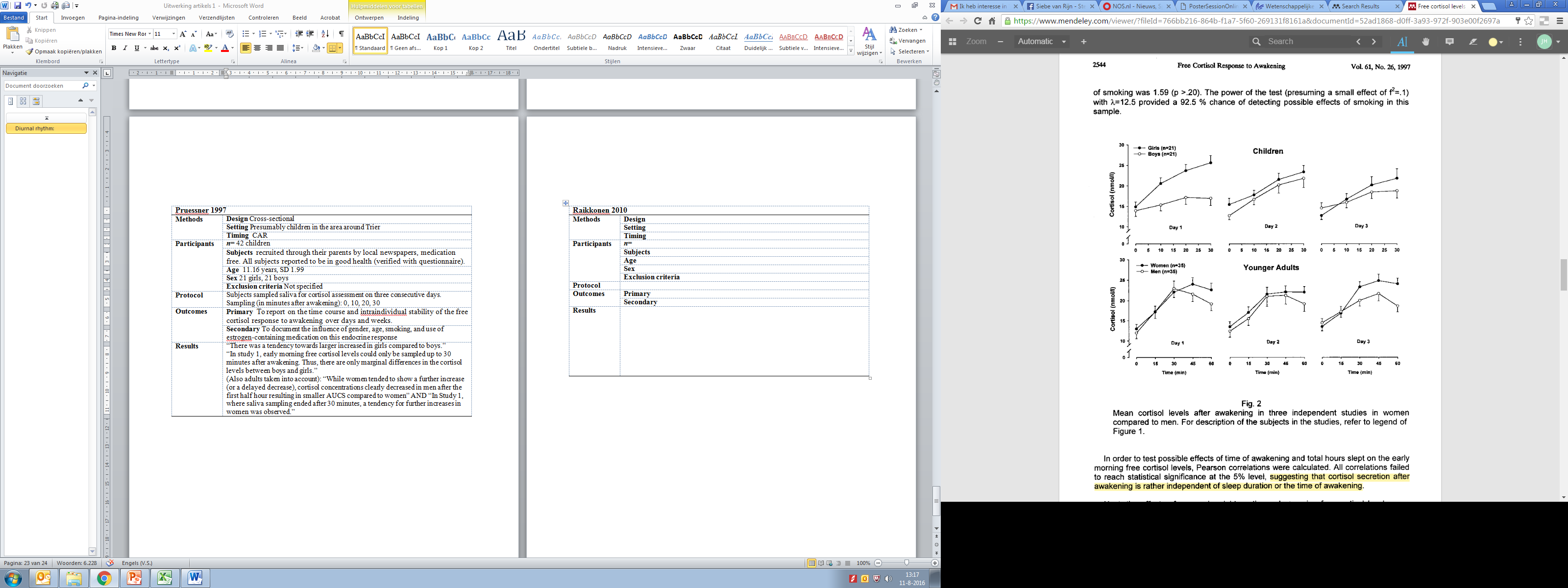 |

### Raikkonen 2010 [63]

| **Methods** | **Design** A random, population-based urban cohort (initially used to investigate the effects of maternal licorice consumption) of initially 1049 infants born between March 1 and November 30 1998 |
| --- | --- |
|  | **Setting** Helsinki, Finland |
|  | **Timing**  Salivary samples were obtained during a 1-d peiod, at awakening (mean – 0753 h; SD = 50 min), 15 and 30 min thereafter, and at 1030 h, 1200 h, 1730 h and at bedtime (mean = 2115 h; SD = 75 min). + TSST-C |
| **Participants** | ***n=*** 286 for diurnal salivary cortisol, 292 for salivary cortisol during stress |
|  | **Subjects**  Random, population-based sample |
|  | **Age** 8.1 years, SD=0.3 years |
|  | **Sex** No numbers mentioned, but 47.4-48.2% male depending on association studied |
|  | **Exclusion criteria** Due to financial constraints: only those families still living close to or in the greater Helsinki area were invited |
| **Protocol** | Salivary samples were obtained during a 1-d period, at awakening (mean – 0753 h; SD = 50 min), 15 and 30 min thereafter, and at 1030 h, 1200 h, 1730 h and at bedtime (mean = 2115 h; SD = 75 min).  TSST: arrival 1200h or 1400h, salivary samples were obtained at arrival and at baseline, an immediately and 10, 20, 30, and 45 min after rewarding the child with the favorite toy, which indexed completion of the TSST-C stress protocol  Ethical approval: obtained |
| **Outcomes** | **Primary** The associations between actigraphy-based sleep pattern with diurnal salivary cortisol pattern and with salivary cortisol responses to the TSST-C. |
|  | **Secondary** The association between sleep pattern and salivary a-amylase responses to stress |
| **Results** | " Finally, because associations with cortisol (22) and a -amylase (28) may vary according a-amylase to sex, we tested whether sex moderated any of the associations. In no instance was there a significant sex-interaction term (P> 0.07) (data not shown). For this reason, we report the results in both sexes combined."  “Boys (relative to girls) and older (relative to younger) children displayed lower cortisol levels after the TSST-C (P<0.05). There were no other significant associations between sex, age, BMI, or mother’s occupational status and hormonal parameters.” |

### Rosmalen 2005 [64]

| **Methods** | **Design** Longitudinal |
| --- | --- |
|  | **Setting** “five municipalities in the North of the Netherlands, including both urban and rural areas.” |
|  | **Timing**  Diurnal rhythm |
| **Participants** | ***n=*** 1768 |
|  | **Subjects** “the municipalities selected were requested to give names and addresses of all inhabitants born between 10-01-1989 and 09-30-1990 (first two municipalities) or 10-01-1990 and 09-30-1991 (last three municipalities), yielding 3483 names. Simultaneously, primary schools (including schools for special education) within these municipalities were approached with the request to participate in TRAILS; i.e. pass on students’ lists, provide information about the children’s behavior and performance at school, and allow class administration of questionnaires and individual testing (neurocognitive, intelligence, and physical) at school. School participation was a prerequisite for eligible children and their parents to be approached by the TRAILS staff, with the exception of children already attending secondary schools (1%)” |
|  | **Age** 11.08±0.55 years |
|  | **Sex** 894 girls, 874 boys |
|  | **Exclusion criteria** “Children were excluded from the study if they were incapable to participate due to mental retardation or a serious physical illness or handicap; or if no Dutch-speaking parent or parent surrogate was available, and it was not feasible to administer part of the measurements in the parent’s language” |
| **Protocol** | “Children were instructed to collect three saliva samples: the first sample shortly after waking up (still lying in bed), the second sample 30 min later, and the third sample at 20:00 h. Both the sampling and the preceding day should be normal (school) days, without special events or stressful circumstances. Since the in TRAILS participating schools started at approximately the same time, the sampling-time variation of the morning samples among the children is limited and the estimated corresponding times are 0700 h for the first sample (Cort0700) and 0730 h for the second sample (Cort0730). Children were instructed not to collect saliva when they were ill, had a cold, had a headache, or were menstruating. Furthermore, they were requested not to take any medication, if possible.”  Ethical approval: obtained |
| **Outcomes** | **Primary** “to gain insight into individual differences in HPA-axis physiology in the morning and evening phases of the diurnal cycle, and to study their relationship  with potential confounders in a large community cohort of 10–12 year-old boys and girls.” |
|  | **Secondary** |
| **Results** | Girls and boys differ significantly with regard to both morning cortisol levels (Cort0700 and Cort0730) but not with respect to their evening cortisol level (Cort2000).  Sex-differences were already present in prepubertal children: significant differences between boys and girls in tanner stage 1 are found regarding Cort0700 (11.06 vs. 11.99, t=2.330; p=0.020), Cort0730 (14.75 vs. 15.88, t=2.049; p= 0.041) and AUCG (6.43 vs. 6.93, t=2.717; p= 0.007), but not in Cort2000 (1.84 vs. 2.05, t=1.813; p=0.070) or AUCI (0.87 vs. 0.91, t=0.260; p= 0.795).  Next, we examined the effect of age and puberty. Despite its small range, age is significantly related to Cort2000 in the total group (r=0.098; p< 0.001) and in boys (r=0.099; p=0.004) and girls (r=0.101; p=0.003) separately. There were no significant correlations between age and the other cortisol variables although Cort0700 in the total group (r=0.046, p=0.061) and AUCG in the total group (r=0.043; p=0.081) and in girls separately (r=0.068; p=0.052) approached significance (all other variables \|r\|<0.057, pO0.10)). However, since age is related to season of sampling (F(11,1697)=24.295; p<0.001), these results could be due to confounding by seasonal influences. We performed linear regression analyses for each cortisol variable, with age and the quadratic effect of month as independent variables. After correction for seasonal effects, age was not significantly related to Cort2000 in the total group (b=0.043; p=0.101) or in girls (b=0.061; p=0.092) and boys (b=0.026; p=0.504) separately.  To study the influence of pubertal development, in accordance with earlier studies we categorized the children in pre-early puberty (Tanner stages 1 and 2) and mid-post puberty (Tanner stages 3, 4 and 5). There were no significant differences between these groups in any of the cortisol variables in the total group (in all cases \|t\|<1.64\|, p>0.10) or for boys and girls separately (boys: in all cases \|t\|<1.57, p>0.11; girls: in all cases \|t\|<0.84, p>0.40). There were also no significant correlations between Tanner stage and any of the cortisol variables in the total group (in all cases \|r\|<0.05, p>0.10) or for boys and girls separately (boys: in all cases \|r\|<0.020, p>0.59; girls: in all cases \|r\|<0.048, p>0.18). Since Tanner stage was significantly related to sampling month (Х^2^ (df=11)=20.58, p=0.038) and to age (r=0.237; p<0.001), we again performed linear regression analyses for each cortisol variable, with Tanner stage, age and the quadratic effect of month as independent variables, for the total group and for boys and girls separately. This correction for age and seasonal influences did not change the results in the total group (in all cases \|b\|<0.041, p>0.12) or for boys and girls separately (boys: in all cases \|b\|<0.017, p>0.65; girls: in all cases \|b\|<0.070, p>0.07).  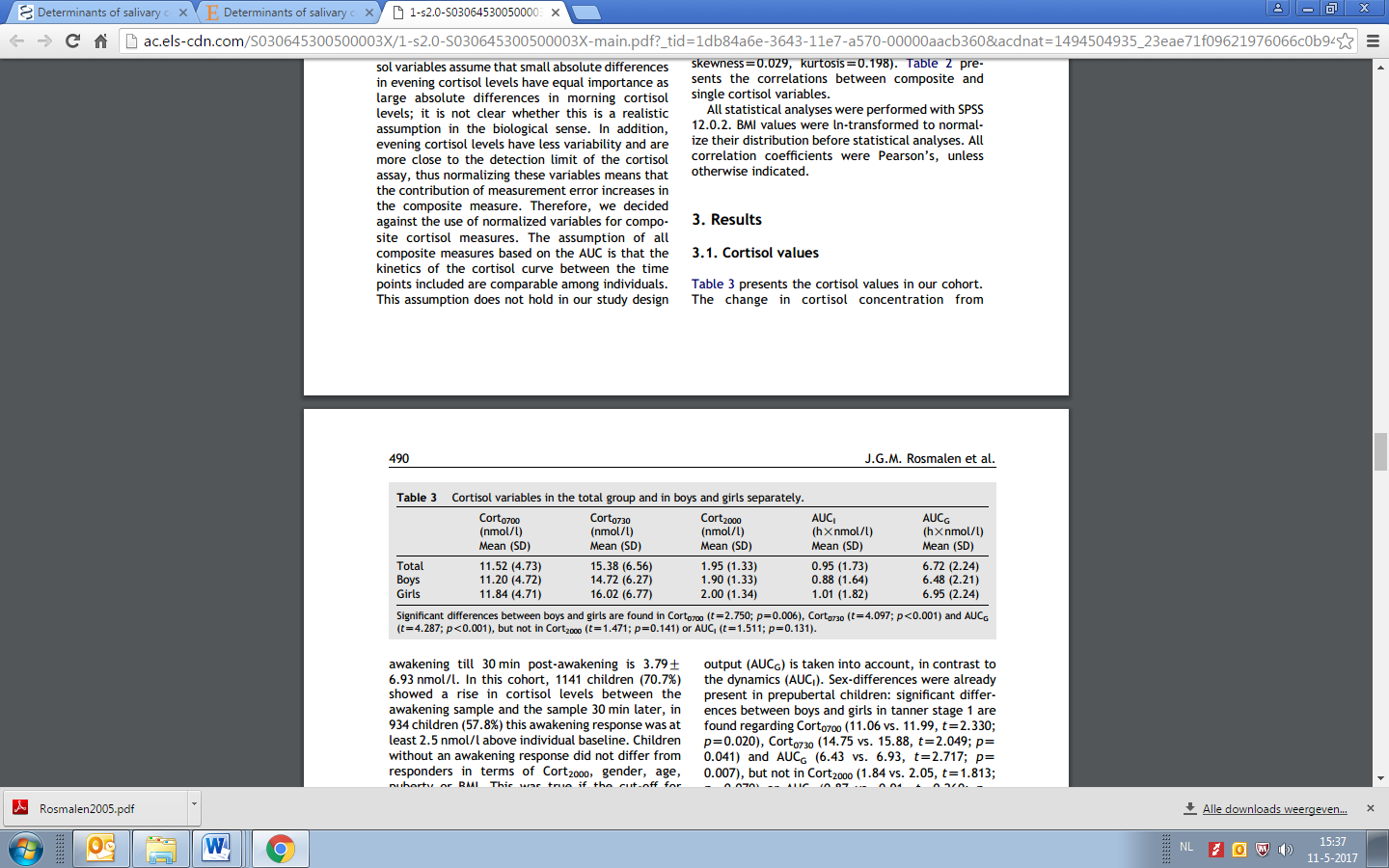 |

### Ross 1986 [65]

| **Methods** | **Design** Cross-sectional |
| --- | --- |
|  | **Setting** “All studies were performed at the National Institutes of Health Clinical Center |
|  | **Timing** CRH simulation test |
| **Participants** | ***n=*** 21 |
|  | **Subjects**  “normal children” |
|  | **Age** 6 to 15 years |
|  | **Sex** Not specified |
|  | **Exclusion criteria** Not specified |
| **Protocol** | One hour before testing, an iv catheter was inserted in an antecubital vein. All subjects remained supine throughout the test. An iv bolus dose of CRH (1ug/kg) was given between 1900 and 2000 h. (…) Blood was drawn at -15, 0, 30, 60, 90, 120 and 180 min after CRH.  Ethical approval: obtained |
| **Outcomes** | **Primary** To assess whether there are age-related changes in the ACTH and cortisol responses to CRH during childhood. |
|  | **Secondary** We also evaluated the relationship between cortisol-binding globulin (CBG) and chronological age in normal girls and boys, since developmental changes in CBG levels might influence the cortisol responses to CRH. |
| **Results** | “The plasma ACTH and cortisol levels did not differ significantly between the girls and the boys.”  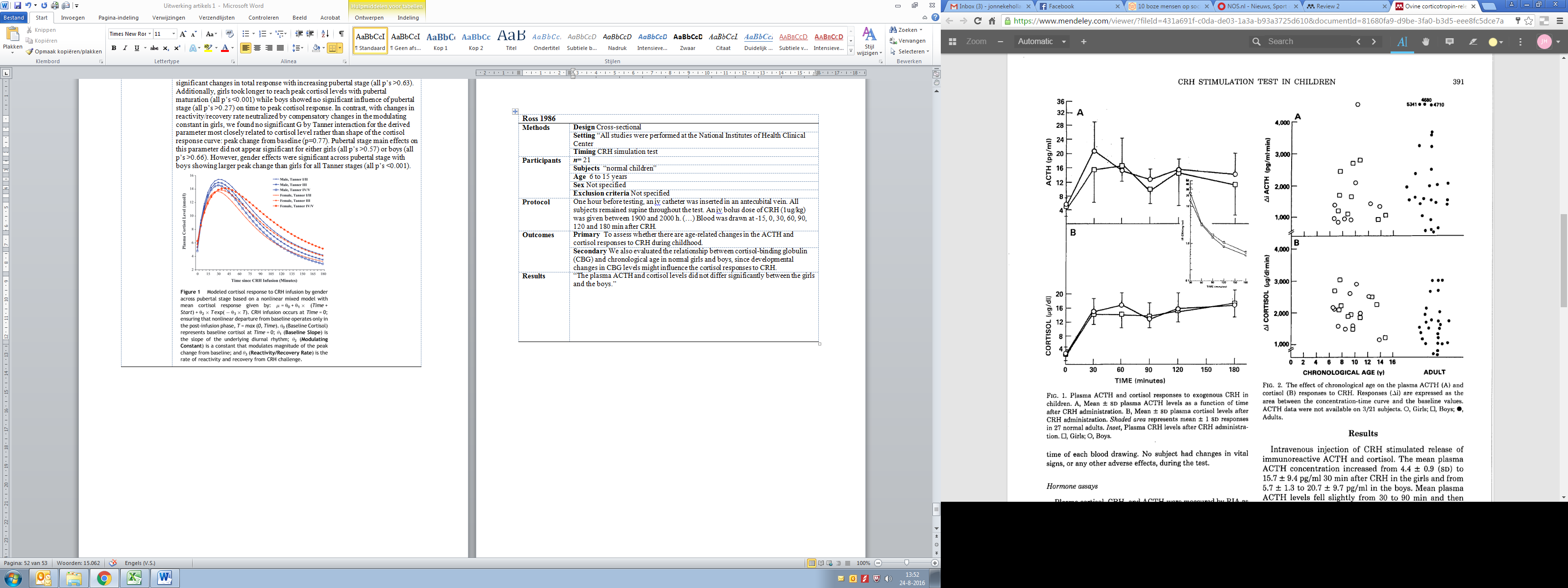 |

| **Methods** | **Design** Cohort study |
| --- | --- |
|  | **Setting** Milwaukee and Madison, Wisconsin |
|  | **Timing**  Diurnal rhythm (no CAR) |
| **Participants** | ***n=*** 346 |
|  | **Subjects**  Pregnant women and their partners/husbands were recruited at health care clinics in Milwaukee (80%) and Madison (20%), Wisconsin. Because of the original focus of the project, female participants were included in the study based on the following criteria: 1) older than age 18; 2) living with the biological father, 3) at least one member of the couple working for pay; 4) not a student, and 5) not unemployed. […] Families were followed repeatedly over time and, of the initial eligible families, 346 adolescent participants (62%) provided saliva samples and measurements of height and weight for at least one assessment between early and mid-adolescence, which made them eligible for the present analyses. |
|  | **Age** Follow-up at ages 11, 13, and 15 |
|  | **Sex** Not specified (sex is “averaged” between 0 and 1, and mean is 0.51 = 51% boys?) |
|  | **Exclusion criteria** Not specified, but it appears that not having saliva samples taken at at least one of the ages meant exclusion from this study |
| **Protocol** | When participants were 11,13, and 15 years old, they collected saliva samples at home for 3 days across three specific target collection times set by participants with research staff to match study needs and participants’ individual schedules: (1) shortly after waking; (2) a time between 3:00P.M . and 7:00P.M .; and (3) a time just before bed. Average times of actual sample collection were 8:53A.M. (SD-82 minutes), 4:50P.M . (SD-75 minutes), and 9:54P.M. (SD- 63 minutes). At each of these three assessment periods, adolescents were instructed to record pertinent time variables, collect samples prior to eating, and freeze samples immediately after collection.  Ethical approval: obtained |
| **Outcomes** | **Primary** The current study examines concurrent (i.e., measured at the same point in time) and longitudinal (i.e., using earlier cortisol measures to predict later body mass index [BMI]) associations between diurnal cortisol and BMI across adolescence. |
|  | **Secondary** The present study also considers a number of other pertinent variables that may help to explain previous null associations in the adolescent obesity literature |
| **Results** | “There were sex difference in level of cortisol, although associations changed over time”  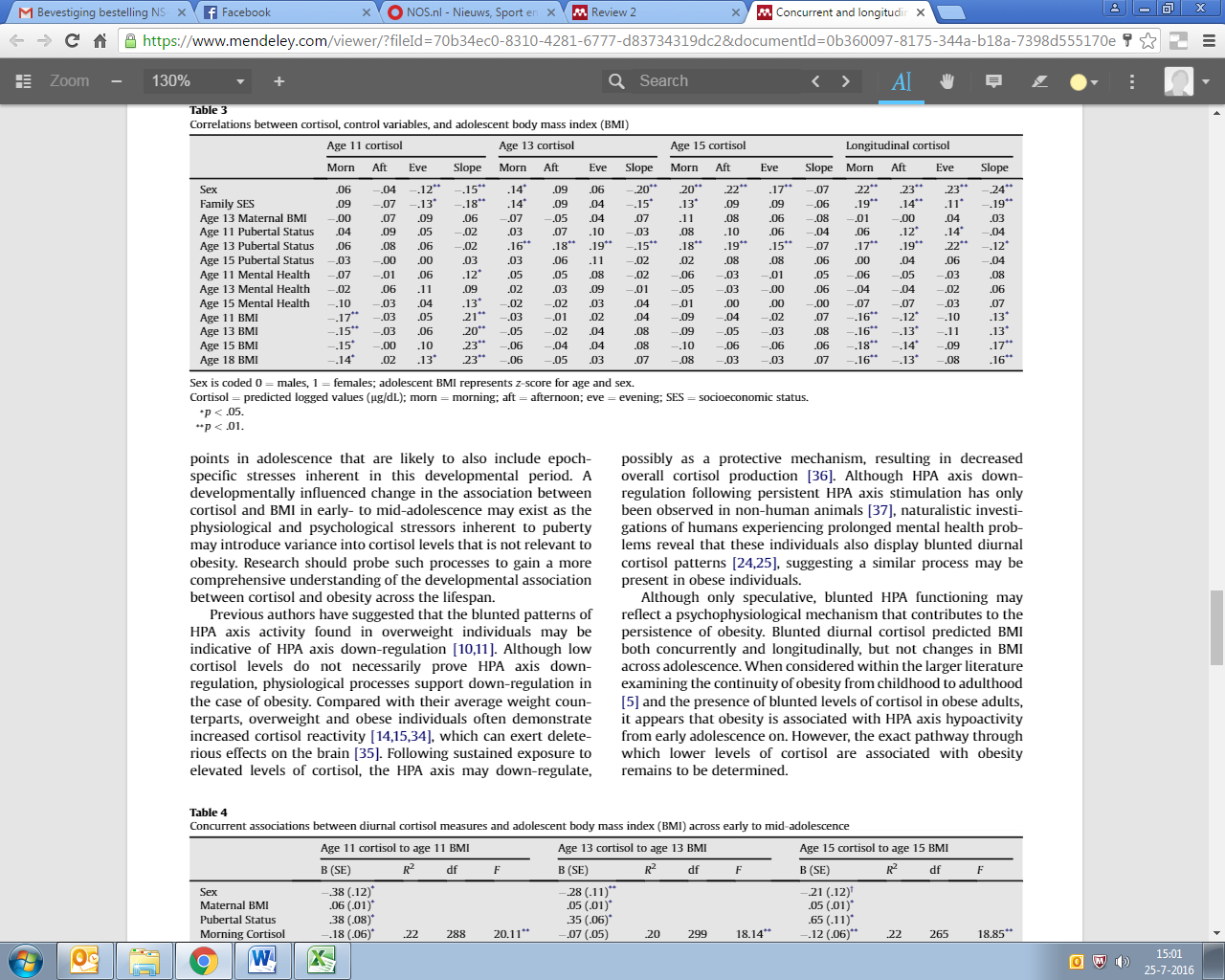  Not quite sure how to interpret these “correlations” |

### Ruttle 2013 [66]

| **Methods** | **Design** Cohort study |
| --- | --- |
|  | **Setting** Madison and Milwaukee, Wisconsin |
|  | **Timing**  Diurnal rhythm (no CAR) |
| **Participants** | ***n=*** 357 |
|  | **Subjects**  Participants in a longitudinal study, the Wisconsin Study of Families and Work (WSFW). Originally, 570 pregnant women and their partners were recruited from prenatal clinics for a study of maternity leave and health outcomes. To be eligible, female participants were required to be over the age of 18, in the second trimester of pregnancy, living with the baby’s biological father, and either employed or a full-time homemaker. |
|  | **Age** Follow-up at ages 11, 13, and 15 |
|  | **Sex** Not specified |
|  | **Exclusion criteria** Not specified |
| **Protocol** | Within each assessment, children were asked to collect saliva for three consecutive days (weekend and/or weekday) across three target collection times: (1) shortly after waking (before brushing teeth or eating breakfast); (2) between 3:00 PM and 7:00 PM (prior to dinner); and (3) just before going to bed. Target times were selected by the family prior to sample collection to accommodate their schedules and avoid their mealtimes; the same target time was selected across all 3 days.  Ethical approval: obtained |
| **Outcomes** | **Primary** to investigate the stability in cortisol and its circadian rhythm across ages 9 to 15 (grades 3 to 9), the years covering the transition to adolescence, thereby testing the hypothesis that there is trait-like stability across development |
|  | **Secondary** to investigate developmental influences by examining how HPA functioning changes within individuals across this time period, which spans on average from initiation through completion of pubertal maturation |
| **Results** | Gender impacts nearly every component of HPA-axis activity. Girls have higher cortisol, β=.037, t(355)=2.37, p<.018, steeper slopes, β=−.0025, t(355)=3.91, p <.0001, and more curvature to their rhythm than boys, β=.00011, t(9131)=2.99, p <.003. The decline in waking cortisol across development is less pronounced in girls than boys, β=−.0096, t (1038)=2.04, p<.04, and the quadratic age-effect shows greater curvature in girls than boys, β =.0008, t(1038)=2.43, p <.015 (see Figure 3). When puberty alone is examined, gender moderates the effect of puberty on the slope, β=.0002, t(1039)=1.95, p =.05. The circadian rhythm becomes flatter as children advance through puberty, especially girls. This persists after controlling for age, β =.001, t(1038)=2.1, p=.036  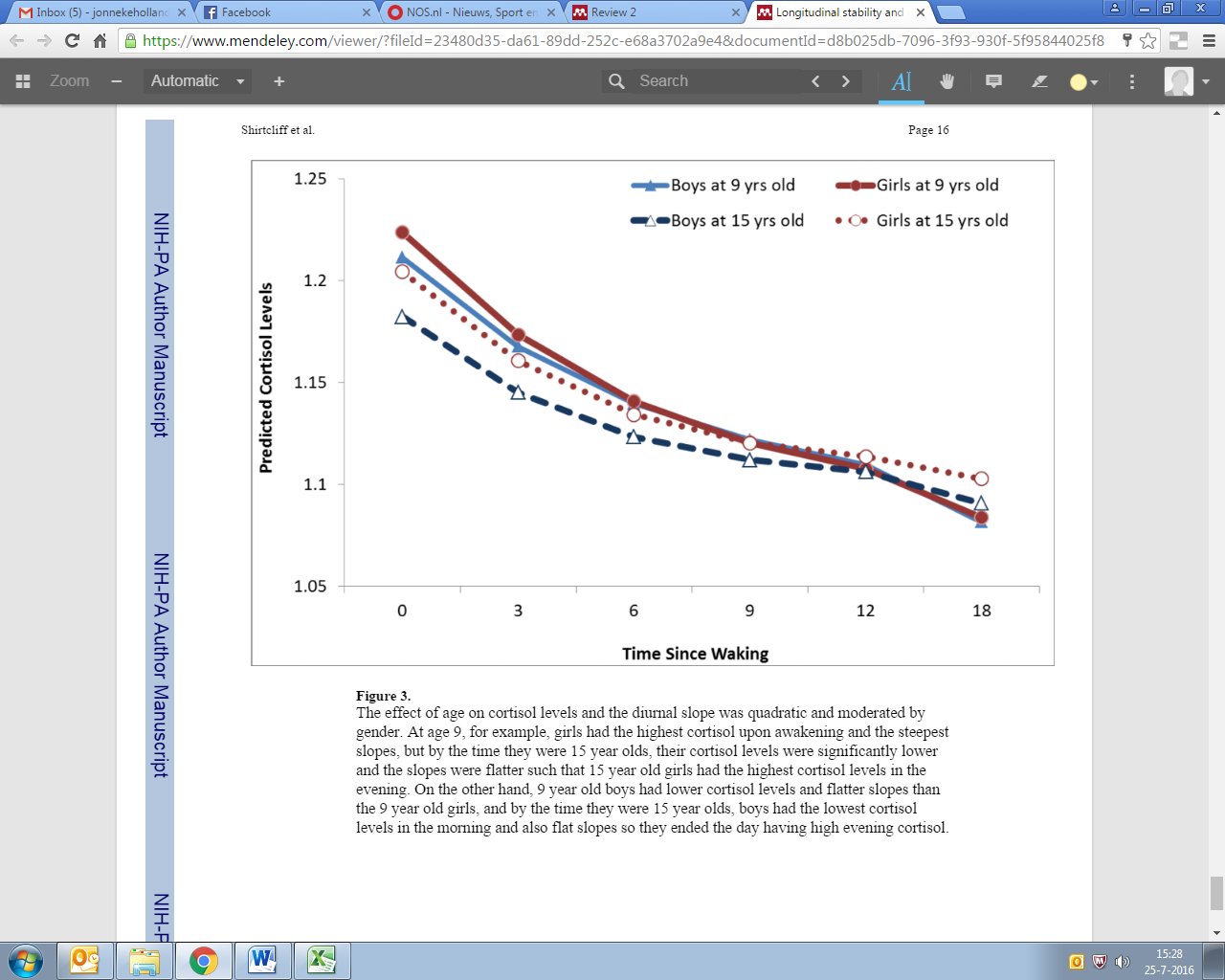 |

### Shirtcliff 2012 [67]

| **Methods** | **Design** Longitudinal |
| --- | --- |
|  | **Setting** “A large Southwestern city” |
|  | **Timing**  “Children participated in a series of tasks designed to assess children’s effortful control, social competence, and cognitive developmental level. |
| **Participants** | ***n=*** 84 |
|  | **Subjects** “Preschool-aged children residing in a large Southwestern city who were part of a longitudinal study of children’s social and emotional development.” |
|  | **Age** 54.07 months (SD=0.97 months) |
|  | **Sex** 43 boys, 41 girls |
|  | **Exclusion criteria** Not specified |
| **Protocol** | “immediately before the first saliva collection, children participated in a series of executive functioning tasks that required them to inhibit a predominant response behavior. Specifically, the children were trained to imitate the experimenter either knocking or tapping (flat hand) on the table. After the training, the rules to the game changed, so that the child was told to knock when the experimenter tapped and to tap when the experimenter knocked on the table. In the next executive functioning task, the child was instructed to say the word, “grass” when the experimenter held up a white card and to say , “snow” when the experimenter held up a green card. These tasks were presented as games and were not expected to elicit negative emotion.  After the executive functioning tasks, the experimenters then collected a saliva sample (see below). Next the children participated in an emotion-eliciting task from the Preschool Laboratory Assessment Battery and provided a second saliva sample approximately 10 minutes following the stressful task. A third saliva sample was collected at the end of the laboratory visit, approximately 40 minutes following the task.  Ethical approval: not specified (Smider, Child Dev, 2002: parental consent obtained) |
| **Outcomes** | **Primary** To investigate preschool children’s cortisol and sAA reactivity following a frustrating task and to examine the relations between cortisol and sAA reactivity to children’s observed emotions and maternal reports of dispositional emotionality, regulation, impulsivity, and maladjustment.” |
|  | **Secondary** “We were interested in analysing whether the relations between stress reactivity and children’s emotions and social functioning differed for boys and girls.”  “We testes whether preschool children’s cortisol moderated the relations between sAA and children’s emotions, regulation, and adjustment.” |
| **Results** | “We examined whether there were differences in the study variables as a function of children’s sex. There were no differences in either pre-test levels of cortisol and sAA or in cortisol or sAA reactivity” |

### Spinrad 2009 [68]

### Strahler 2010 [69]

| **Methods** | **Design** Cross-sectional |
| --- | --- |
|  | **Setting** Dresden, Germany, and surroundings |
|  | **Timing**  TSST-C |
| **Participants** | ***n=*** 62 children |
|  | **Subjects**  Children […] were recruited via a notice posted on campus of the Technische Universität Dresden, via an advertisement in a local newspaper and via personal contact. The group of children was recruited with the help of a local hospital, through which parents who delivered a baby there between 1998 and 2002 were contacted.  Participants were free of psychiatric and severe somatic diseases as evaluated by interview by one of the authors. |
|  | **Age** 6-10 years |
|  | **Sex** 32 boys and 30 girls |
|  | **Exclusion criteria** BMI>30, smokers, excessive alcohol consumption, under anti-hypertensive medication, asthma medication, anti-rheumatic medication, using psychotropic substances, sleeping pills or painkillers. |
| **Protocol** | Four saliva samples were collected immediately before, immediately after, 10 and 20 min after the stressor with the help of cotton swabs.  Ethical approval: obtained |
| **Outcomes** | **Primary** To investigate sAA responses to acute psychosocial stress in three relevant age groups representing relevant stages of human development. […] we aimed to test for associations of sAA with more established stress system markers, i.e., salivary cortisol. |
|  | **Secondary** To examine how subjective chronic stress levels influence sAA as ewll as HR and HRV stress responses. |
| **Results** | No sex effect, but a significant time x group x sex interaction was found. Univariate ANOVA of delta scores revealed a significant main effect of group. However, post hoc tests showed no difference between the groups, although older participants showed the highest mean stress response. While there was no main effect of sex, a significant group x sex interaction was found. As shown in figure 2B, acute cortisol stress responses increase with age in male participants, but remained constant through all age groups in female participants.  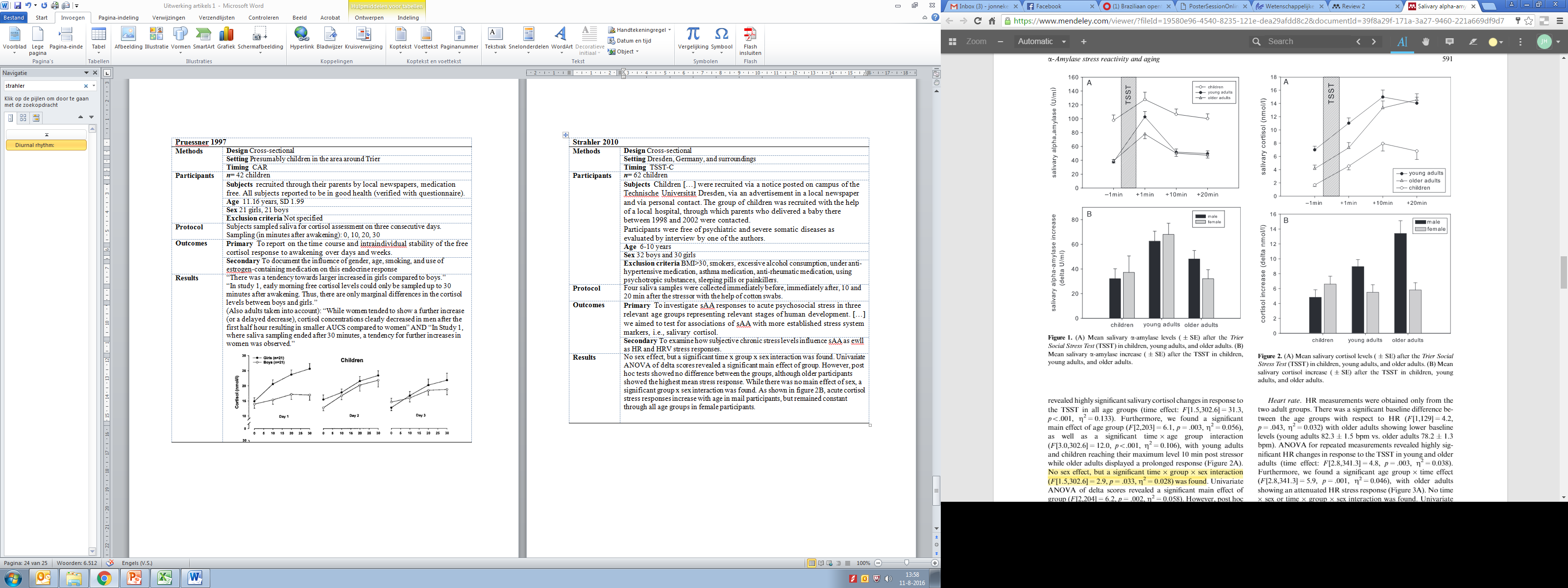  E-mail from the author:  “here comes the statistics for boys vs. girls only cortisol delta  (increase from baseline to the mean of +10min and +20min -N-logarithm):  t[58]=-1.391, p=0.170  Repeated measures ANOVA: F[1.41;81.48]=1.661, p=0.202  absolut values (boys/girls):  -1min: 1.72/1.63  +1min: 4.43/4.64  +10min: 6.98/8.96  +20min: 6.13/7.52  forgot the standard errors - as I'm too lazy, just copy paste from  SPSS output male  1: 1,72 /,18  2: 4,43 /,66  3: 6,98 /1,13  4: 6,13 /1,03  female  1: 1,63 /,19  2: 4,64 /,68  3: 8,96 /1,17  4: 7,52 /1,06   - Slight, non-significant higher response in girls |

### Stroud 2011 [70]

| **Methods** | Design Longitudinal |
| --- | --- |
|  | Setting Pittsburgh |
|  | Timing CRH challenge |
| **Participants** | *n=* 68 |
|  | Subjects Carefully screened controls recruited over three phases of a mutlti-project study of neurobehavioral characteristics of pediatric affective disolders who completed at least one baseline CRH challenge with available cortisol and Tanner stage data.  Participants were recruited through printed advertisements, health fairs, direct mailings, and personal contacts. The sample was primarily middle/upper class. All participants were physically healthy with no current or personal history of psychiatric disorder with low familial risk for depression. Medical history, physical examination, laboratory tests(…) were collected as part of the larger protocol. Personal history of psychiatric disorders was assessed using School Age Schedule for Schizophrenia and Affective Disorders, Epidemiologic version. (..) family history of psychopathology was assessed through interviews with first- and second-degree relatives. |
|  | Age Participants ages were 7.1-14.2 years old at baseline (m=10.5, SD 1.7), with age range 7.1 -16.5 (m:11.6, SD 1.9) including longitudinal data. |
|  | Sex 41% girls, 59% boys |
|  | Exclusion criteria Not further specified than mentioned under “subjects” |
| **Protocol** | The CRH infusion protocol began at 4:00 pm (phases 2 and 3) or 5.00 pm (phase 1) on the second laboratory day. These time were chosen as points when the HPA axis is believed to be relatively quiescent. The CRH challenge protocol included 30-40 min pre-infusion baseline, followed by 1ug/kg human CRH (hCRH) administered as an intravenous infusion over two minutes, then 90-180 min of recovery. Nine to ten plasma cortisol samples were collected over the course of the CRH challenge protocol. For phase one of the study, basal samples were collected at -30, -15 and 0 min, with 0 as the time of CRH infusion. After hCRH infusion, samples were obtained at 15, 30, 60, 90, 120, 150, and 180 min. Time points for phases two and three were -40, -20 and 0 min for the basal sampling, and 5, 10, 15, 20, 60, 90 min after hCRH infusion.  Ethical approval: obtained |
| **Outcomes** | Primary “We investigated sex differences in HPA response to corticotropin releasing hormone (CRH) challenge over puberty in a carefully screened normative sample. |
|  | Secondary |
| **Results** | “We found highly significant G by Tanner interactions for Baseline cortisol, modulating constant and reactivity/recovery rate (P’s<0.0001). Specifically, girls showed significant increases in baseline cortisol with pubertal maturation (p’s <0.02 for all three pairwise differences between pubertal stage groups), while boys showed highly significant declines of comparable magnitude (all p’s <0.01); when subject-level rather than session-level random effects were included, the magnitude of the changes was attenuated for boys, but not for girls. Girls also showed very highly significant decreases in the Modulating constant with increasing pubertal stage (all p’s <0.001), while no significant differences emerged for boys p’s >0.29). Finally, very highly significant decreases in reactivity / Recovery rates were observed in girls with increasing pubertal stage (all p’s <0.0001), while boys showed little change in reactivity/recovery rates over puberty (all p’s >0.027).  Significant G by Tanner interactions in reactivity/recovery rates led to significant G by Tanner interactions for two of the derived parameters more closely related to the shape rather than the overall levels of the cortisol response curve: AUC (p<0.05) and time to peak (p<0.0001). Specifically, girls showed significant increases in total cortisol response to CRH challenge with pubertal maturation (p’s <0.01 for all three pairwise differences in AUC between pubertal groups), while boys showed no significant changes in total response with increasing pubertal stage (all p’s >0.63). Additionally, girls took longer to reach peak cortisol levels with pubertal maturation (all p’s <0.001) while boys showed no significant influence of pubertal stage (all p’s >0.27) on time to peak cortisol response. In contrast, with changes in reactivity/recovery rate neutralized by compensatory changes in the modulating constant in girls, we found no significant G by Tanner interaction for the derived parameter most closely related to cortisol level rather than shape of the cortisol response curve: peak change from baseline (p=0.77). Pubertal stage main effects on this parameter did not appear significant for either girls (all p’s >0.57) or boys (all p’s >0.66). However, gender effects were significant across pubertal stage with boys showing larger peak change than girls for all Tanner stages (all p’s <0.001).  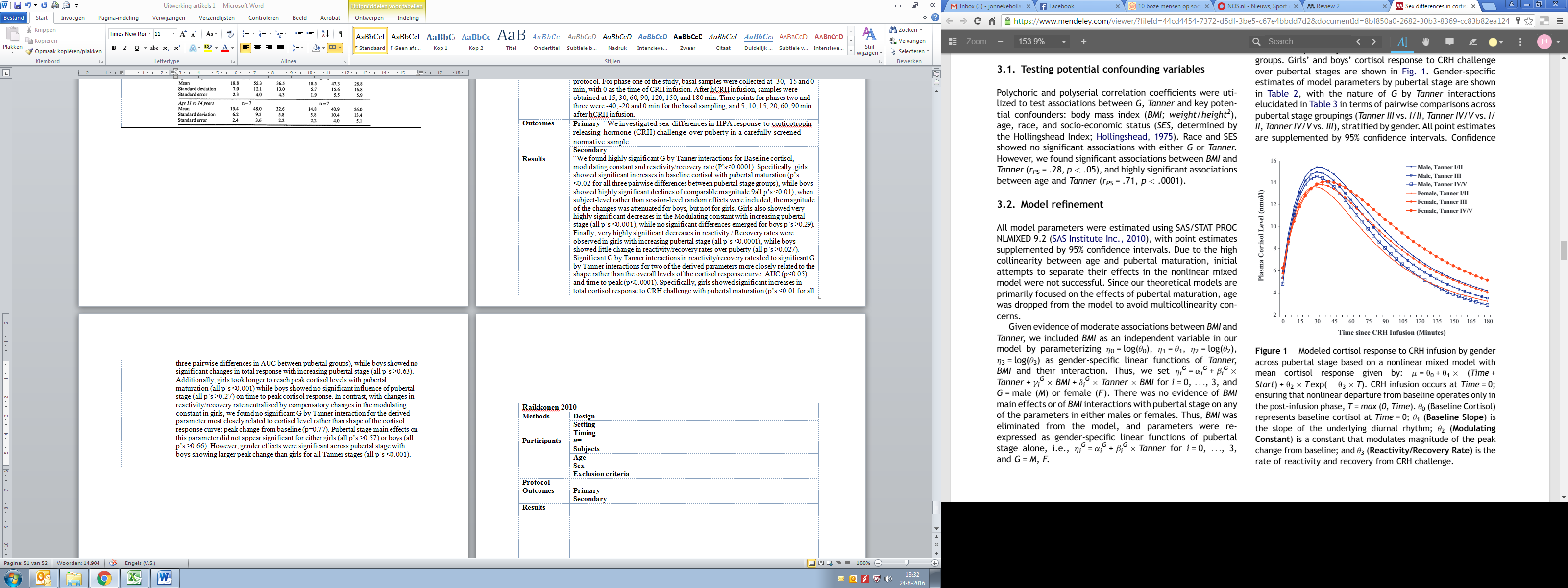 |

| **Methods** | **Design** Cross-sectional |
| --- | --- |
|  | **Setting** Not specified, presumably somewhere in Poland |
|  | **Timing**  Before and after exercise |
| **Participants** | ***n=*** 29 |
|  | **Subjects** Junior rowers (between 2,5 and 3 years of experience) |
|  | **Age** Men: 17.3 +/- 0.8 years, women: 16.4 +/- 0.6 years |
|  | **Sex** 14 men, 15 women |
|  | **Exclusion criteria** Not specified |
| **Protocol** | “Graded exercise, performed on a rowing ergometer (Concept II), consisting of three periods (5 min each) of exercise separated by 5-min intermissions. Consecutive periods were of increasing intensity, 50%, 70% and 85% of individual mean power output recorded previously in a laboratory exercise simulating a 2-km run.”  Cortisol: “Blood was withdrawn from an antecubital vein just before and 2 min after the end of the exercise.”  Ethical approval: obtained |
| **Outcomes** | **Primary** “A possibility exists that not only the postexercise cortisol concentration would depend on the exercise intensity and duration, but that the exercise performance might be expected to depend on the pre-exercise blood concentration of that hormone and that supposition prompted us to undertake this study” |
|  | **Secondary** |
| **Results** | Table 3: no significant correlation in junior male rowers pre- and post-cortisol, but there was a significant correlation in junior female rowers.  From table 2: approximately same pre-exercise levels, but males decrease on average, while females show an increase in cortisol levels. |

### Stupnicki 1995 [71]

| **Methods** | **Design** Longitudinal |
| --- | --- |
|  | **Setting** Cincinnati / Pennsylvania? |
|  | **Timing**  Diurnal rhythm + CAR |
| **Participants** | ***n=*** 111 |
|  | **Subjects** Participants in a longitudinal study of puberty and behaviour. Eligibility criteria were as follows: boys ages 9, 11, or 13 years; girls ages 8, 10, or 12 years; not on medications that would interfere with hormone levels (e.g., oral steroids; and free from chronic health problems (e.g., diabetes, cancer) or serious mental health problems that would interfere with completing the questionnaires. Children on psychotropic medications were included. |
|  | **Age** boys: 9, 11, or 13 years; girls 8, 10, or 12 years. |
|  | **Sex** Boys 56, girls 55 |
|  | **Exclusion criteria** Not further specified than previously mentioned |
| **Protocol** | Three samples of saliva were collected at 20-min intervals upon awakening and prior to breakfast, teeth brushing, or eating: Sample 1 was obtained immediately on wakening, Sample 2 at 20 min postwake time, and Sample 3 at 40 min postwake time. Additional samples were collected at noon prior to the midday meal, at 4:00 p.m., and at bedtime. Participants were instructed to rinse their mouths with water before passively drooling into a 5-mL tube and to collect saliva to the 4-mL mark on the tube within 5 min and then put the tube in the refrigerator  Ethical approval: obtained |
| **Outcomes** | **Primary** To examine the relationship between morningness/eveningness (M/E) and morning-to-afternoon cortisol ratio, pubertal timing, and antisocial behavior |
|  | **Secondary** |
| **Results** | “The mean and standard deviations appear in Table 2 for M/E, am to pm cortisol ratio, CBCL syndromes (attention problems, rule-breaking behavior, and aggressive behavior), DISC-IV (parent) ODD and CD symptoms and relational aggression for boys and girls. There were no sex differences in the means for any of the variables.”  “The am to pm cortisol ratio was negatively related to attention problems for boys but not for girls.” AND “a small change from am to pm cortisol characterized only boys with attention behavior problems” |

### Susman 2007 [72]

### Trickett 2014 [73]

| **Methods** | **Design** data from the initial assessment of a longitudinal study |
| --- | --- |
|  | **Setting** “A large wester U.S. city” |
|  | **Timing**  TSST-C |
| **Participants** | ***n=*** 303 maltreated children, 151 in the comparison group |
|  | **Subjects** “The participants who comprised the maltreatment group (N = 303) were recruited from active cases in a county Department of Children and Family Services (DCFS) of a large western U.S. city. The inclusion criteria were (1) a new substantiated referral to DCFS in the preceding month for any type of maltreatment (i.e., neglect, physical abuse, sexual abuse, emotional maltreatment); (2) child age of 9–12 years; (3) child identified as Latino, African American or Caucasian (non-Latino); (4) child residing in 1 of 10 urban zip codes within the county at the time of referral to DCFS.”  “The comparison group (N = 151) was recruited via mail using names from school lists of children aged 9–12 years residing in the same 10 zip codes as the maltreatment group. These children were screened for DCFS referrals for abuse or neglect.” |
|  | **Age** maltreated: M = 10.84 years, SD = 1.16); comparison: M = 11.11 years, SD = 1.15 |
|  | **Sex** 50% male maltreated, 60% male comparison |
|  | **Exclusion criteria** “We also excluded 12 cases due to the use of steroidal medications that might influence cortisol levels.” |
| **Protocol** | TSST-C  Cortisol samples: “We collected six saliva samples, two before and four after the TSST-C.” “the first saliva sample occurred immediately after the informed consent procedure and 30 min before the stressor (i.e., the TSST-C). The second sample was collected 10 min before the stressor, immediately after a 5-min relaxation protocol involving listening to soft music while viewing a still slide of a beach scene. The TSST-C procedure lasted 14 min. The third sample was collected immediately after the stressor, and the fourth, fifth, and sixth samples were collected 10, 20, and 30 min after the end of the stressor, respectively.”  Ethical approval: obtained |
| **Outcomes** | **Primary** “to examine the association of recent maltreatment experiences with cortisol reactivity in young adolescents.” |
|  | **Secondary** “Our study also aimed to shed light on the association between child maltreatment and HPA axis functioning in male versus female youth” |
| **Results** | “The 6 (sampling time) X 2 (maltreatment status) X 2 (sex) repeated measures analysis, controlling for pubertal stage, time of day, and caregiver status, revealed a significant within sub-jects’ effect for sampling time, F(5, 426) =2.26, p = .048, a marginally significant between-group main effect for maltreatment group, F(1, 426) = 3.16, p = .076, and a main effect for sex, F(1, 426) = 14.36, p = .002. These main effects indicate that, on average, the release of cortisol is blunted in the mal-treated adolescents as compared with those in the comparison group and in girls compared to boys.”  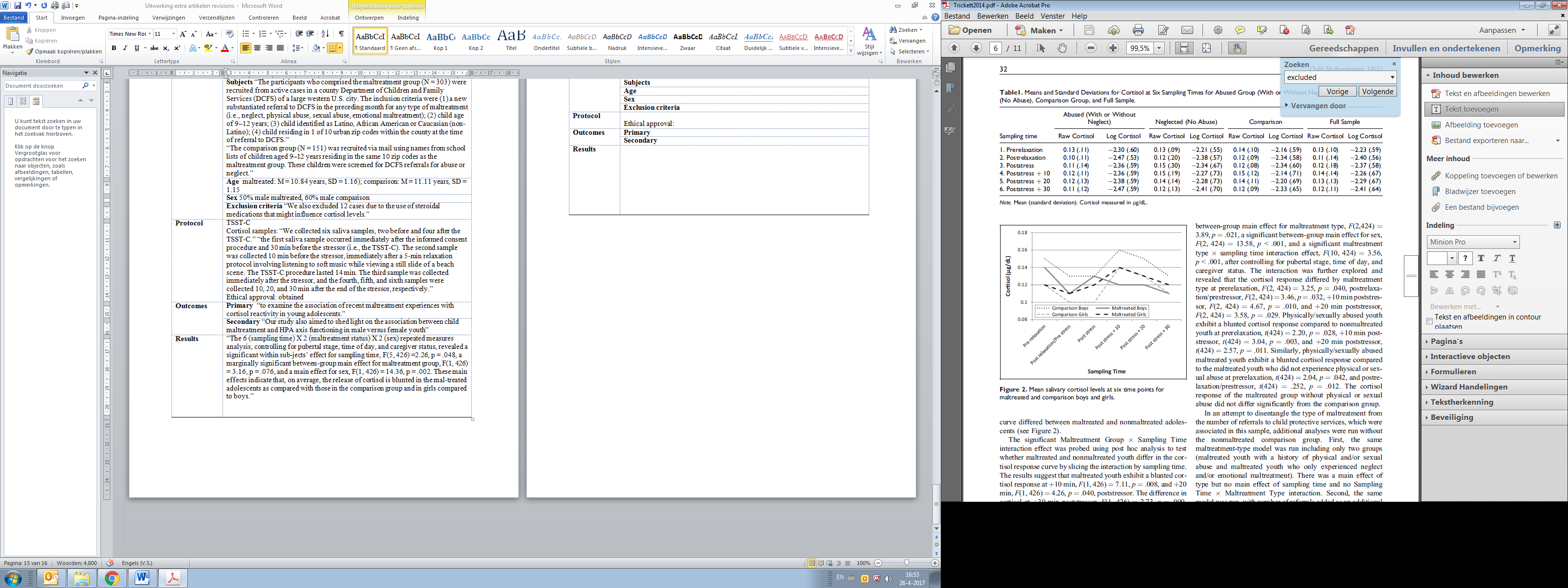 |

### Tsvetkova 1977 [74]

| **Methods** | **Design** Cross-sectional |
| --- | --- |
|  | **Setting** Not specified, presumably Bulgaria |
|  | **Timing**  Synthetic ACTH stimulation |
| **Participants** | ***n=*** 31 |
|  | **Subjects** Healthy children. “The tests were carried out as part of a larger trial using the same preparation, which has been the subject of previous reports.” |
|  | **Age** 4-14 years |
|  | **Sex** 16 boys, 15 girls |
|  | **Exclusion criteria** Not specified |
| **Protocol** | At 8 am, before breakfast, 5 ml of blood was withdrawn from the cubital vein. The children were then given 0.5mg tetracosactrin intramuscularly, and another 5 ml blood sample was taken 2 hours later. Plasma cortisol levels before and after drug administration were determined fluorometrically for all the children using the Ankov et al modification of the method of Silber et al.  Ethical approval: “Parental consent was obtained for this investigation, which involved only a minor stress on the children being tested” |
| **Outcomes** | **Primary** To test adrenocortical function in healthy children in various age and sex groups after administering synthetic ACTH. |
|  | **Secondary** |
| **Results** | “From the Tables it is clear that the cortisol excretion is independent of age or sex.” AND “although the cortisol level after stimulation with ACTH seems to be higher in boys than in girls, the difference is not statistically significant.”  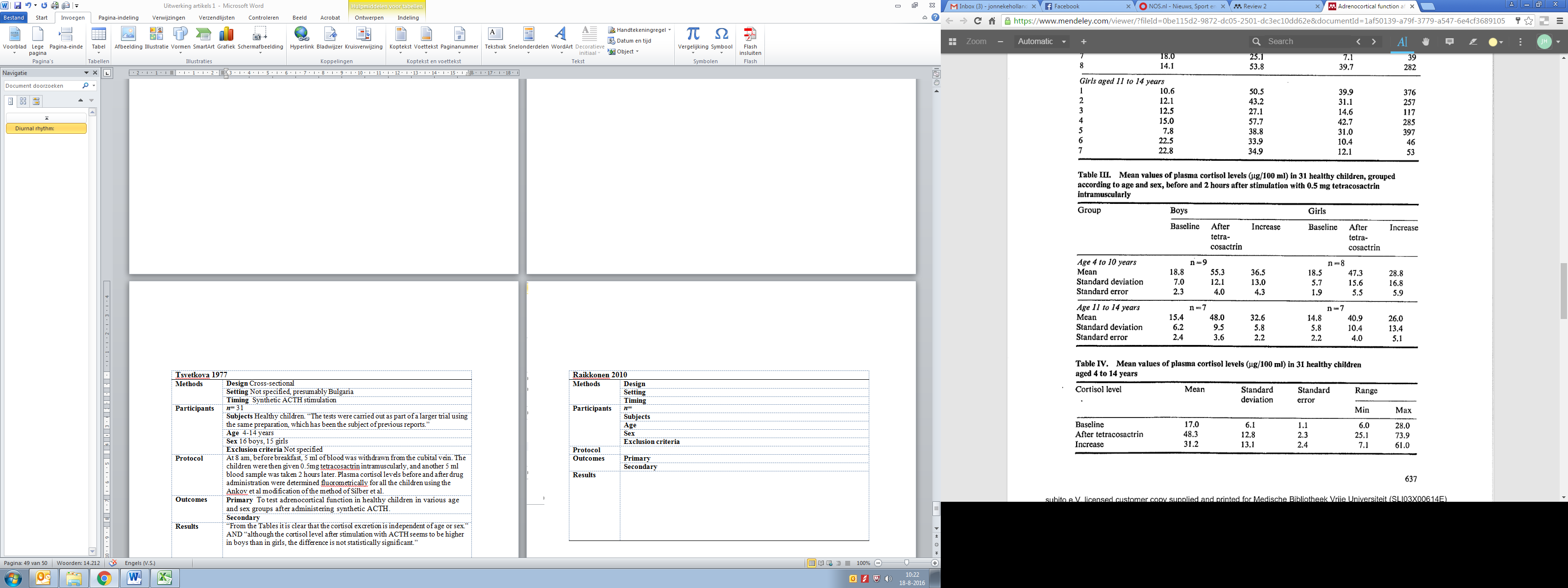 |

| **Methods** | **Design** Cross-sectional |
| --- | --- |
|  | **Setting** Regensburg, Germany |
|  | **Timing**  Diurnal rhythm + CAR |
| **Participants** | ***n=*** 21 |
|  | **Subjects**  School children of both genders between 10 and 14 years of age were recruited from local schools, direct communication with colleagues and their acquaintances, and a local dental office. |
|  | **Age** 10-14 years |
|  | **Sex** 11 males, 10 females |
|  | **Exclusion criteria** chronic diseases, medication intake, hormone therapy, allergies, or any invasive medical procedure |
| **Protocol** | Each participant was given 22 saliva collecting sets consisting of a SaliCap® collection tube (IBL Immuno Biological Laboratories, Hamburg, Germany) and one short plastic straw. They were instructed to start sam -pling immediately after waking up: during the first three hours, one sample each 20 minutes, then one sample each hour until bedtime. The children used the plastic straw provided to expel the saliva into the collection tubes. Altogether, 20 samples were collected over a period of one day. In accordance with Gröschl et al.13 we tried to exclude any additional stressful event that might affect cortisol levels. Since this tight sampling schedule interfered with school routine, sampling took place on weekends, holidays or during vacations.  Ethical approval: obtained |
| **Outcomes** | **Primary** to establish detailed levels of daily salivary cortisol secretion in school children in order to determine the precise time range of its lowest concentration and individual variability |
|  | **Secondary** |
| **Results** | We tested for gender differences in waking times and cortisol concentration using Mann-Whitney’s U-test and found no statistical significant differences between gender for both measures.  Within 20min after waking up the cortisol concentration given as mean (± S.D.) reached its maximum of 9.69 (±3.89) nmol/L, and decreased by 50 % after an additional 60 minutes. During the remaining part of the day, saliva cortisol levels dropped to 1/20th of the initial concentration (Figure 3 ). Between 3 and 6 hours after waking up the saliva cortisol stagnated at 4.03 (±2.44) nmol/L (Figure 3 ). The potential interaction between gender and time was tested using SPSS’s GLM procedure for repeated measures. No gender specificity was found (p = 0.550)  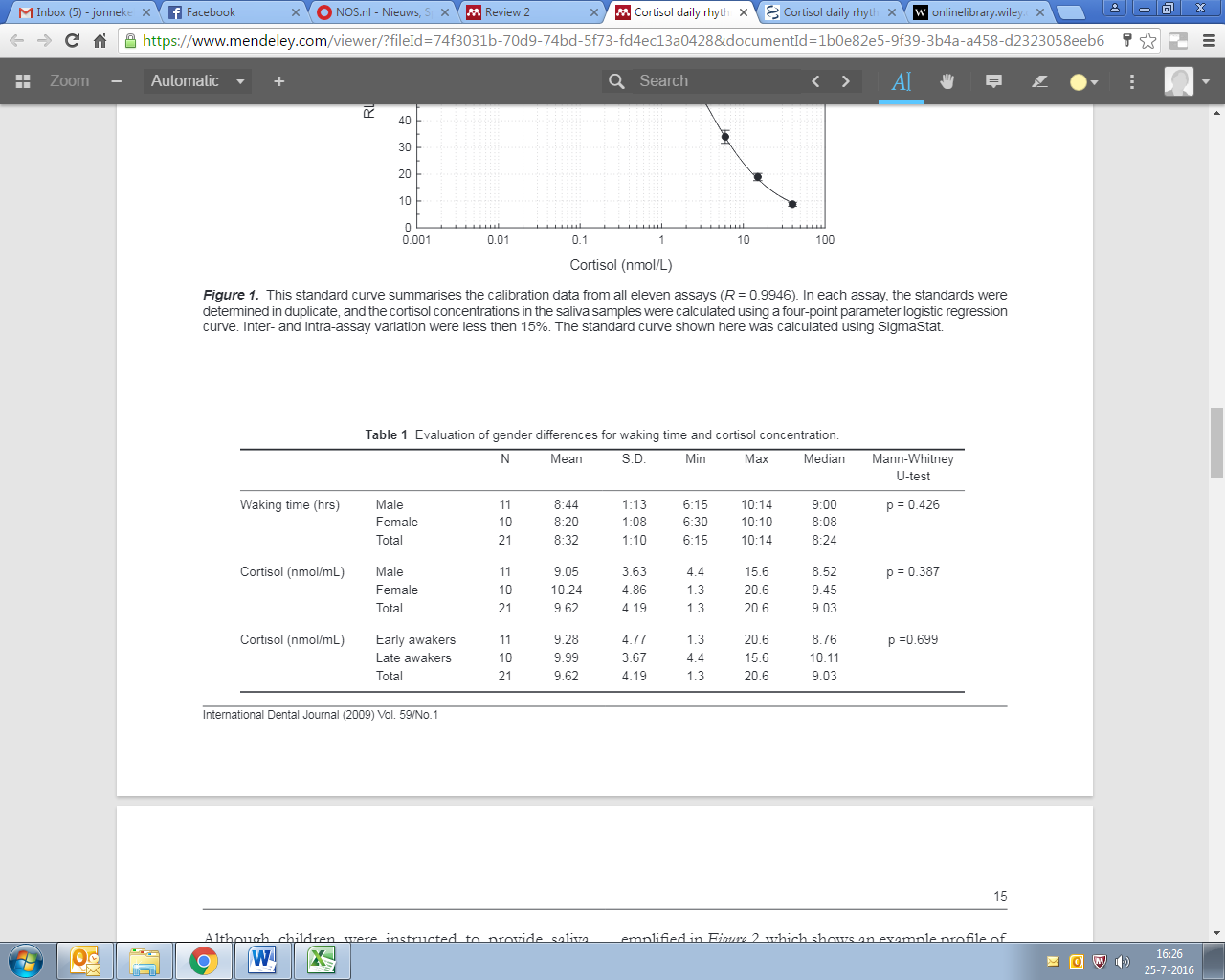 |

### Tzortzi 2009 [75]

| **Methods** | **Design** Cross-sectional |
| --- | --- |
|  | **Setting** Not specified, aside from “school settings and newspaper advertisments” |
|  | **Timing**  Diurnal rhythm |
| **Participants** | ***n=*** 154 |
|  | **Subjects** “Caucasian middle-income children (…) primarily revruited from school setting and newspaper advertisements” |
|  | **Age** 147 months (SD: 9.07 months) |
|  | **Sex** 74 boys, 80 girls |
|  | **Exclusion criteria** A history of childhood maltreatment, diagnosed psychiatric condition or significant psychological issue, changeable home situation, a history of aggression directed toward peers and/or family member, usage of psychotropic medication or oral contraceptives. |
| **Protocol** | They were asked to provide their samples 20 min after waking (between 0700 and 0800 hr on weekdays and 0900 and 1000 hr on Saturday) and at 2100 hr on Monday, Thursday and Saturday evening.  Ethical approval: “The suicide item was omitted from the CDI at the request of the research ethics board” 🡪 we therefore assume approval was obtained |
| **Outcomes** | **Primary** To examine the relationship between peer victimization and cortisol levels in a community sample of 12-year old children. |
|  | **Secondary** |
| **Results** | “A statistically significant sex difference in log(cortisol) means was present on Saturday morning (f=5.54, P<0.02) with girls having higher morning levels (M=1.90, SD=0.72) than boys (M=1.61, SD=0.80).”  “When we modeled the observed circadian pattern in the data using multilevel regression with orthogonal polynomial contrasts we found a statistically significant relation between log(cortisol) and sex; girls consistently produces higher cortisol levels than boys." |

### Vaillancourt 2008 [76]

| **Methods** | **Design** Longitudinal / cohort study |
| --- | --- |
|  | **Setting** Aalter, Belgium |
|  | **Timing**  Diurnal rhythm + CAR |
| **Participants** | ***n=*** 355 |
|  | **Subjects** Elementary school children |
|  | **Age** 5-10 years, median age for both sexes: 8 years |
|  | **Sex** Boys 186, girls 169 |
|  | **Exclusion criteria** Any children with missing data for stress-related lifestyle parameters. |
| **Protocol** | The participating children were asked to collect saliva during two consecutive weekdays at four time points: immediately on awakening (T0), 30 min after awakening (T30), 60 min after waking up (T60) and in the evening between 7 and 9 pm (Tev).  Ethical approval: obtained |
| **Outcomes** | **Primary** To examine the association between psychosocial stress and body composition in young children (5-10 years old). |
|  | **Secondary** |
| **Results** | 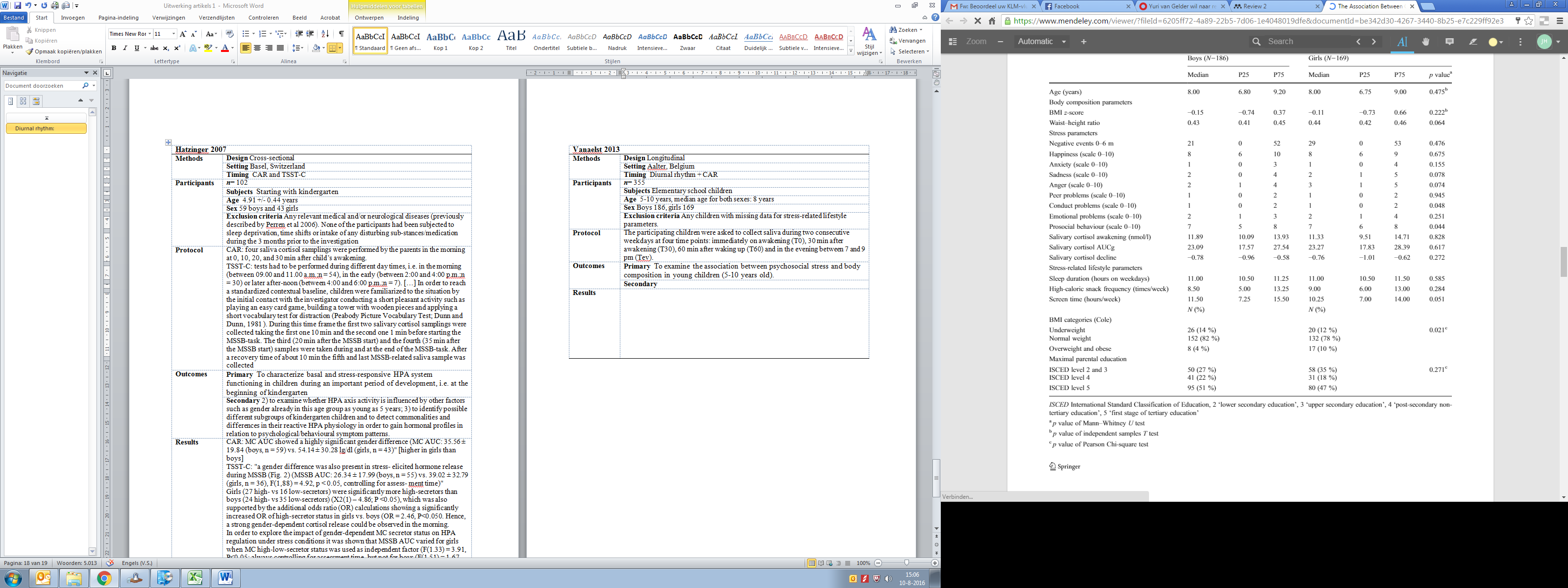  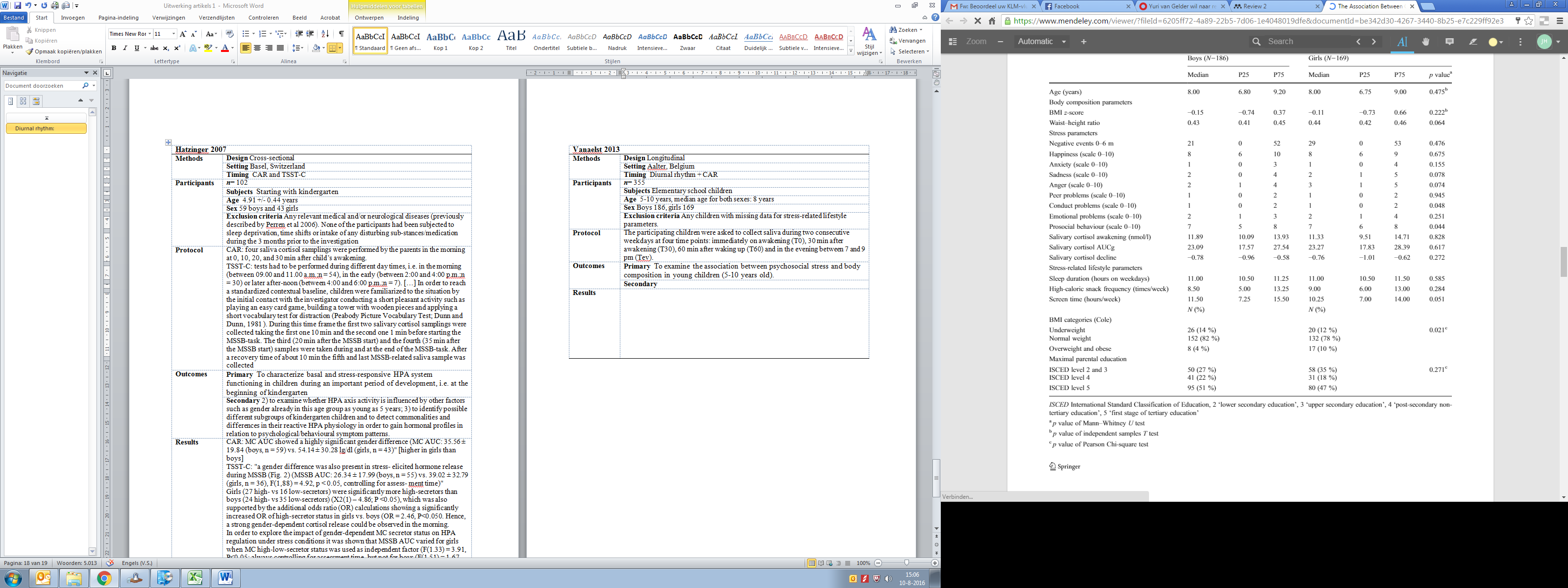  No differences were found  AUCg = CAR  Decline = morning – evening/hours between these samples |

### Vanaelst 2013 [77]

| **Methods** | **Design** cross-sectional study |
| --- | --- |
|  | **Setting** Louisville, USA |
|  | **Timing** Diurnal rhythm + CAR |
| **Participants** | ***n=*** 27 |
|  | **Subjects** “Participants were recruited through the use of flyers distributed in local schools and places of business (...) The prevalence of an anxiety diagnosis within this population was somewhat higher than what is reported in extant epidemiological studies (..) the elevated prevalence rates were likely an artefact of the recruitment flyers, which indicated that the study was exploring stress and anxiety in families.” |
|  | **Age** 9.13 ± 1.41 yrs |
|  | **Sex** 14 girls and 13 boys |
|  | **Exclusion criteria** “if the child or the mother met criteria for a primary diagnosis other than anxiety.” |
| **Protocol** | “Investigators provided instructions to participating mothers to not eat, drink, or brush their teeth 30 min prior to every sample, and to collect samples on two consecutive days, 3 times per day: at waking, 30 min after waking, and at bedtime (…) Post-waking samples were excluded if they were collected less than 15 min or more than 45 min after the waking sample (…) both the diurnal cortisol rhythm and the cortisol awakening response were calculated as the slope of change scores, using the unstandardized beta of natural-log transformed cortisol regressed on collection time. Only the waking and bedtime values were used in calculation of the diurnal cortisol slope, and only the waking and +30 values were used in calculating CAR.”  Ethical approval: “Mothers provided written informed consent for their participation in the study, as well as their child’s participation. Children provided informed assent.” |
| **Outcomes** | **Primary** “The first aim of this exploratory study is to test for synchrony between cortisol profiles of mothers and their children.” |
|  | **Secondary** “Second, we were interested in the contribution of anxiety to cortisol concentration at different times during the day. In addition, we were interested in exploring the contribution of anxiety and family environment to the independent diurnal cortisol rhythms of both mother and child.  Third, we were interested in exploring the dyadic nature of diurnal cortisol rhythm.” |
| **Results** | “Gender predicted significant differences in both actor diurnal slope and  the effect of partner diurnal slope. More specifically, male children tended to exhibit flatter diurnal slopes, and mothers of male children with flatter diurnal slopes themselves tended to exhibit flatter diurnal slopes.”  “Similar to the initial model using diurnal slope, we tested a null model using CAR slope as our dependent variable and partner CAR slope as the independent variable at Level-1. A calculation of interclass correlation indicated that, as with diurnal slope, mothers and their children possess a very strong degree of synchrony (ICC = 0.99). Following examination of random effects, it was indicated that exploring additional random effects at Level-2 was not warranted as the chi-square statistic suggested little variance remained to be accounted for by other variables (􀀁10 = 0.00009; SD = 0.01; 􀀀2(18) = 4.22, p > 0.50). Further, the null model accounted for a signiﬁcant portion in the variance (􀀂2 = 0.19), which provided support for this suggestion. No further model testing was conducted.” 🡪 no gender effects assumed |

### Williams 2013 [78]

### Yfanti 2014 [79]

| **Methods** | **Design** Prospective cohort study |
| --- | --- |
|  | **Setting** Postgraduate Clinic of Pediatric Dentistry, School of Dentistry, National and Kapodistrian University of Athens |
|  | **Timing**  Cortisol levels before and after dental treatment |
| **Participants** | ***n=*** 97 |
|  | **Subjects** Mentally and physically who presented to the Postgraduate Clinic of Pediatric Dentisty (…) for a planned dental appointment. Al had previously visited the Clinic for dental examination, and 78 of them also had a previous dental treatment experience in the same place. |
|  | **Age** 6-10 years (mean 89.73 months, SD 15) |
|  | **Sex** 58 boys, 39 girls |
|  | **Exclusion criteria** the exclusion criteria were based on factors known to influence cortisol and sAA levels, including children with active respiratory problems and children under medication for any reason, especially corticosteroids |
| **Protocol** | Upon arrival to the Clinic, parents and children were informed for the project, and after a written parent’s consent, the children were first asked to fill out the three questionnaires and then to provide the first saliva sample (within 15–20 min from  arrival). They were then brought in the treatment room where they underwent clinical examination and recording of caries lesions, followed by either a cleaning with rotary instrument or a small restorative procedure with the use of local anesthesia (articaine HCl 4% with epinephrine 1:200,000). Forty-two of the 97 children (17 girls and 25 boys) received local anesthesia during treatment. Immediately after the end of the procedure, which lasted approximately 30 min, the second saliva sample was collected. The parents were given pre-labeled collection tubes and instructions in order to collect the next two saliva samples at home, which were scheduled for the same evening (at bed time) and the next morning (within 30 min from awakening), without eating or drinking anything prior to morning sampling. (…) Patients were asked to return for a recall appointment after 7–14 d and another sample was collected at that time, within 15–20 min after  arrival and prior to dental treatment. Patients’ appointments were limited between 9 a.m. and 2 p.m.  Ethical approval: obtained |
| **Outcomes** | **Primary** to examine the neuroendocrine and autonomic nervous system responses to dental treatment and their possible interactions and associations with psychometric indices of anxiety, caries, previous dental experience, anesthesia, age and gender in school children. |
|  | **Secondary** |
| **Results** | No significant associations were detected between cortisol levels and the other variables (i.e. trait anxiety, caries, gender, age, previous dental experience or anesthesia). |

| **Yong Ping 2014** | |
| --- | --- |
| **Methods** | **Design** prospective longitudinal study |
|  | **Setting** Iowa |
|  | **Timing**  Maternal separation |
| **Participants** | ***n=*** 94 |
|  | **Subjects** “Women were eligible to participate if they were 18 years of age or over at the time of recruitment, English-speaking, and pregnant at the time of the [Iowa] ﬂood (June 15, 2008).” |
|  | **Age** 29.9±1.1 months |
|  | **Sex** 48 girls, 46 boys |
|  | **Exclusion criteria** “Ten dyads were removed from the sample due to providing insufﬁcient quantities of toddler saliva for assay (n = 9) or invalid cortisol assay results (n = 1).” |
| **Protocol** | “Maternal sep-aration commenced when a research assistant knocked on the two-way mirror, signalling for mothers to leave their toddlers alone in the monitored testing room for 2 min (M = 1.89 min, SD = 0.49).”  Cortisol samples: “Four saliva samples were collected from each toddler to assess cortisol levels throughout the procedure: a buffer sample taken 10 minutes after arriving in the laboratory; a baseline sample taken approximately 45 minutes after the buffer sample and 15 minutes before maternal separation (stressor); and two samples taken 20 and 45 minutes post-stressor (after the toddler was reunited with his or her mother).”  Ethical approval: obtained |
| **Outcomes** | **Primary** “The present study aimed to build on the existing body of literature by examining the association between PNMS and toddler cortisol reactivity.” |
|  | **Secondary** |
| **Results** | Overall: “When split by sex, results remained signiﬁcant for females from baseline to 20-minute post-stressor sampling (t(40) = −2.019, p < .05) and to 45-minute post-stressor sample (t(42) = −2.386, p < .05), but not for males (data not shown). Independent samples t-tests revealed that cortisol levels at baseline, 20-minute post-stressor, and 45-minute post-stressor did not differ signiﬁcantly between males and females.”  Baseline to 20 minute post-stressor: no association  Baseline to 45 minute post-stressor: no association  AUCi: “Toddler sex did not explain additional variance”  AUCg: no association |

### Yong Ping 2014 [80]

### Zijlmans 2013 [81]

| **Methods** | **Design** Cross-sectional |
| --- | --- |
|  | **Setting** Nijmegen, the Netherlands |
|  | **Timing**  Computer paradigm: social evaluative stress test |
| **Participants** | ***n=*** 52 |
|  | **Subjects** Children were recruited from five primary and three secondary schools in Nijmegen (The Netherlands). |
|  | **Age** 12.5 years, SD 1.21 |
|  | **Sex** 23 boys, 29 girls |
|  | **Exclusion criteria** Participants who used medication, had a diagnosis for neuropsychiatric or mental disorders, and children with recent exposure to traumatic events were excluded from the study (n=7). Furthermore, 17 children were not included in the study because of scheduling difficulties and illness on the testing day. |
| **Protocol** | Upon entering the cubicle, participants' two fingers of their non-dominant hand were connected to a "heart-rate device" connected to the computer. This device was actually a dummy, but the participant was told that it registered heart rate and that even small movements of the hand could negatively affect its working. As movements could ruin the data, the child was asked to keep this hand as still as possible. However, and irrespective of the child's actual movements, the alarm light on the screen turned on at two predetermined time points during the answering of the 36 questions (see below) to indicate inappropriate movements.  The investigator then told the child that multiple-choice questions would appear on the computer screen. These could be answered with the dominant hand by means of a button box. A panel would judge the answers as well as the participant's facial expression and posture by means of a camera next to the computer. After that, the children completed the practice session with a total of 28 easy, non-personal questions (e.g. what is the color of this flower?). Thereafter, the following instructions were displayed on the screen in Dutch.  The Dutch Film Association (DFA) has produced a brand new and thrilling 3D film. They want it to be a success! They asked our research institute to find out whether kids of your age like the film. We are looking for kids who represent the youth of today. Their opinion about the film is important to us. They will be the first kids to watch the film and give their opinion about it! From research we know that nice kids represent the youth of today best. To find out whether you are nice and represent the youth of today, we ask you to fill in personal questions on the computer. A strict panel will compare your answers with those of four other children and select the three who represent today's youth the best. Those three kids may watch the film and give their opinion. However, if you belong to the remaining two you will have to give a presentation in front of the panel telling them why you think you are nice and represent the youth. If you are able to convince the panel, you may watch the film as well.  After these instructions, salivary cortisol sample 1 (C1) was taken. To increase the credibility of the story, the photograph of the participant together with photographs of the four other "participating children," as well as a photograph of the "panel" (i.e., four adults sitting in laboratory coats behind a table, with neutral expressions on their faces) were displayed on the screen. Besides that, one of the panel members wearing a laboratory coat in the photograph entered the room before the test began to resolve a supposed "program error that was delaying the start" and introduced him/herself to the participant.  Figure 2 shows the set-up of the computer screen during the test session. The photographs on the right show the position of each child: children in the green zone would watch the movie, while children in the red zone would have to give a presentation to the panel, acting as a jury.  After the panel member had left, a total of 36 multiple-choice questions was displayed with a fixed time window of 20 s per question (the seconds remaining were shown on the screen). The questions included personal questions (i.e., addressing personal traits, ideas, hobbies, and position in life), and knowledge questions (i.e., addressing knowledge of facts with high levels of difficulty). Personal and knowledge questions were shown in a fixed design; in each block, three personal questions were followed by three knowledge questions. Prior to testing, 20 other children had evaluated the personal questions on age appropriateness, and meaningfulness. Based on their feedback, two test versions were designed: for ages of 10–12 years and 13–15 years. In total, participants were presented with six blocks of questions. After each block, intermediate judgments of the panel changed the participant's position from initially high to low. After answering all the questions, participants looked at a neutral screen for 5 min before being told that their final ranking was the fourth position and that they now had to prepare a presentation for the panel. After another 5 min, perceived stress was assessed and the stress test ended with the debriefing of the participant. In the debriefing, all participants were told that they did not actually have to present in front of the jury. Additionally, they were informed that the SEST was preprogrammed in such a way that it was impossible to win the competition. The SEST was programmed using E-prime version 1.0 (Schneider et al., 2002 Schneider W, Eschman A, Zuccolotto A. (2002). E-Prime (Version 1.0) [Computer Software]. Pittsburgh, PA: Psychology Software Tools, Inc), and was designed to exclude speech and movement in the participant. In this way, it is compatible for use during future neuroimaging sessions. Moreover, the experimental design will enable explicit allocation of neural activation to specific test elements: the control condition (i.e., training session without social evaluation and including only easy, non-personal, knowledge questions), the test session with personal questions, and the test session with knowledge questions.  In sum, the SEST contains elements of social evaluation (i.e., picture of jury, comparison with other children, several judging moments, camera), unpredictability (i.e., unknown paradigm) and of uncontrollability (i.e., "heart-rate device" giving an alarm signal twice irrespective of movements, time pressure for answering questions, falling in the ranking, too difficult questions). In this way, the paradigm capitalized on the elements that are known to induce stress in children and adults in laboratory settings.  Seven saliva samples were taken to obtain cortisol measurements of pre-stress, stress, and recovery concentrations. The saliva samples (C0–C6) were obtained by having the participants spit through a short straw into a 2.5-ml tube at – 45, 5, 30, 40, 50, 60, and 80 min relative to the onset of the computerized paradigm  Ethical approval: obtained |
| **Outcomes** | **Primary** This study investigated Cortisol responses and perceived stress of 10–15-year-olds to a computerized paradigm including elements of social evaluation, unpredictability, and uncontrollability. Both age and sex differences were examined. |
|  | **Secondary** |
| **Results** | Independent sample t-tests showed that the time of testing occurred significantly later for girls than boys (t(50) = −2.48, p < 0.05). Also, the older children were tested significantly later compared to the younger age group (t(50) = −2.20, p < 0.05). Boys showed significantly higher actual cortisol concentration increases (delta) than girls (t(50) = −3.49, p < 0.001  Regarding sex differences, for boys, paired samples t-tests showed a significant increase in cortisol concentration (t(22) = −4.04, p < 0.001, d = 1.22), followed by a significant decrease in cortisol concentration (t(22) = 4.37, p < 0.001, d = 0.82). For girls, the increase in cortisol showed only a trend for significance (t(28) = −1.88, p = 0.07, d = 0.27), whereas the ensuing decrease in cortisol concentration was significant (t(28) = 3.54, p < 0.001, d = 0.56).  Figure 4 shows the course of the cortisol concentrations separately for girls and boys. Independent samples t-tests showed that boys had significantly higher cortisol values than girls at C3 (t(33.43) = 3.06, p < 0.05, d = 0.93), C4 (t(30.04) = 2.70, p < 0.05, d = 0.83), C5 (t(34.30) = 2.82, p < 0.05, d = 0.85), and C6 (t(50) = 2.80, p < 0.05, d = 0.80).  The correlations (Table 2) showed that the actual increase in cortisol concentration (delta) was not correlated with child age, but was significantly correlated with child sex; boys reacted with significantly higher increases in cortisol concentration than girls (r(50) = −0.39, p < 0.01).  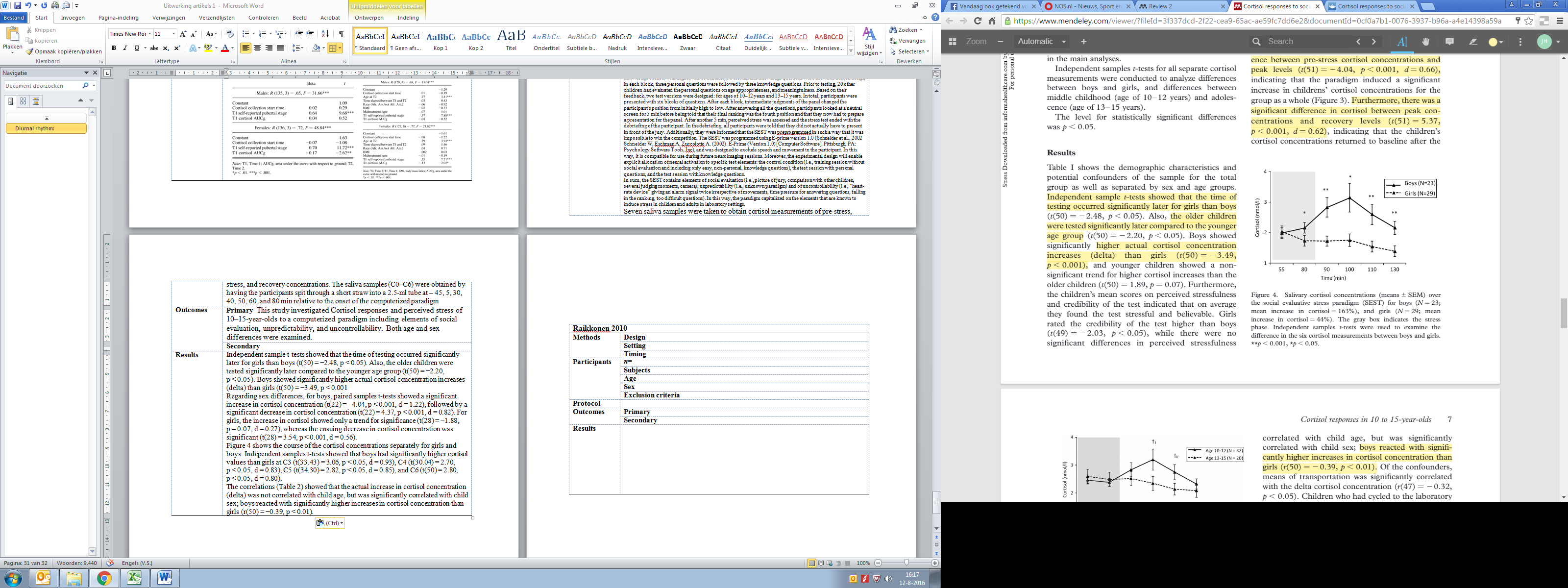 |

References

1. Adam EK, Doane LD, Zinbarg RE, Mineka S, Craske MG, Griffith JW: **Prospective prediction of major depressive disorder from cortisol awakening responses in adolescence.** *Psychoneuroendocrinology* 2010, **35:**921-931.

2. Allen LB, Lu Q, Tsao JC, Worthman CM, Zeltzer LK: **Sex differences in the association between cortisol concentrations and laboratory pain responses in healthy children.** *Gend Med* 2009, **6 Suppl 2:**193-207.

3. Bae YJ, Stadelmann S, Klein AM, Jaeger S, Hiemisch A, Kiess W, Ceglarek U, Gaudl A, Schaab M, von Klitzing K, et al: **The hyporeactivity of salivary cortisol at stress test (TSST-C) in children with internalizing or externalizing disorders is contrastively associated with alpha-amylase.** *J Psychiatr Res* 2015, **71:**78-88.

4. Barbosa TS, Castelo PM, Leme MS, Gaviao MB: **Associations between oral health-related quality of life and emotional statuses in children and preadolescents.** *Oral Dis* 2012, **18:**639-647.

5. Bartels M, de Geus EJ, Kirschbaum C, Sluyter F, Boomsma DI: **Heritability of daytime cortisol levels in children.** *Behav Genet* 2003, **33:**421-433.

6. Bouma EM, Riese H, Ormel J, Verhulst FC, Oldehinkel AJ: **Adolescents' cortisol responses to awakening and social stress; effects of gender, menstrual phase and oral contraceptives. The TRAILS study.** *Psychoneuroendocrinology* 2009, **34:**884-893.

7. Bouma EM, Riese H, Nolte IM, Oosterom E, Verhulst FC, Ormel J, Oldehinkel AJ: **No associations between single nucleotide polymorphisms in corticoid receptor genes and heart rate and cortisol responses to a standardized social stress test in adolescents: the TRAILS study.** *Behav Genet* 2011, **41:**253-261.

8. Bright MA, Frick JE, Out D, Granger DA: **Individual differences in the cortisol and salivary alpha-amylase awakening responses in early childhood: relations to age, sex, and sleep.** *Dev Psychobiol* 2014, **56:**1300-1315.

9. Carrion VG, Weems CF, Ray RD, Glaser B, Hessl D, Reiss AL: **Diurnal salivary cortisol in pediatric posttraumatic stress disorder.** *Biol Psychiatry* 2002, **51:**575-582.

10. Chiodo S, Tessitore A, Cortis C, Cibelli G, Lupo C, Ammendolia A, De Rosas M, Capranica L: **Stress-related hormonal and psychological changes to official youth Taekwondo competitions.** *Scand J Med Sci Sports* 2011, **21:**111-119.

11. Covelli MM, Wood CE, Yarandi HN: **Biologic measures as epidemiological indicators of risk for the development of hypertension in an African American adolescent population.** *J Cardiovasc Nurs* 2012, **27:**476-484.

12. Dahl RE, Siegel SF, Williamson DE, Lee PA, Perel J, Birmaher B, Ryan ND: **Corticotropin releasing hormone stimulation test and nocturnal cortisol levels in normal children.** *Pediatr Res* 1992, **32:**64-68.

13. Daughters SB, Gorka SM, Matusiewicz A, Anderson K: **Gender specific effect of psychological stress and cortisol reactivity on adolescent risk taking.** *J Abnorm Child Psychol* 2013, **41:**749-758.

14. Davis M, Emory E: **Sex differences in neonatal stress reactivity.** *Child Dev* 1995, **66:**14-27.

15. de Veld DM, Riksen-Walraven JM, de Weerth C: **The relation between emotion regulation strategies and physiological stress responses in middle childhood.** *Psychoneuroendocrinology* 2012, **37:**1309-1319.

16. de Weerth C, Zijlmans MA, Mack S, Beijers R: **Cortisol reactions to a social evaluative paradigm in 5- and 6-year-old children.** *Stress* 2013, **16:**65-72.

17. Dietrich A, Ormel J, Buitelaar JK, Verhulst FC, Hoekstra PJ, Hartman CA: **Cortisol in the morning and dimensions of anxiety, depression, and aggression in children from a general population and clinic-referred cohort: An integrated analysis. The TRAILS study.** *Psychoneuroendocrinology* 2013, **38:**1281-1298.

18. Dockray S, Susman EJ, Dorn LD: **Depression, cortisol reactivity, and obesity in childhood and adolescence.** *J Adolesc Health* 2009, **45:**344-350.

19. Doom JR, Cicchetti D, Rogosch FA, Dackis MN: **Child maltreatment and gender interactions as predictors of differential neuroendocrine profiles.** *Psychoneuroendocrinology* 2013, **38:**1442-1454.

20. Dorn LD, Burgess ES, Susman EJ, von Eye A, DeBellis MD, Gold PW, Chrousos GP: **Response to oCRH in depressed and nondepressed adolescents: does gender make a difference?** *J Am Acad Child Adolesc Psychiatry* 1996, **35:**764-773.

21. Eiden RD, Molnar DS, Granger DA, Colder CR, Schuetze P, Huestis MA: **Prenatal tobacco exposure and infant stress reactivity: role of child sex and maternal behavior.** *Dev Psychobiol* 2015, **57:**212-225.

22. Evans BE, Greaves-Lord K, Euser AS, Tulen JH, Franken IH, Huizink AC: **Determinants of physiological and perceived physiological stress reactivity in children and adolescents.** *PLoS One* 2013, **8:**e61724.

23. Forest MG: **Age-related response of plasma testosterone, delta 4-androstenedione, and cortisol to adrenocorticotropin in infants, children, and adults.** *J Clin Endocrinol Metab* 1978, **47:**931-937.

24. Fransson E, Folkesson L, Bergstrom M, Ostberg V, Lindfors P: **Exploring salivary cortisol and recurrent pain in mid-adolescents living in two homes.** *BMC Psychol* 2014, **2:**46.

25. Frias J, Rodriguez R, Torres JM, Ruiz E, Ortega E: **Effects of acute alcohol intoxication on pituitary-gonadal axis hormones, pituitary-adrenal axis hormones, beta-endorphin and prolactin in human adolescents of both sexes.** *Life Sci* 2000, **67:**1081-1086.

26. Garcia L, Hermida RC, Ayala DE, Lodeiro C, Iglesias T: **Circadian characteristics of plasma cortisol in children with standard and short stature.** *Chronobiol Int* 1990, **7:**221-225.

27. Gecgelen M, Aksoy A, Kirdemir P, Doguc DK, Cesur G, Koskan O, Ozorak O: **Evaluation of stress and pain during rapid maxillary expansion treatments.** *J Oral Rehabil* 2012, **39:**767-775.

28. Grunau RE, Tu MT, Whitfield MF, Oberlander TF, Weinberg J, Yu W, Thiessen P, Gosse G, Scheifele D: **Cortisol, behavior, and heart rate reactivity to immunization pain at 4 months corrected age in infants born very preterm.** *Clin J Pain* 2010, **26:**698-704.

29. Gunnar MR, Wewerka S, Frenn K, Long JD, Griggs C: **Developmental changes in hypothalamus-pituitary-adrenal activity over the transition to adolescence: normative changes and associations with puberty.** *Dev Psychopathol* 2009, **21:**69-85.

30. Gunnar MR, Kryzer E, Van Ryzin MJ, Phillips DA: **The rise in cortisol in family day care: associations with aspects of care quality, child behavior, and child sex.** *Child Dev* 2010, **81:**851-869.

31. Hackman DA, Betancourt LM, Brodsky NL, Hurt H, Farah MJ: **Neighborhood disadvantage and adolescent stress reactivity.** *Front Hum Neurosci* 2012, **6:**277.

32. Haen EH, F.; Cornelissen, G.: **Cortisol marker rhythmometry in pediatrics and clinical pharmacology.** *Annual Review of Chronopharmacology* 1984, **1:**165-168.

33. Hatzinger M, Brand S, Perren S, von Wyl A, von Klitzing K, Holsboer-Trachsler E: **Hypothalamic-pituitary-adrenocortical (HPA) activity in kindergarten children: importance of gender and associations with behavioral/emotional difficulties.** *J Psychiatr Res* 2007, **41:**861-870.

34. Hostinar CE, McQuillan MT, Mirous HJ, Grant KE, Adam EK: **Cortisol responses to a group public speaking task for adolescents: variations by age, gender, and race.** *Psychoneuroendocrinology* 2014, **50:**155-166.

35. Hostinar CE, Johnson AE, Gunnar MR: **Parent support is less effective in buffering cortisol stress reactivity for adolescents compared to children.** *Dev Sci* 2015, **18:**281-297.

36. Ji J, Negriff S, Kim H, Susman EJ: **A study of cortisol reactivity and recovery among young adolescents: Heterogeneity and longitudinal stability and change.** *Dev Psychobiol* 2016, **58:**283-302.

37. Jones A, Godfrey KM, Wood P, Osmond C, Goulden P, Phillips DI: **Fetal growth and the adrenocortical response to psychological stress.** *J Clin Endocrinol Metab* 2006, **91:**1868-1871.

38. Kelly SJ, Young R, Sweeting H, Fischer JE, West P: **Levels and confounders of morning cortisol collected from adolescents in a naturalistic (school) setting.** *Psychoneuroendocrinology* 2008, **33:**1257-1268.

39. Khilnani P, Munoz R, Salem M, Gelb C, Todres ID, Chernow B: **Hormonal responses to surgical stress in children.** *J Pediatr Surg* 1993, **28:**1-4.

40. Kjolhede EA, Gustafsson PE, Gustafsson PA, Nelson N: **Overweight and obese children have lower cortisol levels than normal weight children.** *Acta Paediatr* 2014, **103:**295-299.

41. Knutsson U, Dahlgren J, Marcus C, Rosberg S, Bronnegard M, Stierna P, Albertsson-Wikland K: **Circadian cortisol rhythms in healthy boys and girls: relationship with age, growth, body composition, and pubertal development.** *J Clin Endocrinol Metab* 1997, **82:**536-540.

42. Kryski KR, Smith HJ, Sheikh HI, Singh SM, Hayden EP: **HPA axis reactivity in early childhood: associations with symptoms and moderation by sex.** *Psychoneuroendocrinology* 2013, **38:**2327-2336.

43. Kudielka BM, Buske-Kirschbaum A, Hellhammer DH, Kirschbaum C: **HPA axis responses to laboratory psychosocial stress in healthy elderly adults, younger adults, and children: impact of age and gender.** *Psychoneuroendocrinology* 2004, **29:**83-98.

44. Kuhlman KR, Geiss EG, Vargas I, Lopez-Duran NL: **Differential associations between childhood trauma subtypes and adolescent HPA-axis functioning.** *Psychoneuroendocrinology* 2015, **54:**103-114.

45. Lashansky G, Saenger P, Fishman K, Gautier T, Mayes D, Berg G, Di Martino-Nardi J, Reiter E: **Normative data for adrenal steroidogenesis in a healthy pediatric population: age- and sex-related changes after adrenocorticotropin stimulation.** *J Clin Endocrinol Metab* 1991, **73:**674-686.

46. Lopez-Duran NL, McGinnis E, Kuhlman K, Geiss E, Vargas I, Mayer S: **HPA-axis stress reactivity in youth depression: evidence of impaired regulatory processes in depressed boys.** *Stress* 2015, **18:**545-553.

47. Lu Q, Tao F, Hou F, Zhang Z, Sun Y, Xu Y, Xu S, Zhao Y: **Cortisol reactivity, delay discounting and percent body fat in Chinese urban young adolescents.** *Appetite* 2014, **72:**13-20.

48. Lumeng JC, Miller A, Peterson KE, Kaciroti N, Sturza J, Rosenblum K, Vazquez DM: **Diurnal cortisol pattern, eating behaviors and overweight in low-income preschool-aged children.** *Appetite* 2014, **73:**65-72.

49. Martikainen S, Pesonen AK, Lahti J, Heinonen K, Feldt K, Pyhala R, Tammelin T, Kajantie E, Eriksson JG, Strandberg TE, Raikkonen K: **Higher levels of physical activity are associated with lower hypothalamic-pituitary-adrenocortical axis reactivity to psychosocial stress in children.** *J Clin Endocrinol Metab* 2013, **98:**E619-627.

50. Martin A, Hellhammer J, Hero T, Max H, Schult J, Terstegen L: **Effective prevention of stress-induced sweating and axillary malodour formation in teenagers.** *Int J Cosmet Sci* 2011, **33:**90-97.

51. Matchock RL, Dorn LD, Susman EJ: **Diurnal and seasonal cortisol, testosterone, and DHEA rhythms in boys and girls during puberty.** *Chronobiol Int* 2007, **24:**969-990.

52. Michels N, Sioen I, Huybrechts I, Bammann K, Vanaelst B, De Vriendt T, Iacoviello L, Konstabel K, Ahrens W, De Henauw S: **Negative life events, emotions and psychological difficulties as determinants of salivary cortisol in Belgian primary school children.** *Psychoneuroendocrinology* 2012, **37:**1506-1515.

53. Mills RS, Imm GP, Walling BR, Weiler HA: **Cortisol reactivity and regulation associated with shame responding in early childhood.** *Dev Psychol* 2008, **44:**1369-1380.

54. Minkley N, Kirchner WH: **Influence of test tasks with different cognitive demands on salivary cortisol concentrations in school students.** *Int J Psychophysiol* 2012, **86:**245-250.

55. Morin-Major JK, Marin MF, Durand N, Wan N, Juster RP, Lupien SJ: **Facebook behaviors associated with diurnal cortisol in adolescents: Is befriending stressful?** *Psychoneuroendocrinology* 2016, **63:**238-246.

56. Mrug S, Tyson A, Turan B, Granger DA: **Sleep problems predict cortisol reactivity to stress in urban adolescents.** *Physiol Behav* 2016, **155:**95-101.

57. Netherton C, Goodyer I, Tamplin A, Herbert J: **Salivary cortisol and dehydroepiandrosterone in relation to puberty and gender.** *Psychoneuroendocrinology* 2004, **29:**125-140.

58. Osika W, Friberg P, Wahrborg P: **A new short self-rating questionnaire to assess stress in children.** *Int J Behav Med* 2007, **14:**108-117.

59. Peckins MK, Dockray S, Eckenrode JL, Heaton J, Susman EJ: **The longitudinal impact of exposure to violence on cortisol reactivity in adolescents.** *J Adolesc Health* 2012, **51:**366-372.

60. Plusquellec P, Ouellet-Morin I, Feng B, Perusse D, Tremblay RE, Lupien SJ, Boivin M: **Salivary cortisol levels are associated with resource control in a competitive situation in 19 month-old boys.** *Horm Behav* 2011, **60:**159-164.

61. Portnoy J, Raine A, Glenn AL, Chen FR, Choy O, Granger DA: **Digit ratio (2D:4D) moderates the relationship between cortisol reactivity and self-reported externalizing behavior in young adolescent males.** *Biol Psychol* 2015, **112:**94-106.

62. Pruessner JC, Wolf OT, Hellhammer DH, Buske-Kirschbaum A, von Auer K, Jobst S, Kaspers F, Kirschbaum C: **Free cortisol levels after awakening: a reliable biological marker for the assessment of adrenocortical activity.** *Life Sci* 1997, **61:**2539-2549.

63. Raikkonen K, Matthews KA, Pesonen AK, Pyhala R, Paavonen EJ, Feldt K, Jones A, Phillips DI, Seckl JR, Heinonen K, et al: **Poor sleep and altered hypothalamic-pituitary-adrenocortical and sympatho-adrenal-medullary system activity in children.** *J Clin Endocrinol Metab* 2010, **95:**2254-2261.

64. Rosmalen JG, Oldehinkel AJ, Ormel J, de Winter AF, Buitelaar JK, Verhulst FC: **Determinants of salivary cortisol levels in 10-12 year old children; a population-based study of individual differences.** *Psychoneuroendocrinology* 2005, **30:**483-495.

65. Ross JL, Schulte HM, Gallucci WT, Cutler GB, Jr., Loriaux DL, Chrousos GP: **Ovine corticotropin-releasing hormone stimulation test in normal children.** *J Clin Endocrinol Metab* 1986, **62:**390-392.

66. Ruttle PL, Javaras KN, Klein MH, Armstrong JM, Burk LR, Essex MJ: **Concurrent and longitudinal associations between diurnal cortisol and body mass index across adolescence.** *J Adolesc Health* 2013, **52:**731-737.

67. Shirtcliff EA, Allison AL, Armstrong JM, Slattery MJ, Kalin NH, Essex MJ: **Longitudinal stability and developmental properties of salivary cortisol levels and circadian rhythms from childhood to adolescence.** *Dev Psychobiol* 2012, **54:**493-502.

68. Spinrad TL, Eisenberg N, Granger DA, Eggum ND, Sallquist J, Haugen RG, Kupfer A, Hofer C: **Individual differences in preschoolers' salivary cortisol and alpha-amylase reactivity: relations to temperament and maladjustment.** *Horm Behav* 2009, **56:**133-139.

69. Strahler J, Mueller A, Rosenloecher F, Kirschbaum C, Rohleder N: **Salivary alpha-amylase stress reactivity across different age groups.** *Psychophysiology* 2010, **47:**587-595.

70. Stroud LR, Papandonatos GD, Williamson DE, Dahl RE: **Sex differences in cortisol response to corticotropin releasing hormone challenge over puberty: Pittsburgh Pediatric Neurobehavioral Studies.** *Psychoneuroendocrinology* 2011, **36:**1226-1238.

71. Stupnicki R, Obminski Z, Klusiewicz A, Viru A: **Pre-exercise serum cortisol concentration and responses to laboratory exercise.** *Eur J Appl Physiol Occup Physiol* 1995, **71:**439-443.

72. Susman EJ, Dockray S, Schiefelbein VL, Herwehe S, Heaton JA, Dorn LD: **Morningness/eveningness, morning-to-afternoon cortisol ratio, and antisocial behavior problems during puberty.** *Dev Psychol* 2007, **43:**811-822.

73. Trickett PK, Gordis E, Peckins MK, Susman EJ: **Stress reactivity in maltreated and comparison male and female young adolescents.** *Child Maltreat* 2014, **19:**27-37.

74. Tsvetkova V: **Adrenocortical function after stimulation with synthetic ACTH.** *Curr Med Res Opin* 1977, **4:**635-639.

75. Tzortzi C, Proff P, Redlich M, Aframian DJ, Palmon A, Golan I, Muessig D, Wichelhaus A, Baumert U: **Cortisol daily rhythm in saliva of healthy school children.** *Int Dent J* 2009, **59:**12-18.

76. Vaillancourt T, Duku E, Decatanzaro D, Macmillan H, Muir C, Schmidt LA: **Variation in hypothalamic-pituitary-adrenal axis activity among bullied and non-bullied children.** *Aggress Behav* 2008, **34:**294-305.

77. Vanaelst B, Michels N, Clays E, Herrmann D, Huybrechts I, Sioen I, Vyncke K, De Henauw S: **The association between childhood stress and body composition, and the role of stress-related lifestyle factors--cross-sectional findings from the baseline ChiBSD survey.** *Int J Behav Med* 2014, **21:**292-301.

78. Williams SR, Cash E, Daup M, Geronimi EM, Sephton SE, Woodruff-Borden J: **Exploring patterns in cortisol synchrony among anxious and nonanxious mother and child dyads: a preliminary study.** *Biol Psychol* 2013, **93:**287-295.

79. Yfanti K, Kitraki E, Emmanouil D, Pandis N, Papagiannoulis L: **Psychometric and biohormonal indices of dental anxiety in children. A prospective cohort study.** *Stress* 2014, **17:**296-304.

80. Yong Ping E, Laplante DP, Elgbeili G, Hillerer KM, Brunet A, O'Hara MW, King S: **Prenatal maternal stress predicts stress reactivity at 2(1/2) years of age: the Iowa Flood Study.** *Psychoneuroendocrinology* 2015, **56:**62-78.

81. Zijlmans MA, Beijers R, Mack S, Pruessner JC, de Weerth C: **Cortisol responses to social evaluation in 10- to 15-year-old boys and girls.** *Stress* 2013, **16:**393-401.
